# Sequence Inversion Dictates the Antiproliferative Activity of Bidirectional Tryptophan‐Containing Dipeptide Libraries

**DOI:** 10.1002/psc.70128

**Published:** 2026-09-16

**Authors:** J. Benamer Díaz, Adam N. Khan, Aday González‐Bakker, José M. Padrón

**Affiliations:** ^1^ BioLab, Instituto Universitario de Bio‐Orgánica Antonio González Universidad de La Laguna La Laguna Spain

**Keywords:** anticancer drugs, antiproliferative activity, peptide sequence inversion, phenotypic screening, structure–activity relationships (SAR), tryptophan dipeptides

## Abstract

Two complementary series of novel tryptophan‐derived dipeptides (Trp‐X and X‐Trp) were designed and synthesized to systematically evaluate how backbone sequence inversion alters chemical accessibility and in vitro antineoplastic profiles against a human solid tumor cell line panel (A549, HeLa, MIA PaCa‐2, SW1573, T‐47D, and WiDr). Reversing the peptide connectivity revealed prominent sequence‐dependent chemical reactivities and biological variations. Steric repulsion at the β‐carbon limited basic hydrolysis during X‐Trp precursor assembly, whereas the Trp‐X series allowed straightforward chemical couplings. Phenotypic screening demonstrated that the Trp‐X configuration is biologically superior to the X‐Trp layout. The conformationally restricted L‐proline conjugate (Trp‐Pro) emerged as the lead architecture, exhibiting consistent, single‐digit sub‐micromolar growth inhibition across all histotypes (GI_50_ = 1.36–2.06 μM) and effectively outperforming clinical standards cisplatin and 5‐fluorouracil in resistant models. Interestingly, an inversion of structure–activity relationships was observed in the reverse series, where specific residues like L‐phenylglycine and L‐tyrosine experienced a prominent rescue of potency upon sequence relocation. These results confirm that the antiproliferative profile of these peptidomimetics is strictly dictated by a highly directional, sequence‐specific molecular topology rather than aggregate lipophilicity alone.

## Graphical abstract

Reversing the peptide backbone sequence of novel tryptophyl‐dipeptides uncovers profound changes in synthetic accessibility and antineoplastic profiles against human solid tumors. Phenotypic screening identifies a conformationally restricted lead architecture that achieves sub‐micromolar growth inhibition, confirming sequence topology as a critical regulatory determinant in small‐molecule drug discovery.

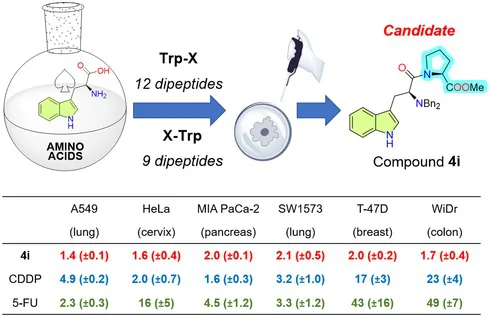

## 1 Introduction

The identification of new antineoplastic agents remains a major challenge in medicinal chemistry, particularly for highly aggressive solid tumors characterized by a poor prognosis and limited therapeutic options. Despite advances in chemotherapy and targeted therapies, treatment resistance and severe side effects continue to compromise clinical outcomes, underscoring the urgent need for novel compounds with improved efficacy and selectivity. In this context, bioactive amino acids and short peptides have attracted increasing attention due to their potential to interfere with cancer cell metabolism and proliferation through multiple mechanisms [1, 2].

Dipeptides, defined as molecules composed of two amino acids linked by a peptide bond, represent minimal yet highly versatile molecular entities for drug discovery. Their low molecular weight, favorable solubility profiles, and structural simplicity allow fine‐tuning of physicochemical and biological properties through rational modification. Moreover, dipeptides have been reported to act as privileged scaffolds capable of modulating a wide range of biological targets, including enzymes, transporters, and signaling pathways, making them attractive candidates for pharmacological development [3]. In recent years, short peptides and peptidomimetics have emerged as highly efficient tools in oncology due to their superior tissue penetration, low toxicity in non‐transformed cells, and ability to disrupt cancer cell membranes or specifically block intracellular survival pathways [4].

**FIGURE 1:**
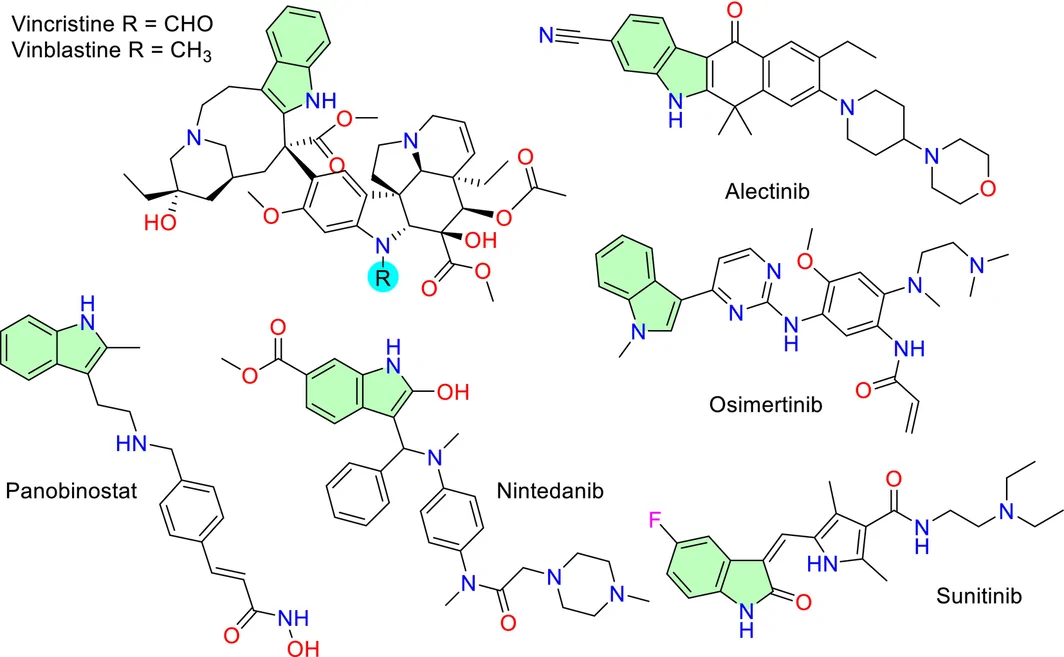
Approved anticancer drugs with an indole scaffold (highlighted).

Among proteinogenic amino acids, L‐tryptophan occupies a prominent position in medicinal chemistry due to the presence of the indole moiety, a structural motif frequently encountered in bioactive natural products and approved drugs. The indole ring is known to engage in π–π stacking, hydrogen bonding, and hydrophobic interactions, thereby playing a crucial role in molecular recognition processes. Indole derivatives serve as a versatile scaffold in modern drug discovery, demonstrating a remarkable ability to target diverse biological pathways [5]. The clinical translation of the indole scaffold is well established across diverse oncological modalities, serving as a core pharmacophoric framework for both classic chemotherapeutics and modern targeted agents. This privileged architecture is prominently featured in the antimitotic *Vinca* alkaloids [6], such as vincristine and vinblastine (Figure 1), which disrupt microtubule dynamics during cell division. Furthermore, small‐molecule kinase inhibitors heavily exploit this versatile core, as demonstrated by the multi‐targeted receptor tyrosine kinase inhibitor sunitinib used in renal cell carcinoma and gastrointestinal stromal tumors [7], the selective ALK inhibitor alectinib for advanced non‐small cell lung cancer [8], and nintedanib, a potent angiokinase inhibitor indicated for specific lung and adenocarcinoma profiles [9]. Additionally, the indole ring is central to the epigenetic mechanism of panobinostat, a histone deacetylase inhibitor utilized in multiple myeloma regimens [10], further reinforcing the adaptable nature of this scaffold in steering contemporary anticancer drug discovery.

Numerous tryptophan‐derived compounds have demonstrated anticancer, antimicrobial, and neuroactive properties, highlighting the versatility of this amino acid as a building block for drug design [11]. In addition, diverse studies have shown that the incorporation of tryptophan into short peptides can significantly enhance biological activity, particularly when combined with strategic *N*‐terminal or side‐chain modifications that increase lipophilicity or modulate polarity. Such modifications may improve membrane permeability, interaction with amino acid transporters, and intracellular accumulation, all of which are relevant for anticancer activity [1, 2]. In this regard, dipeptides containing tryptophan offer an ideal framework for exploring structure–activity relationships while maintaining synthetic accessibility.

In this context, our group previously explored a targeted library of glutamic acid‐based dipeptides, identifying lead compounds with potent antiproliferative activity against human solid tumor cell lines. Notably, the tryptophan‐containing dipeptides within that library displayed a remarkable cell line‐selective growth inhibition [11]. They actively suppressed proliferation in T‐47D breast cancer cells but remained completely inactive against HBL‐100 cells. This selective behavior was attributed to the differential expression of the ATB^0,+^ (SLC6A14) transporter, an amino acid carrier markedly upregulated in specific tumors that accepts tryptophan as a high‐affinity substrate. Although subsequent investigations could not definitively establish ATB^0,+^ as the primary target for these compounds, the tryptophan core remained highly worth exploring due to its clear involvement in driving cell line selectivity.

Inspired by these intriguing findings and the privileged role of the indole moiety in molecular recognition, we herein describe the design, synthesis, and biological evaluation against human solid tumor cell lines of a novel series of tryptophan‐derived dipeptides aimed at identifying novel candidates for anticancer drug discovery programs.

## 2 Materials and Methods

### 2.1 Chemistry

Unless otherwise specified, reagents from commercial sources were used as received. Solvents were dried and purified by conventional methods prior to use. Cell culture medium, supplements, reagents, and sterile plasticware for biological assays were purchased from commercial suppliers.

#### 2.1.1 Synthesis of Methyl *N*,*N*‐Dibenzyl‐L‐Tryptophanate (**2**)

To a solution of commercially available L‐tryptophan methyl ester hydrochloride (4.6 mmol), sodium bicarbonate (16 mmol) and TBAI (2.3 mmol) in a tetrahydrofuran‐dimethyl sulfoxide (THF/DMSO) 4:1 mixture (25 mL), benzyl bromide (11.4 mmol) was added dropwise. The reaction mixture was stirred at reflux overnight. After this time, the reaction mixture was diluted with diethyl ether (25 mL) and the organic layer was washed with water (50 mL), saturated sodium bicarbonate solution (50 mL), brine (50 mL), dried over Na_2_SO_4_, and filtered. The solvent was removed under vacuum and the residue was purified using flash column chromatography to give the corresponding product **2**. Yield: 74%, amorphous white solid. ^1^H NMR (500 MHz, CDCl_3_) δ 7.97 (s, 1H), 7.44–7.40 (t, 4H), 7.37–7.29 (m, 7H), 7.21 (d, 2H), 7.03 (t, 1H), 6.09 (s, 1H), 4.12 (d, 2H), 3.92–3.89 (m, 1H), 3.75 (s, 3H), 3.65–3.63 (d, 2H), 3.49–3.45 (m, 1H), 3.19–3.16 (dd, 1H). ^13^C NMR (125 MHz, CDCl_3_) δ 173.08, 139.6, 136.2, 128.9, 128.6, 128.3, 127.5, 127.05, 122.8, 121.8, 119.2, 118.7, 112.0, 111.0, 61.5, 54.7, 51.0, 26.3. HRMS S (ESI‐TOF): calculated for C_26_H_26_N_2_O_2_ [M + H^+^] 399.1994, found: 399.1888. [α]^25^_D_: −55.01° (c = 0.1, CHCl_3_).

#### 2.1.2 Synthesis of *N*,*N*‐Dibenzyl‐L‐Tryptophan (**3**)

To a solution of **2** (2.5 mmol), in THF/MeOH/H_2_O 2:1:1 (50 mL), LiOH (12.6 mmol) was added and the reaction mixture was stirred at reflux overnight. The reaction mixture was extracted with diethyl ether (3 × 25 mL) and the combined organic layer was washed with sodium bicarbonate saturated solution (50 mL), brine (50 mL), dried over Na_2_SO_4_ and filtered. The solvent was removed under vacuum to give **3**. Yield: 81%, brown clear amorphous solid. ^1^H NMR (500 MHz, CD_3_OD) δ 7.57–7.09 (m, 15H), 4.20–4.17 (d, 2H), 4.09–4.06 (t, 1H), 4.02–3.99 (d, 2H), 3.68–3.64 (m, 1H), 3.42–3.38 (m, 1H). ^13^C NMR (125 MHz, CDCl_3_) δ 173.64, 137.62, 136.73, 128.78, 128.05, 127.19, 123.07, 120.94, 118.26, 118.00, 110.82, 110.52, 62.27, 54.34, 24.95. HRMS S (ESI‐TOF): calculated for C_25_H_24_N_2_O_2_ [M + H^+^] 385.1838, found: 385.1913. [α]^25^_D_: −42.00° (c = 0.1, CHCl_3_).

#### 2.1.3 General Procedure for the Synthesis of Tryptophyl‐X Dipeptides

To a solution of **3** (0.26 mmol), the appropriate amino acid methyl ester (0.52 mmol), Et_3_N (0.52 mmol) and HOBt (0.286 mmol) in DCM (20 mL) at 0°C, 1‐ethyl‐3‐(3‐dimethylaminopropyl) carbodiimide hydrochloride (0.286 mmol) was added in portions and the reaction mixture was stirred at room temperature overnight. The solvent was removed under vacuum and the crude was reconstituted with AcOEt (50 mL). The organic layer was washed with 0.5 M citric acid solution (50 mL), sodium bicarbonate saturated solution (50 mL), brine (50 mL), dried over Na_2_SO_4_, and filtered. The solvent was removed under vacuum and the residue was purified using flash column chromatography.

Methyl *N*,*N*‐dibenzyl‐L‐tryptophyl‐glycinate (**4a**): Yield: 60%, colorless oil. ^1^H NMR (500 MHz, CDCl_3_) δ 8,06–6,94 (m, 16H), 4,20–4,16 (dd, 1H), 3,87–3,77 (m, 8H), 3,68 (t, 1H), 3,56–3,52 (dd, 1H), 3,27–3,23 (dd, 1H). ^13^C NMR (125 MHz, CDCl_3_) δ 172.99, 170.30, 139.27, 136.20, 128.78, 128.46, 127.31, 127.19, 123.20, 121.91, 119.23, 118.83, 113.35, 111.11, 62.51, 54.49, 52.27, 41.15, 21.96. HRMS S (ESI‐TOF): calculated for C_28_H_29_N_3_O_3_ [M + Na^+^] 478.2209, found: 478.2111. [α]^25^_D_: −13.6° (c = 0.1, CHCl_3_).

Methyl *N*,*N*‐dibenzyl‐L‐tryptophyl‐L‐alaninate (**4b**): Yield: 85%, yellow oil. ^1^H NMR (500 MHz, CDCl_3_) δ 8.11 (s, 1H), 7.50–7.04 (m, 15H), 4.53 (t, 1H), 3.79–3.64 (m, 8H), 3.52–3.48 (dd, 1H), 3.23–3.20 (dd, 1H), 1.41 (d, 3H). ^13^C NMR (125 MHz, CDCl_3_) δ 173.24, 172.35, 139.19, 136.22, 128.92, 128.66, 128.49, 128.33, 127.36, 127.22, 123.20, 121.89, 119.22, 118.88, 113.37, 111.13, 62.48, 54.53, 52.36, 48.06, 29.71, 21.84, 18.87. HRMS S (ESI‐TOF): calculated for C_29_H_31_N_3_O_3_ [M + H^+^] 470.2365, found: 470.2443. [α]^25^_D_: −17.1° (c = 0.1, CHCl_3_).

Methyl *N*,*N*‐dibenzyl‐L‐tryptophyl‐L‐phenylglycinate (**4c**): Yield: 80%, orange oil. ^1^H NMR (500 MHz, CDCl_3_) δ 8.00 (s, 1H), 7.60 (d, 1H), 7.46 (d, 1H), 7.29 (d, 1H), 7.19–7.11 (m, 12H), 7.02 (t, 1H), 6.99 (s, 1H), 5.47 (d, 1H), 3.69–3.61 (m, 8H), 3.46 (m, 1H), 3.17 (m, 1H). ^13^C NMR (125 MHz, CDCl_3_) δ 172.33, 171.09, 139.07, 137.02, 136.21, 128.99, 128.64, 128.45, 127.32, 127.18, 123.20, 121.92, 119.25, 118.87, 113.43, 111.12, 62.40, 56.49, 54.45, 52.68, 21.50. HRMS S (ESI‐TOF): calculated for C_34_H_33_N_3_O_3_ [M + H^+^] 532.2522, found: 532.2602. [α]^25^_D_: −11.1° (c = 0.1, CHCl_3_).

Methyl *N*,*N*‐dibenzyl‐L‐tryptophyl‐L‐leucinate (**4d**): Yield: 70%, colorless oil. ^1^H NMR (500 MHz, CDCl_3_) δ 8.10 (s, 1H), 7.51 (d, 1H), 7.26 (s, 8H), 7.23–7.16 (m, 3H), 7.08–7.04 (t, 2H), 6.94 (d, 1H), 4.61 (q, 1H), 3.82–3.75 (q, 4H), 3.66 (s, 4H), 3.54–3.49 (dd, 1H), 3.25–3.21 (dd, 1H), 0.94 (d, 3H), 0.87 (d, 3H). ^13^C NMR (125 MHz, CDCl_3_) δ 173.16, 172.61, 139.18, 136.24, 128.50, 128.47, 127.35, 127.19, 123.20, 121.90, 119.23, 118.88, 113.33, 111.12, 62.61, 54.56, 52.14, 50.65, 42.05, 24.88, 22.68, 22.11, 21.99. HRMS S (ESI‐TOF): calculated for C_32_H_37_N_3_O_3_ [M + H^+^] 512.2835, found: 512.2909. [α]^25^_D_: −48.6° (c = 0.1, CHCl_3_).

Methyl *N*,*N*‐dibenzyl‐L‐tryptophyl‐L‐phenylalaninate (**4e**): Yield: 92%, colorless oil. ^1^H NMR (500 MHz, CDCl_3_) δ 8.08 (s, 1H), 7.50 (d, 1H), 7.37 (d, 1H), 7.28–7.03 (m, 20H), 4.87–4.84 (q, 1H), 3.71–3.63 (m, 8H), 3.53–3.49 (dd, 1H), 3.24–3.21 (dd, 2H), 3.08–3.04 (dd, 1H). ^13^C NMR (125 MHz, CDCl_3_) δ 172.66, 171.99, 139.10, 136.22, 136.02, 129.15, 128.58, 128.37, 127.35, 127.05, 123.09, 121.91, 119.24, 118.90, 113.35, 111.09, 62.54, 54.44, 53.39, 52.22, 38.09, 22.41. HRMS S (ESI‐TOF): calculated for C_35_H_35_N_3_O_3_ [M + H^+^] 546.2678, found: 546.2759. [α]^25^_D_: −17.3° (c = 0.1, CHCl_3_).

Methyl *N*,*N*‐dibenzyl‐L‐tryptophyl‐L‐tyrosinate (**4f**): Yield: 81%, yellow oil. ^1^H NMR (500 MHz, CDCl_3_) δ 8.00 (s, 1H), 7.47 (d, 1H), 7.35 (d, 1H), 7.25–7.16 (m, 8H), 7.14–7.13 (d, 5H), 7.08–7.05 (t, 1H), 6.99 (s, 1H), 6.89 (d, 2H), 6.61 (d, 2H), 5.00 (s, 1H), 4.80–4.76 (q, 1H), 3.70–3.66 (ds, 7H), 3.64–3.61 (t, 1H), 3.49–3.45 (dd, 1H), 3.22–3.18 (dd, 1H), 3.14–3.10 (dd, 1H), 2.96–2.92 (dd, 1H). ^13^C NMR (125 MHz, CDCl_3_) δ 172.67, 172.05, 154.64, 139.04, 136.17, 130.30, 128.54, 128.37, 128.00, 127.34, 127.07, 123.08, 121.91, 119.25, 118.89, 115.43, 113.40, 111.04, 62.49, 54.46, 53.50, 52.23, 37.33, 22.15. HRMS S (ESI‐TOF): calculated for C_35_H_35_N_3_O_4_ [M + H^+^] 562.2628, found: 562.2700. [α]^25^_D_: −21.2° (c = 0.1, CHCl_3_).

Methyl *N*,*N*‐dibenzyl‐L‐tryptophyl‐L‐threoninate (**4g**): Yield: 58%, orange oil. ^1^H NMR (500 MHz, CDCl_3_) δ 8.03 (s, 1H), 7.47–7.45 (d, 1H), 7.42–7.40 (d, 1H), 7.33–7.30 (m, 3H), 7.26–7.13 (m, 7H), 7.05–7.03 (s, 2H), 4.52–4.50 (d, 1H), 4.27 (s, 1H), 3.81–3.72 (q, 4H), 3.64 (s, 3H), 3.52–3.47 (dd, 1H), 3.25–3.21 (dd, 1H), 2.07 (s, 1H), 1.14 (d, 3H). ^13^C NMR (125 MHz, CDCl_3_) δ 173.52, 171.20, 139.01, 136.21, 128.64, 128.49, 127.34, 127.23, 123.14, 121.96, 119.28, 118.90, 113.42, 111.12, 68.30, 62.71, 57.41, 54.57, 52.46, 29.71, 22.10, 20.19. HRMS S (ESI‐TOF): calculated for C_30_H_33_N_3_O_4_ [M + H^+^] 500.2471, found: 500.2555. [α]^25^_D_: −23.8° (c = 0.1, CHCl_3_).

Methyl *N*,*N*‐dibenzyl‐L‐tryptophyl‐L‐serinate (**4h**): Yield: 38%, colorless oil. ^1^H NMR (500 MHz, CDCl_3_) δ 8.07 (s, 1H), 7.58–7.50 (dd, 2H), 7.36 (d, 1H), 7.30 (s, 7H), 7.23–7.18 (q, 3H), 7.10–7.07 (t, 2H), 4.56 (s, 1H), 3.92 (s, 2H), 3.83–3.71 (m, 9H), 3.55–3.50 (dd, 1H), 3.28–3.24 (dd, 1H), 2.62 (s, 1H). ^13^C NMR (125 MHz, CDCl_3_) δ 173.53, 170.56, 139.03, 136.19, 128.72, 128.51, 127.32, 127.26, 123.14, 121.98, 119.30, 118.86, 113.32, 111.13, 63.98, 62.61, 55.05, 54.56, 52.65, 21.82. HRMS S (ESI‐TOF): calculated for C_29_H_31_N_3_O_4_ [M + H^+^] 486.2315, found: 486.2391. [α]^25^_D_: −18.6° (c = 0.1, CHCl_3_).

Methyl *N*,*N*‐dibenzyl‐L‐tryptophyl‐L‐prolinate (**4i**): Yield: 43%, colorless oil. ^1^H NMR (500 MHz, CDCl_3_) δ 8.10 (s, 1H), 7.56 (d, 1H), 7.35–7.27 (m, 9H), 7.22–7.16 (m, 3H), 7.11–7.08 (t, 2H), 4.45 (t, 1H), 4.03 (d, 2H), 3.92 (dd, 1H), 3.85 (d, 1H), 3.69 (s, 3H), 3.46 (t, 1H), 3.30 (dd, 1H), 2.73 (q, 1H), 2.24 (q, 1H), 2.10 (q, 1H), 1.79 (q, 1H), 1.65 (q, 2H). ^13^C NMR (125 MHz, CDCl_3_) δ 172.94, 172.13, 140.15, 136.10, 128.74, 128.30, 128.19, 127.60, 126.92, 123.75, 121.63, 119.12, 118.44, 111.76, 111.11, 59.90, 58.66, 54.81, 52.03, 46.09, 29.04, 24.86, 23.41. HRMS S (ESI‐TOF): calculated for C_31_H_33_N_3_O_3_ [M + H^+^] 496.2522, found: 496.2599. [α]^25^_D_: +11.8° (c = 0.1, CHCl_3_).

Methyl *N*,*N*‐dibenzyl‐L‐tryptophyl‐L‐valinate (**4j**): Yield: 41%, orange oil. ^1^H NMR (500 MHz, CDCl_3_) δ 8.02 (s, 1H), 7.48 (d, 1H), 7.33 (d, 1H), 7.25–7.14 (9H, m), 7.08–7.03 (3H, m), 4.49–4.47 (q, 1H), 3.82–3.75 (q, 4H), 3.68 (s, 1H), 3.52–3.47 (dd, 1H), 3.24–3.20 (dd, 1H), 2.13–2.10 (q, 1H), 0.91 (d, 3H), 0.83 (d, 3H). ^13^C NMR (125 MHz, CDCl_3_) δ 172.27, 172.11, 139.14, 136.21, 128.45, 127.35, 127.16, 123.09, 121.92, 119.27, 118.92, 113.51, 111.05, 62.76, 57.27, 54.55, 51.98, 31.46, 22.31, 19.02, 18.04. HRMS S (ESI‐TOF): calculated for C_31_H_35_N_3_O_3_ [M + H^+^] 498.2678, found: 498.2771. [α]^25^_D_: −14.01° (c = 0.1, CHCl_3_).

Methyl *N*,*N*‐dibenzyl‐L‐tryptophyl‐L‐isoleucinate (**4k**): Yield: 46%, orange oil. ^1^H NMR (500 MHz, CDCl_3_) δ 8.03 (s, 1H), 7.49 (d, 1H), 7.33 (d, 1H), 7.26–7.03 (m, 15H), 4.53–4.50 (q, 1H), 3.82–3.74 (q, 4H), 3.67–3.65 (s, 4H), 3.52–3.48 (dd, 1H), 3.24–3.20 (dd, 1H), 1.83 (s, 1H) 1.41–1.36 (m, 1H), 1.12–1.03 (m, 1H), 0.86–0.83 (t, 6H). ^13^C NMR (125 MHz, CDCl_3_) δ 172.64, 172.09, 139.14, 136.22, 128.46, 127.35, 127.16, 123.11, 121.92, 119.26, 118.91, 113.49, 111.07, 62.70, 56.54, 54.55, 51.92, 38.19, 25.43, 22.19, 15.51, 11.57. HRMS S (ESI‐TOF): calculated for C_32_H_37_N_3_O_3_ [M + H^+^] 512.2835, found: 512.2910. [α]^25^_D_: −37.4° (c = 0.1, CHCl_3_).

Methyl *N*,*N*‐dibenzyl‐L‐tryptophyl‐L‐methioninate (**4l**): Yield: 70%, colorless oil. ^1^H NMR (500 MHz, CDCl_3_) δ 8.07 (s, 1H), 7.51 (d, 1H), 7.36 (d, 1H), 7.29 (s, 8H), 7.25 (m, 3H) 7.20–7.17 (m, 1H), 7.09 (t, 2H), 4.65 (q, 1H), 3.84–3.76 (q, 4H), 3.72 (s, 3H), 3.70 (d, 1H), 3.55 (dd, 1H), 3.27 (dd, 1H), 2.42 (q, 2H), 2.20 (q, 1H), 2.01 (m, 4H). ^13^C NMR (125 MHz, CDCl_3_) δ 172.75, 172.11, 139.04, 136.22, 128.53, 128.50, 127.31, 127.25, 123.14, 121.96, 119.29, 118.89, 113.40, 111.11, 62.62, 54.59, 52.40, 51.55, 32.03, 29.86, 22.01, 15.37. HRMS S (ESI‐TOF): calculated for C_31_H_35_N_3_O_3_S [M + H^+^] 530.2400, found: 530.2480. [α]^25^_D_: −17.9° (c = 0.1, CHCl_3_).

#### 2.1.4 General Procedure for the Synthesis of *N*,*N*‐Dibenzyl‐Amino Acids (**5**)

To a stirred solution of the corresponding commercially available L‐amino acid methyl ester hydrochloride (1 mmol) in a 4:1 mixture of tetrahydrofuran‐dimethyl sulfoxide (THF/DMSO, 25 mL), benzyl bromide (3 mmol), TBAI (10% mol), and sodium bicarbonate (3.5 mmol) were added at room temperature. The reaction mixture was refluxed overnight. The crude mixture was diluted with diethyl ether (50 mL), and the organic layer was washed sequentially with water (50 mL), a saturated aqueous solution of sodium bicarbonate (50 mL) and brine (50 mL). The organic phase was dried over anhydrous Na_2_SO_4_, filtered, and concentrated under reduced pressure; the resulting crude intermediate was used in the next step without further purification. Subsequently, this crude material was dissolved in a 2:1:1 THF/MeOH/H_2_O mixture (40 mL), and NaOH (5.0 equiv) was added at 0°C. The mixture was allowed to warm to room temperature and stirred overnight. The aqueous phase was washed with diethyl ether (3 times), and then acidified to pH 3 using a 37% HCl solution. The product was extracted with ethyl acetate (3 times), dried over anhydrous Na_2_SO_4_, and the solvent was evaporated under vacuum to afford the corresponding target product (**5**).

*N*,*N*‐Dibenzyl‐glycine (**7a**): Yield: 53%, white solid, mp 129°C. ^1^H NMR (500 MHz, DMSO–*d*_6_) δ 7.28–7.37 (m, 10H, Ar.), 3.77 (s, 4H, 2× CH_2_‐Ph), 3.18 (s, 2H). ^13^C NMR (125 MHz, DMSO—d_6_) δ 53.85, 57.63, 127.81, 129.07, 129.37, 139.84, 173.04. HRMS S (ESI‐TOF): calculated for C_16_H_17_NO_2_ [M + H^+^] 255.1259, found: 255.1341.

*N*,*N*‐Dibenzyl‐L‐alanine (**7b**): Yield: 40%, colorless oil. ^1^H NMR (500 MHz, CDCl_3_) δ 10.43 (s, 1H, COOH), 7.39–7.29 (m, 10H, Ar.), 3.93–3.91 (d, 2H), 3.72–3.70 (d, 2H), 3.64–3.59 (q, 1H), 1.44–1.42 (d, 3H). ^13^C NMR (125 MHz, CDCl_3_) δ 175.52, 137.11, 129.09, 128.75, 127.93, 57.58, 54.59, 11.29. HRMS S (ESI‐TOF): calculated for C_17_H_19_NO_2_ [M + H^+^] 269.1416, found: 269.1500. [α]^25^_D_: −50.9° (c = 0.1, CHCl_3_).

*N*,*N*‐Dibenzyl‐L‐phenylglycine (**7c**): Yield: 18%, orange oil. ^1^H NMR (500 MHz, CDCl_3_) δ 7.39–7.28 (m, 15H, Ar.), 4.66 (s, 1H), 3.91–3.88 (d, 2H), 3.48–3.45 (d, 2H). ^13^C NMR (125 MHz, CDCl_3_) δ 173.66, 137.48, 130.02, 129.01, 128.73, 128.52, 128.46, 127.76, 66.64, 54.64. HRMS S (ESI‐TOF): calculated for C_22_H_21_NO_2_ [M + H^+^] 331.1572, found: 331.1641. [α]^25^_D_: +6.0° (c = 0.1, CHCl_3_).

*N*,*N*‐Dibenzyl‐L‐leucine (**7d**): Yield: 26%, orange oil. ^1^H NMR (500 MHz, CDCl_3_) δ 11.74 (s, 1H, COOH), 7.56–7.40 (m, 10H, Ar.), 4.12–4.09 (d, 2H), 3.95–3.92 (d, 2H), 3.69–3.67 (t, 1H), 2.08–2.06 (m, 1H), 1.87–1.82 (m, 2H), 1.03–0.85 (dd, 6H). ^13^C NMR (125 MHz, CDCl_3_) δ 177.67, 138.12, 128.83, 128.04, 127.04, 59.11, 54.24, 37.61, 24.27, 22.60, 21.57. HRMS S (ESI‐TOF): calculated for C_20_H_25_NO_2_ [M + H^+^] 311.1885, found: 311.1968. [α]^25^_D_: −48.9° (c = 0.1, CHCl_3_).

*N*,*N*‐Dibenzyl‐L‐phenylalanine (**7e**): Yield: 80%, white crystalline powder, m.p. 113°C. ^1^H NMR (500 MHz, CDCl_3_) δ 10.07 (s, 1H, COOH), 7.43–7.17 (m, 15H, Ar.), 3.95–3.92 (d, 2H), 3.86–3.81 (t, 3H), 3.37–3.33 (m, 1H), 3.14–3.09 (m, 1H). ^13^C NMR (125 MHz, CDCl_3_) δ 176.58, 138.19, 138.26, 129.51, 128.96, 128.48, 128.42, 127.45, 126.56, 62.69, 54.52, 34.67. HRMS S (ESI‐TOF): calculated for C_23_H_23_NO_2_ [M + H^+^] 345.1729, found: 345.1810. [α]^25^_D_: −11.0° (c = 0.1, CHCl_3_).

*N*,*N*‐Dibenzyl‐L‐tyrosine (**7f**): Yield: 29%, orange oil. ^1^H NMR (500 MHz, CDCl_3_) δ 7.28–7.20 (m, 10H, Ar.), 6.97–6.80 (dd, 4H, Ar.), 3.87–3.84 (d, 2H), 3.74–3.72 (d, 3H), 3.35–3.31 (m, 1H), 2.97–2.92 (m, 1H). ^13^C NMR (125 MHz, CDCl_3_) δ 174.07, 155.12, 136.29, 130.32, 129.13, 129.00, 128.77, 128.04, 115.73, 64.01, 54.67, 32.92. HRMS S (ESI‐TOF): calculated for C_23_H_23_NO_3_ [M + H^+^] 361.1678, found: 361.1755. [α]^25^_D_: −12.2° (c = 0.1, CHCl_3_).

*N*,*N*‐Dibenzyl‐L‐threonine (**7g**): Yield: 32%, yellow oil. ^1^H NMR (500 MHz, CDCl_3_) δ 7.78 (s, 1H, OH), 7.31–7.25 (m, 10H, Ar.), 4.20–4.15 (t, 3H), 3.87–3.85 (d, 2H), 3.26–3.24 (d, 1H), 1.25–1.24 (d, 3H). ^13^C NMR (125 MHz, CDCl_3_) δ 172.51, 136.48, 129.79, 129.05, 128.37, 67.85, 63.96, 55.34, 20.45. HRMS S (ESI‐TOF): calculated for C_18_H_21_NO_3_ [M + H^+^] 299.1521, found: 299.1606. [α]^25^_D_: −77.3° (c = 0.1, CHCl_3_).

*N*,*N*‐Dibenzyl‐L‐serine (**7h**): Yield: 83%, white crystalline powder, m.p. 145°C. ^1^H NMR (500 MHz, DMSO–*d*_*6*_) δ 7.33–7.21 (m, 10H, Ar.), 3.84–3.78 (m, 3H), 3.67–3.26 (m, 3H), 3.36 (s, 1H, OH), 3.29–3.26 (t, 1H). ^13^C NMR (125 MHz, DMSO–*d*_*6*_) δ 173.10, 140.27, 128.86, 128.64, 127.30, 63.35, 60.90, 55.12. HRMS S (ESI‐TOF): calculated for C_17_H_19_NO_3_ [M + H^+^] 285.1365, found: 285.1447. [α]^25^_D_: −28.6° (c = 0.1, DMSO).

#### 2.1.5 Synthesis of *N*‐Benzyl‐L‐Proline (**7i**)

To a solution of commercially available L‐proline (1.00 g, 8.69 mmol) in *i*‐PrOH (25 mL), potassium hydroxide (2.5 eq.) and benzyl bromide (2.0 eq.) were added at room temperature, and the reaction mixture was stirred at reflux overnight. The reaction was diluted with water (40 mL), and the aqueous layer was washed with diethyl ether (3 × 20 mL). Then, the aqueous layer was acidified to pH 3 with a 37% HCl solution, and the product was extracted with a 3:1 v/v mixture of chloroform *i*‐PrOH (3 × 20 mL). The organic layer was dried over anhydrous Na_2_SO_4_, filtered, and the solvent was removed under vacuum to give the corresponding product **7i**. Yield: 86%, white solid, m.p. 168°C. ^1^H NMR (500 MHz, DMSO–*d*_*6*_) δ 7.51–7.43 (d, 5H, Ar.), 4.37–4.35 (m, 1H), 4.16–4.13 (m, 1H), 3.92 (s, 1H), 3.32 (s, 1H), 3.03 (s, 1H), 2.32 (s, 1H), 1.97 (s, 2H), 1.82 (s, 1H). ^13^C NMR (125 MHz, DMSO–*d*_*6*_) δ 171.66, 133.80, 130.95, 129.53, 129.37, 66.51, 58.05, 54.41, 29.03, 23.14. HRMS S (ESI‐TOF): calculated for C_12_H_15_NO_2_ [M + H^+^] 205.1103, found: 205.1180. [α]^25^_D_: −5.7° (c = 0.1, DMSO).

#### 2.1.6 General Procedure for the Synthesis of Methyl X‐Tryptophanate Dipeptides

To a solution of the corresponding *N*,*N*‐dibenzyl amino acid (**7a**–**i**) (100 mg) in dichloromethane (15 mL) at 0°C, Et_3_N (2 eq.) HOBt (1.1 eq.) and 1‐ethyl‐3‐(3‐dimethylaminopropyl) carbodiimide hydrochloride (1.1 eq.) were added and the reaction was stirred 30 min. After this time, commercially available L–tryptophan methyl ester hydrochloride (**1**, 2 eq.) was added and the reaction was stirred at room temperature overnight. The solvent was removed under vacuum and the crude was reconstituted with AcOEt (30 mL). The organic layer was washed with 0.5 M citric acid solution (20 mL), sodium bicarbonate saturated solution (20 mL), brine (20 mL), dried over Na_2_SO_4_, and filtered. The solvent was removed under vacuum and the residue was purified using flash column chromatography.

Methyl *N*,*N*‐dibenzylglycyl L‐tryptophanate (**8a**): Yield: 38%, colorless oil. ^1^H NMR (500 MHz, CDCl_3_) δ 8.10 (s, 1H), 7.82–8.80 (d, 1H), 7.56–7.55 (d, 1H), 7.35–7.33 (d, 1H), 7.23–7.18 (m, 7H), 7.12 (s, 5H), 6.84 (s, 1H), 4.92–4.91 (d, 1H), 3.65 (s, 3H), 3.61–3.58 (d, 2H), 3.37–3.31 (m, 4H), 3.17–3.14 (d, 1H), 3.04–3.01 (d, 1H). ^13^C NMR (125 MHz, CDCl_3_) δ 172.34, 170.96, 137.86, 136.16, 128.90, 128.41, 127.57, 127.33, 122.79, 122.31, 119.70, 118.63, 111.24, 109.91, 58.69, 57.33, 52.27, 29.71, 27.54. HRMS S (ESI‐TOF): calculated for C_28_H_29_N_3_O_3_ [M + Na^+^] 455.2209, found: 455.2111. [α]^25^_D_: +12.3° (c = 0.1, CDCl_3_).

Methyl *N*,*N*‐dibenzyl‐L‐alanyl‐L‐tryptophanate (**8b**): Yield: 71%, yellow oil. ^1^H NMR (500 MHz, CDCl_3_) δ 8.09–8.08 (d, 1H), 7.86 (s, 1H), 7.60–7.58 (d, 1H), 7.31 (d, 5H), 7.23–7.12 (m, 7H), 6.83 (s, 1H), 4.94–4.93 (d, 1H), 3.78 (s, 3H), 3.59–3.56 (d, 2H), 3.51–3.47 (m, 1H), 3.43–3.40 (m, 1H), 3.36–3.33 (d, 2H), 1.33–1.32 (d, 3H). ^13^C NMR (125 MHz, CDCl_3_) δ 173.84, 172.86, 138.94, 136.33, 128.93, 128.73, 128.70, 128.47, 127.96, 127.42, 122.84, 122.38, 119.75, 118.96, 111.46, 110.32, 57.97, 54.60, 53.19, 52.64, 30.00, 27.94, 8.15. HRMS S (ESI‐TOF): calculated for C_29_H_31_N_3_O_3_ [M + Na^+^] 469.2365, found: 469.2260. [α]^25^_D_: −22.5° (c = 0.1, CDCl_3_).

Methyl ((*S*)‐2‐(*N*,*N*‐dibenzylamino)‐2‐phenylacetyl)‐L‐tryptophanate (**8c**): Yield: 43%, orange oil. ^1^H NMR (500 MHz, CDCl_3_) δ 8.10–7.96 (m, 1H), 7.83 (s, 1H), 7.61–7.59 (d, 1H), 7.44–7.39 (m, 1H), 7.34 (s, 2H), 7.29–7.28 (m, 8H), 7.24 (m, 3H), 7.16–7.14 (m, 2H), 6.92–6.85 (d, 1H), 5.06–5.05 (d, 1H), 4.43–4.40 (d, 1H), 3.86–3.79 (s, 3H), 3.72 (s, 1H), 3.66–3.64 (d, 1H), 3.52–3.48 (m, 1H), 3.45–3.36 (m, 1H), 3.20–3.17 (d, 2H). ^13^C NMR (125 MHz, CDCl_3_) δ 172.56, 171.38, 1136.22, 136.07, 134.07, 130.76, 130.20, 128.79, 128.62, 128.44, 128.13, 128.04, 127.79, 127.60, 127.20, 127.14, 122.83, 122.72, 122.44, 122.44, 122.25, 119.82, 119.62, 118.72, 118.67, 111.26, 111.20, 110.07, 67.86, 54.41, 54.34, 52.64, 52.43, 52.36, 27.80, 27.54. HRMS S (ESI‐TOF): calculated for C_34_H_33_N_3_O_3_ [M + Na^+^] 531.2522, found: 531.2417. [α]^25^_D_: +25.6° (c = 0.1, CDCl_3_).

Methyl *N*,*N*‐dibenzyl‐L‐leucyl‐L‐tryptophanate (**8d**): Yield: 72%, yellow oil. ^1^H NMR (500 MHz, CDCl_3_) δ 7.99 (s, 1H), 7.64–7.62 (d, 1H), 7.48–7.47 (d, 1H), 7.41–7.40 (d, 1H), 7.32–7.30 (d, 4H), 7.23–7.22 (m, 5H), 7.18–7.16 (t, 1H), 6.95 (s, 1H), 4.99–4.96 (q, 1H), 3.78 (s, 3H), 3.58–3.38 (m, 6H), 3.23–3.21 (q, 1H), 0.96–0.95 (d, 3H), 0.88–0.87 (d, 3H). ^13^C NMR (125 MHz, CDCl_3_) δ 173.22, 172.41, 138.88, 135.90, 128.78, 128.36, 128.31, 128.28, 128.27, 128.12, 127.38, 127.28, 126.83, 122.36, 121.98, 119.36, 118.44, 110.99, 109.90, 59.63, 53.99, 52.61, 52.11, 34.64, 27.58, 25.76, 22.95, 22.06. HRMS S (ESI‐TOF): calculated for C_32_H_37_N_3_O_3_ [M + Na^+^] 511.2835, found: 511.2737. [α]^25^_D_: −14.3° (c = 0.1, CDCl_3_).

Methyl *N*,*N*‐dibenzyl‐L‐phenylalanyl‐L‐tryptophanate (**8e**): Yield: 79%, orange amorphous solid. ^1^H NMR (500 MHz, CDCl_3_) δ 7.85 (s, 1H), 7.65–7.58 (dd, 2H), 7.38–7.28 (m, 10H), 7.22–7.19 (t, 2H), 7.13–7.12 (d, 4H), 6.92 (s, 1H), 4.98–4.96 (d, 1H), 3.79 (s, 3H), 3.61–3.39 (m, 8H), 3.10–3.06 (m, 1H). ^13^C NMR (125 MHz, CDCl_3_) δ 172.48, 172.26, 140.24, 138.68, 136.08, 129.47, 128.51, 128.35, 128.33, 127.68, 127.09, 126.10, 122.53, 122.22, 119.59, 118.73.111.20, 110.18, 63.71, 54.32, 53.02, 52.36, 32.04, 27.70. HRMS S (ESI‐TOF): calculated for C_35_H_35_N_3_O_3_ [M + Na^+^] 545.2678, found: 545.2572. [α]^25^_D_: −10.1° (c = 0.1, CDCl_3_).

Methyl *N*,*N*‐dibenzyl‐L‐tyrosyl‐L‐tryptophanate (**8f**): Yield: 37%, colorless oil. ^1^H NMR (500 MHz, CDCl_3_) δ 7.77–7.75 (d, 1H), 7.52–7.50 (d, 1H), 7.33–7.26 (m, 9H), 7.14 (s, 6H), 6.90–6.88 (d, 2H), 5.07 (s, 6H), 3.92 (s, 3H), 3.73–3.70 (d, 2H), 3.63–3.59 (m, 2H), 3.52 (s, 6H), 3.47–3.38 (m, 2H), 3.28–3.24 (m, 1H), 3.12–3.07 (m, 1H). ^13^C NMR (125 MHz, CDCl_3_) δ 201.89, 184.81, 168.62, 159.42, 157.72, 157.10, 155.84, 152.51, 152.34, 150.56, 147.90, 147.31, 144.00, 140.54, 140.49, 138.51.93.18, 83.16, 82.27, 80.80, 62.10, 56.15. HRMS S (ESI‐TOF): calculated for C_35_H_35_N_3_O_4_ [M + H^+^] 561.2628, found: 561.2708. [α]^25^_D_: −4.4° (c = 0.1, CDCl_3_).

Methyl *N*,*N*‐dibenzyl‐L‐threonyl‐L‐tryptophanate (**8g**): Yield: 79%, orange oil. ^1^H NMR (500 MHz, CDCl_3_) δ 8.21 (s, 1H), 7.66–7.64 (d, 1H), 7.38–7.37 (d, 1H), 7.26–7.17 (m, 8H), 7.08 (s, 1H), 7.02 (s, 4H), 6.15–6.14 (d, 1H), 5.07–5.06 (d, 1H), 4.13–4.10 (t, 1H), 3.93–3.90 (d, 2H), 3.77 (s, 3H), 3.50–3.44 (m, 2H), 3.33–3.28 (1H), 3.12–3.09 (d, 2H), 2.73–2.71 (d, 1H), 1.10–1.09 (d, 3H). ^13^C NMR (125 MHz, CDCl_3_) δ 172.76, 169.89, 138.82, 136.60, 128.94, 128.91, 127.78, 127.63, 122.99, 122.84, 120.35, 118.99, 111.75, 110.38, 68.27, 64.43, 54.96, 52.86, 52.61, 27.92, 19.53. HRMS S (ESI‐TOF): calculated for C_30_H_33_N_3_O_4_ [M + H^+^] 499.2471, found: 499.2543. [α]^25^_D_: −39.5° (c = 0.1, CDCl_3_).

Methyl *N*,*N*‐dibenzyl‐L‐seryl‐L‐tryptophanate (**8h**): Yield: 77%, orange oil. ^1^H NMR (500 MHz, CDCl_3_) δ 8.08–8.07 (d, 1H), 7.71 (s, 1H), 7.58–7.56 (d, 1H), 7.31 (s, 2H), 7.26 (s, 3H), 7.22–7.12 (m, 7H), 6.82 (s, 1H), 4.93–4.92 (d, 1H), 4.09–4.05 (t, 1H), 3.98 (s, 1H), 3.82 (s, 3H), 3.64–3.61 (d, 2H), 3.56–3.52 (m, 1H), 3.45–3.36 (m, 5H), 1.66 (s, 1H), 1.31–1.26 (d, 1H). ^13^C NMR (125 MHz, CDCl_3_) δ 173.63, 172.20, 138.30, 136.02, 128.49, 128.43, 127.60, 127.31, 122.53, 122.23, 119.59, 118.57, 111.21, 109.79, 62.10, 57.96, 54.61, 52.80, 52.54, 27.48. HRMS S (ESI‐TOF): calculated for C_29_H_31_N_3_O_4_ [M + H^+^] 485.2315, found: 485.2397. [α]^25^_D_: −7.5° (c = 0.1, CDCl_3_).

Methyl *N*‐benzyl‐L‐prolyl‐L‐tryptophanate (**8i**): Yield: 41%, colorless oil. ^1^H NMR (500 MHz, CDCl_3_) δ 8.39 (s, 1H), 8.07–8.05 (d, 1H), 7.59–7.57 (d, 1H), 7.39–7.37 (d, 1H), 7.19 (s, 4H), 7.11–7.08 (t, 1H), 6.98 (s, 3H), 4.99–4.98 (d, 1H), 3.93–3.90 (d, 1H), 3.63 (s, 3H), 3.43–3.39 (m, 1H), 3.34–3.30 (m, 1H), 3.27–3.24 (d, 1H), 3.16–3.15 (d, 1H), 2.76 (s, 1H), 2.17–2.15 (d, 2H), 1.77 (s, 1H), 1.66 (s, 1H), 1.52–1.50 (d, 1H). ^13^C NMR (125 MHz, CDCl_3_) δ 174.96, 172.62, 138.85, 136.45, 128.91, 128.51, 127.95, 127.24, 123.02, 122.49, 119.85, 118.90, 111.53, 110.40, 67.61, 59.79, 53.46, 52.60, 52.50, 30.74, 28.00, 24.24. HRMS S (ESI‐TOF): calculated for C_24_H_27_N_3_O_3_ [M + H^+^] 405.2052, found: 405.2134. [α]^25^_D_: −34.5° (c = 0.1, CDCl_3_).

### 2.2 Biology

Cell culture medium, supplements, reagents, and sterile plasticware for biological assays were purchased from commercial suppliers. The human solid tumor cell lines A549, HeLa, MIA PaCa‐2, SW1573, T‐47D, and WiDr were used in this study. These cell lines were a kind gift from Prof. G. J. Peters (VU Medical Center, Amsterdam, The Netherlands). The maintenance of cell cultures was in 60 mm Petri dishes in a humidified air incubator (37°C, 5% CO_2_, 95% humidity). The cell culture medium used was RPMI 1640 (Sigma‐Aldrich, USA) supplemented with 5% heat‐inactivated fetal bovine serum FBS (Lonza, Switzerland), 2 mM L‐glutamine (Lonza, Switzerland), and antibiotics (100 units of penicillin G and 0.1 mg of streptomycin per mL; Lonza, Switzerland). Cell cultures were passaged biweekly using 0.05% trypsin (Trypsin–EDTA solution; Lonza, Switzerland) and maintained at low passage. Single cell suspensions were counted using EVE Plus (NanoEntek Europe, Germany) automated cell counter.

#### 2.2.1 Antiproliferative Assay

In vitro antiproliferative activity was evaluated following a modified version of the National Cancer Institute (NCI) protocol [12]. Cells were seeded in 96‐well plates at densities of 2500 cells/well for the A549, HeLa, MIA PaCa‐2, and SW1573 lines, and 5000 cells/well for T‐47D and WiDr. The dipeptide derivatives were dissolved in DMSO to prepare 40 mM stock solutions, with 100 μM serving as the highest evaluated concentration. Following a 48‐h incubation period, cellular mass was quantified using the sulforhodamine B (SRB) colorimetric assay [13]. The compound effects were determined as the concentration causing 50% growth inhibition (GI_50_) [14].

## 3 Results and Discussion

Building upon our group's previous findings regarding cell‐line‐selective growth inhibition driven by tryptophan‐containing dipeptides, we prepared both the Trp‐X (*C‐series*) and X‐Trp (*N‐series*) isomeric libraries to fully map their biological potential. Alternating the sequential order of the amino acids enabled us to assess how the positional presentation of the indole ring dictates both chemical accessibility and cellular interactions. From a synthetic viewpoint, this bidirectional arrangement shifts the steric constraints imposed by the *N*,*N*‐dibenzyl groups between the nucleophilic and electrophilic centers during peptide coupling. Mechanistically, this structural inversion provides crucial insights into whether the structural parameters required for intracellular accumulation or specific carrier affinity require a definitive N‐ or C‐terminal placement of the tryptophan motif.

### 3.1 Synthesis and Peptide Coupling of the *C*‐Series Dipeptides (Trp‐X)

**FIGURE 2:**
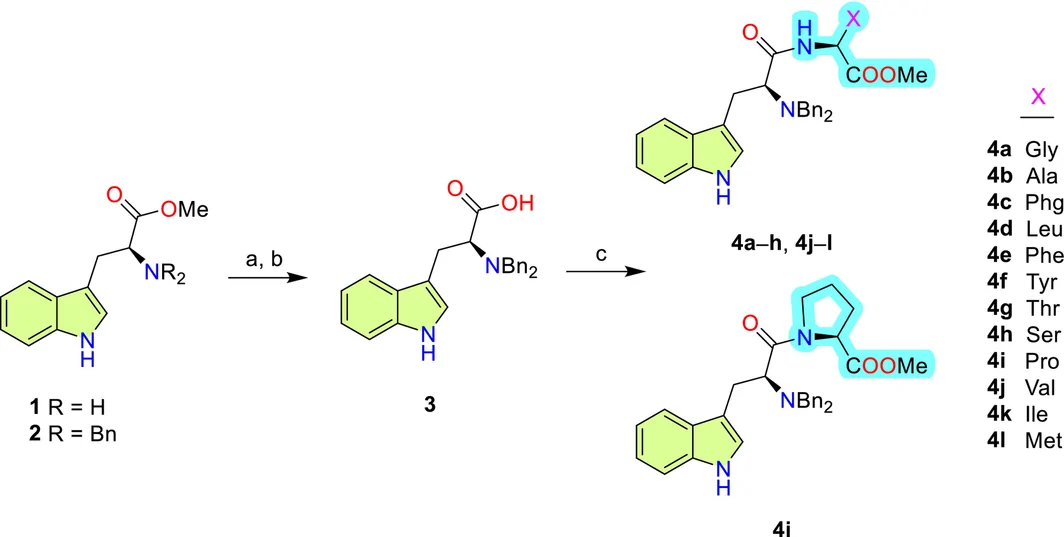
Synthesis of tryptophan‐based dipeptides **4a**–**l**. Reagents and conditions: **a** BnBr, NaHCO_3_, TBAI, THF/DMSO (4:1), Δ, overnight, 74%; **b** LiOH, THF/MeOH/H_2_O, Δ, overnight, 81%; **c** L‐amino acid methyl ester hydrochloride (X), HOBt, Et_3_N, EDC, DCM, 0°C, room temperature, overnight, 43%–70%.

The synthetic route (Figure 2) commenced with commercially available L‐tryptophan methyl ester hydrochloride (**1**), which underwent selective *N*‐α‐dibenzylation utilizing benzyl bromide. This nucleophilic substitution was conducted under reflux in a THF/DMSO (4:1) solvent mixture, employing sodium bicarbonate as the base and tetrabutylammonium iodide (TBAI) as a phase‐transfer catalyst. This initial protection of the α‐amino group is critical to preclude undesirable side reactions during subsequent peptide coupling steps. Furthermore, the introduction of the two hydrophobic benzyl rings significantly enhances the lipophilicity of the scaffold, a structural modification intended to facilitate passive membrane permeability, promote intracellular accumulation, and ultimately boost antiproliferative potency. Through this protection strategy, the key intermediate **2** was successfully isolated as an amorphous white solid in a 74% yield.

Saponification of intermediate **2** into the free carboxylic acid **3** was accomplished using LiOH in a THF/MeOH/H_2_O matrix. This transformation took advantage of mild reaction parameters to safeguard the configuration of the adjacent chiral carbon and avoid any migration of the amine‐protecting groups. As a result, *N,N*‐dibenzyl‐L‐tryptophan (**3**) was recovered in a solid 81% yield as the common intermediate for downstream functionalization.

Peptide condensation was then achieved via standard chemical activation using EDC hydrochloride and HOBt with triethylamine in dichloromethane. The mixture was stirred overnight, starting from an ice bath and gradually reaching room temperature. The inclusion of HOBt is essential to block potential racemization pathways of the activated amino acid, while EDC drives efficient amide bond formation via a highly reactive transition state under neutral and non‐destructive conditions.

Regarding the members of the *C*‐series, an analysis of their chemical yields in relation to their structural features reveals a reactivity pattern governed primarily by steric hindrance, polarity, and non‐covalent interactions of the incoming amino acid side chains.

The highest yields within this library were achieved by derivatives featuring either minimal steric bulk or aromatic side chains. The L‐alanine derivative (**4b**) afforded an 85% yield due to its small methyl side chain, which offers negligible steric resistance to the nucleophilic attack. Concurrently, L‐phenylalanine (**4e**, 92%), L‐tyrosine (**4f**, 81%), and L‐phenylglycine (**4c**, 80%) provided the highest conversions of the entire group. In these instances, favorable π–π stacking interactions between the peripheral aromatic rings and the indole core of the tryptophan residue likely stabilize the transition state during the condensation step, promoting clean conversions and efficient isolation.

Aliphatic derivatives with non‐branched, flexible structures also allowed clean transformations, as exemplified by the L‐leucine derivative (**4d**) and the sulfur‐containing L‐methionine derivative (**4l**), which both achieved a high and reproducible yield of 70%. In these cases, the side chains do not compromise the nucleophilic approach to the intermediate activated ester. In contrast, amino acids bearing substituents branched at the β‐carbon experienced a prominent decline in chemical yields due to localized steric repulsion. This trend is clearly illustrated by the L‐valine (**4j**) and L‐isoleucine (**4k**) derivatives, whose yields dropped to 41% and 46%, respectively. The high molecular volume adjacent to the nucleophilic amino group hinders its approach to the activated benzotriazole intermediate. Furthermore, polar hydroxylated amino acids gave moderate‐to‐low yields; the L‐threonine derivative (**4g**) was isolated in 58% yield, whereas L‐serine (**4h**) yielded only 38%. The free hydroxyl group in these side chains can induce minor, competing side reactions and increases the overall polarity of the crude material, thereby complicating flash chromatographic purification. This behavior is consistent with literature precedents highlighting the complex reactive landscapes and unique intramolecular transformations often facilitated by unprotected hydroxyl functions adjacent to N,N‐dibenzyl scaffolds [15, 16].

A similar drop in efficiency was observed for the secondary cyclic amino acid L‐proline (**4i**, 43%), where the conformationally restricted pyrrolidine ring introduces substantial steric constraints during transition‐state organization. Finally, the glycine derivative (**4a**), which lacks an α‐carbon substituent and presents no steric barriers, was obtained in a moderate 60% yield. This lower‐than‐expected recovery was attributed to partial material losses during aqueous extraction washes, caused by the higher hydrophilicity of the unfunctionalized core.

### 3.2 Synthesis of the *N*‐Series Precursors and Structural Limitations (X‐Trp)

**FIGURE 3:**
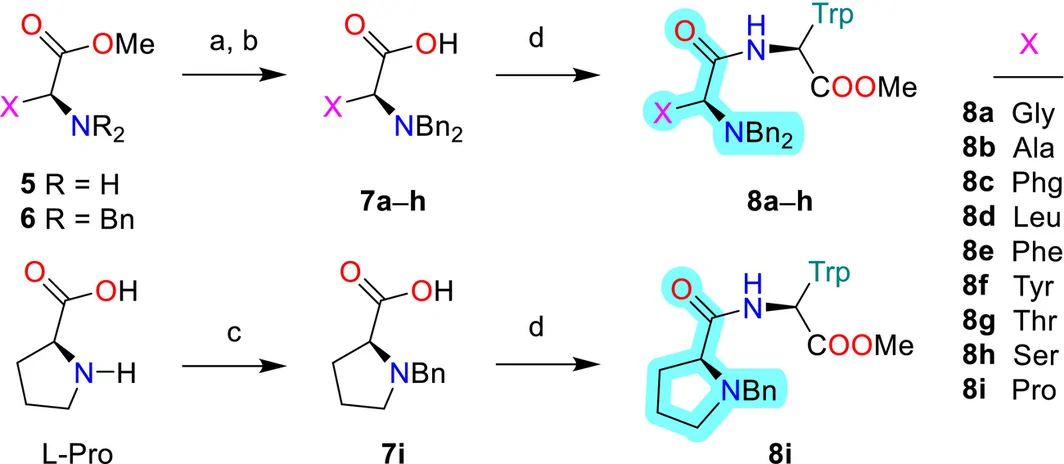
Synthesis of tryptophan‐based dipeptides **8a**–**i**. Reagents and conditions: **a** BnBr, NaHCO_3_, TBAI, THF/DMSO (4:1), Δ, overnight; **b** LiOH, THF/MeOH/H_2_O, Δ, overnight, 18%–86%; **c** BnBr, KOH, *i*‐PrOH, Δ, overnight; **d** HOBt, Et_3_N, EDC, L‐tryptophan methyl ester hydrochloride (**1**), DCM, 0°C to room temperature, overnight, 37%–79%.

The synthesis of the *N*‐series derivatives (Figure 3) commenced with the hydrochlorides of commercially available L‐amino acid methyl esters (**5**), which were subjected to an *N*,*N*‐dibenzylation via nucleophilic substitution following the protocol described for intermediate **2**. In the specific case of L‐proline, which features a cyclic secondary amine framework, the steric and electronic constraints of the pyrrolidine ring restricted the reaction, introducing only a single benzyl group to afford the corresponding *N*‐monobenzylated product [17].

Subsequently, the saponification of the intermediate esters (**6**) to afford the corresponding *N,N*‐dibenzylated carboxylic acids (**7a**–**i**) was carried out utilizing lithium hydroxide in a THF/MeOH/H_2_O ternary mixture, followed by acidification with concentrated hydrochloric acid to pH 3 to facilitate subsequent extraction.

During this initial stage of precursor preparation, prominent synthetic limitations emerged. On the one hand, the derivatives corresponding to L‐valine and L‐isoleucine could not be obtained because their dibenzylated methyl esters proved to be completely inert under basic hydrolysis conditions. The simultaneous presence of the bulky *N,N*‐dibenzyl group at the α‐nitrogen and the branching at the β‐carbon of these specific side chains generates severe steric hindrance around the adjacent ester carbonyl group. This localized congestion effectively prevents the nucleophilic attack of the hydroxide ion, thereby halting the synthesis of the free carboxylic acids [18]. On the other hand, when the dibenzylation protocol was attempted on the methyl ester of L‐methionine, a complex mixture of undesirable byproducts was generated. This outcome was attributed to the high nucleophilic reactivity of the thioether sulfur atom, which actively competed with the α‐amino group for the benzyl bromide electrophile [19]. This competing pathway led to secondary sulfonium salt formation and cross‐alkylation reactions, rendering the isolation of the pure intermediate impossible [20].

Finally, peptide coupling for the successfully isolated precursors (**7a**–**i**) was performed by activating the carboxylic acid cores with EDC hydrochloride and HOBt in the presence of triethylamine in dichloromethane. The reaction was initiated at 0°C and stirred overnight at room temperature using commercial L‐tryptophan methyl ester hydrochloride (**1**) as the nucleophilic component. Following flash chromatographic purification, the target *N*‐series dipeptides (**8a**–**i**) displayed notable variations in their chemical yields. The highest efficiencies were achieved for L‐phenylalanine (**8e**, 79%), L‐threonine (**8g**, 79%), L‐serine (**8h**, 77%), and L‐leucine (**8d**, 72%), owing to their favorable spatial arrangement during condensation. The successful employment of these N,N‐dibenzylated hydroxylated precursors aligns with modern synthetic frameworks where similarly functionalized serine and threonine derivatives are utilized as core building blocks for complex peptide condensations and specialized chemical targets [21, 22].

In contrast, the conjugates of glycine (**8a**, 38%), L‐tyrosine (**8f**, 37%), L‐proline (**8i**, 41%), and L‐phenylglycine (**8c**, 43%) gave substantially lower yields. This diminished efficiency was ascribed to the direct presence of the *N,N*‐dibenzyl shielding on the electrophilic carboxylic component. This spatial congestion creates a direct steric shield projected toward the reaction center, which severely hinders the critical formation of the intermediate *O*‐acylisourea with EDC and reduces the overall conversion rate during coupling.

### 3.3 Antiproliferative Effects Against Human Cancer Cell Lines

**FIGURE 4:**
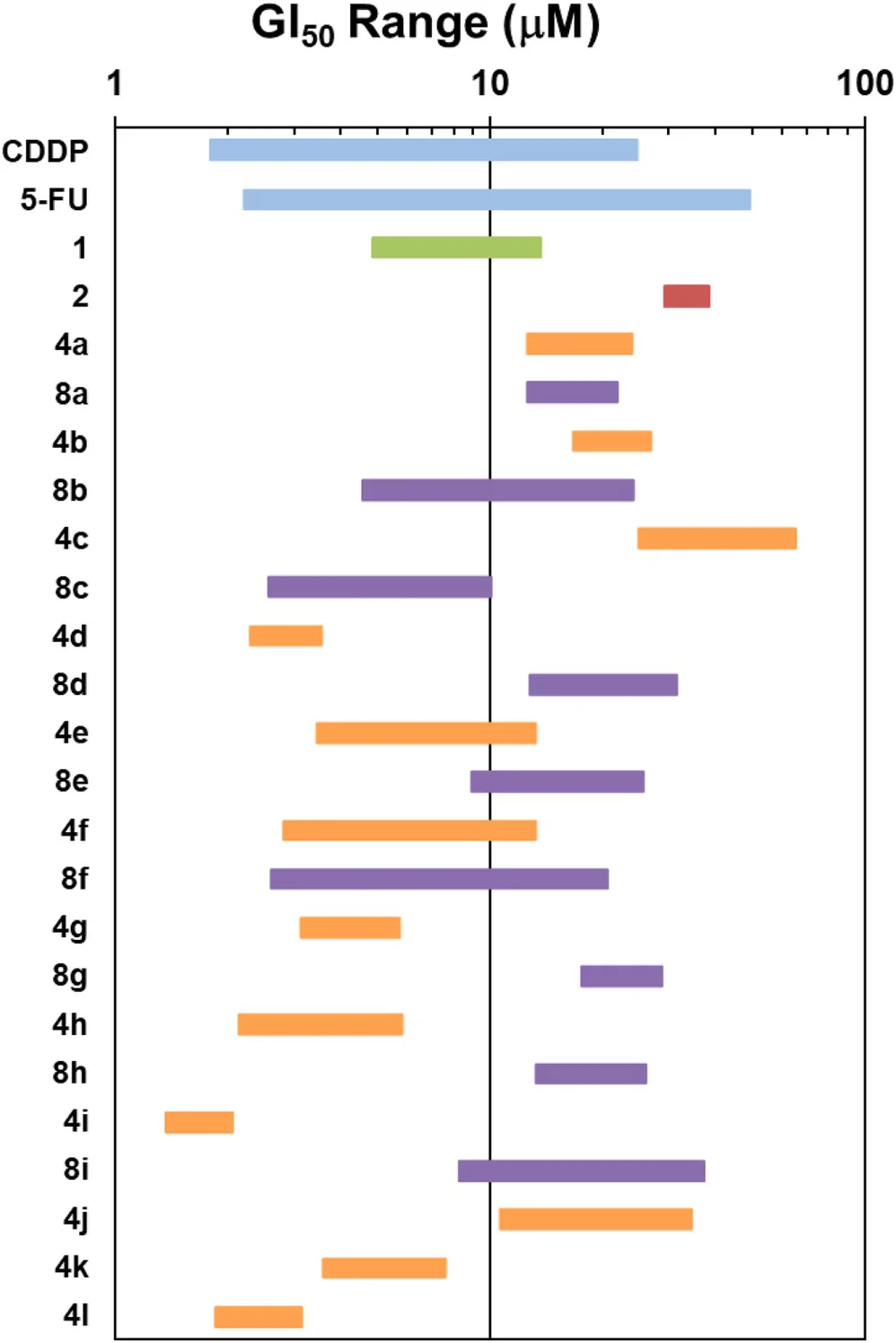
GI_50_ range plot of investigated compounds against human solid tumor cell lines. Bar colors indicate: reference anticancer drugs cisplatin (CDDP) and 5‐fluorouracil (5‐FU) (blue); tryptophan monomers (green and red); Trp‐X series (orange); and X‐Trp series (purple).

The antiproliferative activity of the tryptophan‐based dipeptides (**4a**–**l** and **8a**–**i**) was evaluated against human solid tumor cell lines—comprising A549 (lung carcinoma), HeLa (cervical carcinoma), MIA PaCa‐2 (pancreatic carcinoma), SW1573 (alveolar lung carcinoma), T‐47D (breast carcinoma), and WiDr (colorectal adenocarcinoma)—utilizing an in‐house implementation of the National Cancer Institute (NCI) protocols [12]. The clinical reference drugs cisplatin (CDDP) and 5‐fluorouracil (5‐FU) were included strictly as phenotypic references to establish a baseline for antiproliferative potency. The growth‐inhibitory effects, expressed as half‐maximal growth inhibition (GI_50_) values, are summarized in Table 1. To facilitate a comprehensive comparison of the bioactivity profiles across the cell lines, a visual overview is presented in the range plot (Figure 4).

**TABLE 1:** Antiproliferative activity (GI_50_, μM) against human solid tumor cell lines of tryptophan‐based dipeptides **4a**–**l** and **8a**–**i**.

	A549	HeLa	MIA PaCa‐2	SW1573	T‐47D	WiDr
*Trp‐X series*						
**4a**	16.7 (±2.2)	16.7 (±1.5)	12.6 (±3.9)	24 (±10)	13.3 (±5.0)	17.0 (±2.9)
**4b**	23.2 (±4.0)	17.6 (±1.0)	16.9 (±5.1)	27 (±12)	16.7 (±5.6)	26.9 (±5.8)
**4c**	50 (±17)	44.2 (±1.0)	28.6 (±9.6)	42 (±19)	24.9 (±1.7)	66 (±31)
**4d**	2.93 (±0.40)	2.29 (±0.46)	2.55 (±0.25)	3.56 (±0.48)	2.61 (±0.19)	3.31 (±0.59)
**4e**	3.5 (±1.0)	3.53 (±0.71)	13.2 (±6.2)	4.75 (±0.78)	8.0 (±2.3)	4.66 (±0.80)
**4f**	2.8 (±1.0)	2.99 (±0.99)	7.7 (±2.9)	2.86 (±0.15)	13.3 (±4.2)	10.5 (±4.5)
**4g**	3.51 (±0.96)	3.1 (±1.0)	3.83 (±0.60)	5.6 (±1.5)	5.76 (±0.78)	4.94 (±0.55)
**4h**	2.13 (±0.96)	3.7 (±1.2)	3.6 (±1.4)	5.9 (±2.0)	4.6 (±1.9)	3.6 (±1.1)
**4i**	1.36 (±0.11)	1.59 (±0.35)	2.00 (±0.14)	2.06 (±0.47)	1.98 (±0.24)	1.65 (±0.38)
**4j**	34.5 (±1.0)	28.1 (±6.7)	13.7 (±6.5)	10.7 (±2.4)	19.6 (±2.0)	32.1 (±3.3)
**4k**	3.58 (±0.93)	5.3 (±1.1)	6.24 (±0.91)	6.0 (±1.7)	6.7 (±1.1)	7.6 (±2.1)
**4l**	1.85 (±0.87)	2.24 (±0.13)	3.15 (±0.50)	2.86 (±0.25)	3.01 (±0.89)	2.56 (±0.27)
*X‐Trp series*						
**8a**	14.6 (±1.2)	13.3 (±1.0)	22.0 (±8.7)	12.9 (±2.6)	17.2 (±8.0)	12.6 (±4.3)
**8b**	9.5 (±2.7)	4.60 (±0.10)	24.2 (±6.5)	8.9 (±3.3)	13.4 (±2.7)	13.0 (±3.4)
**8c**	3.15 (±0.65)	2.97 (±0.29)	10.1 (±3.7)	2.57 (±0.59)	9.1 (±4.2)	3.77 (±0.39)
**8d**	17.2 (±1.2)	12.8 (±5.8)	31.5 (±2.2)	15.4 (±6.2)	24.9 (±4.1)	21.9 (±5.2)
**8e**	10.1 (±1.0)	8.9 (±3.4)	25.7 (±8.1)	9.7 (±2.8)	20.1 (±7.4)	12.0 (±1.0)
**8f**	4.35 (±0.34)	3.9 (±1.2)	20.6 (±5.7)	2.60 (±0.73)	6.5 (±1.2)	3.21 (±0.77)
**8g**	20.9 (±4.1)	20.5 (±1.0)	28.8 (±2.2)	17.6 (±7.2)	19.6 (±3.7)	18.9 (±5.1)
**8h**	15.2 (±3.6)	14.7 (±1.5)	26.1 (±2.6)	14.5 (±2.5)	18.4 (±2.2)	13.3 (±1.0)
**8i**	8.4 (±3.6)	16.2 (±4.5)	37.4 (±2.1)	8.83 (±0.70)	32.1 (±7.0)	26 (±11)
*Standard drugs*						
CDDP	4.93 (±0.18)	1.98 (±0.70)	1.60 (±0.27)	3.21 (±0.91)	16.9 (±3.3)	23.0 (±4.3)
5‐FU	2.25 (±0.26)	16.2 (±4.5)	4.5 (±1.2)	3.3 (±1.2)	43 (±16)	49.3 (±6.7)

*Note:* Values represent mean ± standard deviation of at least three independent experiments.

A detailed analysis of the biological data gathered in Table 1 reveals prominent structure–activity relationships governed by the sequential arrangement of the peptide backbone, side‐chain lipophilicity, and localized steric factors. The first overarching trend is the clear superiority of the Trp‐X series (C‐series, **4a**–**l**) over their X‐Trp counter‐isomers (*N*‐series, **8a**–**i**). In the majority of paired sets, positioning the L‐tryptophan core at the *N*‐terminus while anchoring the modifying amino acid at the *C*‐terminal ester framework provides vastly superior growth inhibition. This strategic topological arrangement suggests that the precise spatial exposure of the indole moiety, combined with the presence of the flanking *N,N*‐dibenzyl protection group at the *N*‐terminus, establishes a key pharmacophoric conformation essential for cell membrane interactions or potential transporter recognition pathways.

Within the highly active Trp‐X series, the side chain of the coupled C‐terminal residue exerts a crucial regulatory effect on potency. The L‐proline conjugate (**4i**) emerged as the most potent antineoplastic architecture of the entire chemical library, demonstrating consistent single‐digit sub‐micromolar growth inhibition across all tested solid tumor lines, with GI_50_ values spanning from 1.36 μM (A549) to 2.06 μM (SW1573). This outstanding performance significantly surpasses the clinical reference drugs CDDP and 5‐FU in resistant lines such as T‐47D breast cancer cells and WiDr colorectal models. The exceptional activity of **4i** is attributed to the conformationally constrained pyrrolidine ring, which freezes the peptide conformation into a highly productive bioactive state, maximizing cellular accumulation. Similarly, the lipophilic aliphatic and aromatic cores of L‐methionine (**4l**), L‐leucine (**4d**), and L‐phenylalanine (**4e**) consistently drive robust growth inhibition. Conversely, increasing the steric bulk at the carbon directly adjacent to the backbone drastically impairs activity (GI_50_ > 10 μM), as seen with L‐valine (**4j**) and the highly extended L‐phenylglycine derivative (**4c**), which marked the lowest potency profile of the group in WiDr cells. Furthermore, removing the side chain altogether (**4a**, glycine) or adding polar features without extended aliphatic chains (**4b**, alanine) causes a prominent drop in the general antiproliferative baseline.

When investigating the reverse X‐Trp series (**8a**–**i**), the overall biological performance is severely diminished, yet a unique and contrasting SAR behavior stands out. Most notably, swapping the structural sequence of the unbranched or aliphatic residues leads to a significant loss of activity. However, an intriguing inversion of behavior is witnessed with L‐phenylglycine. While its *C*‐series isomer (**4c**) is virtually inactive, its *N*‐series counterpart (**8c**) displays a remarkable increase in antiproliferative efficacy, achieving promising GI_50_ values below 5 μM against SW1573 and A549, both lung cells. A similar sequence‐dependent rescue of performance is displayed by the aromatic L‐tyrosine (**8f**)—except in MIA PaCa‐2—and the hydroxylated architectures of L‐threonine (**8g**) and L‐serine (**8h**). While their *C*‐series counterparts suffer from poor conversions or low chemical yield parameters, the *N*‐series placement rearranges the spatial configuration of the structural shielding, allowing the free polar functions to participate in favorable localized binding with secondary tumor targets.

In conclusion, these results demonstrate that the anticancer profile of these short tryptophyl‐dipeptides is not merely a consequence of aggregate lipophilicity. Instead, it is strictly governed by a highly directional, sequence‐specific molecular topology where the spatial pairing of the *N*,*N*‐dibenzyl anchor and the flexible indole ring controls cell‐line selectivity. As this investigation focuses on mapping structure–activity relationships within a novel library template, full mechanistic elucidation and target‐validation studies are deferred to subsequent development phases.

## 4 Conclusions

In this study, we designed, synthesized, and biologically evaluated two complementary series of novel tryptophan‐derived dipeptides (Trp‐X and X‐Trp) to analyze how sequence inversion alters both chemical accessibility and in vitro anticancer activity against human solid tumors. From a synthetic standpoint, reversing the amino acid sequence highlights the profound role of steric hindrance; while the *C*‐series allowed straightforward couplings, the *N*‐series presented significant challenges, such as the chemical inertia of valine and isoleucine methyl esters under basic hydrolysis and competing multi‐alkylation pathways for L‐methionine.

Biologically, the dual‐series screening framework demonstrated that antiproliferative activity is strictly sequence‐dependent and governed by a precise three‐dimensional molecular topology rather than aggregate lipophilicity alone. The Trp‐X series emerged as the superior architectural template, with the conformationally restricted L‐proline conjugate (**4i**) identified as the lead compound of the entire library. Compound **4i** displayed consistent, single‐digit sub‐micromolar growth inhibition across all tested lines (GI_50_ = 1.36–2.06 μM). Interestingly, an intriguing inversion of structure–activity relationships was witnessed in the reverse X‐Trp series, where specific residues like L‐phenylglycine (**8c**) and L‐tyrosine (**8f**) experienced a prominent rescue of potency upon sequence relocation. Taken together, these findings confirm that strategic positioning of the L‐tryptophan core coupled with an *N*,*N*‐dibenzyl anchor represents a privileged scaffold for anticancer drug design, providing a solid foundation for further structural optimization and mechanism‐of‐action studies.

While the present study establishes a robust structural framework and clear SAR guidelines demonstrating the superior potency of the Trp‐X layout, uncovering the underlying biological pathways remains critical. To this end, comprehensive follow‐up investigations focusing on the precise molecular mode of action of these prominent leads (such as **4i**) have been independently conducted. These advanced phenotypic evaluations reveal that these minimalist dipeptide architectures drive cell growth inhibition via profound intracellular metabolic disruption and stress‐induced autophagic flux, rather than conventional apoptotic lysis.

## Funding

This project was financed by the Spanish Government (Project PID2021‐123059OB‐I00 funded by MCIN/AEI/FEDER, UE).

## Conflicts of Interest

The authors declare no conflicts of interest.

## Supporting information


**Figure S1:** 1H‐NMR spectrum of methyl *N*,*N*‐dibenzyl‐L‐tryptophanate (**2**).
**Figure S2:** 13C‐NMR spectrum of methyl *N*,*N*‐dibenzyl‐L‐tryptophanate (**2**).
**Figure S3:** HRMS spectrum of methyl *N*,*N*‐dibenzyl‐L‐tryptophanate (**2**).
**Figure S4:** 1H‐NMR spectrum of N,N‐dibenzyl‐L‐tryptophan (**3**).
**Figure S5:** 13C‐NMR spectrum of *N*,*N*‐dibenzyl‐L‐tryptophan (**3**).
**Figure S6:** HRMS spectrum of *N*,*N*‐dibenzyl‐L‐tryptophan (**3**).
**Figure S7:** 1H spectrum of methyl *N*,*N*‐dibenzyl‐L‐tryptophyl‐glycinate (**4a**).
**Figure S8:** 13C spectrum of methyl *N*,*N*‐dibenzyl‐L‐tryptophyl‐glycinate (**4a**).
**Figure S9:** HRMS spectrum of methyl *N*,*N*‐dibenzyl‐L‐tryptophyl‐glycinate (**4a**).
**Figure S10:** 1H spectrum of methyl *N*,*N*‐dibenzyl‐L‐tryptophyl‐L‐alaninate (**4b**).
**Figure S11:** 13C spectrum of methyl *N,N*‐dibenzyl‐L‐tryptophyl‐L‐alaninate (**4b**).
**Figure S12:** HRMS spectrum of methyl *N*,*N*‐dibenzyl‐L‐tryptophyl‐L‐alaninate (**4b**).
**Figure S13:** 1H spectrum of methyl *N*,*N*‐dibenzyl‐L‐tryptophyl‐L‐phenylglycinate (**4c**).
**Figure S14:** 13C spectrum of methyl *N*,*N*‐dibenzyl‐L‐tryptophyl‐L‐phenylglycinate (**4c**).
**Figure S15:** HRMS spectrum of methyl *N*,*N*‐dibenzyl‐L‐tryptophyl‐L‐phenylglycinate (**4c**).
**Figure S16:** 1H spectrum of methyl *N*,*N*‐dibenzyl‐L‐tryptophyl‐L‐leucinate (**4d**).
**Figure S17:** 13C spectrum of methyl *N*,*N*‐dibenzyl‐L‐tryptophyl‐L‐leucinate (**4d**).
**Figure S18:** HRMS spectrum of methyl *N*,*N*‐dibenzyl‐L‐tryptophyl‐L‐leucinate (**4d**).
**Figure S19:** 1H spectrum of methyl *N*,*N*‐dibenzyl‐L‐tryptophyl‐L‐phenylalaninate (**4e**).
**Figure S20:** 13C spectrum of methyl *N*,*N*‐dibenzyl‐L‐tryptophyl‐L‐phenylalaninate (**4e**).
**Figure S21:** HRMS spectrum of methyl *N*,*N*‐dibenzyl‐L‐tryptophyl‐L‐phenylalaninate (**4e**).
**Figure S22:** 1H spectrum of methyl *N*,*N*‐dibenzyl‐L‐tryptophyl‐L‐tyrosinate (**4f**).
**Figure S23:** 13C spectrum of methyl *N*,*N*‐dibenzyl‐L‐tryptophyl‐L‐tyrosinate (**4f**).
**Figure S24:** HRMS spectrum of methyl *N*,*N*‐dibenzyl‐L‐tryptophyl‐L‐tyrosinate (**4f**).
**Figure S25:** 1H spectrum of methyl *N*,*N*‐dibenzyl‐L‐tryptophyl‐L‐threoninate (**4g**).
**Figure S26:** 13C spectrum of methyl *N*,*N*‐dibenzyl‐L‐tryptophyl‐L‐threoninate (**4g**).
**Figure S27:** HRMS spectrum of methyl *N*,*N*‐dibenzyl‐L‐tryptophyl‐L‐threoninate (**4g**).
**Figure S28:** 1H spectrum of methyl *N*,*N*‐dibenzyl‐L‐tryptophyl‐L‐serinate (**4h**).
**Figure S29:** 13C spectrum of methyl *N*,*N*‐dibenzyl‐L‐tryptophyl‐L‐serinate (**4h**).
**Figure S30:** HRMS spectrum of methyl *N*,*N*‐dibenzyl‐L‐tryptophyl‐L‐serinate (**4h**).
**Figure S31:** 1H spectrum of methyl *N,N‐*dibenzyl‐L‐tryptophyl‐L‐prolinate (**4i**).
**Figure S32:** 13C spectrum of methyl *N,N‐*dibenzyl‐L‐tryptophyl‐L*‐*prolinate (**4i**).
**Figure S33:** HRMS spectrum of methyl *N,N‐*dibenzyl‐L‐tryptophyl‐L*‐*prolinate (**4i**).
**Figure S34:** 1H spectrum of methyl *N,N‐*dibenzyl‐L‐tryptophyl‐L*‐*valinate (**4j**).
**Figure S35:** 13C spectrum of methyl *N,N‐*dibenzyl‐L‐tryptophyl‐L*‐*valinate (**4j**).
**Figure S36:** HRMS spectrum of methyl *N,N‐*dibenzyl‐L‐tryptophyl‐L‐valinate (**4j**).
**Figure S37:** 1H spectrum of methyl *N,N‐*dibenzyl‐L‐tryptophyl‐L‐isoleucinate (**4k**).
**Figure S38:** 13C spectrum of methyl *N,N‐*dibenzyl‐L‐tryptophyl‐L‐isoleucinate (**4k**).
**Figure S39:** HRMS spectrum of methyl *N,N‐*dibenzyl‐L‐tryptophyl‐L‐isoleucinate (**4k**).
**Figure S40:** 1H spectrum of methyl *N,N‐*dibenzyl‐L‐tryptophyl‐L‐methioninate (**4l**).
**Figure S41:** 13C spectrum of methyl *N,N‐*dibenzyl‐L‐tryptophyl‐L‐methioninate (**4l**).
**Figure S42:** HRMS spectrum of methyl *N,N‐*dibenzyl‐L‐tryptophyl‐L*‐*methioninate (**4l**).
**Figure S43:** 1H spectrum of *N,N‐*dibenzyl‐glycine (**7a**).
**Figure S44:** 13C spectrum of *N,N*‐dibenzyl‐glycine (**7a**).
**Figure S45:** HRMS spectrum of *N,N‐*dibenzyl‐glycine (**7a**).
**Figure S46:** 1H spectrum of *N,N‐*dibenzyl‐L‐alanine (**7b**).
**Figure S47:** 13C spectrum of *N,N‐*dibenzyl‐L‐alanine (**7b**).
**Figure S48:** HRMS spectrum of *N,N‐*dibenzyl‐L‐alanine (**7b**).
**Figure S49:** 1H spectrum of *N,N‐*dibenzyl‐L‐phenylglycine (**7c**).
**Figure S50:** 13C spectrum of *N,N‐*dibenzyl‐L‐phenylglycine (**7c**).
**Figure S51:** HRMS spectrum of *N,N‐*dibenzyl*‐*L‐phenylglycine (**7c**).
**Figure S52:** 1H spectrum of *N,N‐*dibenzyl‐L‐leucine (**7d**).
**Figure S53:** 13C spectrum of *N,N‐*dibenzyl‐L‐leucine (**7d**).
**Figure S54:** HRMS spectrum of *N,N‐*dibenzyl*‐*L‐leucine (**7d**).
**Figure S55:** 1H spectrum of *N,N‐*dibenzyl‐L‐phenylalanine (**7e**).
**Figure S56:** 13C spectrum of *N,N‐*dibenzyl*‐*L‐phenylalanine (**7e**).
**Figure S57:** HRMS spectrum of *N,N‐*dibenzyl*‐*L‐phenylalanine (**7e**).
**Figure S58:** 1H spectrum of *N,N‐*dibenzyl‐L‐tyrosine (**7f**).
**Figure S59:** 13C spectrum of *N,N‐*dibenzyl*‐*L‐tyrosine (**7f**).
**Figure S60:** HRMS spectrum of *N,N‐*dibenzyl‐L‐tyrosine (**7f**).
**Figure S61:** 1H spectrum of *N,N‐*dibenzyl‐L‐threonine (**7g**).
**Figure S62:** 13C spectrum of *N,N‐*dibenzyl‐L‐threonine (**7g**).
**Figure S63:** HRMS spectrum of *N,N‐*dibenzyl‐L‐threonine (**7g**).
**Figure S64:** 1H spectrum of *N,N‐*dibenzyl‐L‐serine (**7h**).
**Figure S65:** 13C spectrum of *N,N‐*dibenzyl‐L‐serine (**7h**).
**Figure S66:** HRMS spectrum of *N,N‐*dibenzyl‐L‐serine (**7h**).
**Figure S67:** 1H spectrum of *N*‐benzyl‐L‐proline (**7i**).
**Figure S68:** 13C spectrum of *N*‐benzyl‐L‐proline (**7i**).
**Figure S69:** HRMS spectrum of *N*‐benzyl‐L‐proline (**7i**).
**Figure S70:** 1H spectrum of methyl dibenzylglycyl‐L‐tryptophanate (**8a**).
**Figure S71:** 13C spectrum of methyl dibenzylglycyl‐L‐tryptophanate (**8a**).
**Figure S72:** HRMS spectrum of methyl dibenzylglycyl‐L‐tryptophanate (**8a**).
**Figure S73:** 1H spectrum of methyl dibenzyl‐L‐alanyl‐L‐tryptophanate (**8b**).
**Figure S74:** 13C spectrum of methyl dibenzyl‐L‐alanyl‐L‐tryptophanate (**8b**).
**Figure S75:** HRMS spectrum of methyl dibenzyl‐L‐alanyl‐L‐tryptophanate (**8b**).
**Figure S76:** 1H spectrum of methyl ((S)‐2‐(dibenzylamino)‐2‐phenylacetyl)‐L‐tryptophanate (**8c**).
**Figure S77:** 13C spectrum of methyl ((S)‐2‐(dibenzylamino)‐2‐phenylacetyl)‐L‐tryptophanate (**8c**).
**Figure S78:** HRMS spectrum of methyl ((S)‐2‐(dibenzylamino)‐2‐phenylacetyl)‐L‐tryptophanate (**8c**).
**Figure S79:** 1H spectrum of methyl dibenzyl‐L‐leucyl‐L‐tryptophanate (**8d**).
**Figure S80:** 13C spectrum of methyl dibenzyl‐L‐leucyl‐L‐tryptophanate (**8d**).
**Figure S81:** HRMS spectrum of methyl dibenzyl‐L‐leucyl‐L‐tryptophanate (**8d**).
**Figure S82:** 1H spectrum of methyl dibenzyl‐L‐phenylalanyl‐L‐tryptophanate (**8e**).
**Figure S83:** 13C spectrum of methyl dibenzyl‐L‐phenylalanyl‐L‐tryptophanate (**8e**).
**Figure S84:** HRMS spectrum of methyl dibenzyl‐L‐phenylalanyl‐L‐tryptophanate (**8e**).
**Figure S85:** 1H spectrum of methyl dibenzyl‐L‐tyrosyl‐L‐tryptophanate (**8f**).
**Figure S86:** 13C spectrum of methyl dibenzyl‐L‐tyrosyl‐L‐tryptophanate (**8f**).
**Figure S87:** HRMS spectrum of methyl dibenzyl‐L‐tyrosyl‐L‐tryptophanate (**8f**).
**Figure S88:** 1H spectrum of methyl dibenzyl‐L‐threonyl‐L‐tryptophanate (**8g**).
**Figure S89:** 13C spectrum of methyl dibenzyl‐L‐threonyl‐L‐tryptophanate (**8g**).
**Figure S90:** HRMS spectrum of methyl dibenzyl‐L‐threonyl‐L‐tryptophanate (**8g**).
**Figure S91:** 1H spectrum of methyl dibenzyl‐L‐seryl‐L‐tryptophanate (**8h**).
**Figure S92:** 13C spectrum of methyl dibenzyl‐L‐seryl‐L‐tryptophanate (**8h**).
**Figure S93:** HRMS spectrum of methyl dibenzyl‐L‐seryl‐L‐tryptophanate (**8h**).
**Figure S94:** 1H spectrum of methyl dibenzyl‐L‐prolyl‐L‐tryptophanate (**8h**).
**Figure S94:** 13C spectrum of methyl dibenzyl‐L‐prolyl‐L‐tryptophanate (**8h**).
**Figure S95:** HRMS spectrum of methyl dibenzyl‐L‐prolyl‐L‐tryptophanate (**8h**).

## Data Availability

The data that support the findings of this study are available on request from the corresponding authors.
