# Supplementary material for: Sequence Inversion Dictates the Antiproliferative Activity of Bidirectional Tryptophan‐Containing Dipeptide Libraries
Source: J Pept Sci. 2026 Sep 16;32(10):e70128. doi: 10.1002/psc.70128 (PMC13581466; doi:10.1002/psc.70128)
Supplement: Supplementary file 1 [file PSC-32-e70128-s001.pdf]

Supplementary information

Sequence inversion dictates the antiproliferative activity of bidirectional tryptophan-containing dipeptide libraries

J. Benamer Díaz, Aday González-Bakker, Adam N. Khan, José M. Padrón*

BioLab, Instituto Universitario de Bio-Orgánica Antonio González, Universidad de La Laguna, Astrofísico Francisco Sánchez 2, 38206 La Laguna, Spain

* To whom correspondence should be submitted.

Corresponding author email: jmpadron@ull.es

Table of contents

Fig S1 ^{1}H-NMR spectrum of methyl *N*,*N*-dibenzyl-L-tryptophanate (**2**) 4
Fig S2 ^{13}C-NMR spectrum of methyl *N*,*N*-dibenzyl-L-tryptophanate (**2**) 4
Fig S3 HRMS spectrum of methyl *N*,*N*-dibenzyl-L-tryptophanate (**2**) 5
Fig S4 ^{1}H-NMR spectrum of N,N-dibenzyl-L-tryptophan (**3**) 5
Fig S5 ^{13}C-NMR spectrum of *N*,*N*-dibenzyl-L-tryptophan (**3**) 6
Fig S6 HRMS spectrum of *N*,*N*-dibenzyl-L-tryptophan (**3**) 6
Fig S7 ^{1}H spectrum of methyl *N*,*N*-dibenzyl-L-tryptophyl-glycinate (**4a**) 7
Fig S8 ^{13}C spectrum of methyl *N*,*N*-dibenzyl-L-tryptophyl-glycinate (**4a**) 7
Fig S9 HRMS spectrum of methyl *N*,*N*-dibenzyl-L-tryptophyl-glycinate (**4a**) 8
Fig S10 ^{1}H spectrum of methyl *N*,*N*-dibenzyl-L-tryptophyl-L-alaninate (**4b**) 8
Fig S11 ^{13}C spectrum of methyl *N,N*-dibenzyl-L-tryptophyl-L-alaninate (**4b**) 9
Fig S12 HRMS spectrum of methyl *N*,*N*-dibenzyl-L-tryptophyl-L-alaninate (**4b**) 9
Fig S13 ^{1}H spectrum of methyl *N*,*N*-dibenzyl-L-tryptophyl-L-phenylglycinate (**4c**) 10
Fig S14 ^{13}C spectrum of methyl *N*,*N*-dibenzyl-L-tryptophyl-L-phenylglycinate (**4c**) 10
Fig S15 HRMS spectrum of methyl *N*,*N*-dibenzyl-L-tryptophyl-L-phenylglycinate (**4c**) 11
Fig S16 ^{1}H spectrum of methyl *N*,*N*-dibenzyl-L-tryptophyl-L-leucinate (**4d**) 11
Fig S17 ^{13}C spectrum of methyl *N*,*N*-dibenzyl-L-tryptophyl-L-leucinate (**4d**) 12
Fig S18 HRMS spectrum of methyl *N*,*N*-dibenzyl-L-tryptophyl-L-leucinate (**4d**) 12
Fig S19 ^{1}H spectrum of methyl *N*,*N*-dibenzyl-L-tryptophyl-L-phenylalaninate (**4e**) 13
Fig S20 ^{13}C spectrum of methyl *N*,*N*-dibenzyl-L-tryptophyl-L-phenylalaninate (**4e**) 13

Fig S21 HRMS spectrum of methyl *N*,*N*-dibenzyl-L-tryptophyl-L-phenylalaninate (**4e**)............................. 14
Fig S22 ^{1}H spectrum of methyl *N*,*N*-dibenzyl-L-tryptophyl-L-tyrosinate (**4f**)... 14
Fig S23 ^{13}C spectrum of methyl *N*,*N*-dibenzyl-L-tryptophyl-L-tyrosinate (**4f**)... 15
Fig S24 HRMS spectrum of methyl *N*,*N*-dibenzyl-L-tryptophyl-L-tyrosinate (**4f**) 15
Fig S25 ^{1}H spectrum of methyl *N*,*N*-dibenzyl-L-tryptophyl-L-threoninate (**4g**) 16
Fig S26 ^{13}C spectrum of methyl *N*,*N*-dibenzyl-L-tryptophyl-L-threoninate (**4g**) 16
Fig S27 HRMS spectrum of methyl *N*,*N*-dibenzyl-L-tryptophyl-L-threoninate (**4g**)................................... 17
Fig S28 ^{1}H spectrum of methyl *N*,*N*-dibenzyl-L-tryptophyl-L-serinate (**4h**).. 17
Fig S29 ^{13}C spectrum of methyl *N*,*N*-dibenzyl-L-tryptophyl-L-serinate (**4h**) 18
Fig S30 HRMS spectrum of methyl *N*,*N*-dibenzyl-L-tryptophyl-L-serinate (**4h**)... 18
Fig S31 ^{1}H spectrum of methyl *N,N*-dibenzyl-L-tryptophyl-L-prolinate (**4i**).. 19
Fig S32 ^{13}C spectrum of methyl *N,N*-dibenzyl-L-tryptophyl-L-prolinate (**4i**)... 19
Fig S33 HRMS spectrum of methyl *N,N*-dibenzyl-L-tryptophyl-L-prolinate (**4i**) 20
Fig S34 ^{1}H spectrum of methyl *N,N*-dibenzyl-L-tryptophyl-L-valinate (**4j**) 20
Fig S35 ^{13}C spectrum of methyl *N,N*-dibenzyl-L-tryptophyl-L-valinate (**4j**) 21
Fig S36 HRMS spectrum of methyl *N,N*-dibenzyl-L-tryptophyl-L-valinate (**4j**)... 21
Fig S37 ^{1}H spectrum of methyl *N,N*-dibenzyl-L-tryptophyl-L-isoleucinate (**4k**).. 22
Fig S38 ^{13}C spectrum of methyl *N,N*-dibenzyl-L-tryptophyl-L-isoleucinate (**4k**) 22
Fig S39 HRMS spectrum of methyl *N,N*-dibenzyl-L-tryptophyl-L-isoleucinate (**4k**)................................. 23
Fig S40 ^{1}H spectrum of methyl *N,N*-dibenzyl-L-tryptophyl-L-methioninate (**4l**) 24
Fig S41 ^{13}C spectrum of methyl *N,N*-dibenzyl-L-tryptophyl-L-methioninate (**4l**)... 24
Fig S42 HRMS spectrum of methyl *N,N*-dibenzyl-L-tryptophyl-L-methioninate (**4l**) 25
Fig S43 ^{1}H spectrum of *N,N*-dibenzyl-glycine (**7a**).. 25
Fig S44 ^{13}C spectrum of *N,N*-dibenzyl-glycine (**7a**) 26
Fig S45 HRMS spectrum of *N,N*-dibenzyl-glycine (**7a**).. 26
Fig S46 ^{1}H spectrum of *N,N*-dibenzyl-L-alanine (**7b**).. 27
Fig S47 ^{13}C spectrum of *N,N*-dibenzyl-L-alanine (**7b**) 27
Fig S48 HRMS spectrum of *N,N*-dibenzyl-L-alanine (**7b**)... 28
Fig S49 ^{1}H spectrum of *N,N*-dibenzyl-L-phenylglycine (**7c**).. 28
Fig S50 ^{13}C spectrum of *N,N*-dibenzyl-L-phenylglycine (**7c**) 29
Fig S51 HRMS spectrum of *N,N*-dibenzyl-L-phenylglycine (**7c**) 29
Fig S52 ^{1}H spectrum of *N,N*-dibenzyl-L-leucine (**7d**)... 30
Fig S53 ^{13}C spectrum of *N,N*-dibenzyl-L-leucine (**7d**)... 30
Fig S54 HRMS spectrum of *N,N*-dibenzyl-L-leucine (**7d**) 31
Fig S55 ^{1}H spectrum of *N,N*-dibenzyl-L-phenylalanine (**7e**) 31
Fig S56 ^{13}C spectrum of *N,N*-dibenzyl-L-phenylalanine (**7e**)... 32
Fig S57 HRMS spectrum of *N,N*-dibenzyl-L-phenylalanine (**7e**)... 32
Fig S58 ^{1}H spectrum of *N,N*-dibenzyl-L-tyrosine (**7f**)... 33
Fig S59 ^{13}C spectrum of *N,N*-dibenzyl-L-tyrosine (**7f**) 33

Fig S60 HRMS spectrum of *N,N*-dibenzyl-L-tyrosine (**7f**) 34
Fig S61 ^{1}H spectrum of *N,N*-dibenzyl-L-threonine (**7g**) 34
Fig S62 ^{13}C spectrum of *N,N*-dibenzyl-L-threonine (**7g**) 35
Fig S63 HRMS spectrum of *N,N*-dibenzyl-L-threonine (**7g**) 35
Fig S64 ^{1}H spectrum of *N,N*-dibenzyl-L-serine (**7h**) 36
Fig S65 ^{13}C spectrum of *N,N*-dibenzyl-L-serine (**7h**) 36
Fig S66 HRMS spectrum of *N,N*-dibenzyl-L-serine (**7h**) 37
Fig S67 ^{1}H spectrum of *N*-benzyl-L-proline (**7i**) 37
Fig S68 ^{13}C spectrum of *N*-benzyl-L-proline (**7i**) 38
Fig S69 HRMS spectrum of *N*-benzyl-L-proline (**7i**) 38
Fig S70 ^{1}H spectrum of methyl dibenzylglycyl-L-tryptophanate (**8a**) 39
Fig S71 ^{13}C spectrum of methyl dibenzylglycyl-L-tryptophanate (**8a**) 39
Fig S72 HRMS spectrum of methyl dibenzylglycyl-L-tryptophanate (**8a**) 40
Fig S73 ^{1}H spectrum of methyl dibenzyl-L-alanyl-L-tryptophanate (**8b**) 40
Fig S74 ^{13}C spectrum of methyl dibenzyl-L-alanyl-L-tryptophanate (**8b**) 41
Fig S75 HRMS spectrum of methyl dibenzyl-L-alanyl-L-tryptophanate (**8b**) 41
Fig S76 ^{1}H spectrum of methyl ((S)-2-(dibenzylamino)-2-phenylacetyl)-L-tryptophanate (**8c**) 42
Fig S77 ^{13}C spectrum of methyl ((S)-2-(dibenzylamino)-2-phenylacetyl)-L-tryptophanate (**8c**) 43
Fig S78 HRMS spectrum of methyl ((S)-2-(dibenzylamino)-2-phenylacetyl)-L-tryptophanate (**8c**) 43
Fig S79 ^{1}H spectrum of methyl dibenzyl-L-leucyl-L-tryptophanate (**8d**) 44
Fig S80 ^{13}C spectrum of methyl dibenzyl-L-leucyl-L-tryptophanate (**8d**) 44
Fig S81 HRMS spectrum of methyl dibenzyl-L-leucyl-L-tryptophanate (**8d**) 45
Fig S82 ^{1}H spectrum of methyl dibenzyl-L-phenylalanyl-L-tryptophanate (**8e**) 45
Fig S83 ^{13}C spectrum of methyl dibenzyl-L-phenylalanyl-L-tryptophanate (**8e**) 46
Fig S84 HRMS spectrum of methyl dibenzyl-L-phenylalanyl-L-tryptophanate (**8e**) 46
Fig S85 ^{1}H spectrum of methyl dibenzyl-L-tyrosyl-L-tryptophanate (**8f**) 46
Fig S86 ^{13}C spectrum of methyl dibenzyl-L-tyrosyl-L-tryptophanate (**8f**) 47
Fig S87 HRMS spectrum of methyl dibenzyl-L-tyrosyl-L-tryptophanate (**8f**) 47
Fig S88 ^{1}H spectrum of methyl dibenzyl-L-threonyl-L-tryptophanate (**8g**) 48
Fig S89 ^{13}C spectrum of methyl dibenzyl-L-threonyl-L-tryptophanate (**8g**) 48
Fig S90 HRMS spectrum of methyl dibenzyl-L-threonyl-L-tryptophanate (**8g**) 49
Fig S91 ^{1}H spectrum of methyl dibenzyl-L-seryl-L-tryptophanate (**8h**) 50
Fig S92 ^{13}C spectrum of methyl dibenzyl-L-seryl-L-tryptophanate (**8h**) 50
Fig S93 HRMS spectrum of methyl dibenzyl-L-seryl-L-tryptophanate (**8h**) 51
Fig S94 ^{1}H spectrum of methyl dibenzyl-L-prolyl-L-tryptophanate (**8h**) 51
Fig S94 ^{13}C spectrum of methyl dibenzyl-L-prolyl-L-tryptophanate (**8h**) 52
Fig S95 HRMS spectrum of methyl dibenzyl-L-prolyl-L-tryptophanate (**8h**) 52

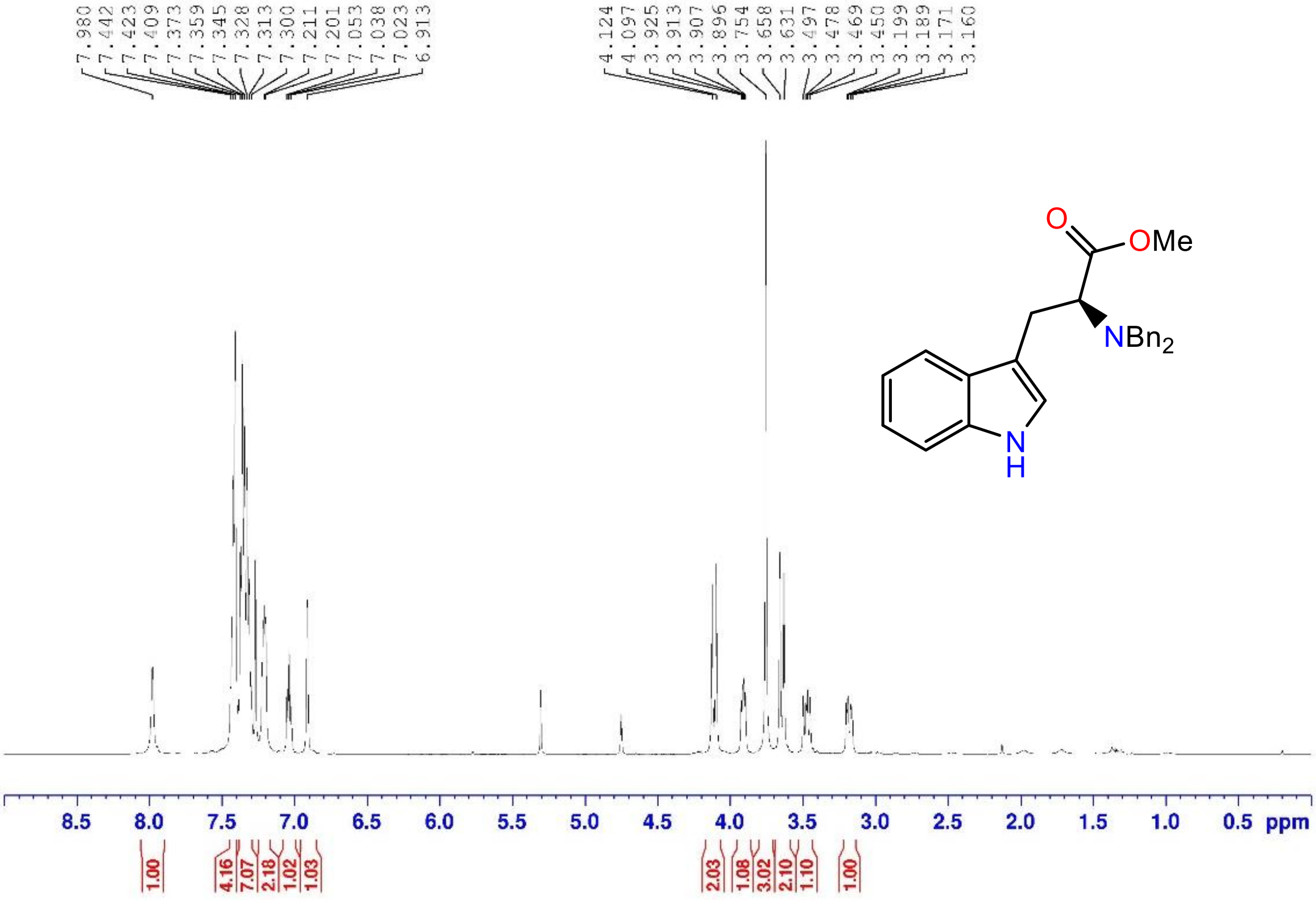

Fig S1 ^{1}H-NMR spectrum of methyl *N*,*N*-dibenzyl-L-tryptophanate (**2**)

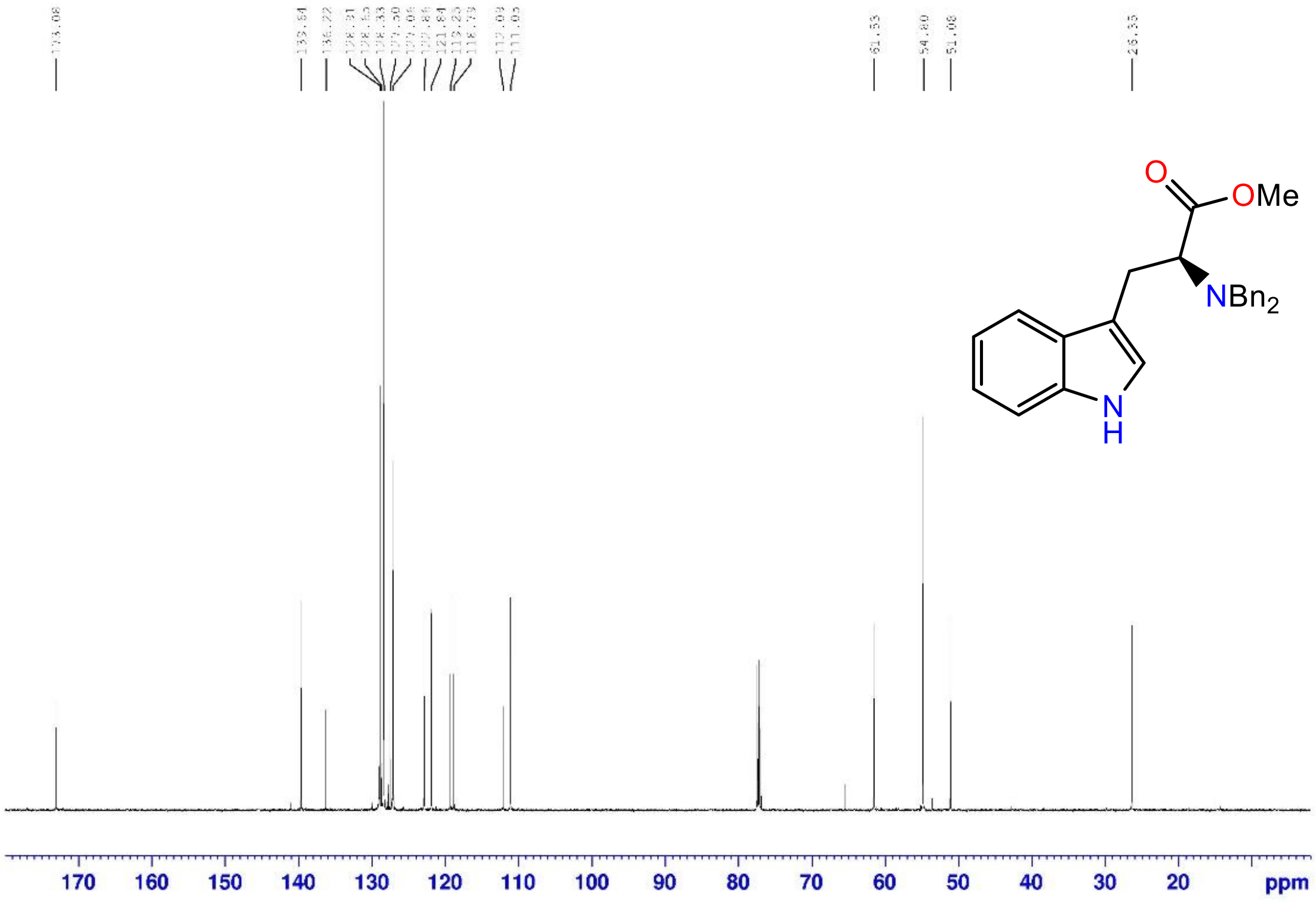

Fig S2 ^{13}C-NMR spectrum of methyl *N*,*N*-dibenzyl-L-tryptophanate (**2**)

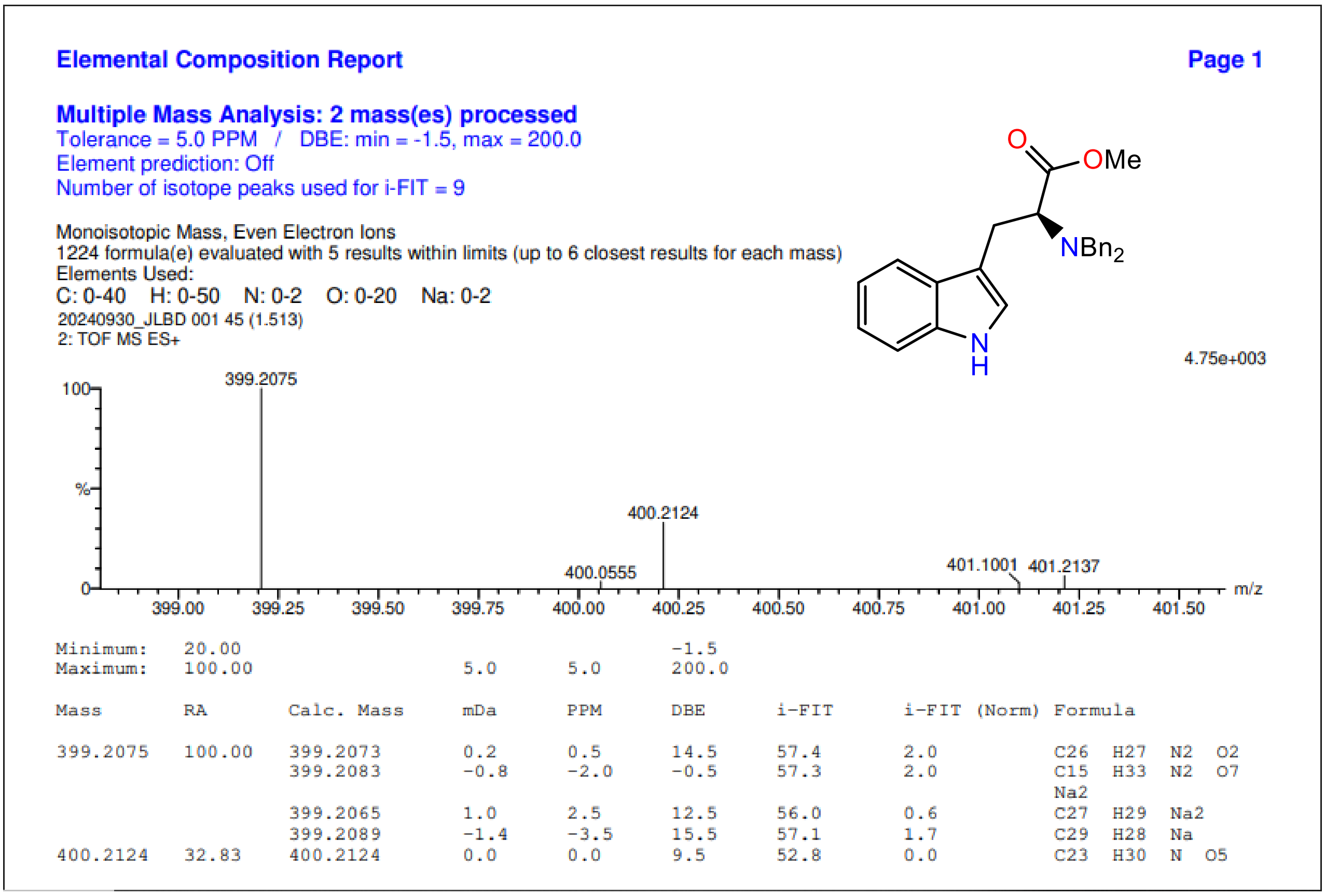

Elemental Composition Report **Page 1**

Multiple Mass Analysis: 2 mass(es) processed
Tolerance = 5.0 PPM / DBE: min = -1.5, max = 200.0
Element prediction: Off
Number of isotope peaks used for i-FIT = 9

Monoisotopic Mass, Even Electron Ions
1224 formula(e) evaluated with 5 results within limits (up to 6 closest results for each mass)
Elements Used:
C: 0-40 H: 0-50 N: 0-2 O: 0-20 Na: 0-2
20240930_JLBD 001 45 (1.513)
2: TOF MS ES+

Minimum:	20.00				-1.5			
Maximum:	100.00		5.0	5.0	200.0			
Mass	RA	Calc. Mass	mDa	PPM	DBE	i-FIT	i-FIT (Norm)	Formula
399.2075	100.00	399.2073	0.2	0.5	14.5	57.4	2.0	C26 H27 N2 O2
		399.2083	-0.8	-2.0	-0.5	57.3	2.0	C15 H33 N2 O7 Na2
		399.2065	1.0	2.5	12.5	56.0	0.6	C27 H29 Na2
		399.2089	-1.4	-3.5	15.5	57.1	1.7	C29 H28 Na
400.2124	32.83	400.2124	0.0	0.0	9.5	52.8	0.0	C23 H30 N O5

Fig S3 HRMS spectrum of methyl *N*,*N*-dibenzyl-L-tryptophanate (**2**)

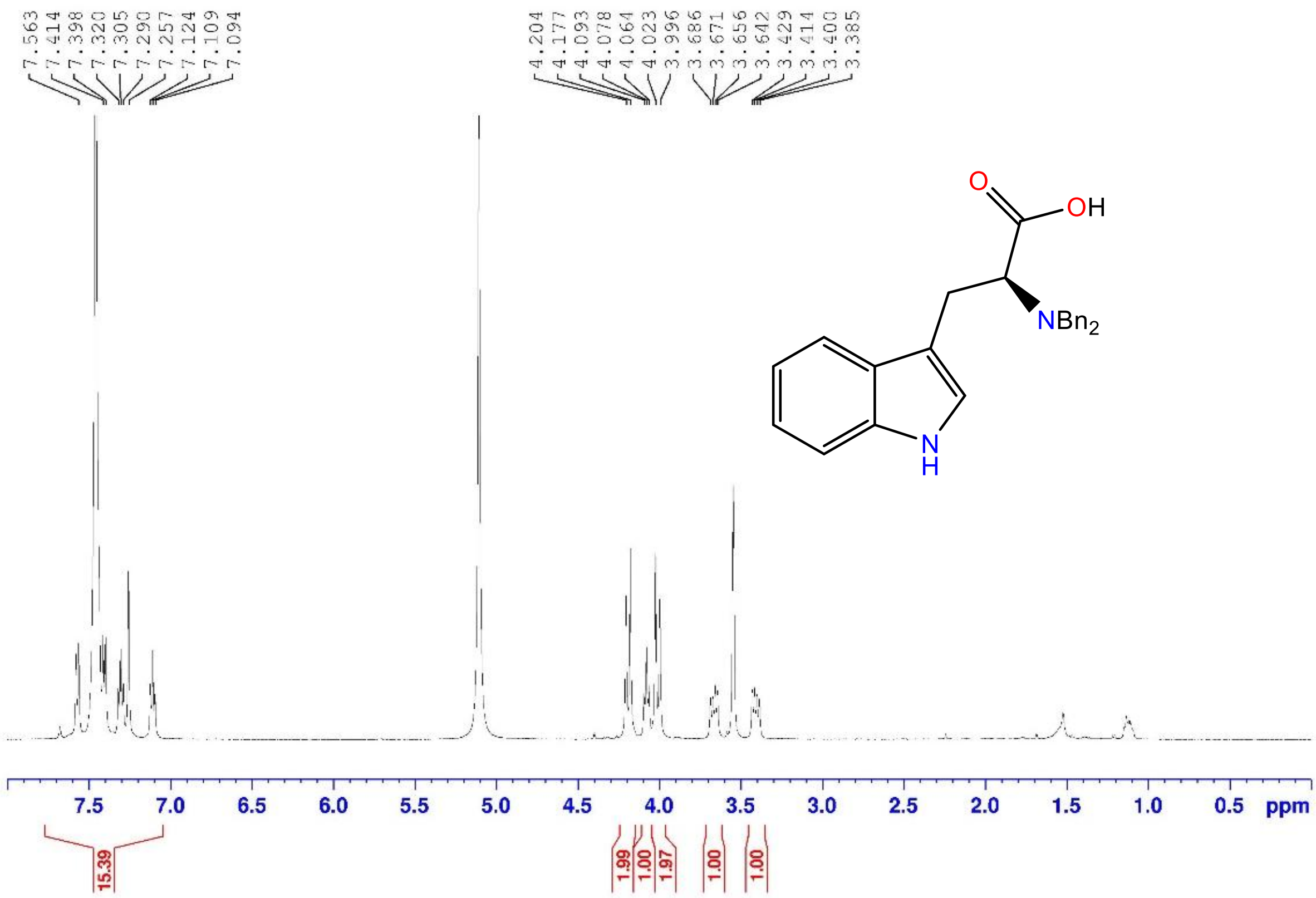

Fig S4 ^{1}H-NMR spectrum of N,N-dibenzyl-L-tryptophan (**3**)

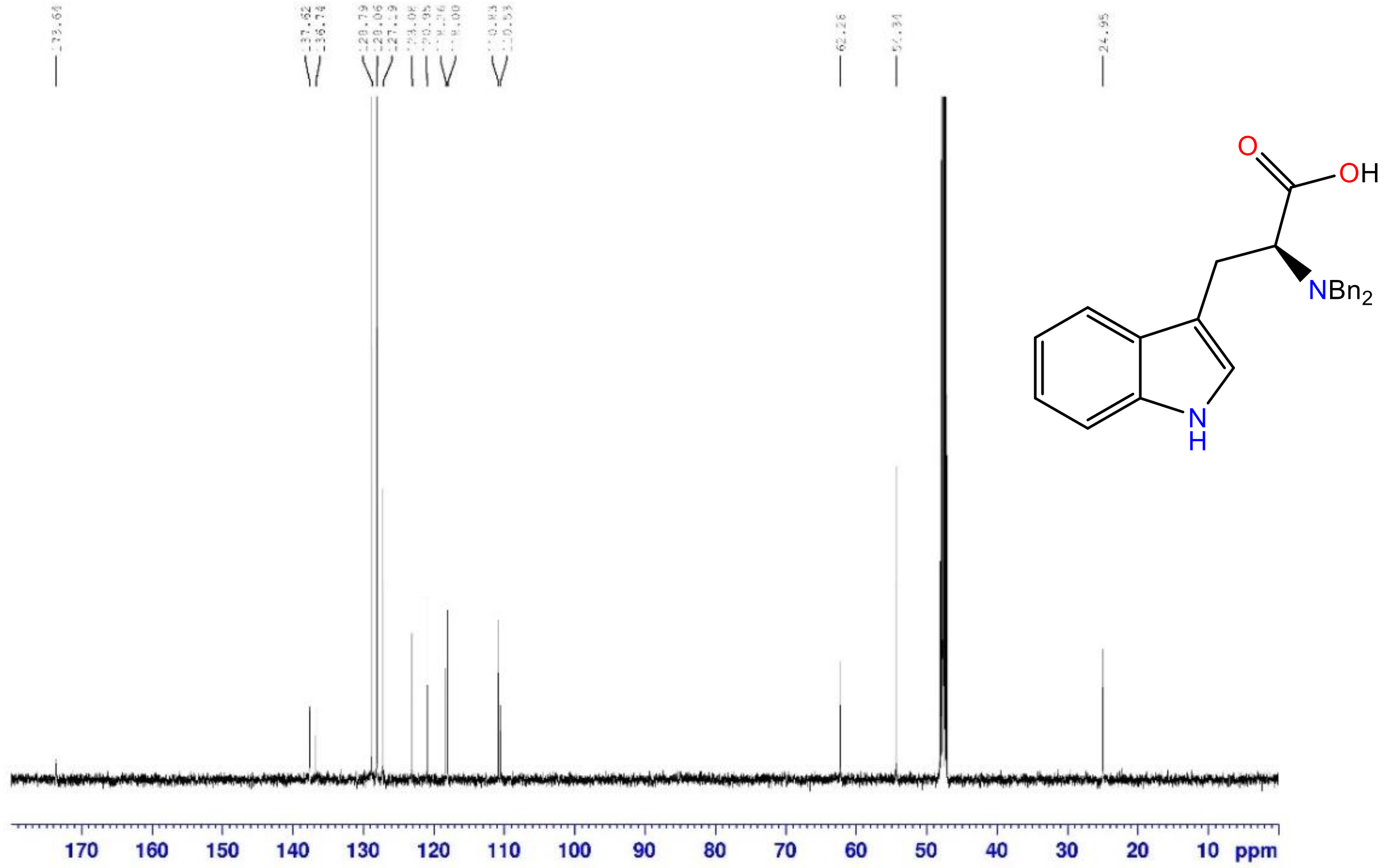

Fig S5 ^{13}C-NMR spectrum of *N*,*N*-dibenzyl-L-tryptophan (**3**)

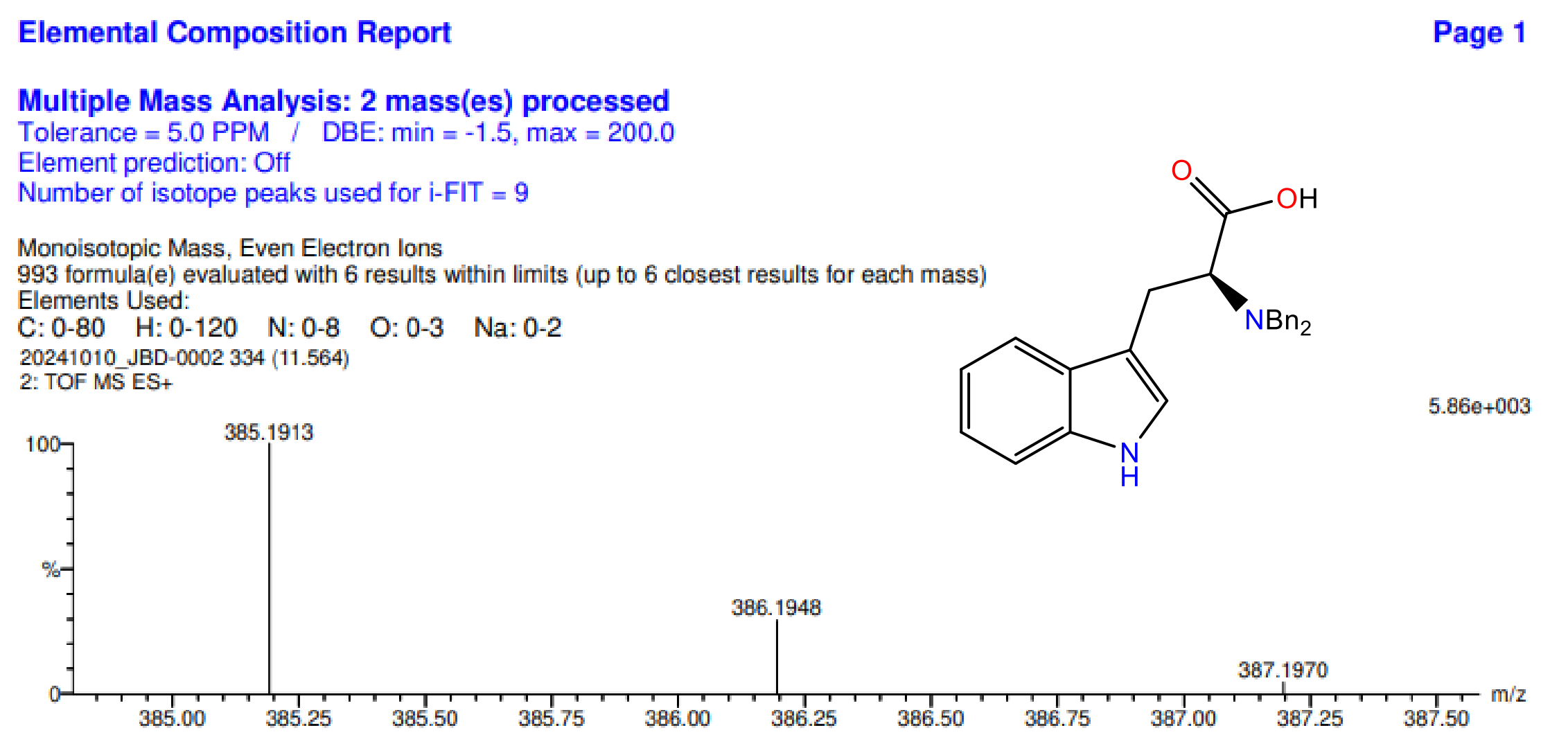

Minimum:	20.00				-1.5			
Maximum:	100.00		5.0	5.0	200.0			

Mass	RA	Calc. Mass	mDa	PPM	DBE	i-FIT	i-FIT (Norm)	Formula
385.1913	100.00	385.1916	-0.3	-0.8	14.5	36.1	2.2	C25 H25 N2 O2
		385.1908	0.5	1.3	12.5	34.1	0.1	C26 H27 Na2
		385.1932	-1.9	-4.9	15.5	38.7	4.7	C28 H26 Na
386.1948	29.44	386.1941	0.7	1.8	10.5	41.8	0.8	C18 H24 N7 O3
		386.1957	-0.9	-2.3	11.5	42.5	1.5	C21 H25 N5 O Na
		386.1933	1.5	3.9	8.5	42.2	1.2	C19 H26 N5 O Na2

Fig S6 HRMS spectrum of *N*,*N*-dibenzyl-L-tryptophan (**3**)

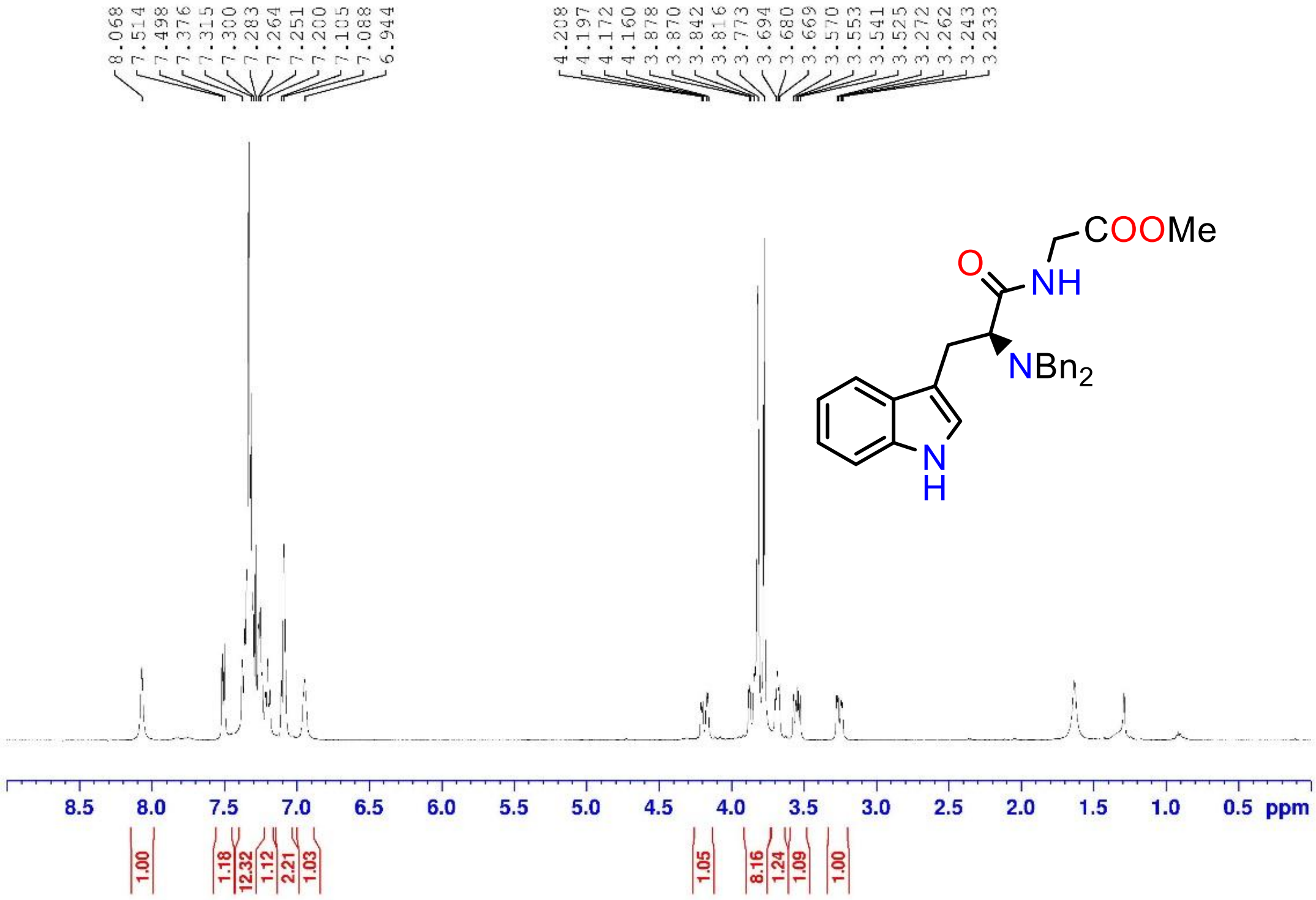

Fig S7 ^{1}H spectrum of methyl *N*,*N*-dibenzyl-L-tryptophyl-glycinate (**4a**)

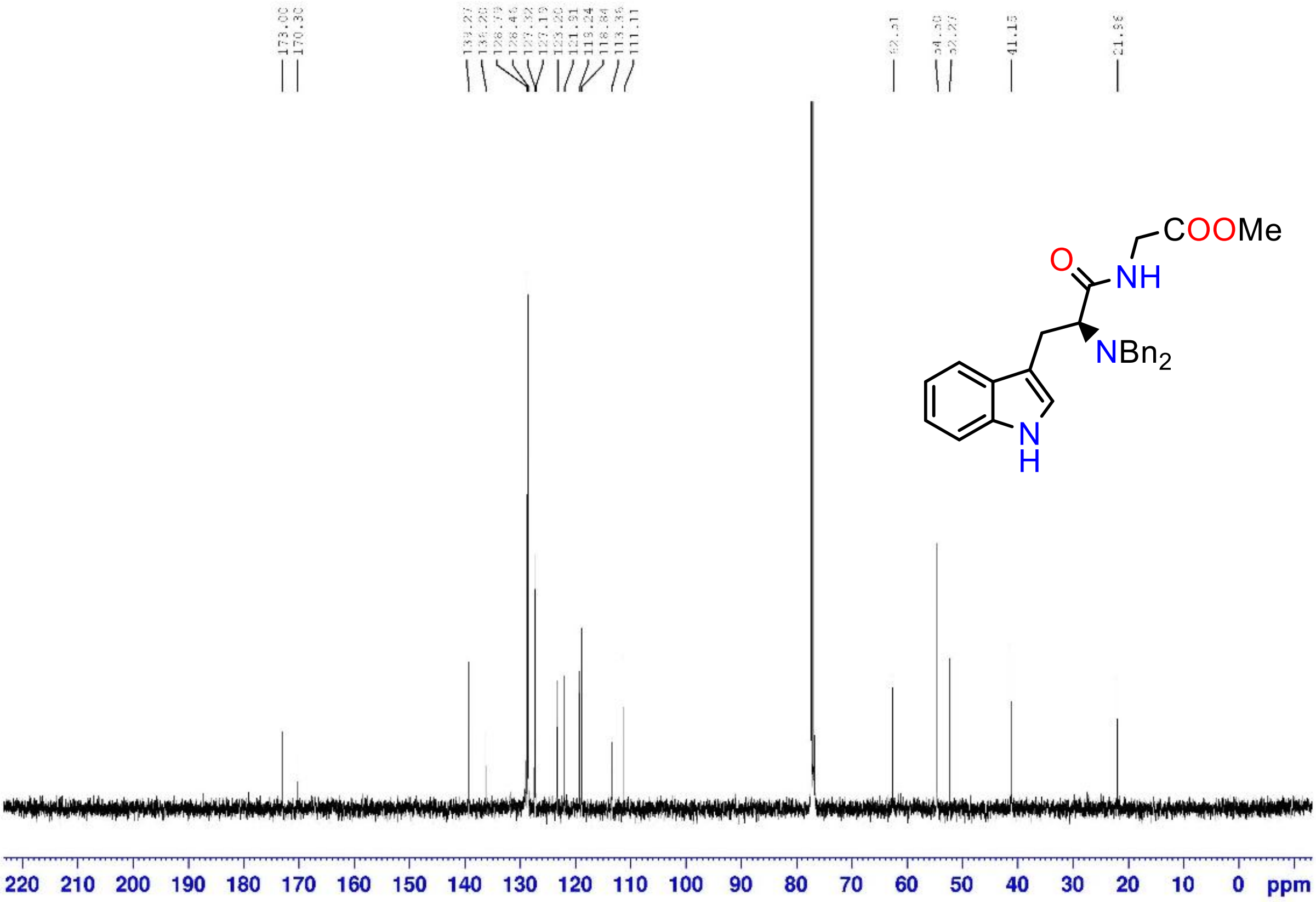

Fig S8 ^{13}C spectrum of methyl *N*,*N*-dibenzyl-L-tryptophyl-glycinate (**4a**)

Elemental Composition Report **Page 1**

Multiple Mass Analysis: 4 mass(es) processed
Tolerance = 5.0 PPM / DBE: min = -1.5, max = 200.0
Element prediction: Off
Number of isotope peaks used for i-FIT = 9

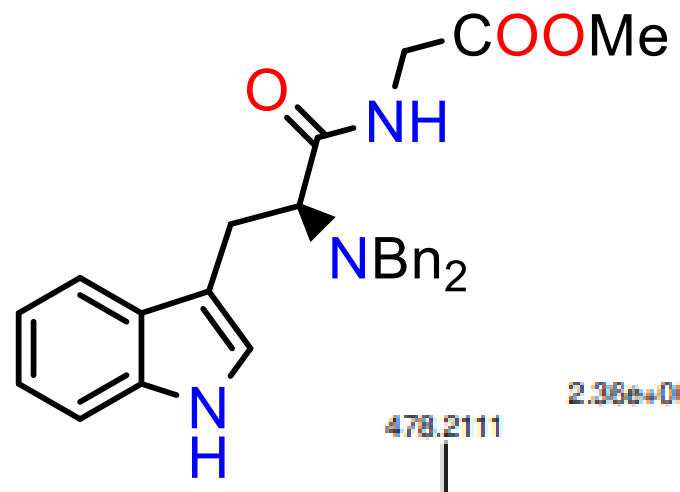

Monoisotopic Mass, Even Electron Ions
1946 formula(e) evaluated with 13 results within limits (up to 6 closest results for each mass)
Elements Used:
C: 0-60 H: 0-120 N: 3-5 O: 0-10 Na: 0-2
20241104 JBD 003 27 (0.889)
2: TOF MS ES+

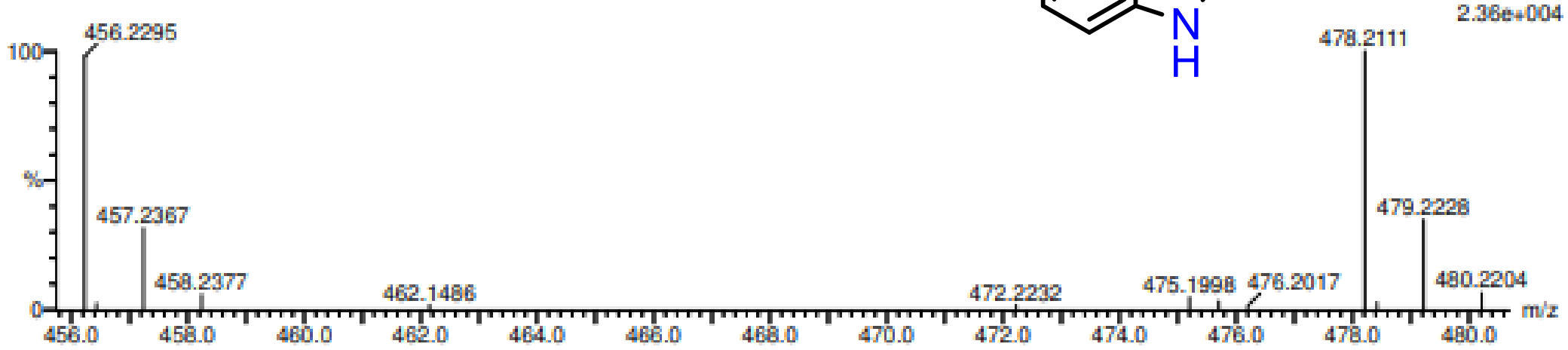

Mass	RA	Calc. Mass	mDa	PPM	DBE	i-FIT	i-FIT (Norm)	Formula
Minimum:	20.00				-1.5			
Maximum:	100.00		5.0	5.0	200.0			
456.2295	97.93	456.2298	-0.3	-0.7	0.5	80.9	0.9	C17 H36 N3 O8 Na2
		456.2287	0.8	1.8	15.5	83.0	3.0	C28 H30 N3 O3
		456.2306	-1.1	-2.4	2.5	81.8	1.8	C16 H34 N5 O10
		456.2282	1.3	2.8	-0.5	81.0	1.0	C14 H35 N5 O10 Na
457.2367	31.47	457.2368	-0.1	-0.2	16.5	67.3	0.0	C29 H30 N4 Na
478.2111	100.00	478.2107	0.4	0.8	15.5	75.6	2.3	C28 H29 N3 O3 Na
		478.2101	1.0	2.1	-0.5	74.0	0.7	C14 H34 N5 O10 Na2
		478.2125	-1.4	-2.9	2.5	74.5	1.2	C16 H33 N5 O10 Na
		478.2131	-2.0	-4.2	18.5	76.1	2.9	C30 H28 N3 O3
		478.2090	2.1	4.4	14.5	75.9	2.6	C25 H28 N5 O5
479.2228	34.79	479.2236	-0.8	-1.7	22.5	58.7	1.2	C33 H27 N4
		479.2212	1.6	3.3	19.5	58.5	1.0	C31 H28 N4 Na
		479.2246	-1.8	-3.8	7.5	58.5	1.1	C22 H33 N4 O5 Na2

Fig S9 HRMS spectrum of methyl *N*,*N*-dibenzyl-L-tryptophyl-glycinate (**4a**)

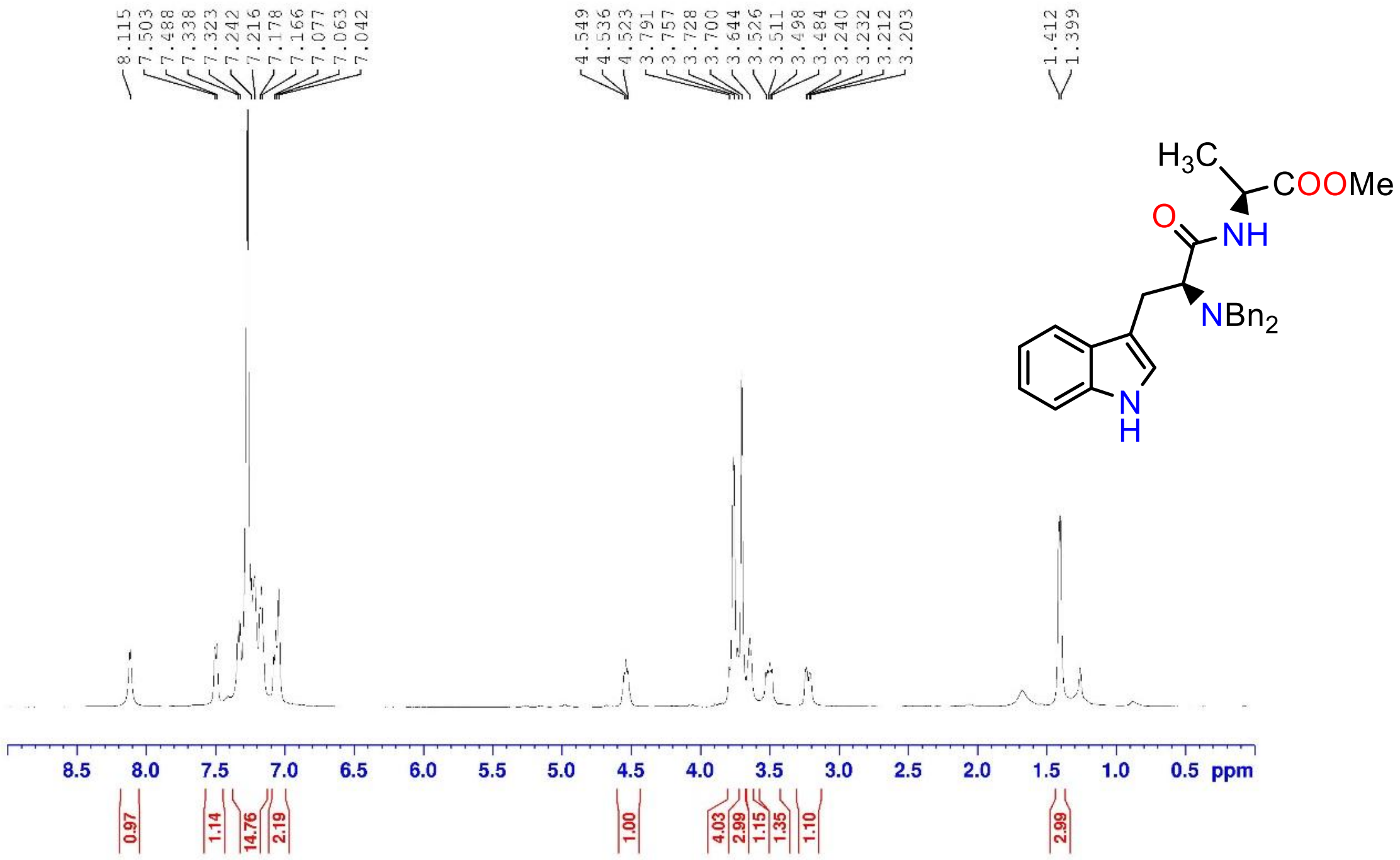

Fig S10 ^{1}H spectrum of methyl *N*,*N*-dibenzyl-L-tryptophyl-L-alaninate (**4b**)

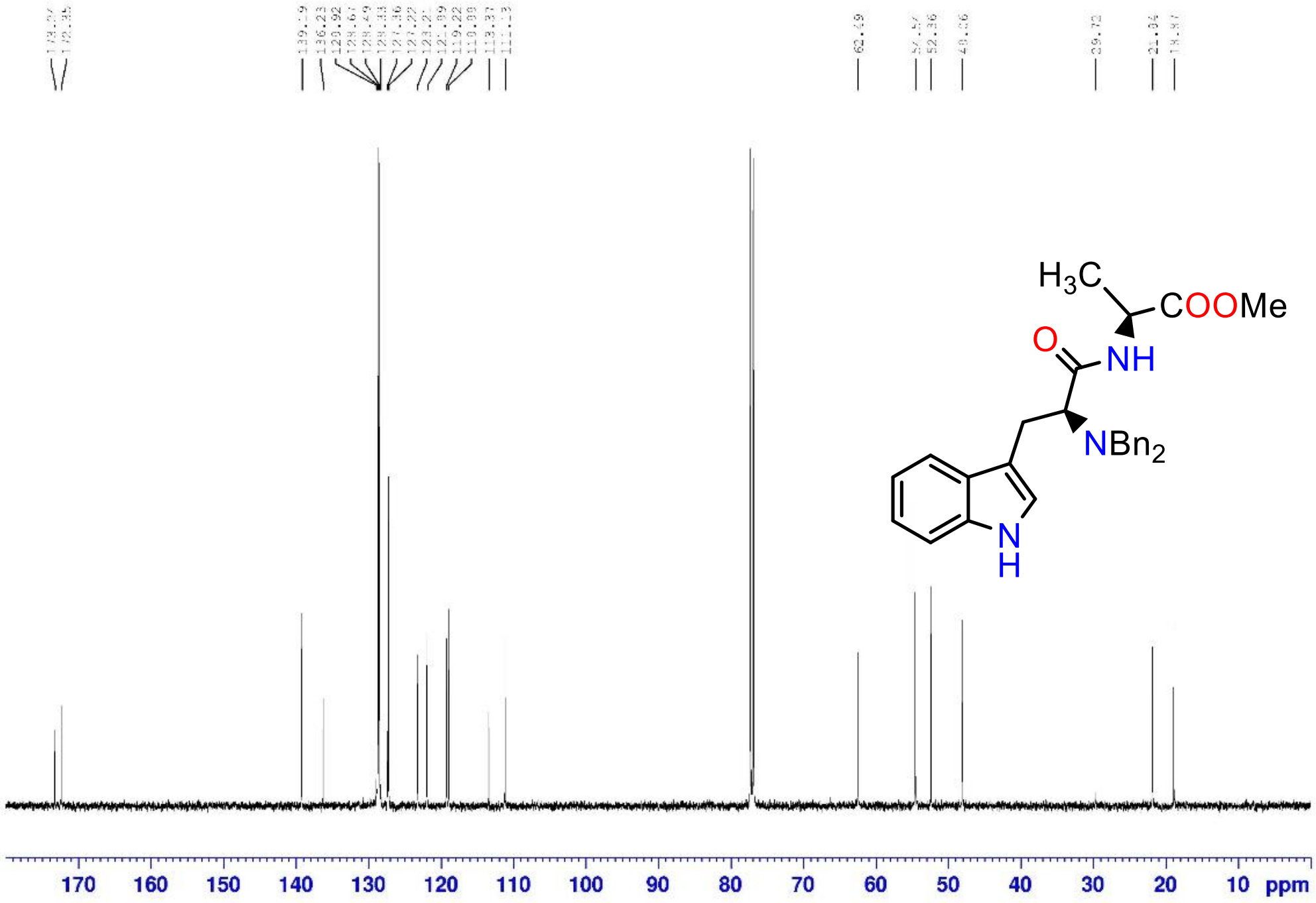

Fig S11 ^{13}C spectrum of methyl *N,N*-dibenzyl-L-tryptophyl-L-alaninate (**4b**)

Elemental Composition Report **Page 1**

Multiple Mass Analysis: 2 mass(es) processed
Tolerance = 5.0 PPM / DBE: min = -1.5, max = 200.0
Element prediction: Off
Number of isotope peaks used for i-FIT = 9

Monoisotopic Mass, Even Electron Ions
5565 formula(e) evaluated with 26 results within limits (up to 6 closest results for each mass)
Elements Used:
C: 0-60 H: 0-120 N: 0-5 O: 0-10 Na: 0-2 S: 0-2
20241104_JBD 004 24 (1.009)
1: TOF MS ES+

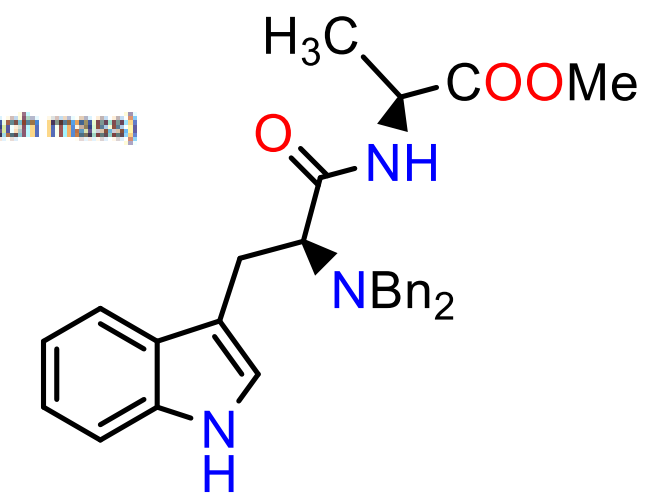

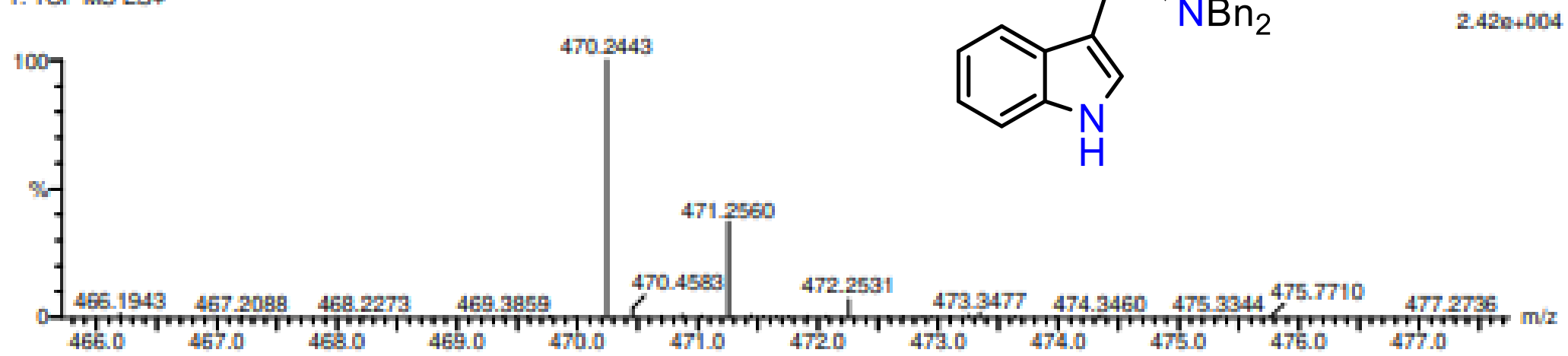

Minimum:	20.00				-1.5			
Maximum:	100.00		5.0	5.0	200.0			
Mass	RA	Calc. Mass	mDa	PPM	DBE	i-FIT	i-FIT (Norm)	Formula
470.2443	100.00	470.2444	-0.1	-0.2	15.5	222.3	0.6	C29 H32 N3 O3
		470.2447	-0.4	-0.9	-1.5	249.9	28.2	C16 H41 N5 O5 Na S2
		470.2438	0.5	1.1	-0.5	234.0	12.2	C15 H37 N5 O10 Na
		470.2437	0.6	1.3	6.5	231.3	9.5	C21 H36 N5 O5 S
		470.2436	0.7	1.5	13.5	222.6	0.8	C30 H34 N O Na2
		470.2453	-1.0	-2.1	7.5	230.4	8.6	C24 H37 N3 O3 Na S
471.2560	36.96	471.2559	0.1	0.2	4.5	181.4	0.0	C21 H37 N4 O5 Na2
		471.2558	0.2	0.4	11.5	188.4	7.0	C27 H36 N4 Na S
		471.2563	-0.3	-0.6	0.5	190.1	8.7	C20 H43 N2 O6 S2
		471.2555	0.5	1.1	-1.5	189.8	8.4	C21 H45 O4 Na2 S2
		471.2568	-0.8	-1.7	3.5	189.9	8.5	C22 H41 N4 Na2 S2
		471.2569	-0.9	-1.9	9.5	188.8	7.4	C28 H39 O4 S

Fig S12 HRMS spectrum of methyl *N,N*-dibenzyl-L-tryptophyl-L-alaninate (**4b**)

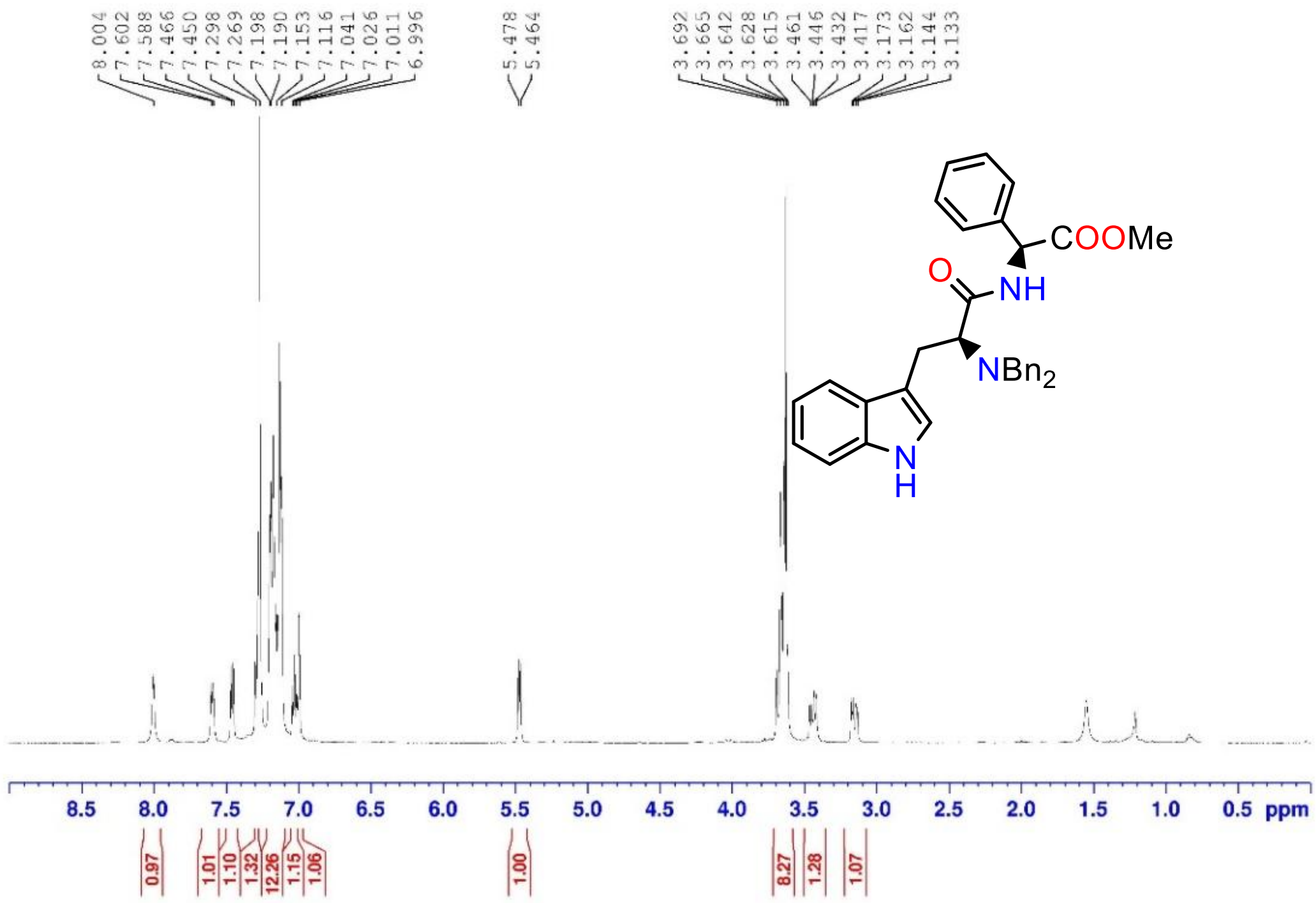

Fig S13 ^{1}H spectrum of methyl *N*,*N*-dibenzyl-L-tryptophyl-L-phenylglycinate (**4c**)

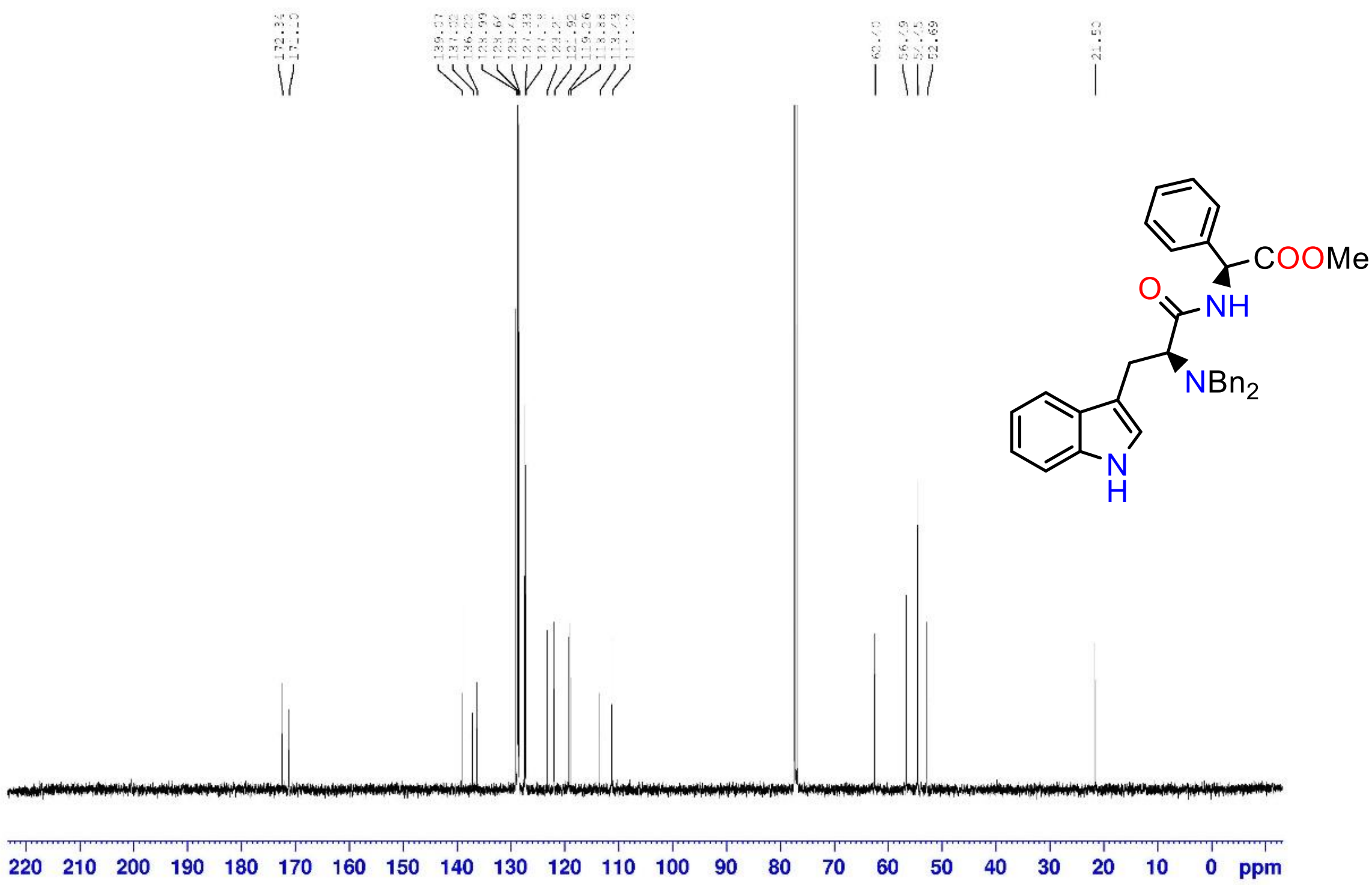

Fig S14 ^{13}C spectrum of methyl *N*,*N*-dibenzyl-L-tryptophyl-L-phenylglycinate (**4c**)

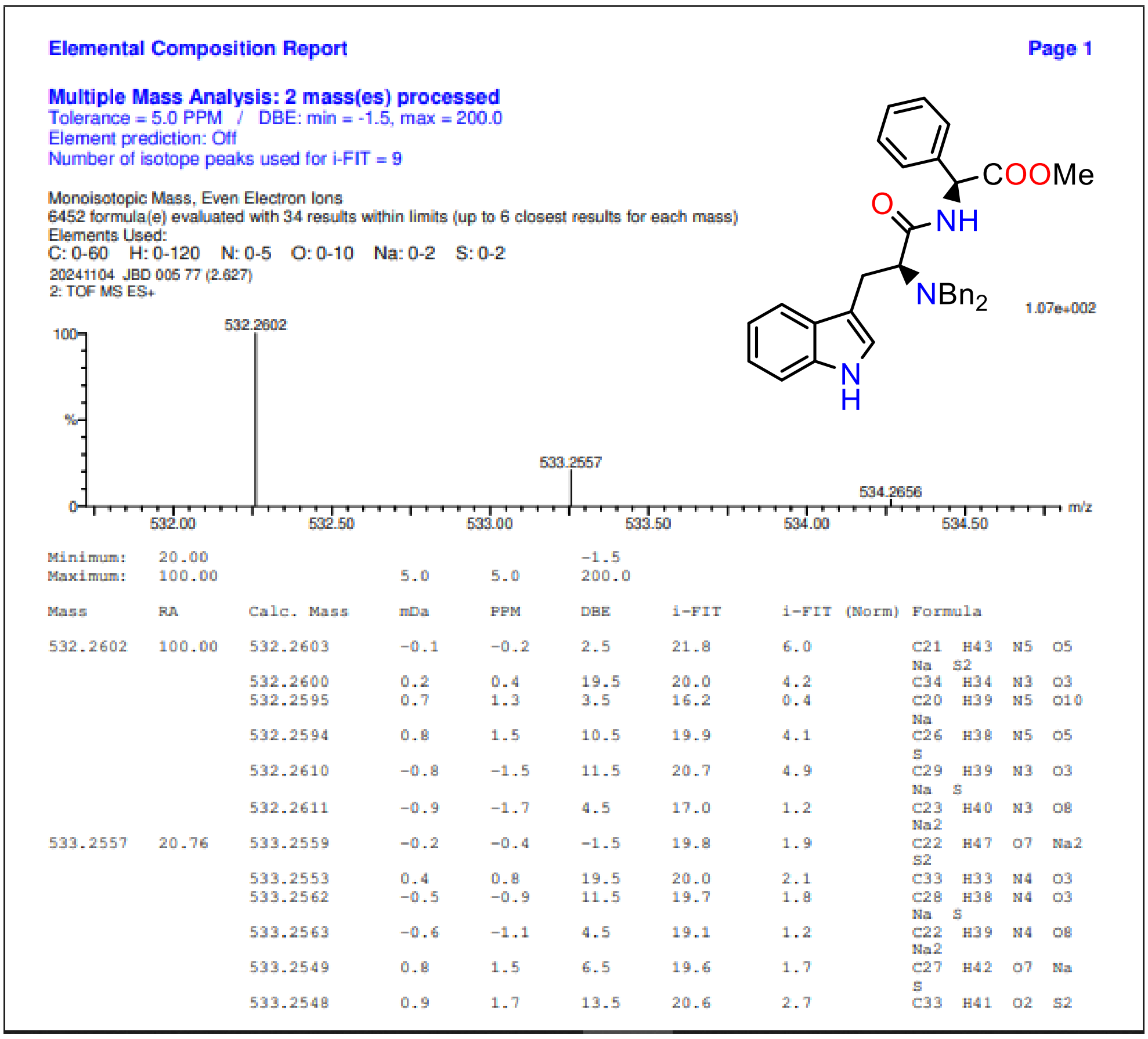

Minimum:	20.00				-1.5			
Maximum:	100.00		5.0	5.0	200.0			

Mass	RA	Calc. Mass	mDa	PPM	DBE	i-FIT	i-FIT (Norm)	Formula
532.2602	100.00	532.2603	-0.1	-0.2	2.5	21.8	6.0	C21 H43 N5 O5 Na S2
		532.2600	0.2	0.4	19.5	20.0	4.2	C34 H34 N3 O3
		532.2595	0.7	1.3	3.5	16.2	0.4	C20 H39 N5 O10 Na
		532.2594	0.8	1.5	10.5	19.9	4.1	C26 H38 N5 O5 S
		532.2610	-0.8	-1.5	11.5	20.7	4.9	C29 H39 N3 O3 Na S
		532.2611	-0.9	-1.7	4.5	17.0	1.2	C23 H40 N3 O8 Na2
533.2557	20.76	533.2559	-0.2	-0.4	-1.5	19.8	1.9	C22 H47 O7 Na2 S2
		533.2553	0.4	0.8	19.5	20.0	2.1	C33 H33 N4 O3
		533.2562	-0.5	-0.9	11.5	19.7	1.8	C28 H38 N4 O3 Na S
		533.2563	-0.6	-1.1	4.5	19.1	1.2	C22 H39 N4 O8 Na2
		533.2549	0.8	1.5	6.5	19.6	1.7	C27 H42 O7 Na S
		533.2548	0.9	1.7	13.5	20.6	2.7	C33 H41 O2 S2

Fig S15 HRMS spectrum of methyl *N*,*N*-dibenzyl-L-tryptophyl-L-phenylglycinate (**4c**)

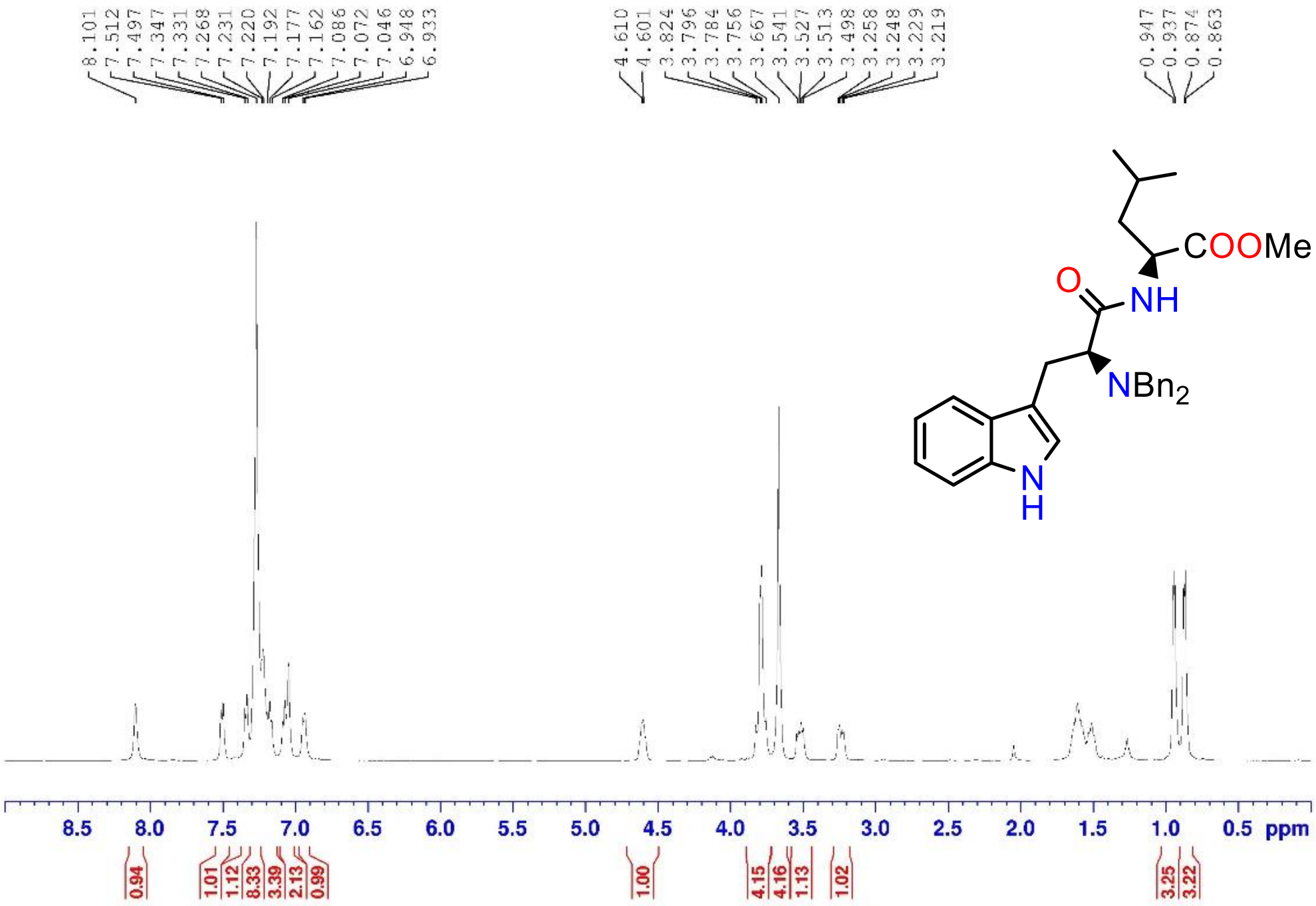

Fig S16 ^{1}H spectrum of methyl *N*,*N*-dibenzyl-L-tryptophyl-L-leucinate (**4d**)

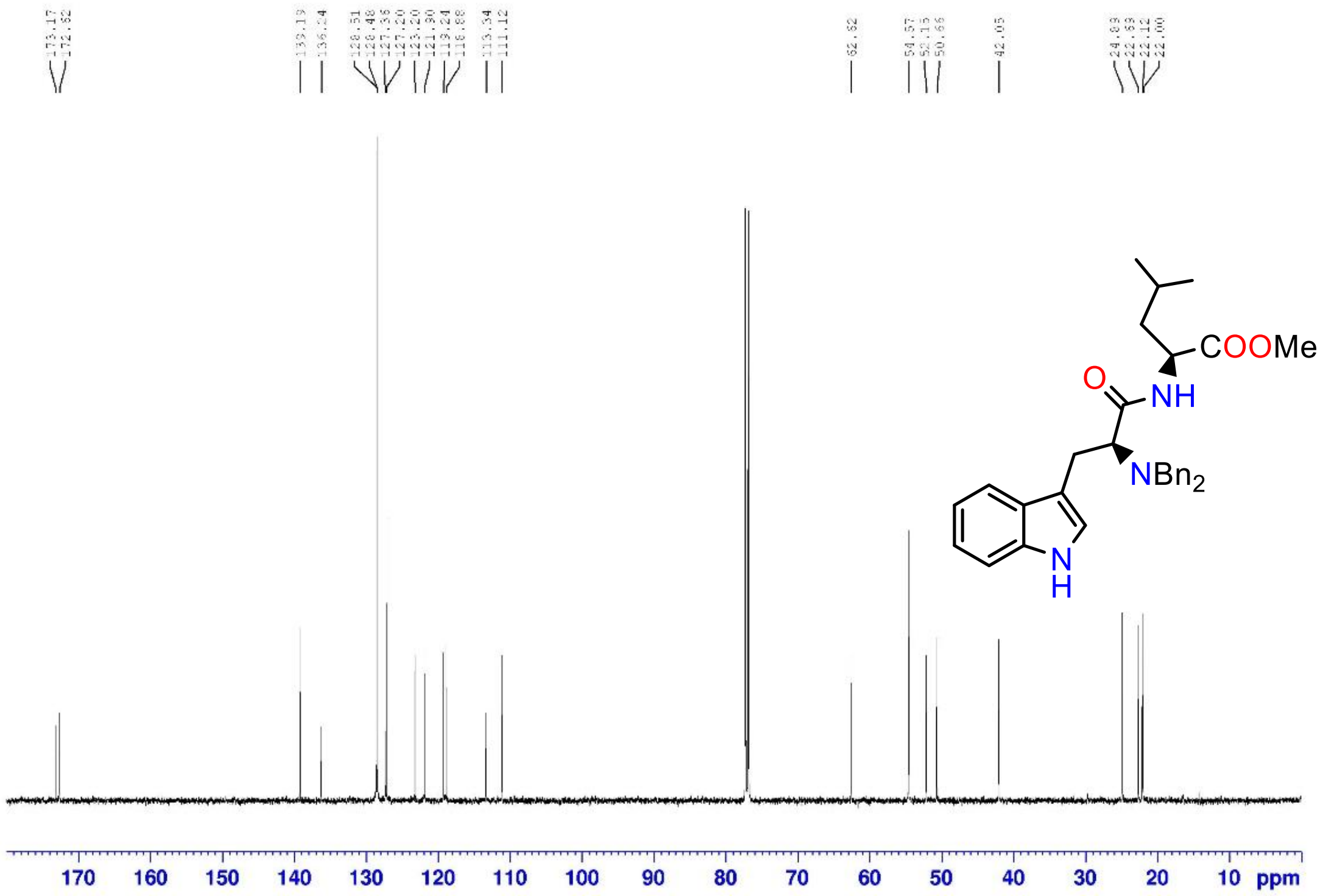

Fig S17 ^{13}C spectrum of methyl *N*,*N*-dibenzyl-L-tryptophyl-L-leucinate (**4d**)

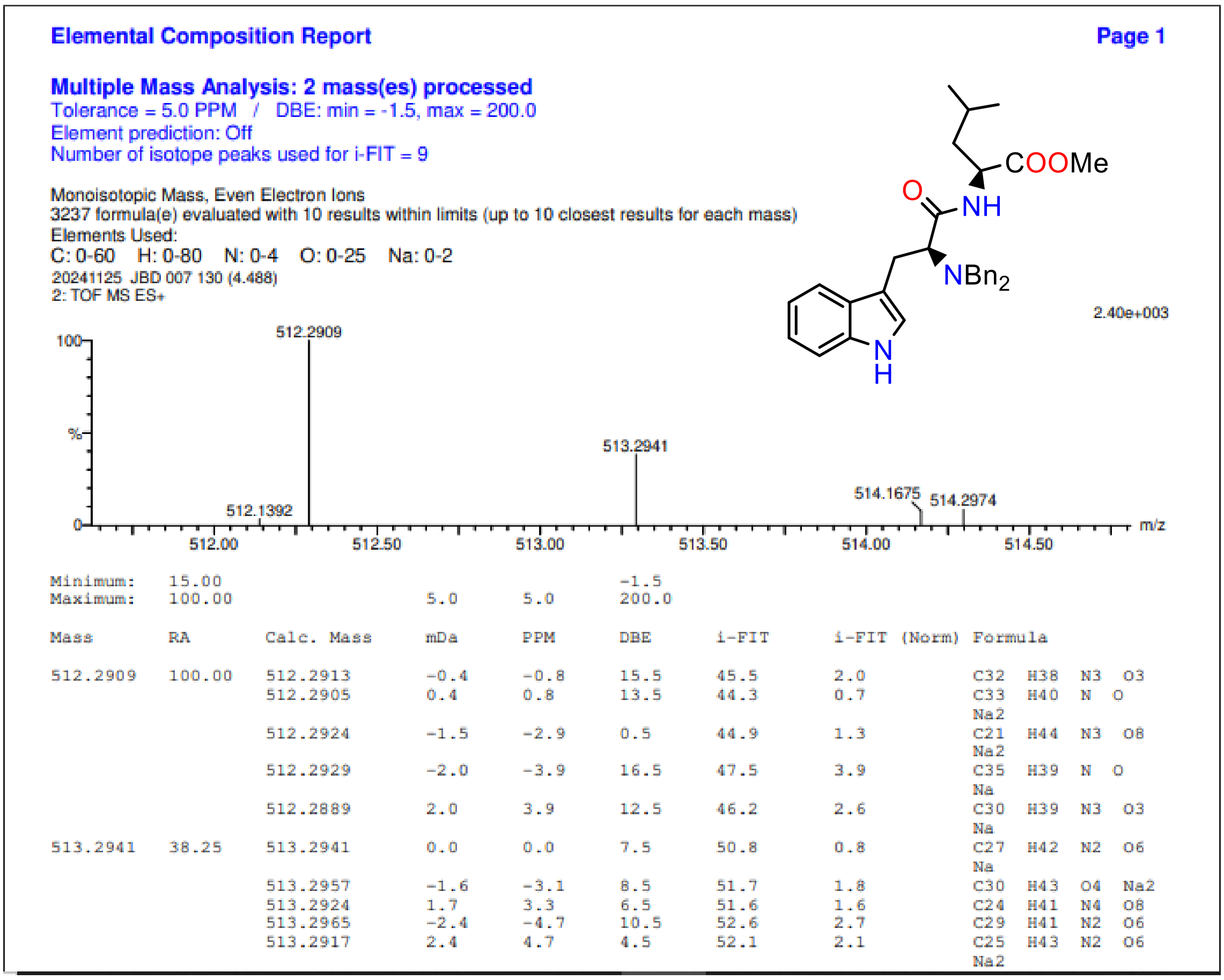

Elemental Composition Report **Page 1**

Multiple Mass Analysis: 2 mass(es) processed
Tolerance = 5.0 PPM / DBE: min = -1.5, max = 200.0
Element prediction: Off
Number of isotope peaks used for i-FIT = 9

Monoisotopic Mass, Even Electron Ions
3237 formula(e) evaluated with 10 results within limits (up to 10 closest results for each mass)
Elements Used:
C: 0-60 H: 0-80 N: 0-4 O: 0-25 Na: 0-2
20241125 JBD 007 130 (4.488)
2: TOF MS ES+

Minimum:	15.00				-1.5			
Maximum:	100.00		5.0	5.0	200.0			
Mass	RA	Calc. Mass	mDa	PPM	DBE	i-FIT	i-FIT (Norm)	Formula
512.2909	100.00	512.2913	-0.4	-0.8	15.5	45.5	2.0	C32 H38 N3 O3
		512.2905	0.4	0.8	13.5	44.3	0.7	C33 H40 N O Na2
		512.2924	-1.5	-2.9	0.5	44.9	1.3	C21 H44 N3 O8 Na2
		512.2929	-2.0	-3.9	16.5	47.5	3.9	C35 H39 N O Na
		512.2889	2.0	3.9	12.5	46.2	2.6	C30 H39 N3 O3 Na
513.2941	38.25	513.2941	0.0	0.0	7.5	50.8	0.8	C27 H42 N2 O6 Na
		513.2957	-1.6	-3.1	8.5	51.7	1.8	C30 H43 O4 Na2
		513.2924	1.7	3.3	6.5	51.6	1.6	C24 H41 N4 O8
		513.2965	-2.4	-4.7	10.5	52.6	2.7	C29 H41 N2 O6
		513.2917	2.4	4.7	4.5	52.1	2.1	C25 H43 N2 O6 Na2

Fig S18 HRMS spectrum of methyl *N*,*N*-dibenzyl-L-tryptophyl-L-leucinate (**4d**)

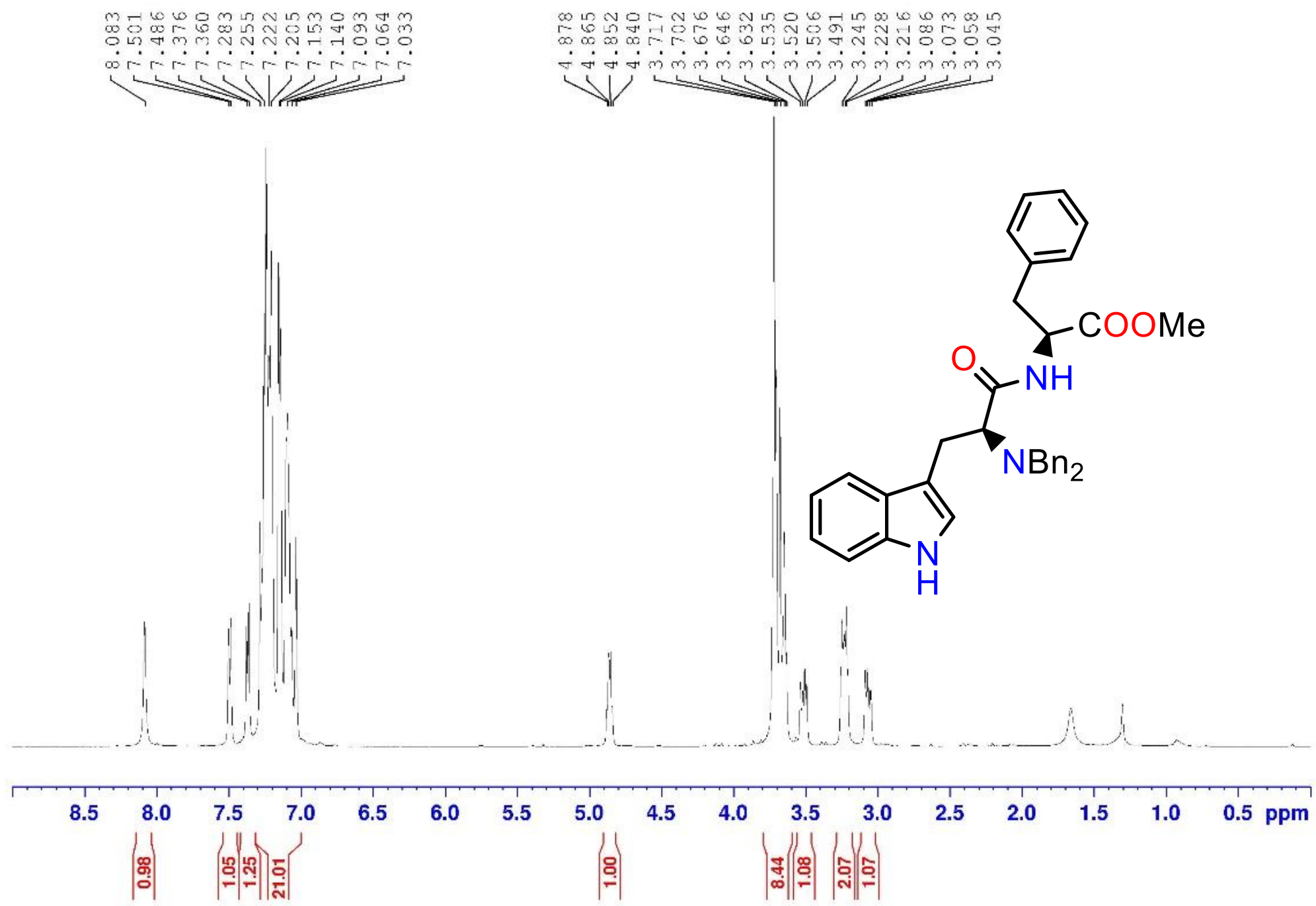

Fig S19 ^{1}H spectrum of methyl *N*,*N*-dibenzyl-L-tryptophyl-L-phenylalaninate (**4e**)

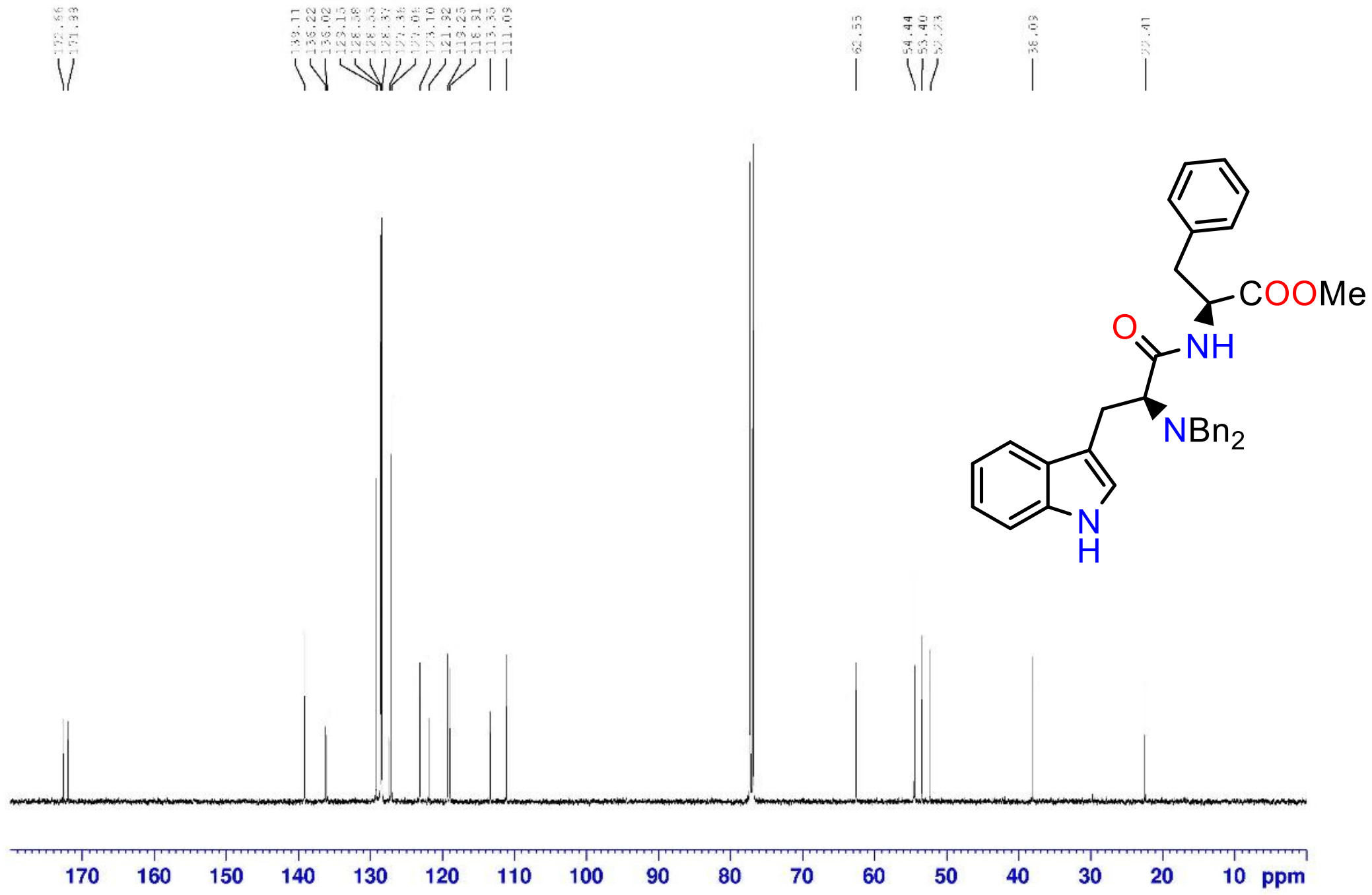

Fig S20 ^{13}C spectrum of methyl *N*,*N*-dibenzyl-L-tryptophyl-L-phenylalaninate (**4e**)

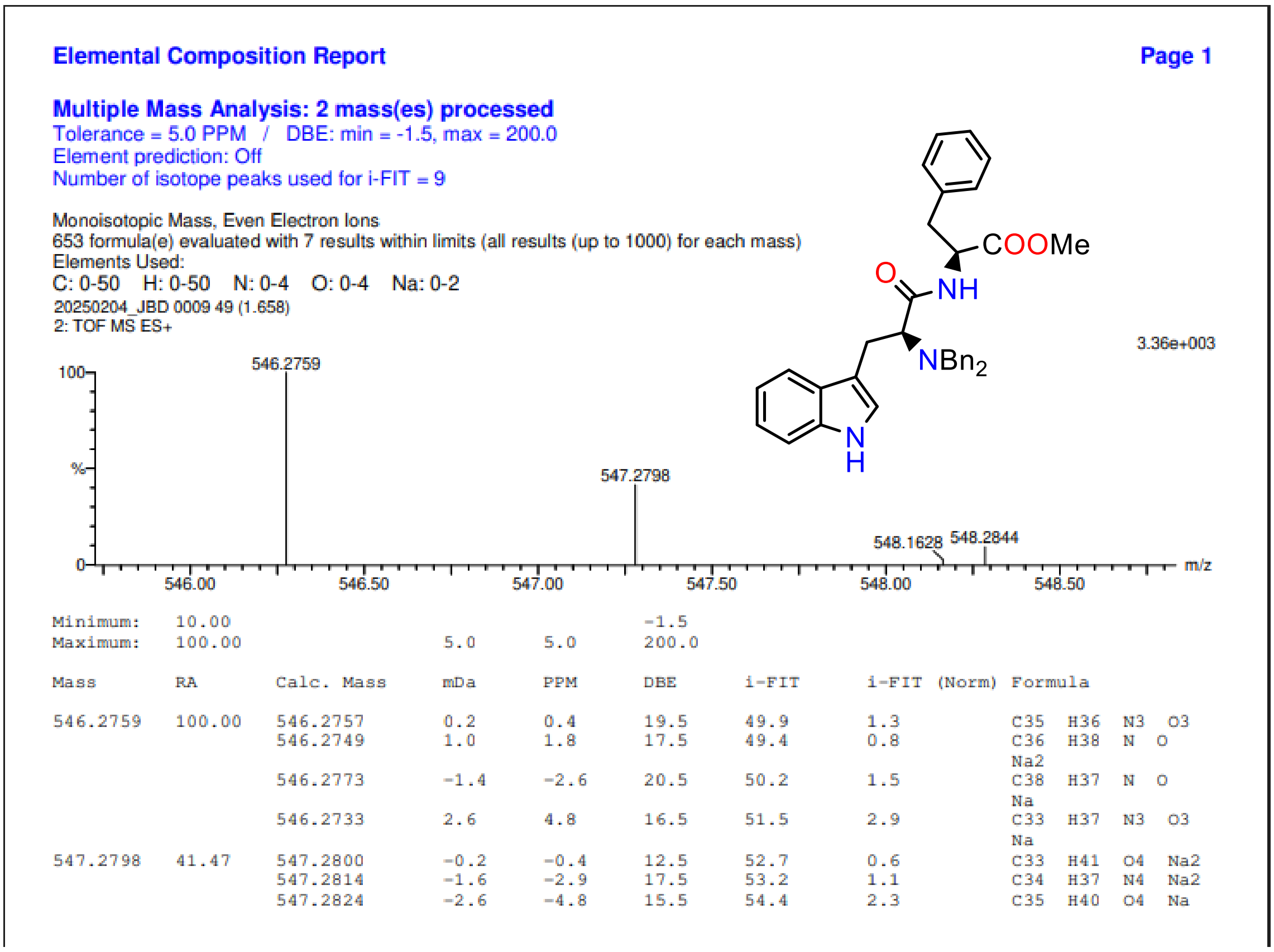

Elemental Composition Report Page 1

Multiple Mass Analysis: 2 mass(es) processed
Tolerance = 5.0 PPM / DBE: min = -1.5, max = 200.0
Element prediction: Off
Number of isotope peaks used for i-FIT = 9

Monoisotopic Mass, Even Electron Ions
653 formula(e) evaluated with 7 results within limits (all results (up to 1000) for each mass)
Elements Used:
C: 0-50 H: 0-50 N: 0-4 O: 0-4 Na: 0-2
20250204_JBD 0009 49 (1.658)
2: TOF MS ES+

Minimum:	10.00				-1.5			
Maximum:	100.00		5.0	5.0	200.0			

Mass	RA	Calc. Mass	mDa	PPM	DBE	i-FIT	i-FIT (Norm)	Formula
546.2759	100.00	546.2757	0.2	0.4	19.5	49.9	1.3	C35 H36 N3 O3
		546.2749	1.0	1.8	17.5	49.4	0.8	C36 H38 N O Na2
		546.2773	-1.4	-2.6	20.5	50.2	1.5	C38 H37 N O Na
		546.2733	2.6	4.8	16.5	51.5	2.9	C33 H37 N3 O3 Na
547.2798	41.47	547.2800	-0.2	-0.4	12.5	52.7	0.6	C33 H41 O4 Na2
		547.2814	-1.6	-2.9	17.5	53.2	1.1	C34 H37 N4 Na2
		547.2824	-2.6	-4.8	15.5	54.4	2.3	C35 H40 O4 Na

Fig S21 HRMS spectrum of methyl *N*,*N*-dibenzyl-L-tryptophyl-L-phenylalaninate (**4e**)

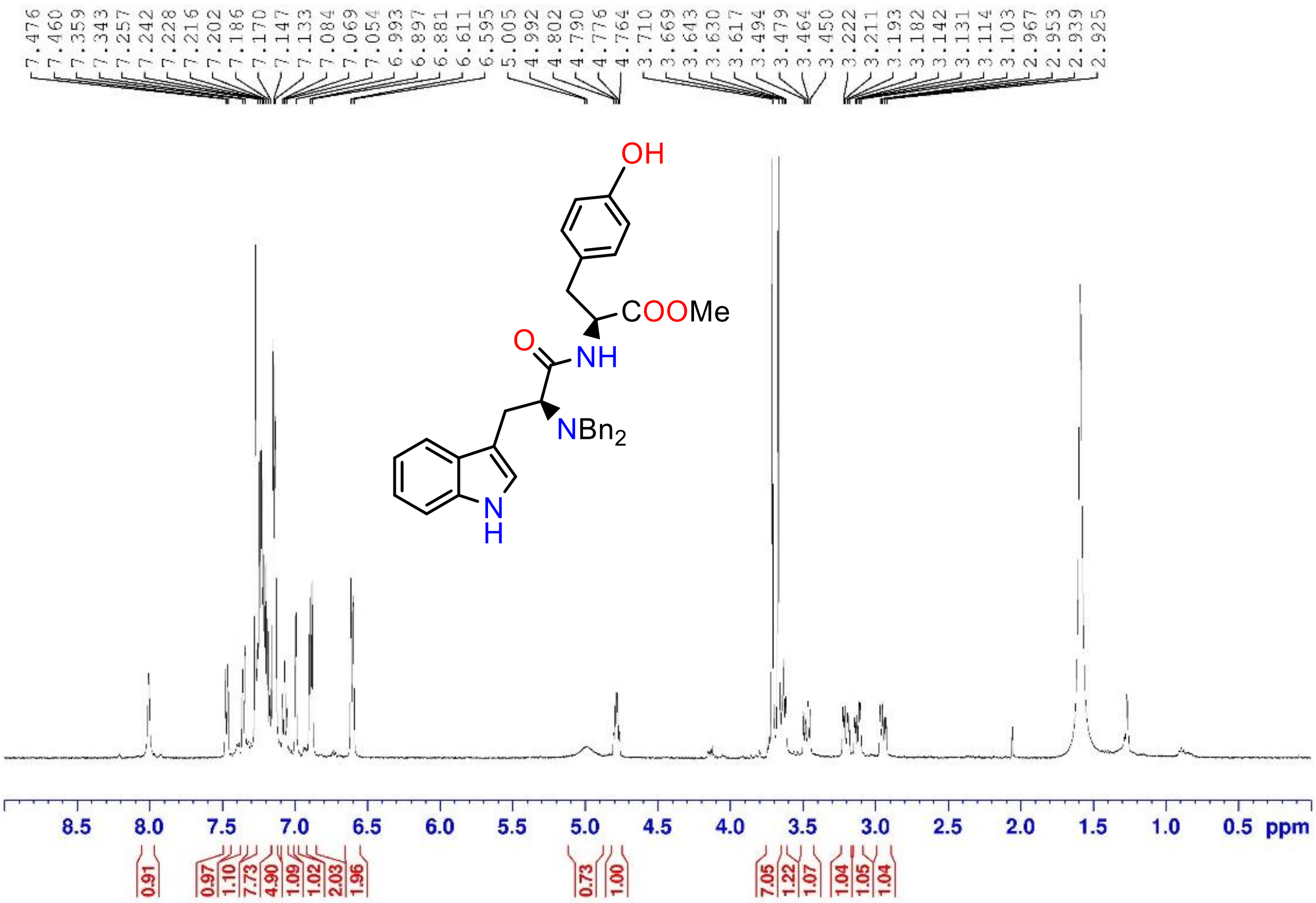

Fig S22 ^{1}H spectrum of methyl *N*,*N*-dibenzyl-L-tryptophyl-L-tyrosinate (**4f**)

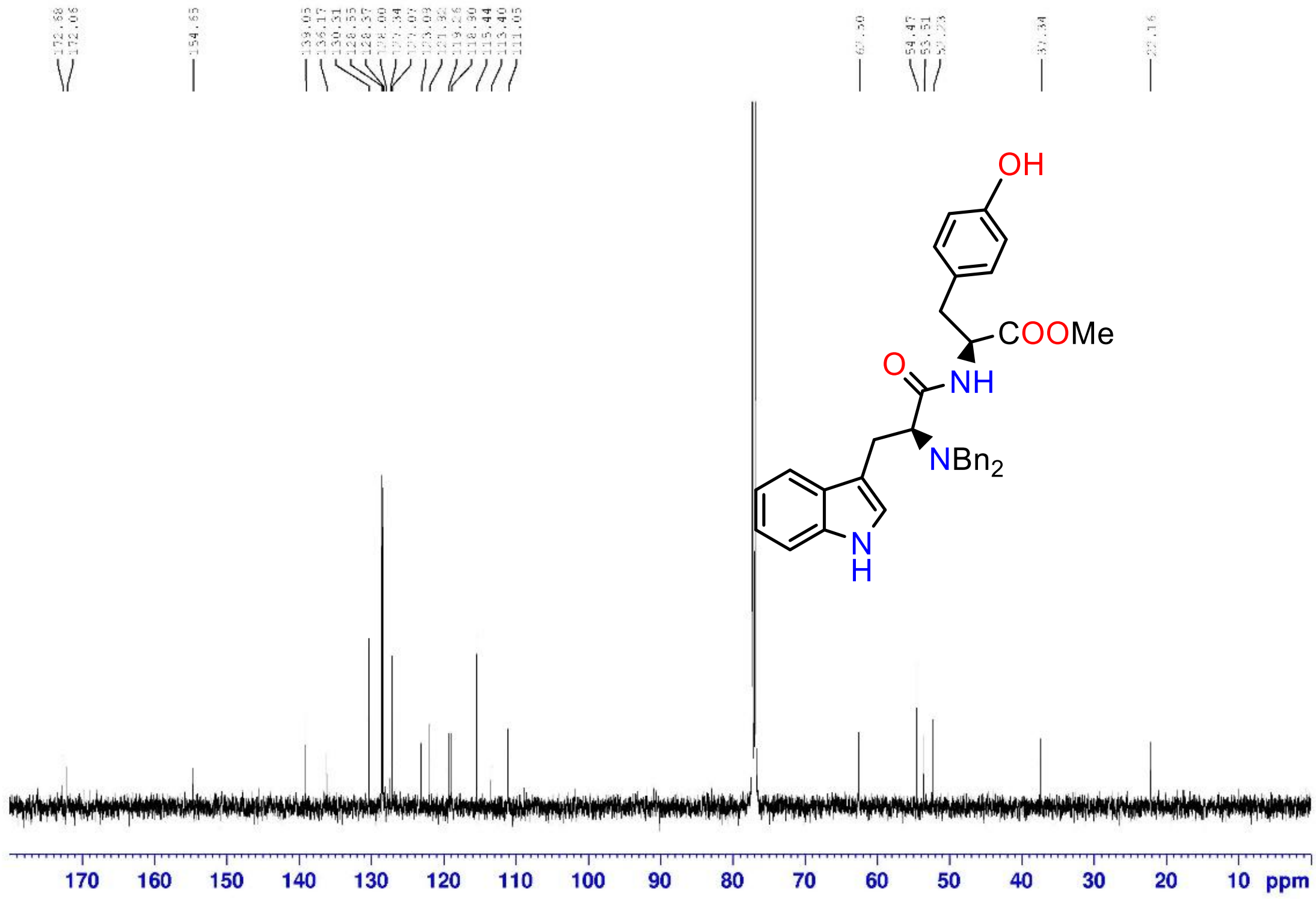

Fig S23 ^{13}C spectrum of methyl *N*,*N*-dibenzyl-L-tryptophyl-L-tyrosinate (**4f**)

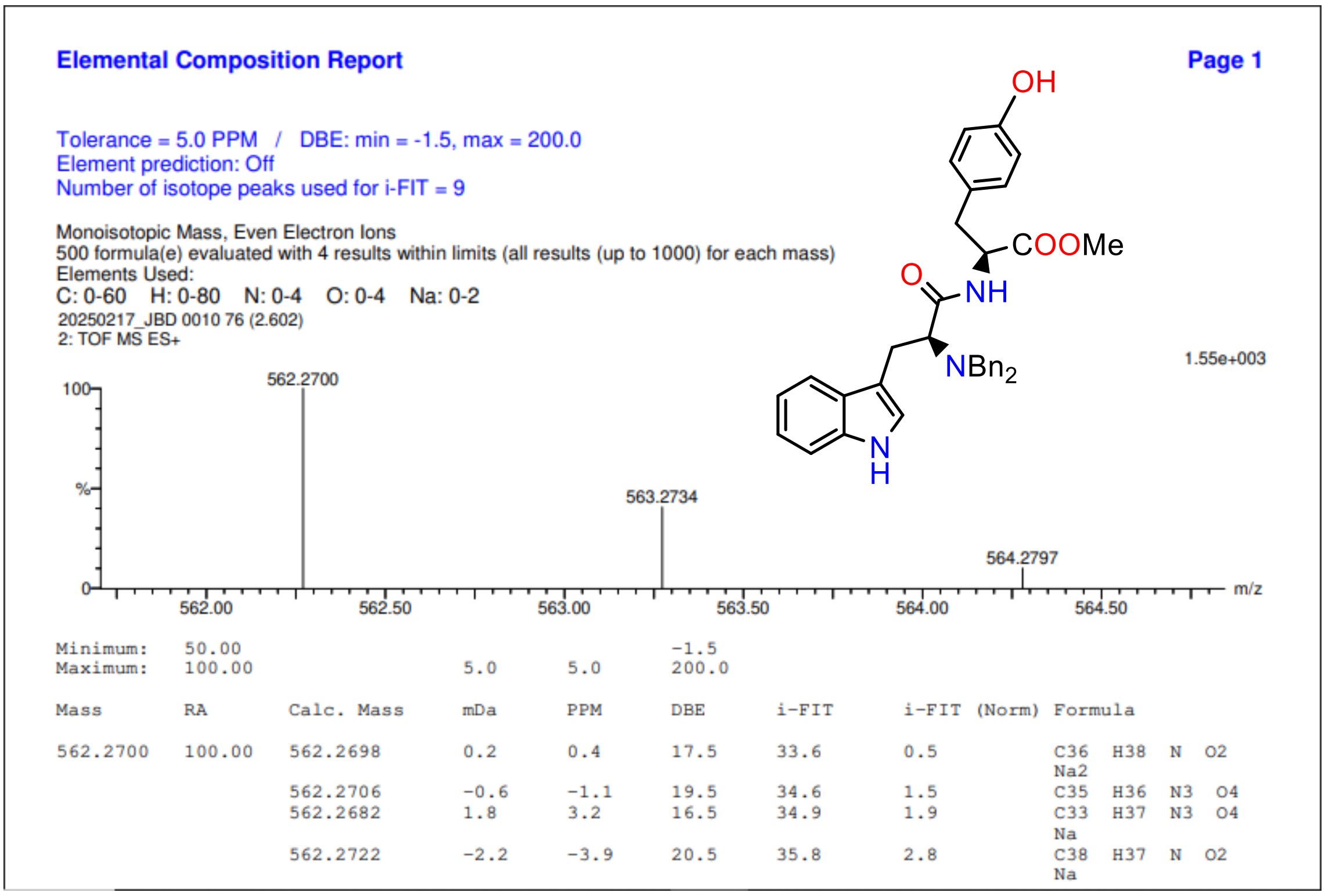

Fig S24 HRMS spectrum of methyl *N*,*N*-dibenzyl-L-tryptophyl-L-tyrosinate (**4f**)

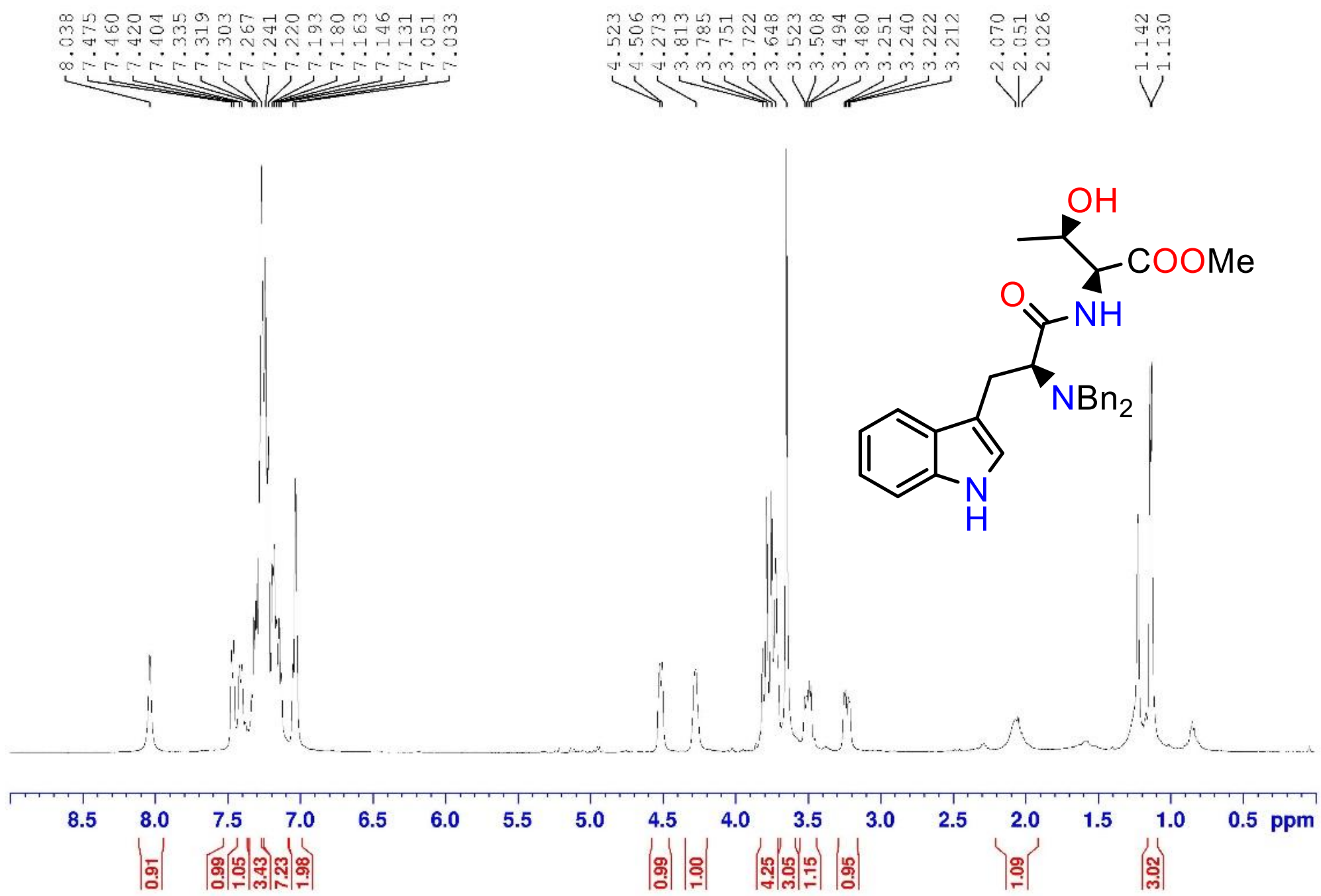

Fig S25 ^{1}H spectrum of methyl *N*,*N*-dibenzyl-L-tryptophyl-L-threoninate (**4g**)

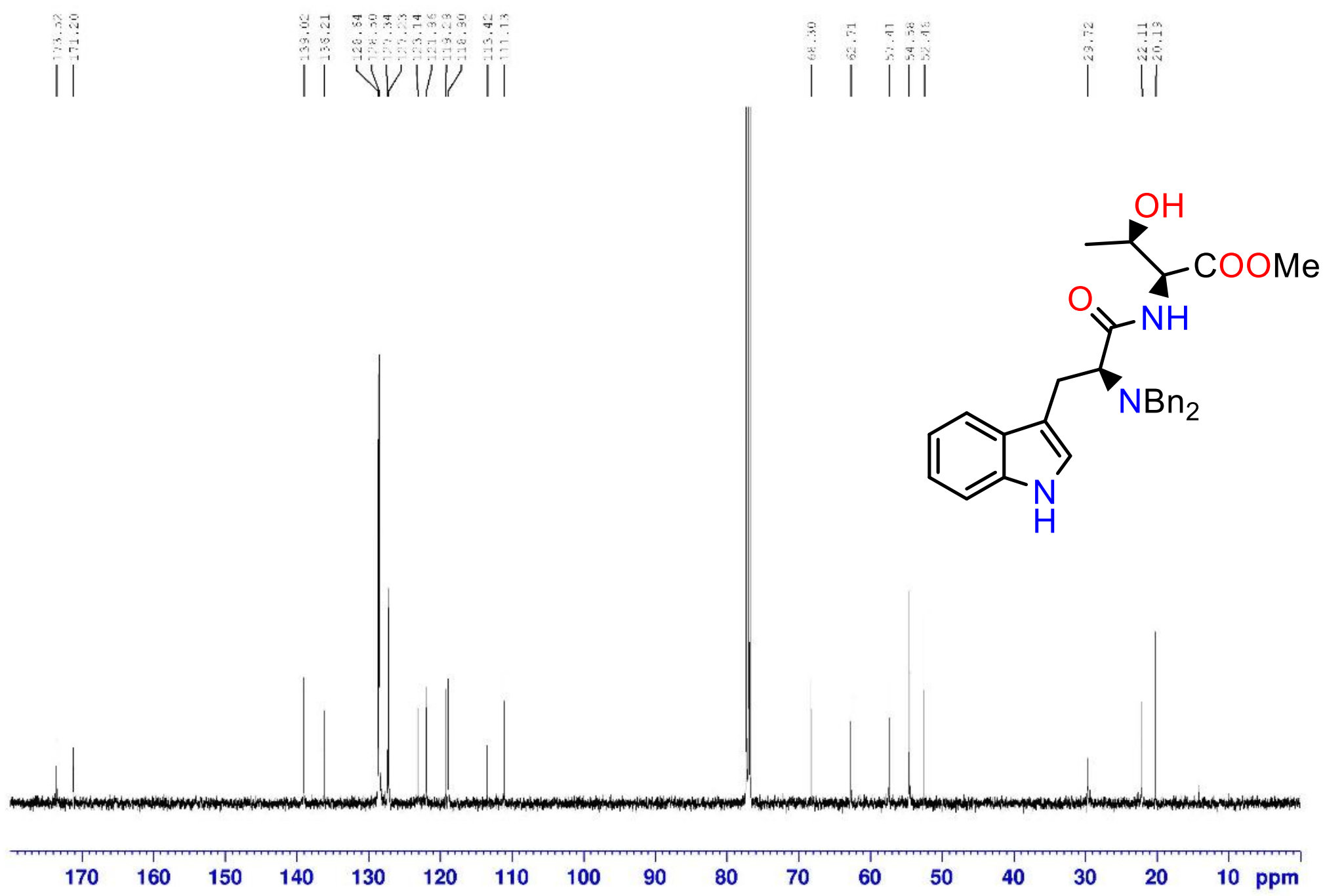

Fig S26 ^{13}C spectrum of methyl *N*,*N*-dibenzyl-L-tryptophyl-L-threoninate (**4g**)

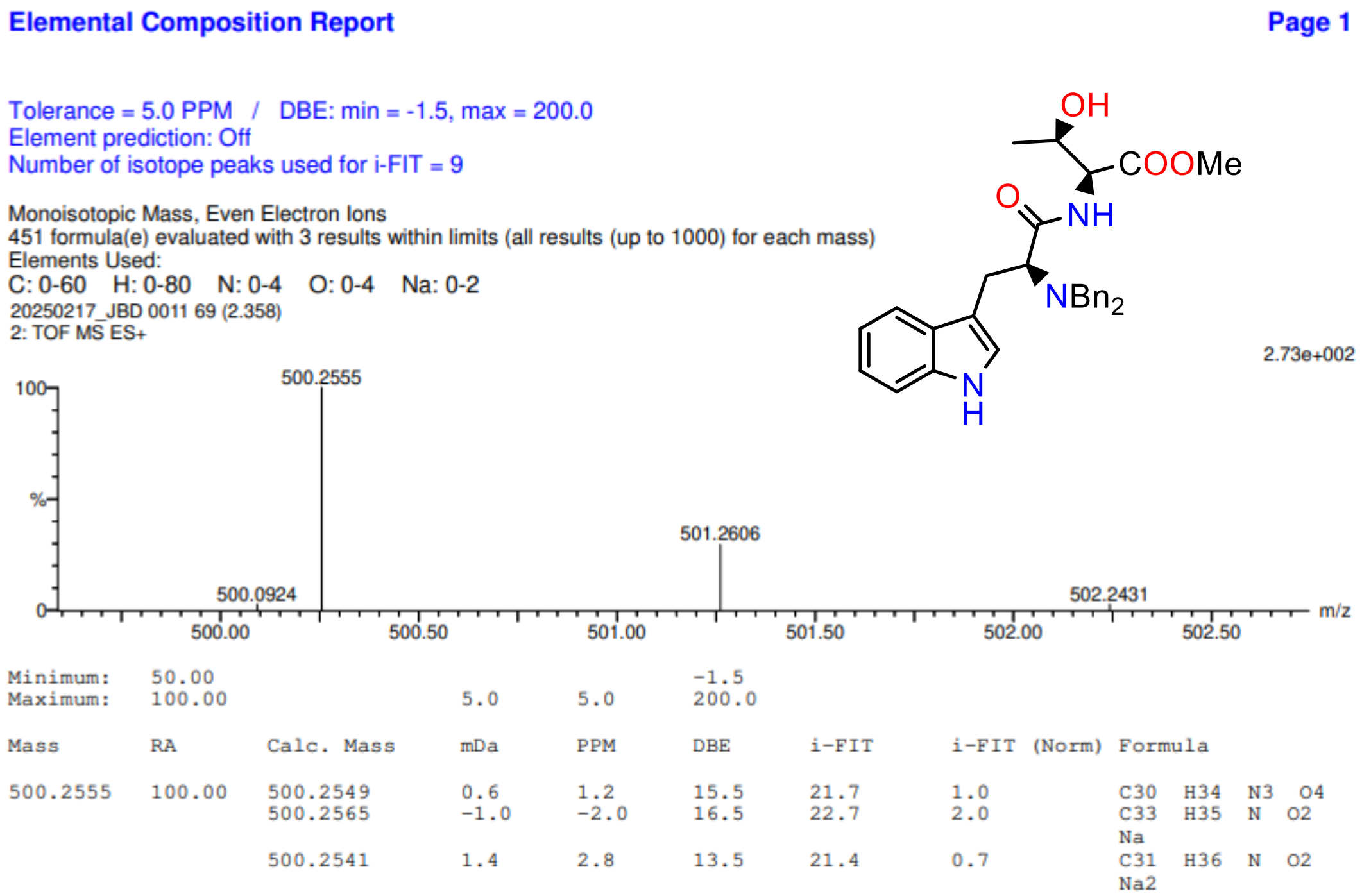

Fig S27 HRMS spectrum of methyl *N*,*N*-dibenzyl-L-tryptophyl-L-threoninate (**4g**)

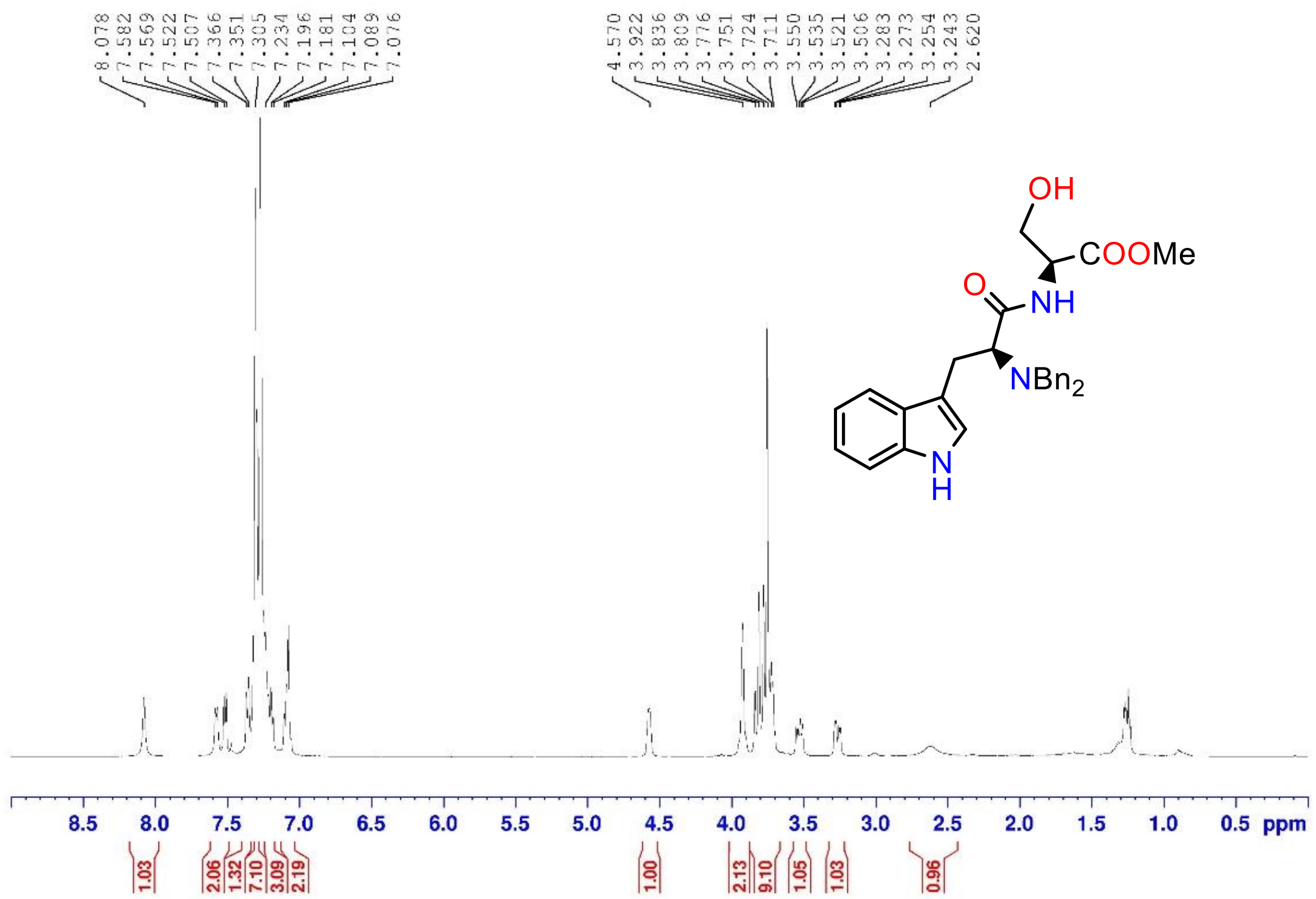

Fig S28 ^{1}H spectrum of methyl *N*,*N*-dibenzyl-L-tryptophyl-L-serinate (**4h**)

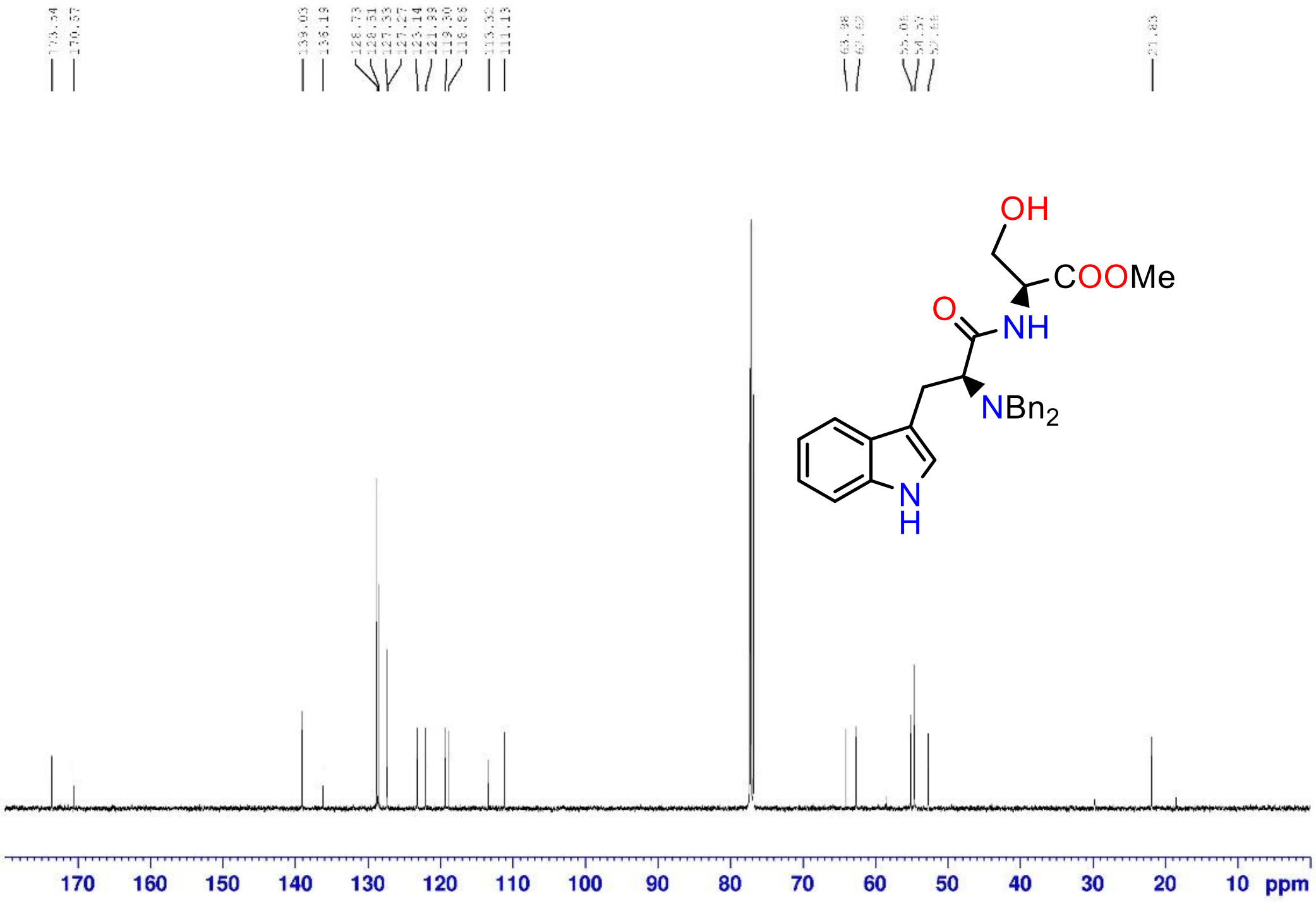

Fig S29 ^{13}C spectrum of methyl *N*,*N*-dibenzyl-L-tryptophyl-L-serinate (**4h**)

Elemental Composition Report **Page 1**

Tolerance = 5.0 PPM / DBE: min = -1.5, max = 200.0
Element prediction: Off
Number of isotope peaks used for i-FIT = 9

Monoisotopic Mass, Even Electron Ions
440 formula(e) evaluated with 4 results within limits (all results (up to 1000) for each mass)
Elements Used:
C: 0-60 H: 0-80 N: 0-4 O: 0-4 Na: 0-2
20250217_JBD 0012 57 (1.942)
2: TOF MS ES+

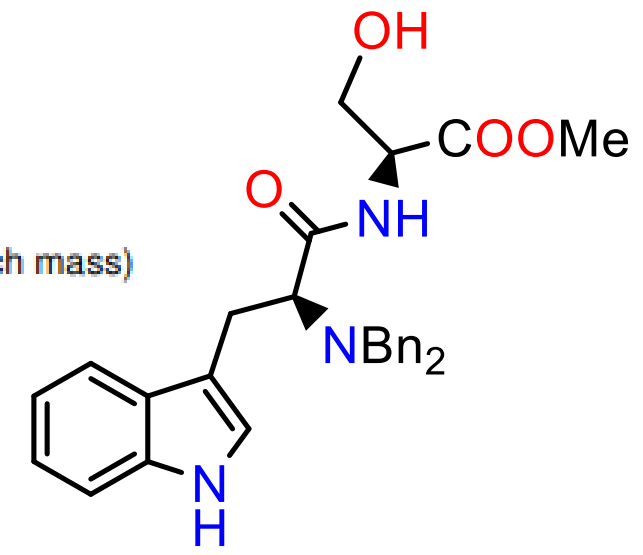

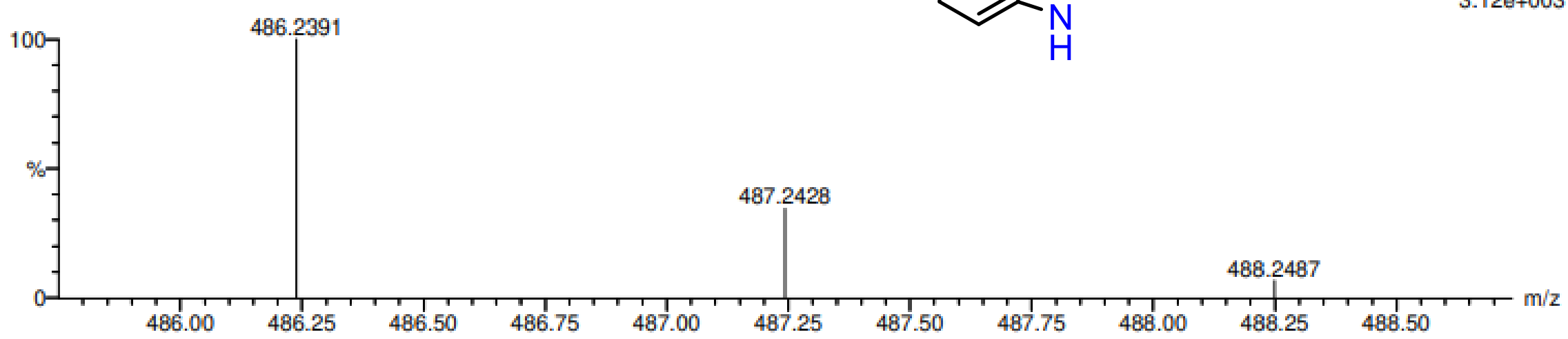

Minimum:	50.00				-1.5			
Maximum:	100.00		5.0	5.0	200.0			
Mass	RA	Calc. Mass	mDa	PPM	DBE	i-FIT	i-FIT (Norm)	Formula
486.2391	100.00	486.2393	-0.2	-0.4	15.5	37.3	1.7	C29 H32 N3 O4
		486.2385	0.6	1.2	13.5	36.1	0.4	C30 H34 N O2 Na2
		486.2409	-1.8	-3.7	16.5	38.1	2.4	C32 H33 N O2 Na
		486.2369	2.2	4.5	12.5	38.3	2.7	C27 H33 N3 O4 Na

Fig S30 HRMS spectrum of methyl *N*,*N*-dibenzyl-L-tryptophyl-L-serinate (**4h**)

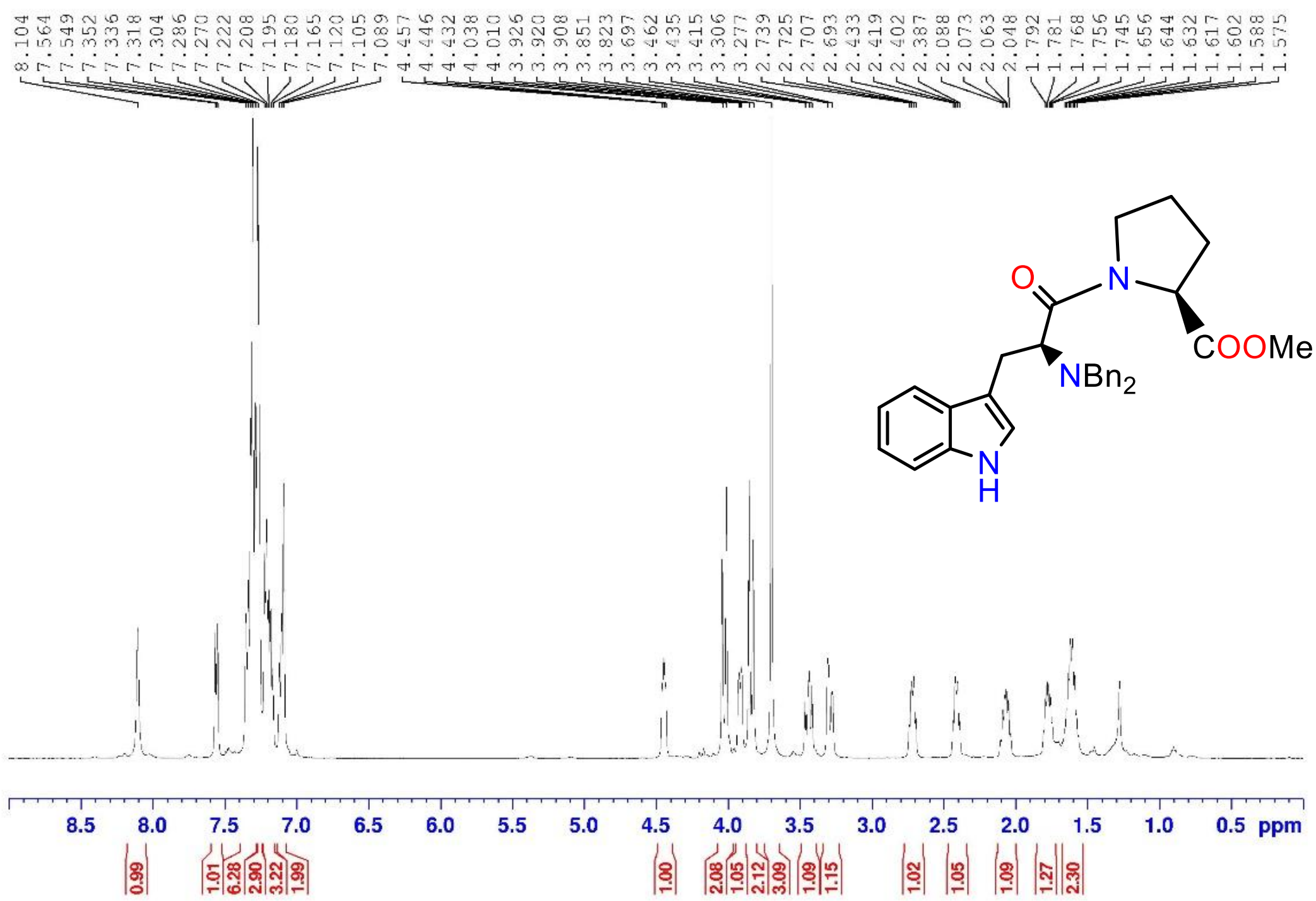

Fig S31 ^{1}H spectrum of methyl *N,N*-dibenzyl-L-tryptophyl-L-prolinate (**4i**)

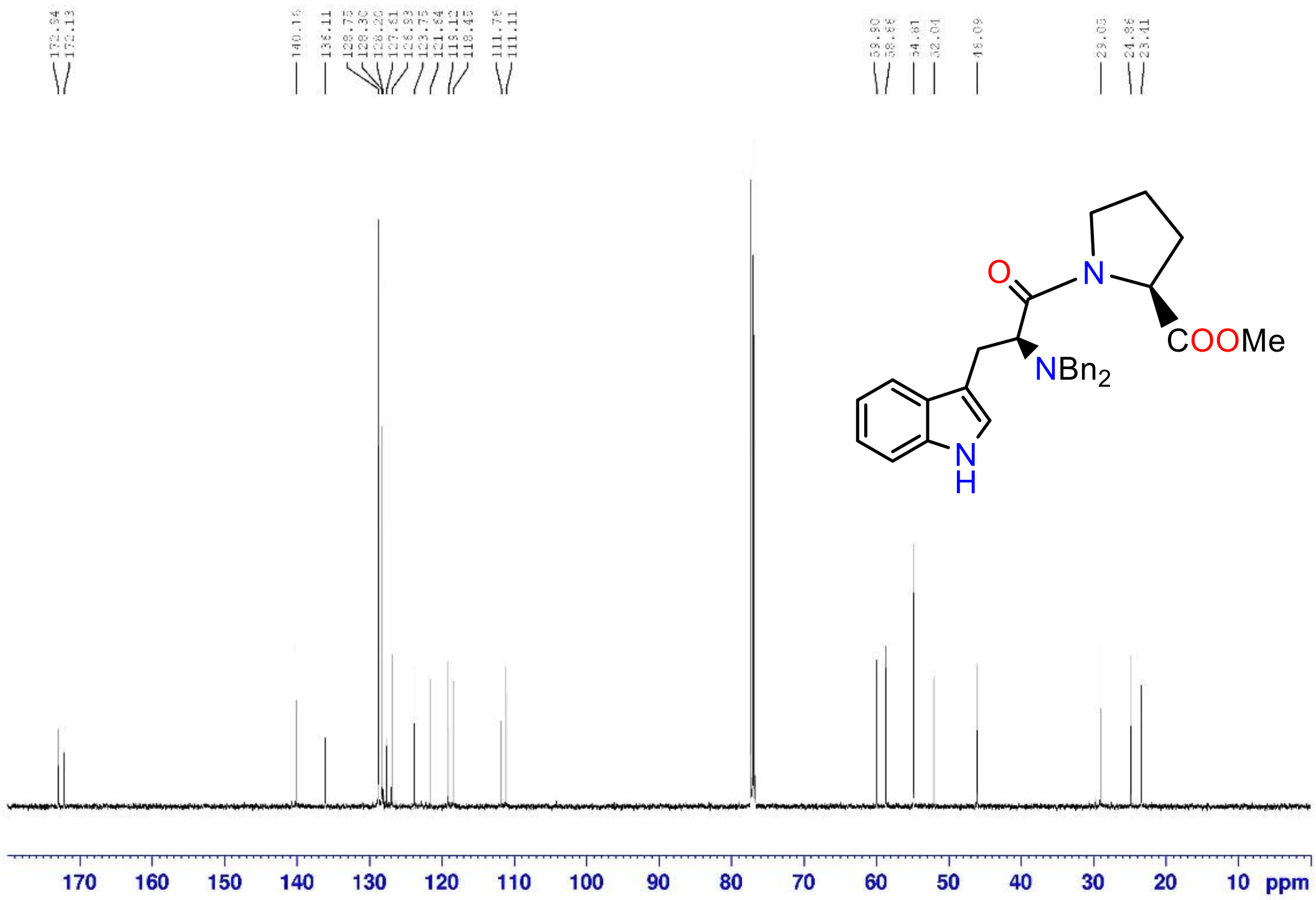

Fig S32 ^{13}C spectrum of methyl *N,N*-dibenzyl-L-tryptophyl-L-prolinate (**4i**)

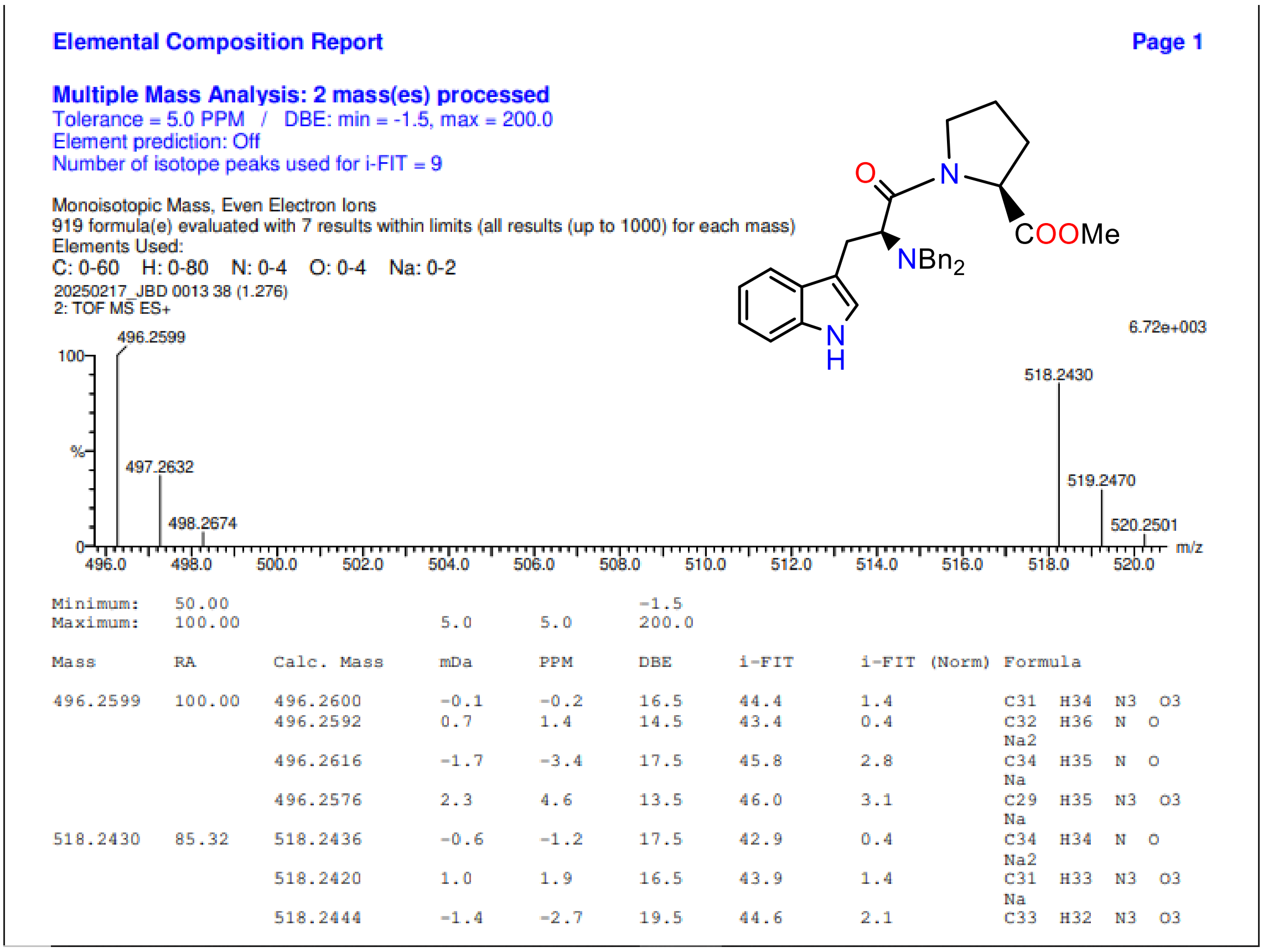

Fig S33 HRMS spectrum of methyl *N,N*-dibenzyl-L-tryptophyl-L-prolinate (**4i**)

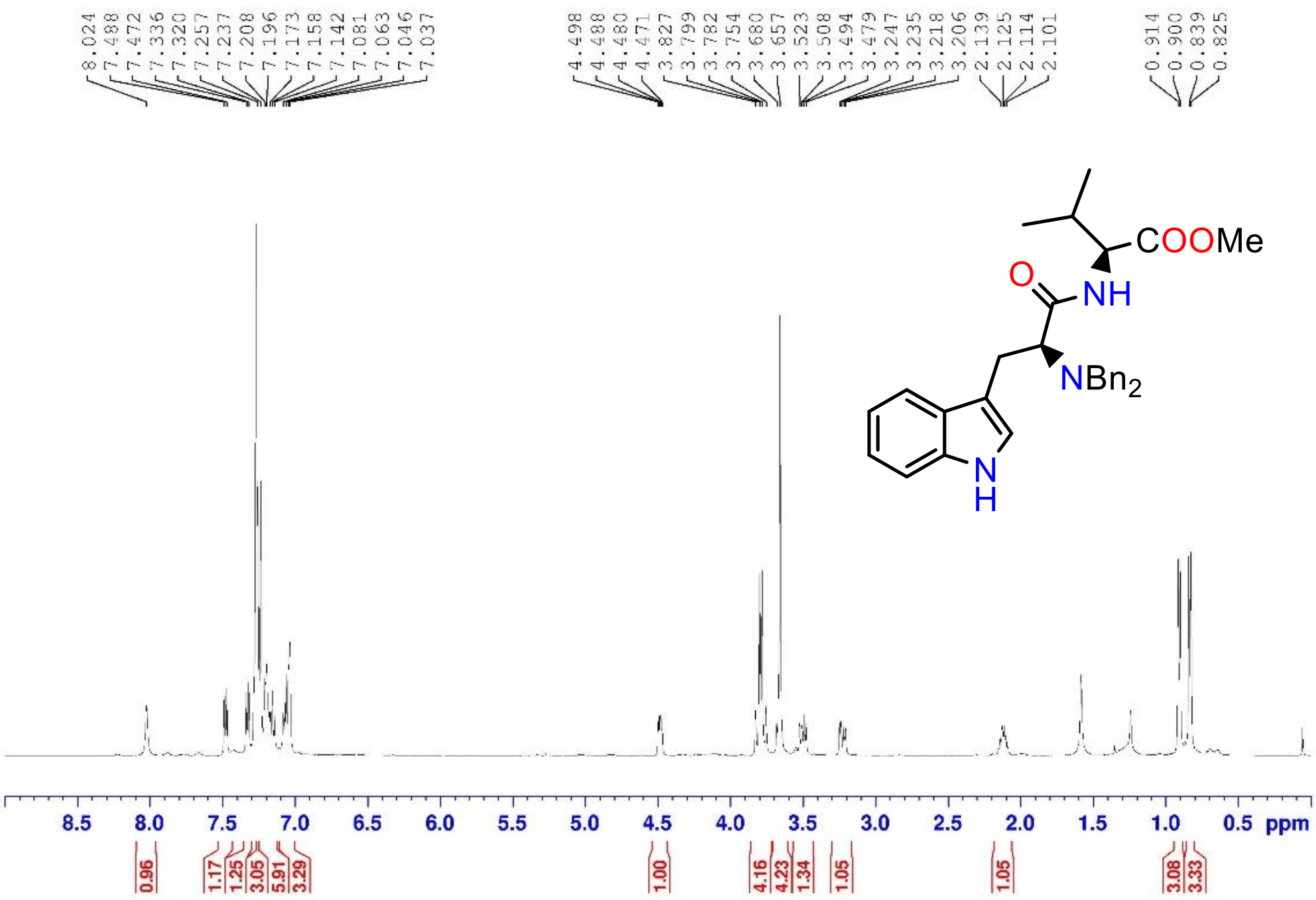

Fig S34 ^{1}H spectrum of methyl *N,N*-dibenzyl-L-tryptophyl-L-valinate (**4j**)

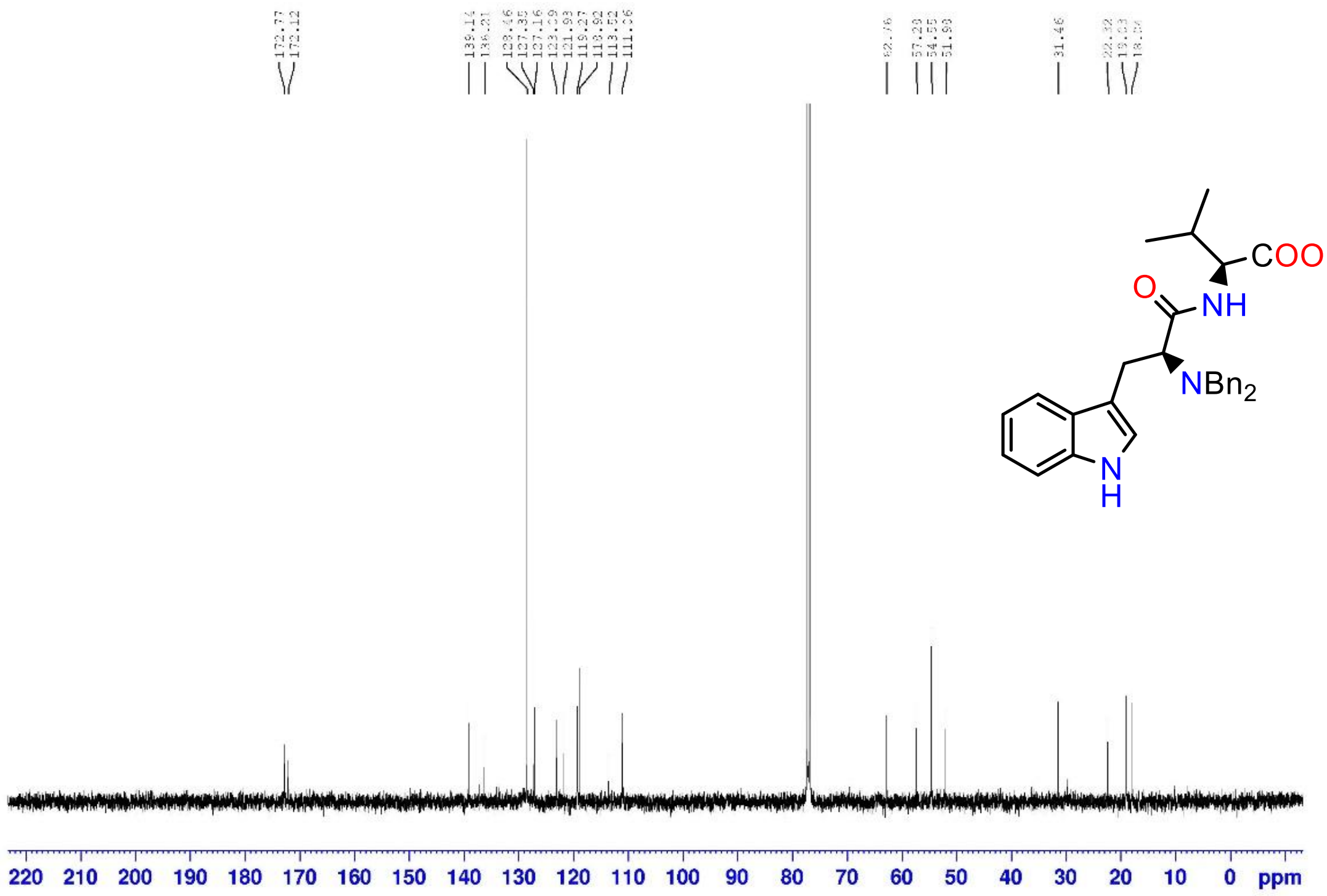

Fig S35 ^{13}C spectrum of methyl *N,N*-dibenzyl-L-tryptophyl-L-valinate (**4j**)

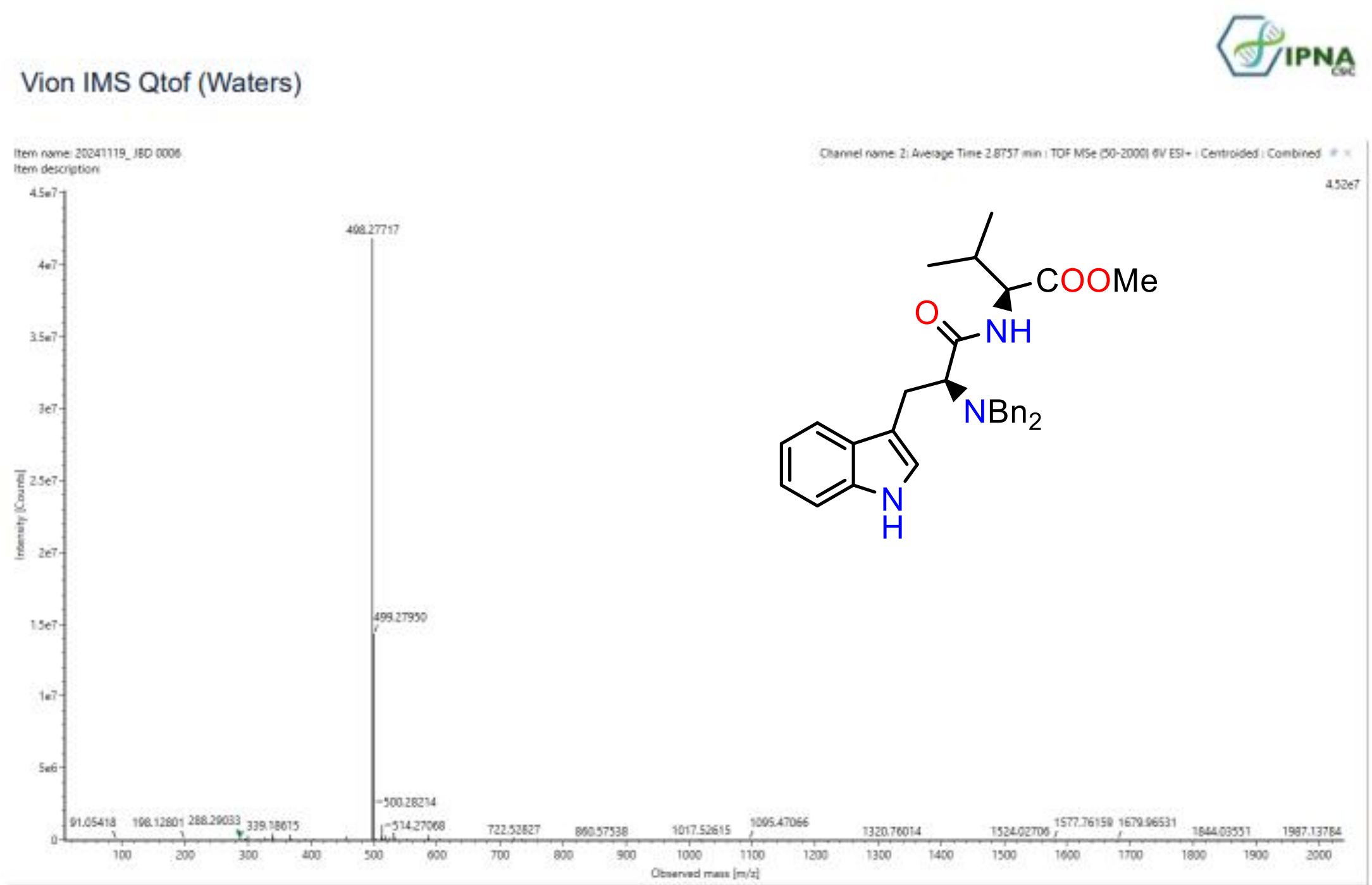

Fig S36 HRMS spectrum of methyl *N,N*-dibenzyl-L-tryptophyl-L-valinate (**4j**)

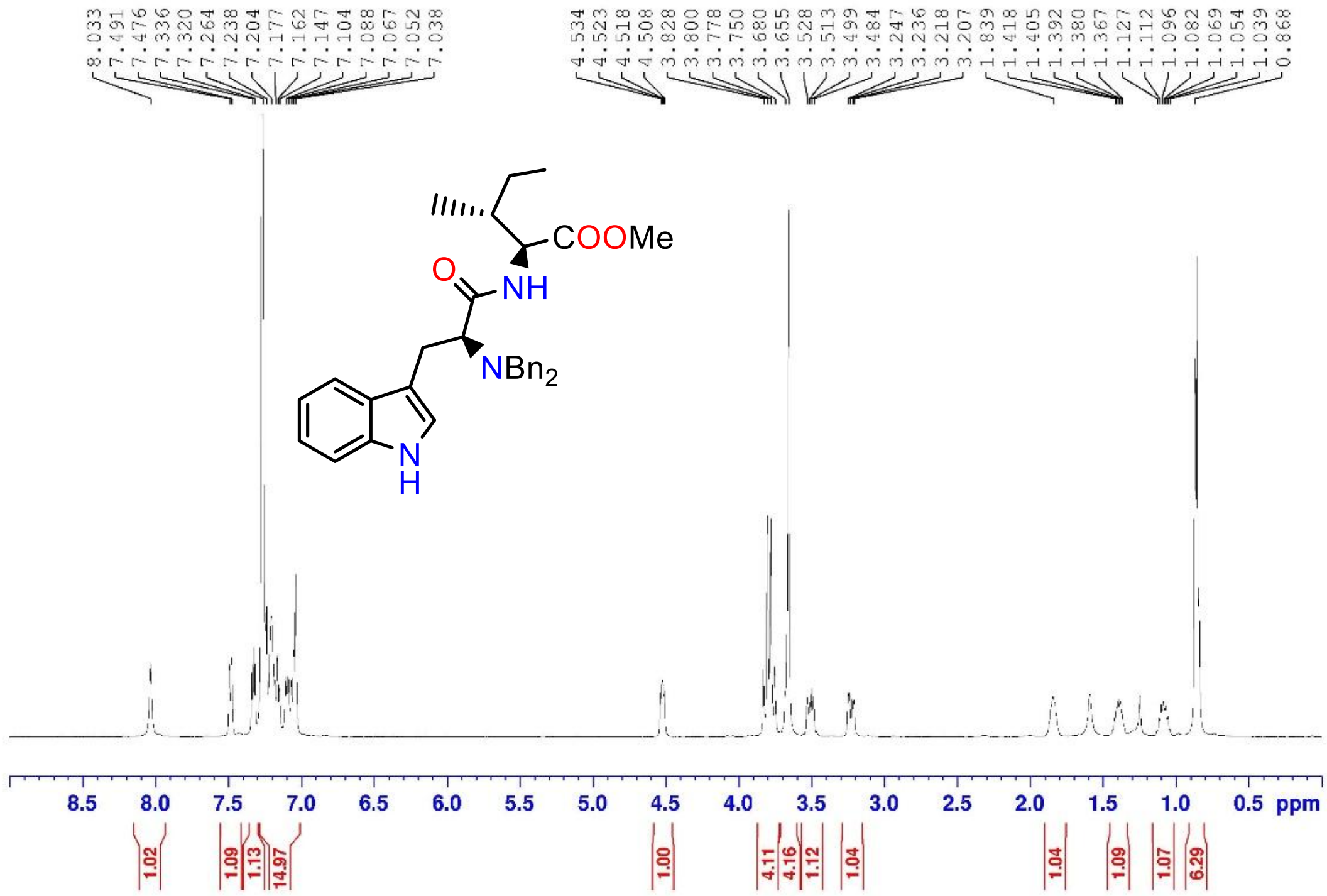

Fig S37 ^{1}H spectrum of methyl *N,N*-dibenzyl-L-tryptophyl-L-isoleucinate (**4k**)

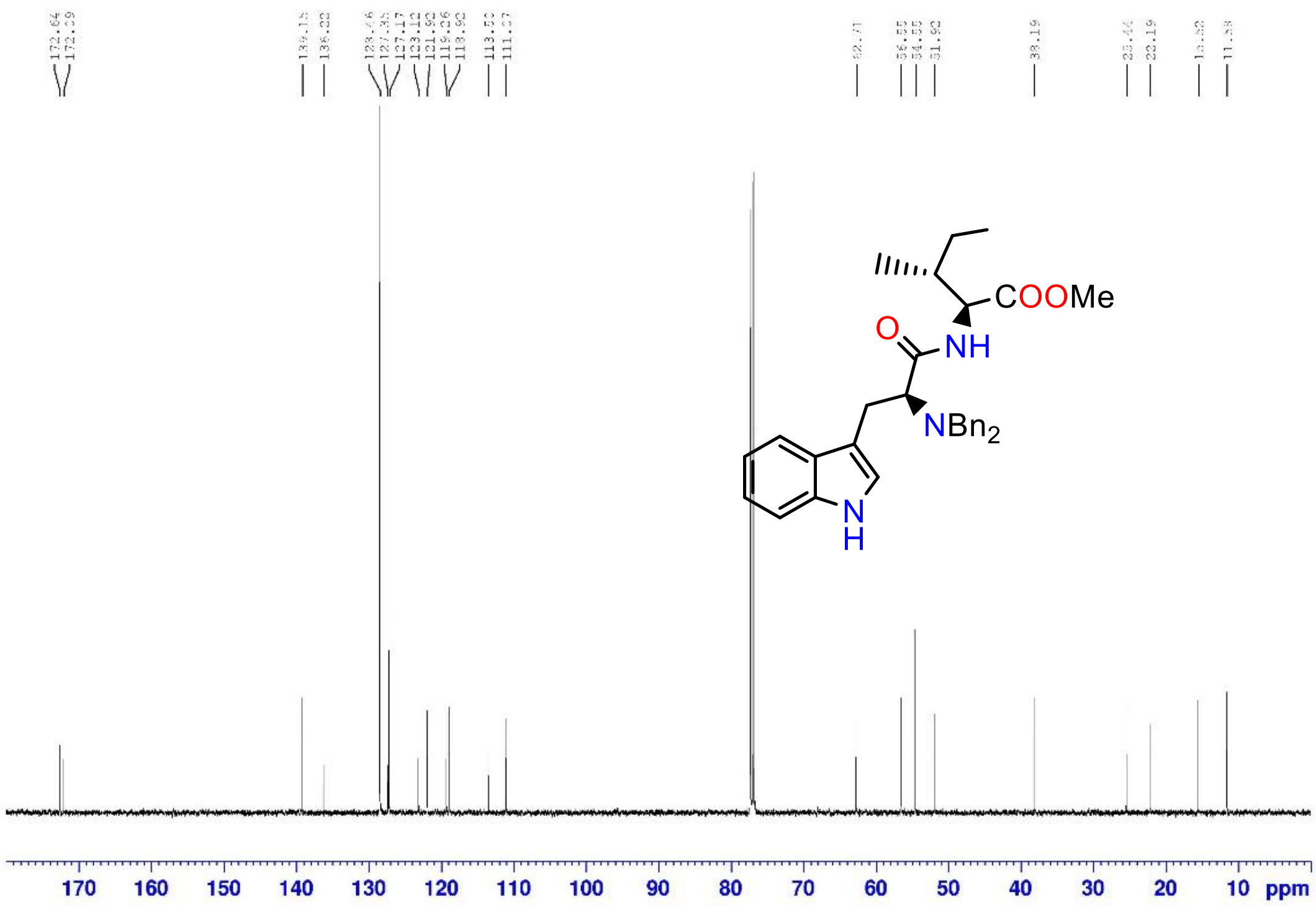

Fig S38 ^{13}C spectrum of methyl *N,N*-dibenzyl-L-tryptophyl-L-isoleucinate (**4k**)

Elemental Composition Report **Page 1**

Multiple Mass Analysis: 2 mass(es) processed
Tolerance = 10.0 PPM / DBE: min = -1.5, max = 200.0
Element prediction: Off
Number of isotope peaks used for i-FIT = 9

Monoisotopic Mass, Even Electron Ions
2361 formula(e) evaluated with 23 results within limits (all results (up to 1000) for each mass)
Elements Used:
C: 0-60 H: 0-80 N: 0-5 O: 0-11 Na: 0-2
20241209_JBD 0008 279 (12.135)
1: TOF MS ES+

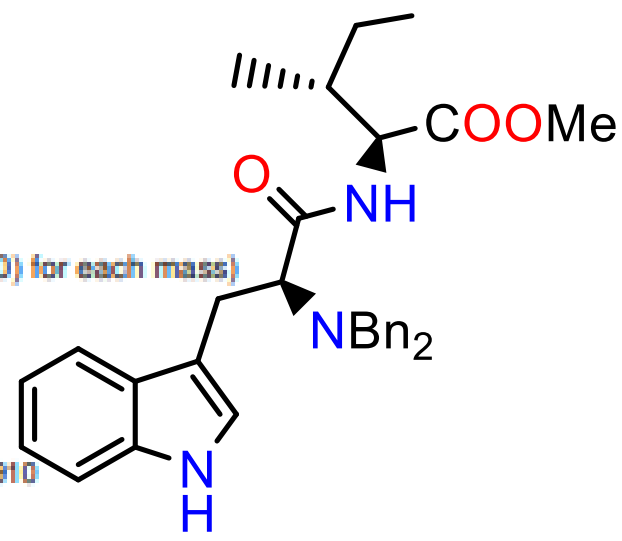

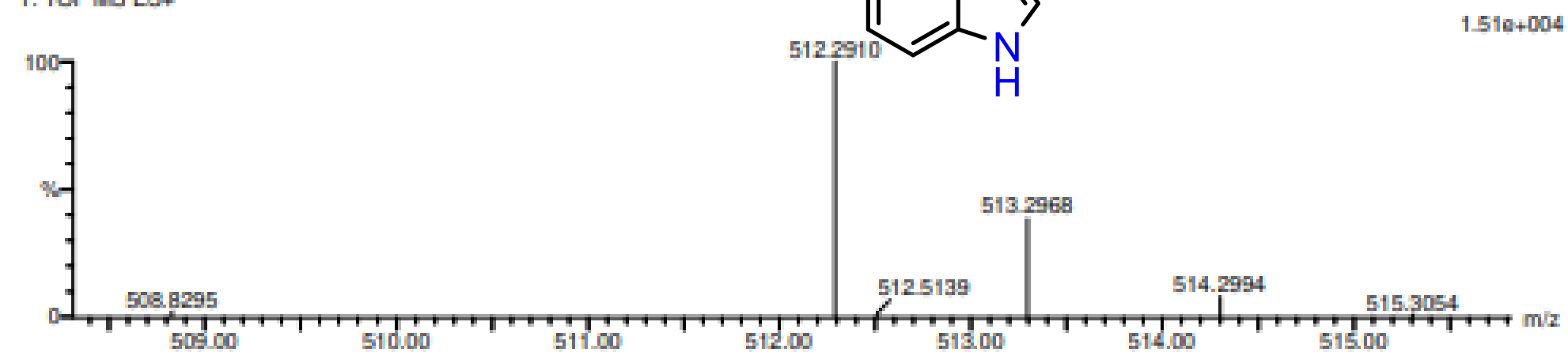

Minimum:	15.00				-1.5			
Maximum:	100.00		5.0	10.0	200.0			

Mass	RA	Calc. Mass	mDa	PPM	DBE	i-FIT	i-FIT (Norm)	Formula
512.2910	100.00	512.2908	0.2	0.4	-0.5	54.3	11.7	C18 H43 N5 O10 Na
		512.2913	-0.3	-0.6	15.5	45.7	3.1	C32 H38 N3 O3
		512.2905	0.5	1.0	13.5	42.9	0.3	C33 H40 N O Na2
		512.2924	-1.4	-2.7	0.5	52.2	9.6	C21 H44 N3 O8 Na2
		512.2929	-1.9	-3.7	16.5	44.5	1.9	C35 H39 N O Na
		512.2889	2.1	4.1	12.5	45.6	3.1	C30 H39 N3 O3 Na
		512.2932	-2.2	-4.3	2.5	52.9	10.3	C20 H42 N5 O10
		512.2873	3.7	7.2	11.5	48.5	5.9	C27 H38 N5 O5
		512.2948	-3.8	-7.4	3.5	51.1	8.5	C23 H43 N3 O8 Na
		512.2953	-4.3	-8.4	19.5	47.6	5.0	C37 H38 N O
		512.2865	4.5	8.8	9.5	47.3	4.7	C28 H40 N3 O3 Na2
		512.2860	5.0	9.8	6.5	51.3	8.7	C26 H42 N O9
513.2968	38.32	513.2970	-0.2	-0.4	13.5	41.4	0.8	C31 H39 N4 Na2
		513.2965	0.3	0.6	10.5	43.2	2.7	C29 H41 N2 O6
		513.2957	1.1	2.1	8.5	42.5	2.0	C30 H43 O4 Na2
		513.2981	-1.3	-2.5	11.5	43.7	3.1	C32 H42 O4 Na
		513.2994	-2.6	-5.1	16.5	43.6	3.1	C33 H38 N4 Na
		513.2941	2.7	5.3	7.5	43.2	2.6	C27 H42 N2 O6 Na
		513.2999	-3.1	-6.0	-1.5	42.6	2.0	C20 H46 N2 O11 Na
		513.3005	-3.7	-7.2	14.5	46.1	5.6	C34 H41 O4
		513.2924	4.4	8.6	6.5	44.2	3.7	C24 H41 N4 O8
		513.3015	-4.7	-9.2	-0.5	43.9	3.3	C23 H47 O9 Na2
		513.3018	-5.0	-9.7	19.5	46.4	5.8	C35 H37 N4

Fig S39 HRMS spectrum of methyl *N,N*-dibenzyl-L-tryptophyl-L-isoleucinate (**4k**)

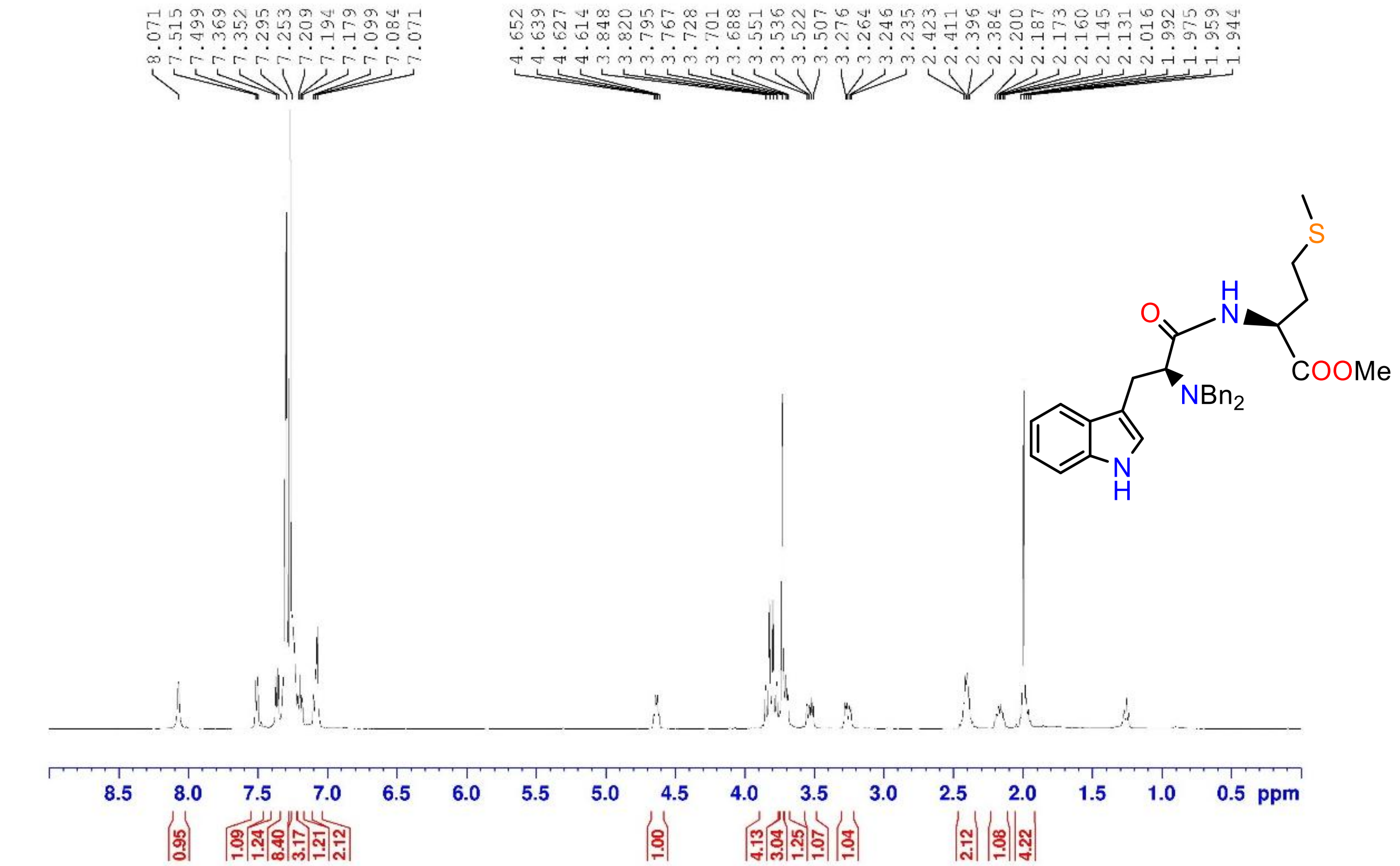

Fig S40 ^{1}H spectrum of methyl *N,N*-dibenzyl-L-tryptophyl-L-methioninate (**4l**)

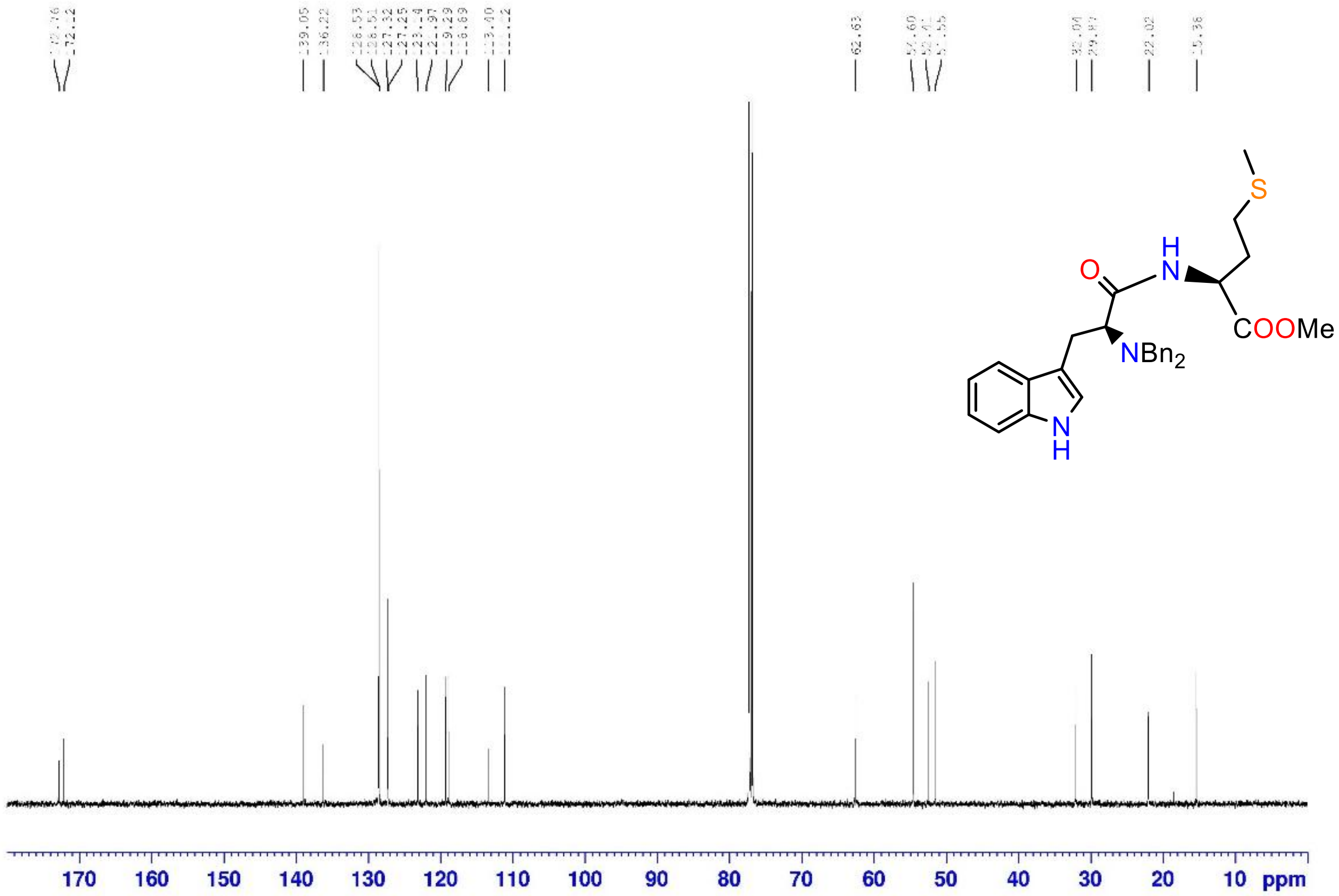

Fig S41 ^{13}C spectrum of methyl *N,N*-dibenzyl-L-tryptophyl-L-methioninate (**4l**)

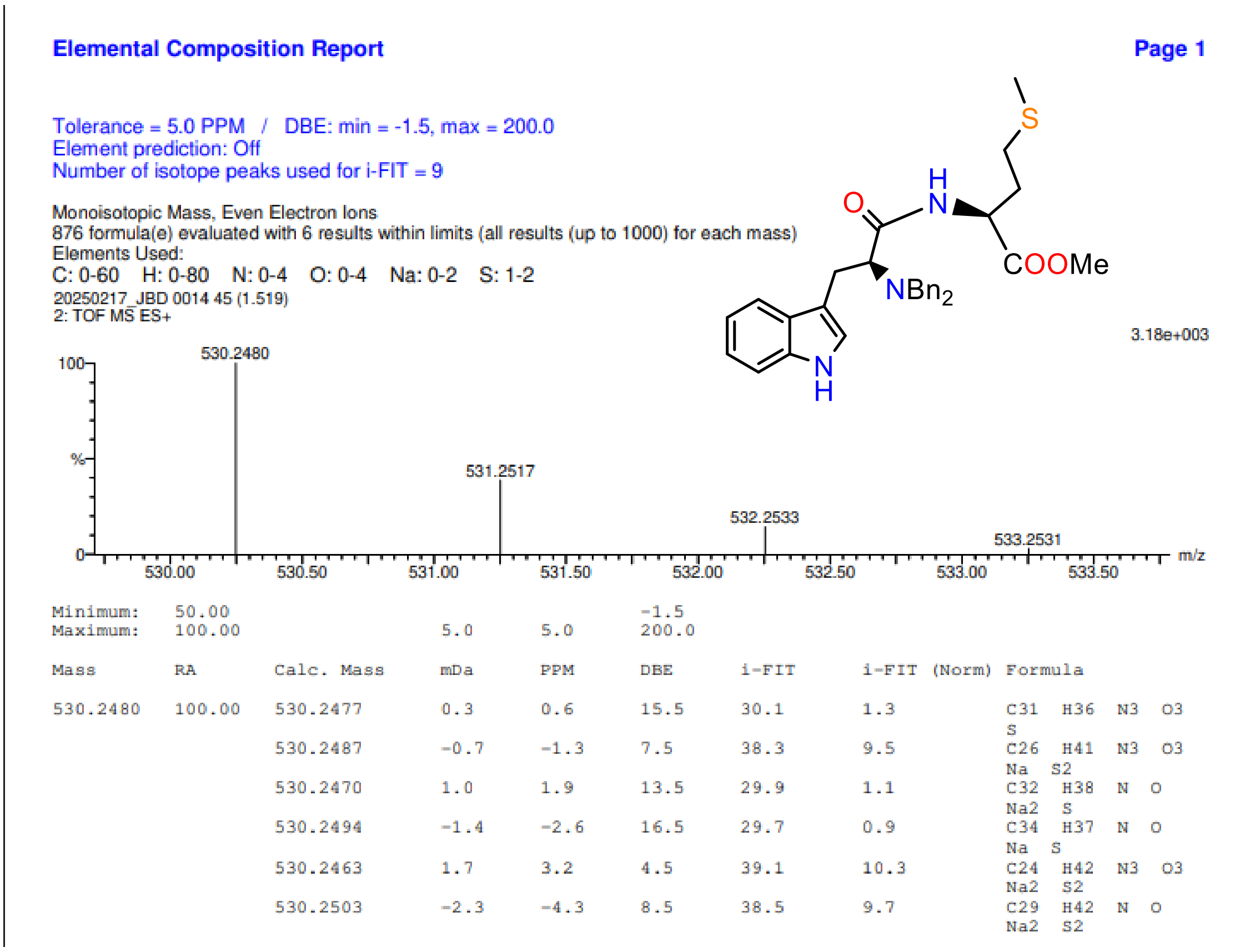

Minimum:	50.00				-1.5			
Maximum:	100.00		5.0	5.0	200.0			
Mass	RA	Calc. Mass	mDa	PPM	DBE	i-FIT	i-FIT (Norm)	Formula
530.2480	100.00	530.2477	0.3	0.6	15.5	30.1	1.3	C31 H36 N3 O3 S
		530.2487	-0.7	-1.3	7.5	38.3	9.5	C26 H41 N3 O3 Na S2
		530.2470	1.0	1.9	13.5	29.9	1.1	C32 H38 N O Na2 S
		530.2494	-1.4	-2.6	16.5	29.7	0.9	C34 H37 N O Na S
		530.2463	1.7	3.2	4.5	39.1	10.3	C24 H42 N3 O3 Na2 S2
		530.2503	-2.3	-4.3	8.5	38.5	9.7	C29 H42 N O Na2 S2

Fig S42 HRMS spectrum of methyl *N,N*-dibenzyl-L-tryptophyl-L-methioninate (**4l**)

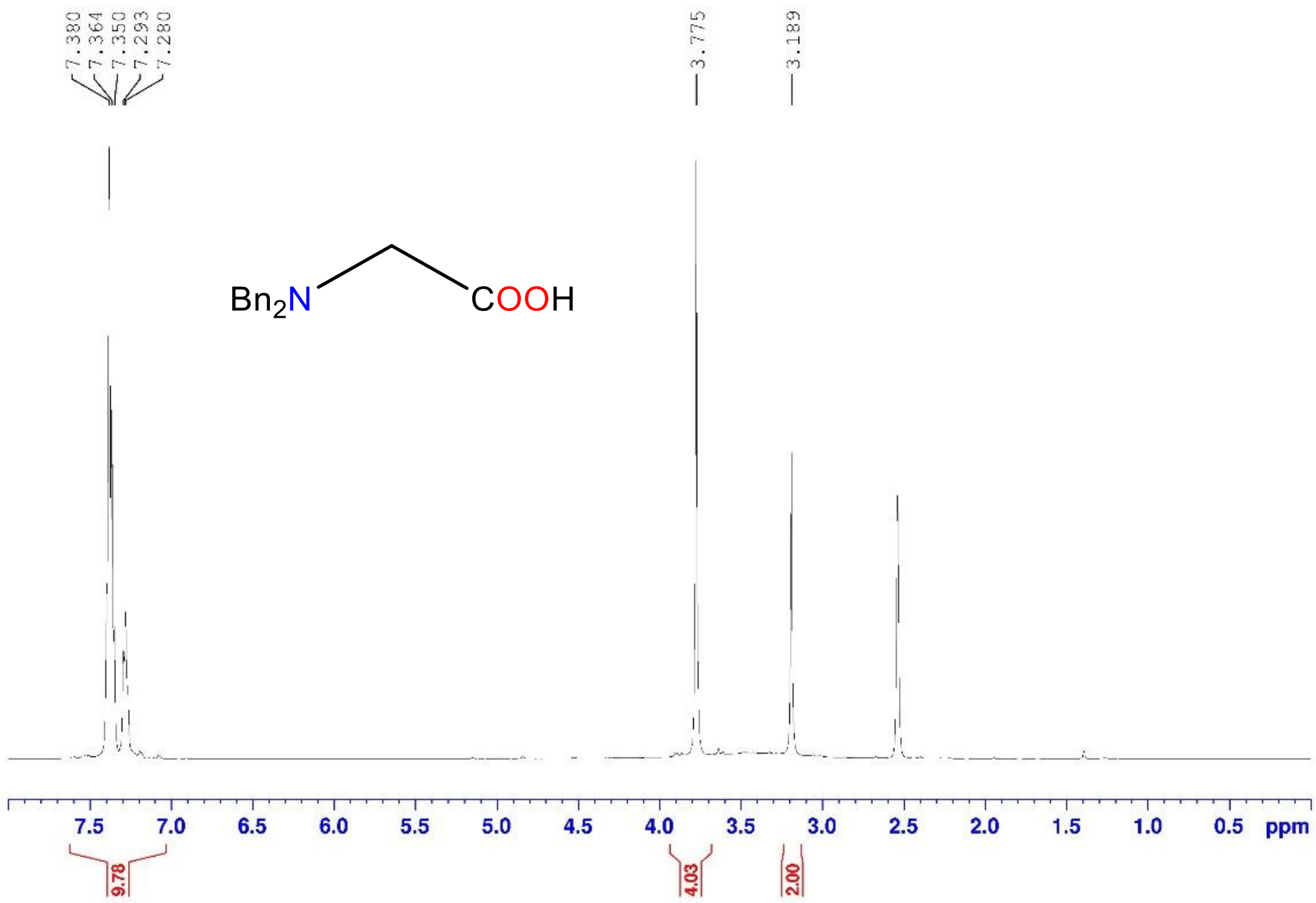

Fig S43 ^{1}H spectrum of *N,N*-dibenzyl-glycine (**7a**)

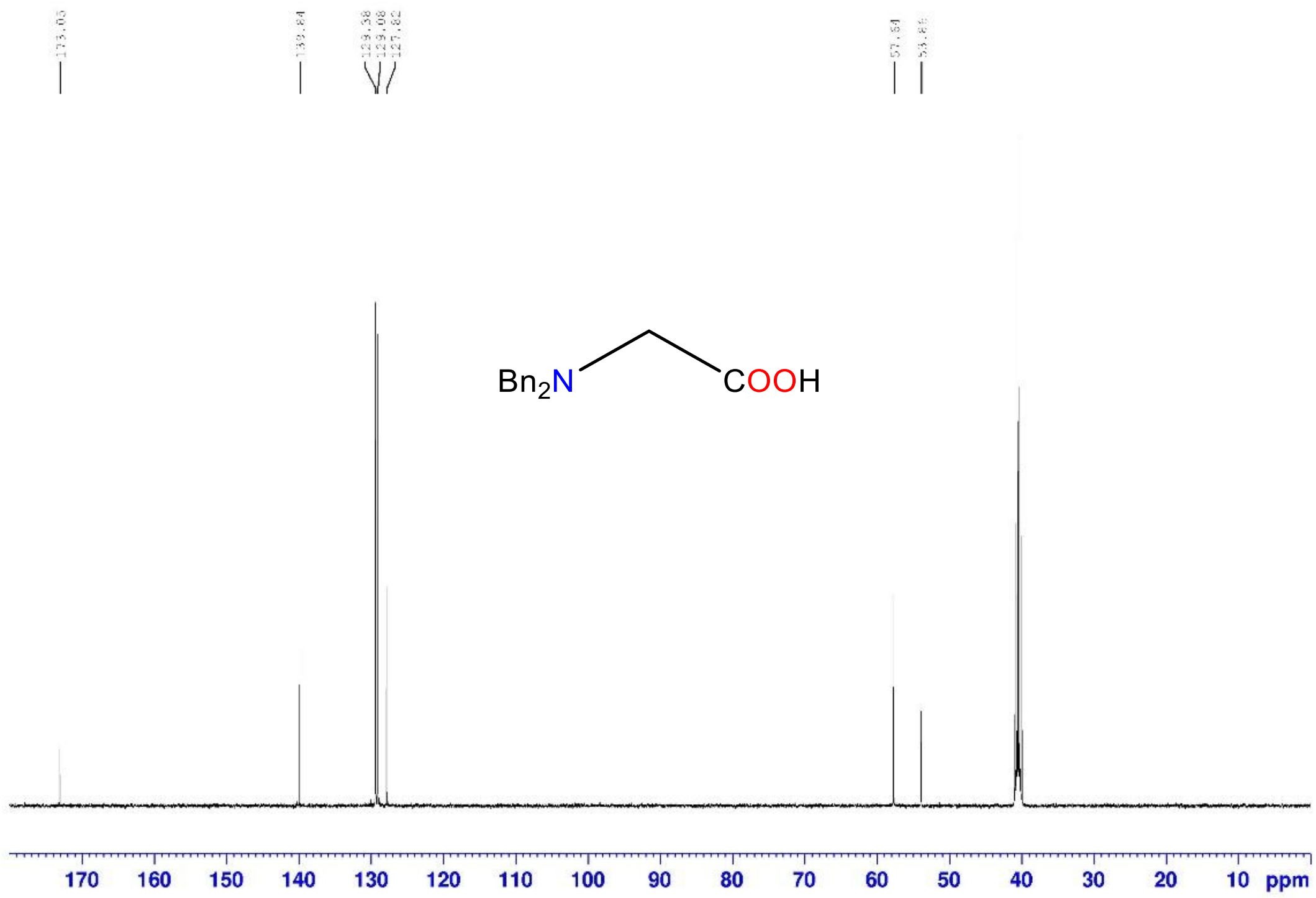

Fig S44 ^{13}C spectrum of *N,N*-dibenzyl-glycine (**7a**)

Elemental Composition Report **Page 1**

Tolerance = 5.0 PPM / DBE: min = -2.0, max = 230.0
Element prediction: Off
Number of isotope peaks used for i-FIT = 9

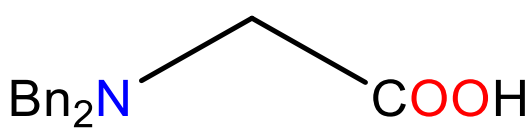

Monoisotopic Mass, Even Electron Ions
27 formula(e) evaluated with 1 results within limits (all results (up to 1000) for each mass)
Elements Used:
C: 0-25 H: 0-30 N: 0-1 O: 0-4
SYNAPT-XS#NotSet 20260720_JBD0015 - I 220 (1.601)
1: TOF MS ES+

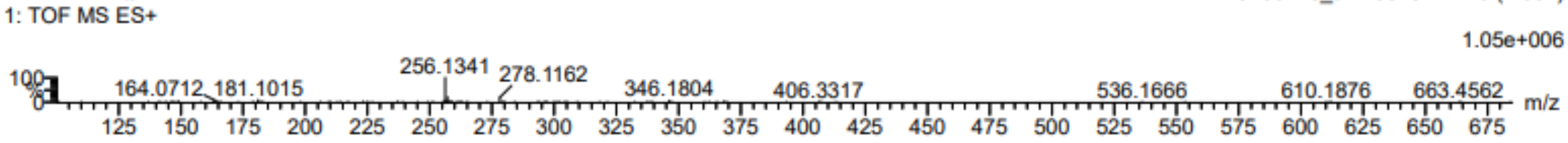

Minimum:	30.00				-2.0				
Maximum:	100.00		5.0	5.0	230.0				
Mass	RA	Calc. Mass	mDa	PPM	DBE	i-FIT	Norm	Conf(%)	Formula
256.1341	100.00	256.1338	0.3	1.2	8.5	159.9	n/a	n/a	C16 H18 N O2

Fig S45 HRMS spectrum of *N,N*-dibenzyl-glycine (**7a**)

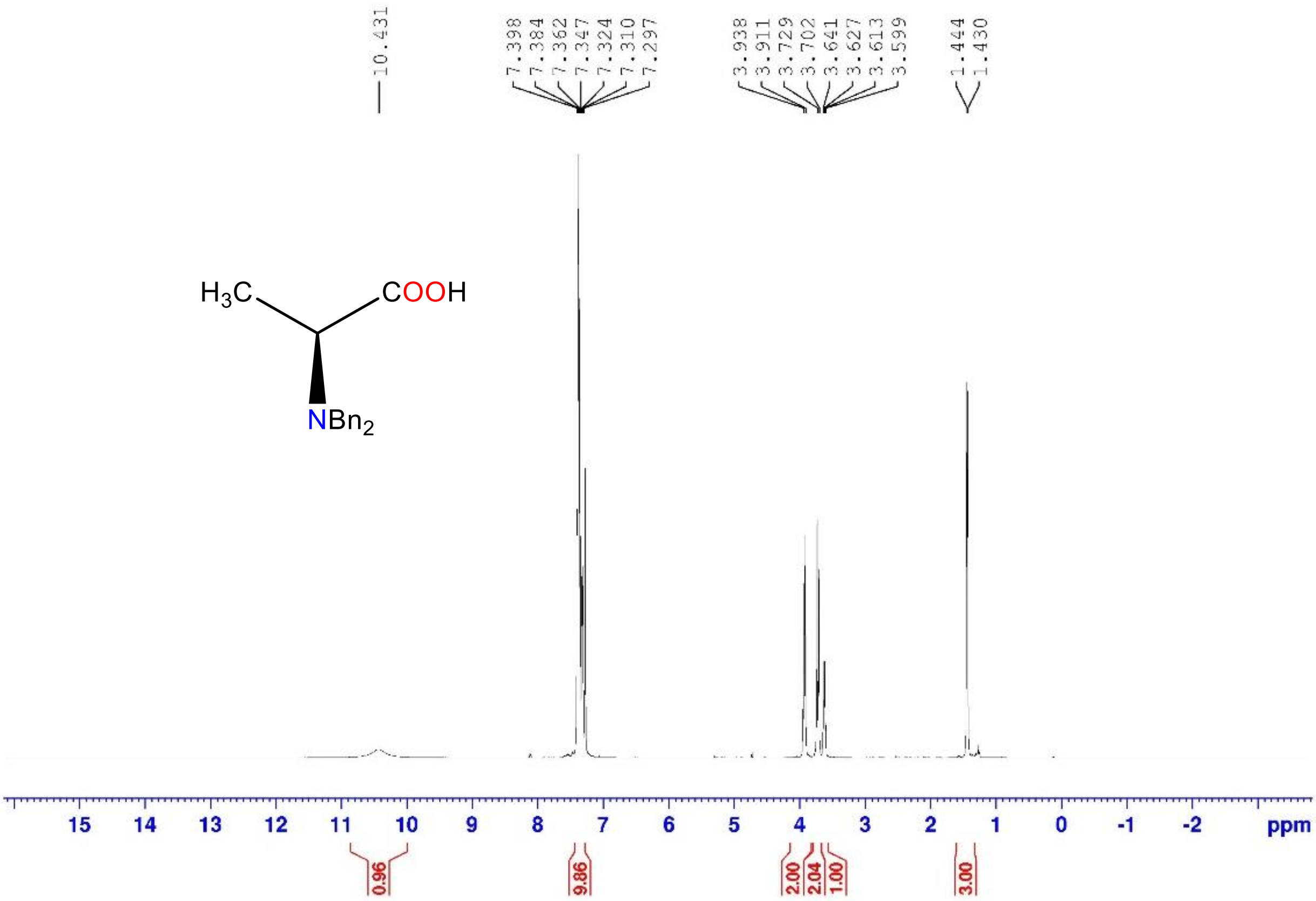

Fig S46 ^{1}H spectrum of *N,N*-dibenzyl-L-alanine (**7b**)

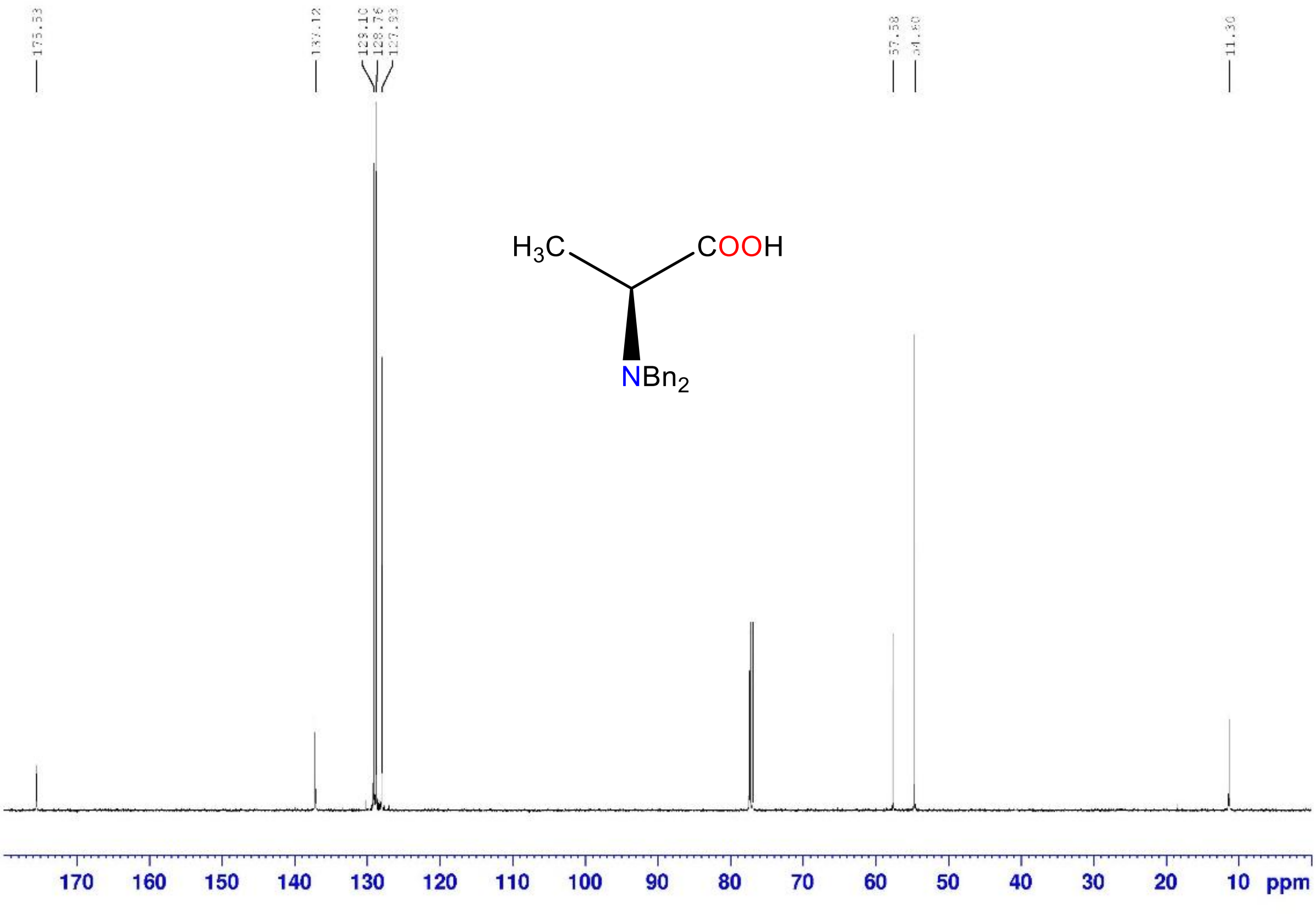

Fig S47 ^{13}C spectrum of *N,N*-dibenzyl-L-alanine (**7b**)

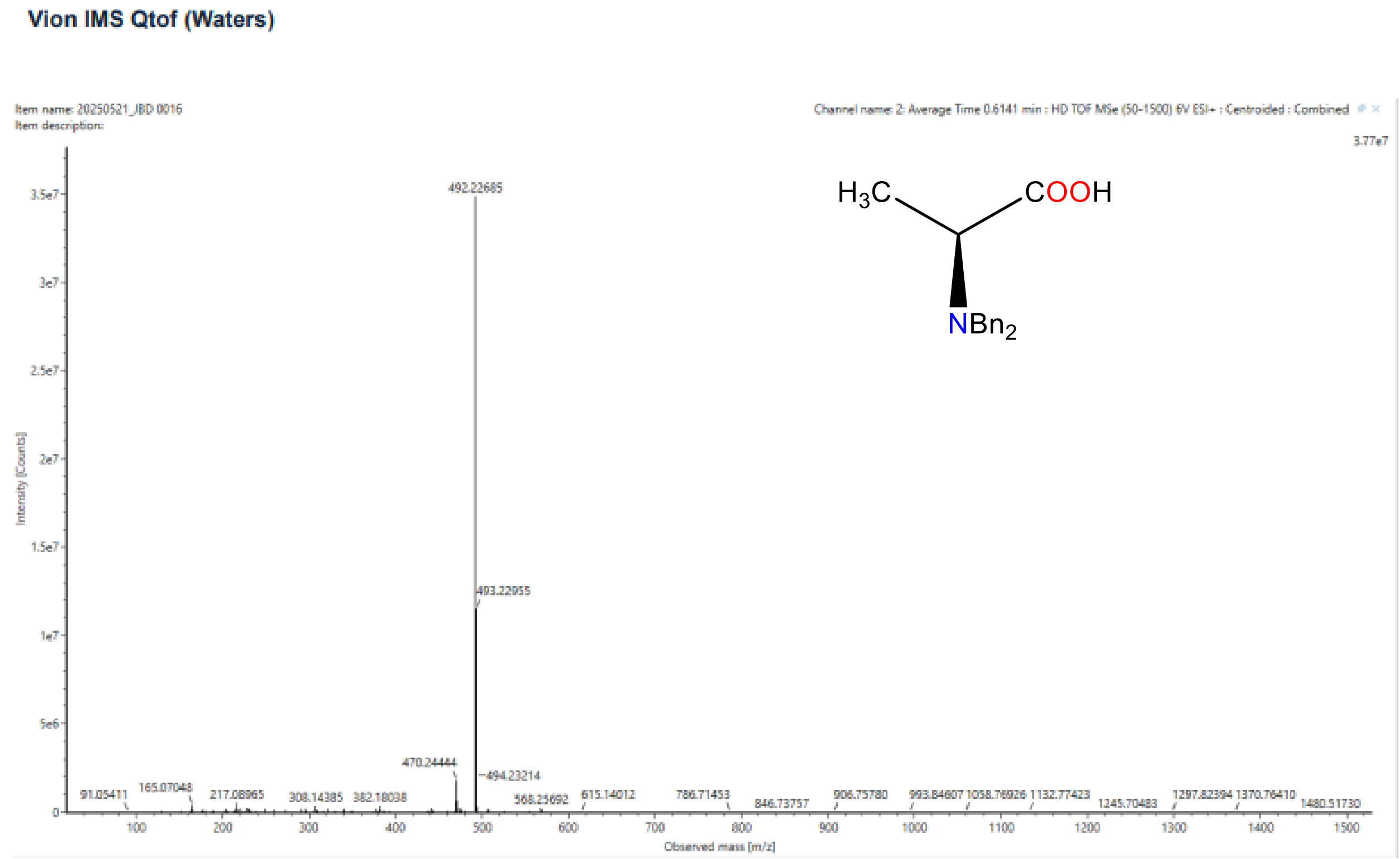

Fig S48 HRMS spectrum of *N,N*-dibenzyl-L-alanine (**7b**)

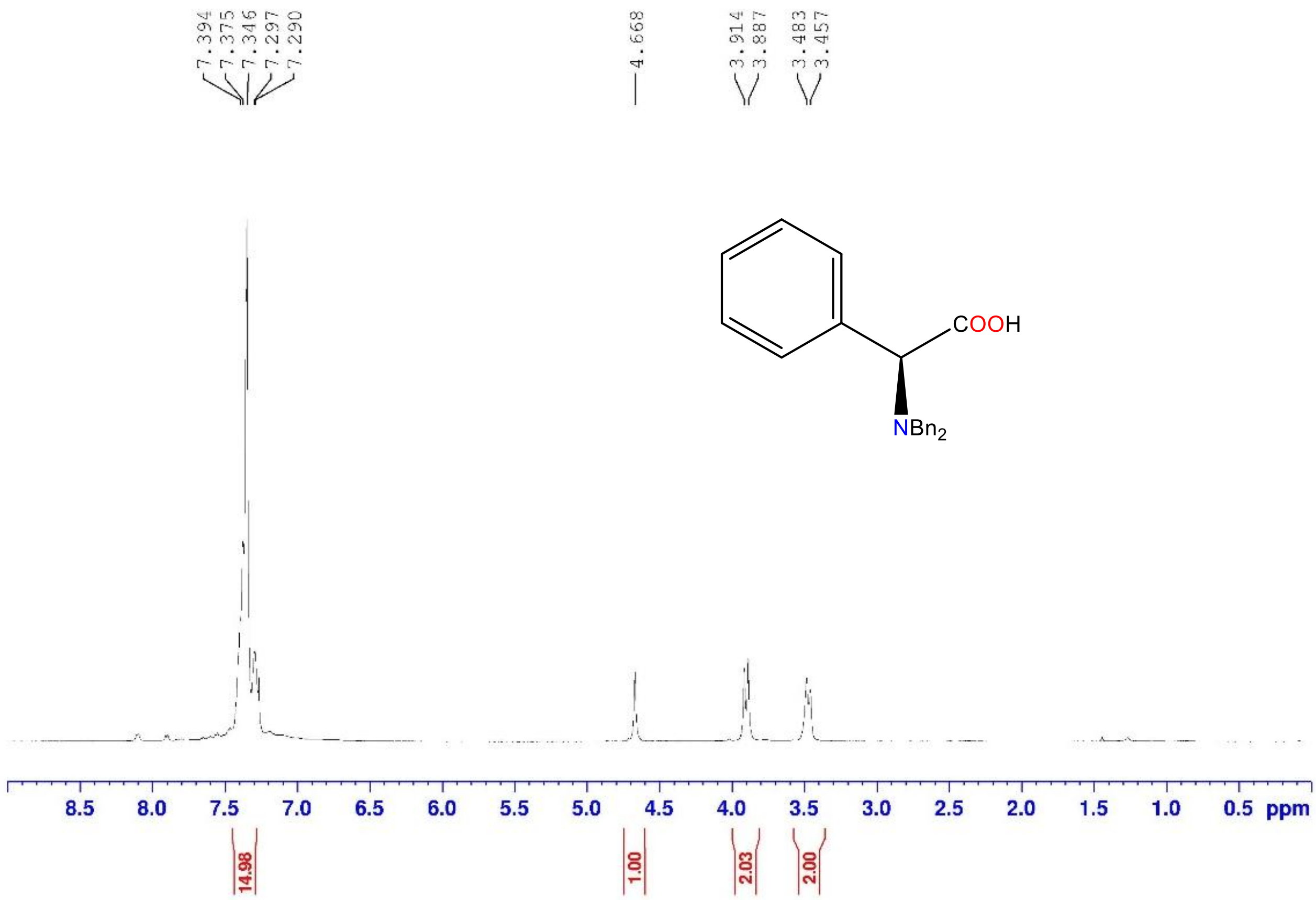

Fig S49 ^{1}H spectrum of *N,N*-dibenzyl-L-phenylglycine (**7c**)

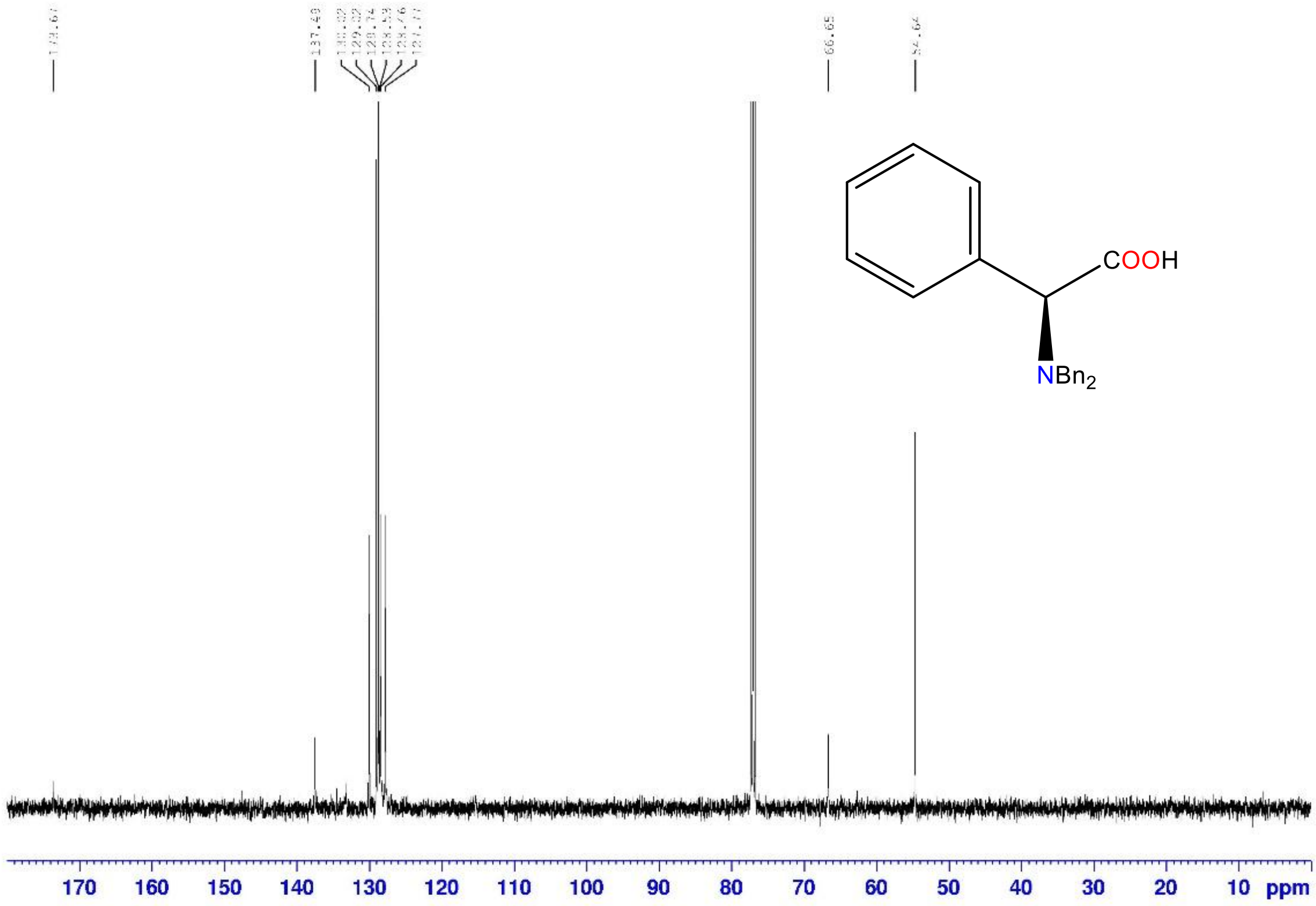

Fig S50 ^{13}C spectrum of *N,N*-dibenzyl-L-phenylglycine (**7c**)

Vion IMS Qtof (Waters)

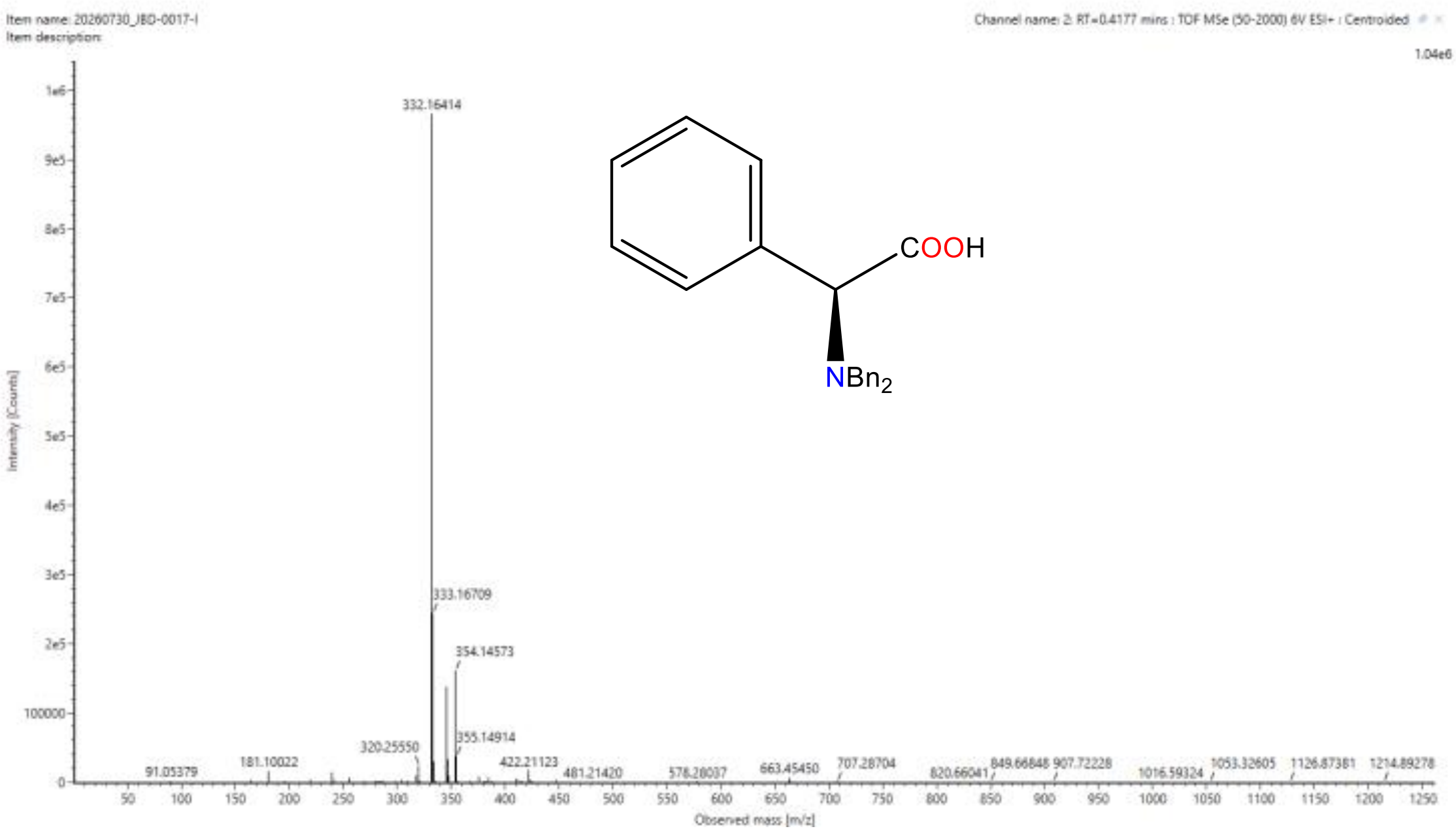

Fig S51 HRMS spectrum of *N,N*-dibenzyl-L-phenylglycine (**7c**)

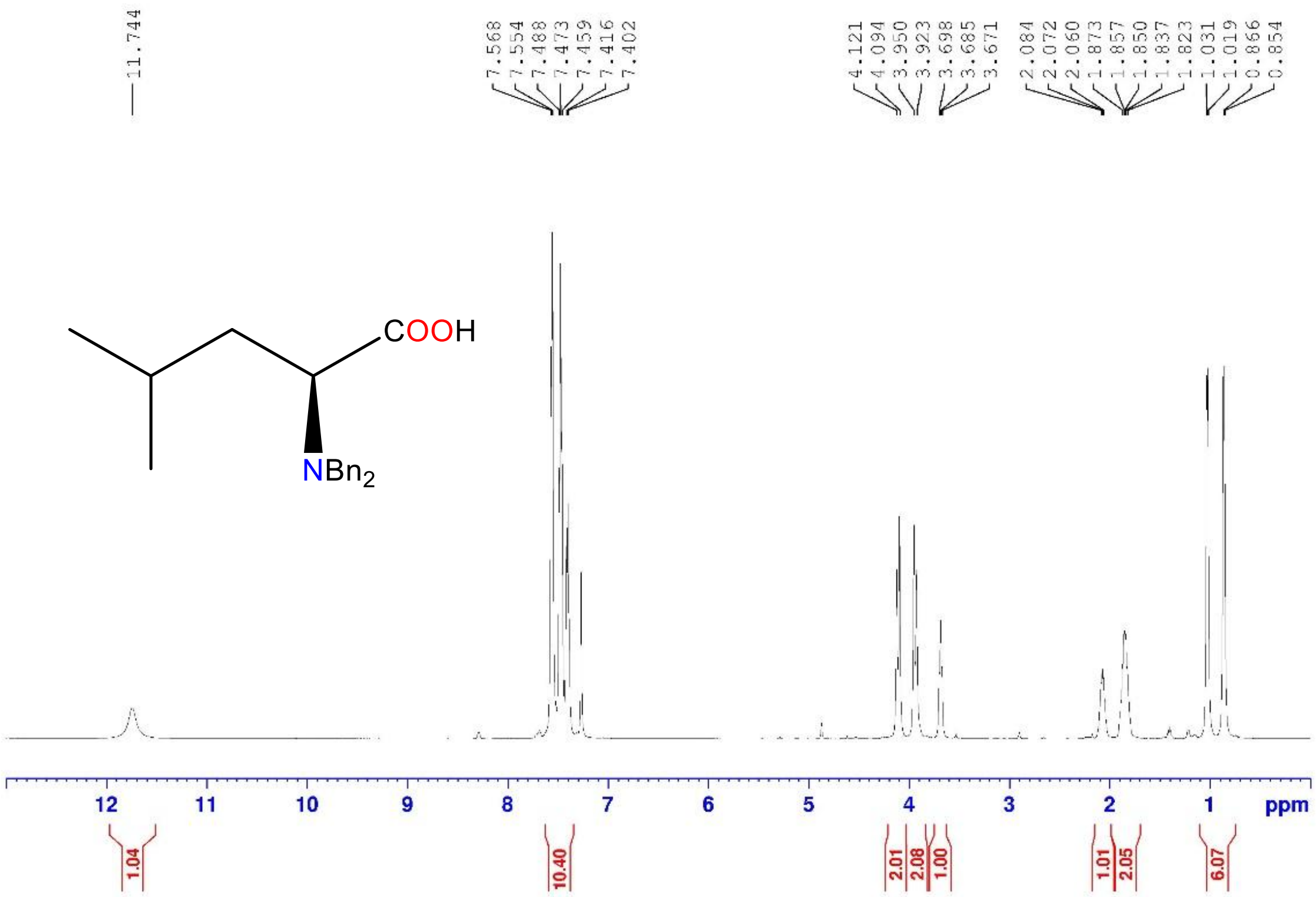

Fig S52 ^{1}H spectrum of *N,N*-dibenzyl-L-leucine (**7d**)

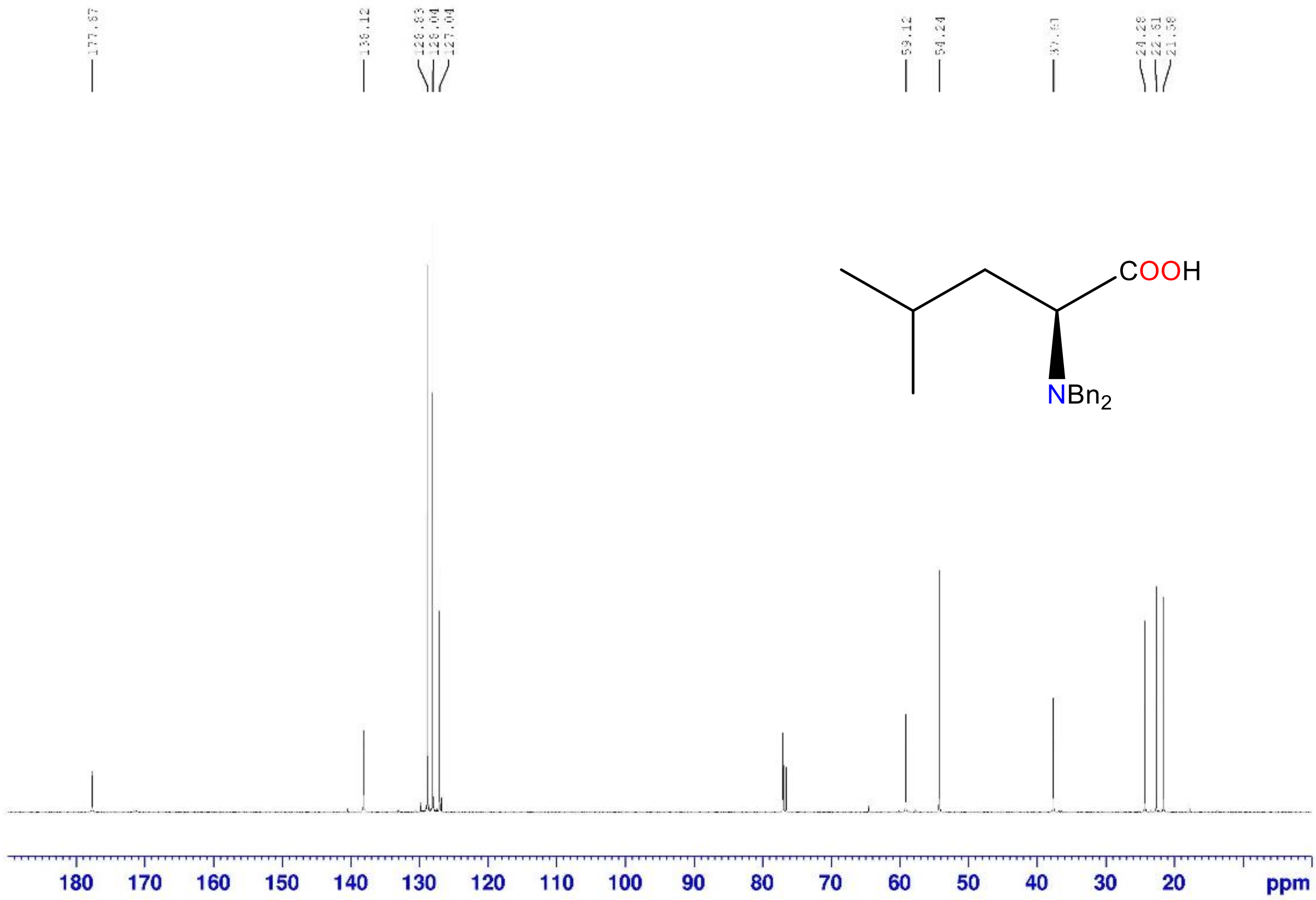

Fig S53 ^{13}C spectrum of *N,N*-dibenzyl-L-leucine (**7d**)

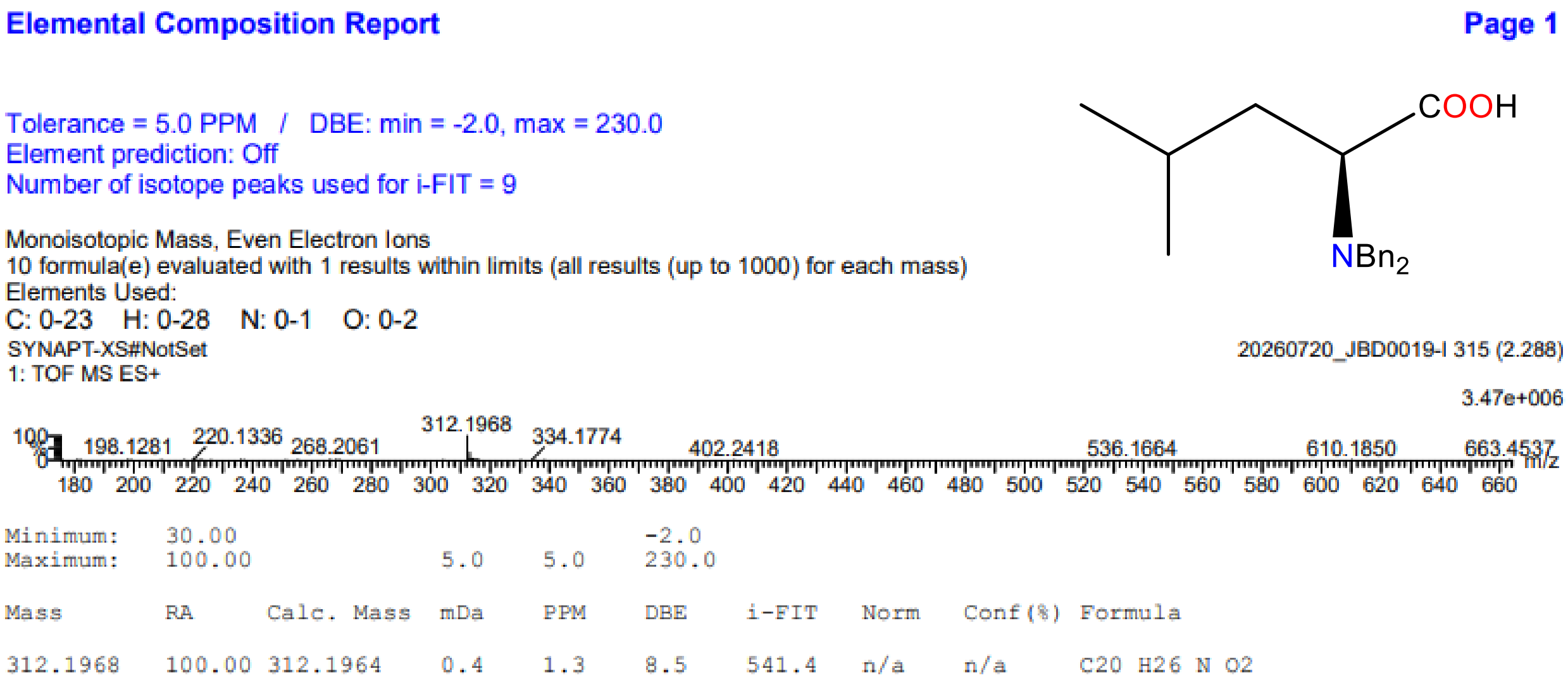

Elemental Composition Report **Page 1**

Tolerance = 5.0 PPM / DBE: min = -2.0, max = 230.0
Element prediction: Off
Number of isotope peaks used for i-FIT = 9

Monoisotopic Mass, Even Electron Ions
10 formula(e) evaluated with 1 results within limits (all results (up to 1000) for each mass)
Elements Used:
C: 0-23 H: 0-28 N: 0-1 O: 0-2
SYNAPT-XS#NotSet
1: TOF MS ES+

Minimum:	30.00				-2.0				
Maximum:	100.00		5.0	5.0	230.0				
Mass	RA	Calc. Mass	mDa	PPM	DBE	i-FIT	Norm	Conf(%)	Formula
312.1968	100.00	312.1964	0.4	1.3	8.5	541.4	n/a	n/a	C20 H26 N O2

Fig S54 HRMS spectrum of *N,N*-dibenzyl-L-leucine (**7d**)

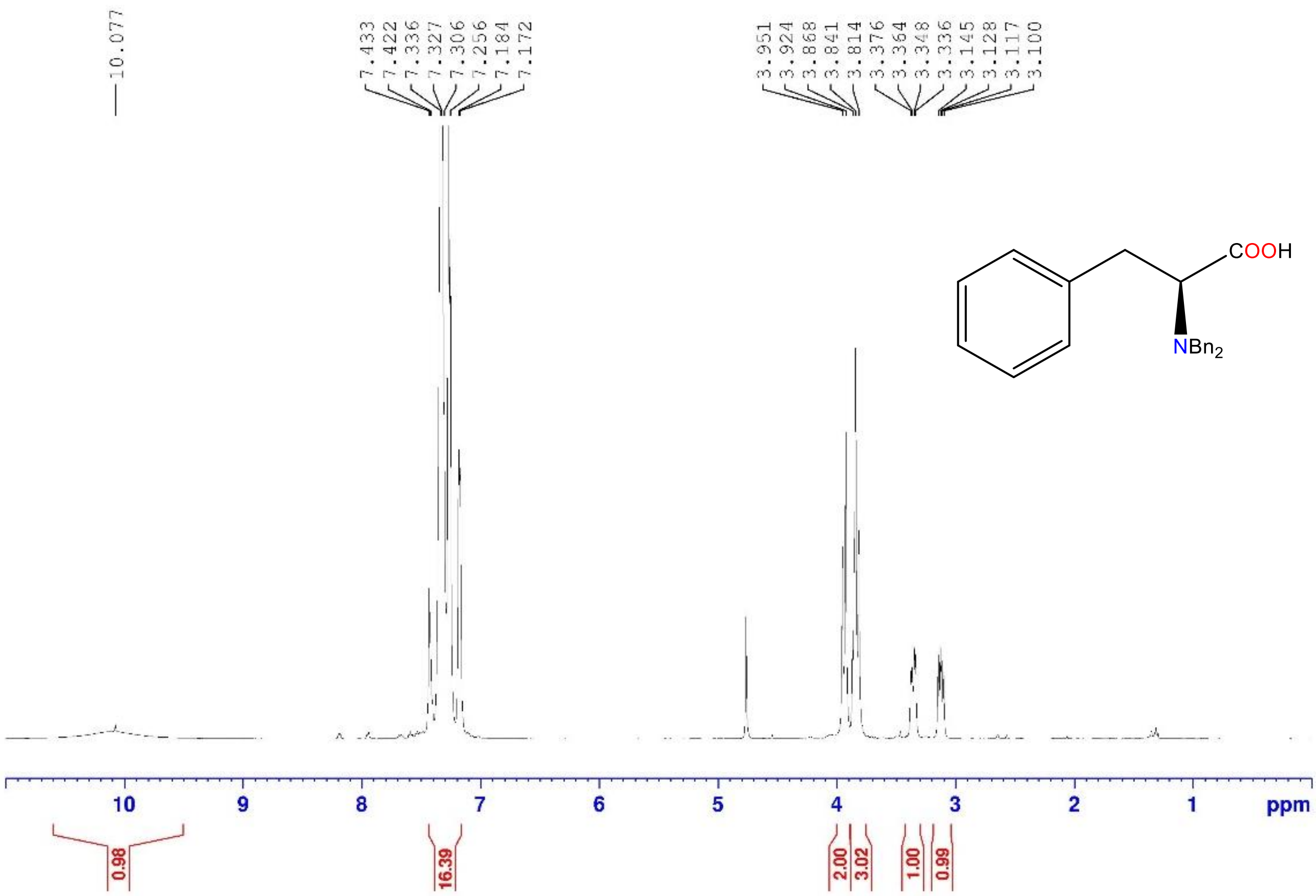

Fig S55 ^{1}H spectrum of *N,N*-dibenzyl-L-phenylalanine (**7e**)

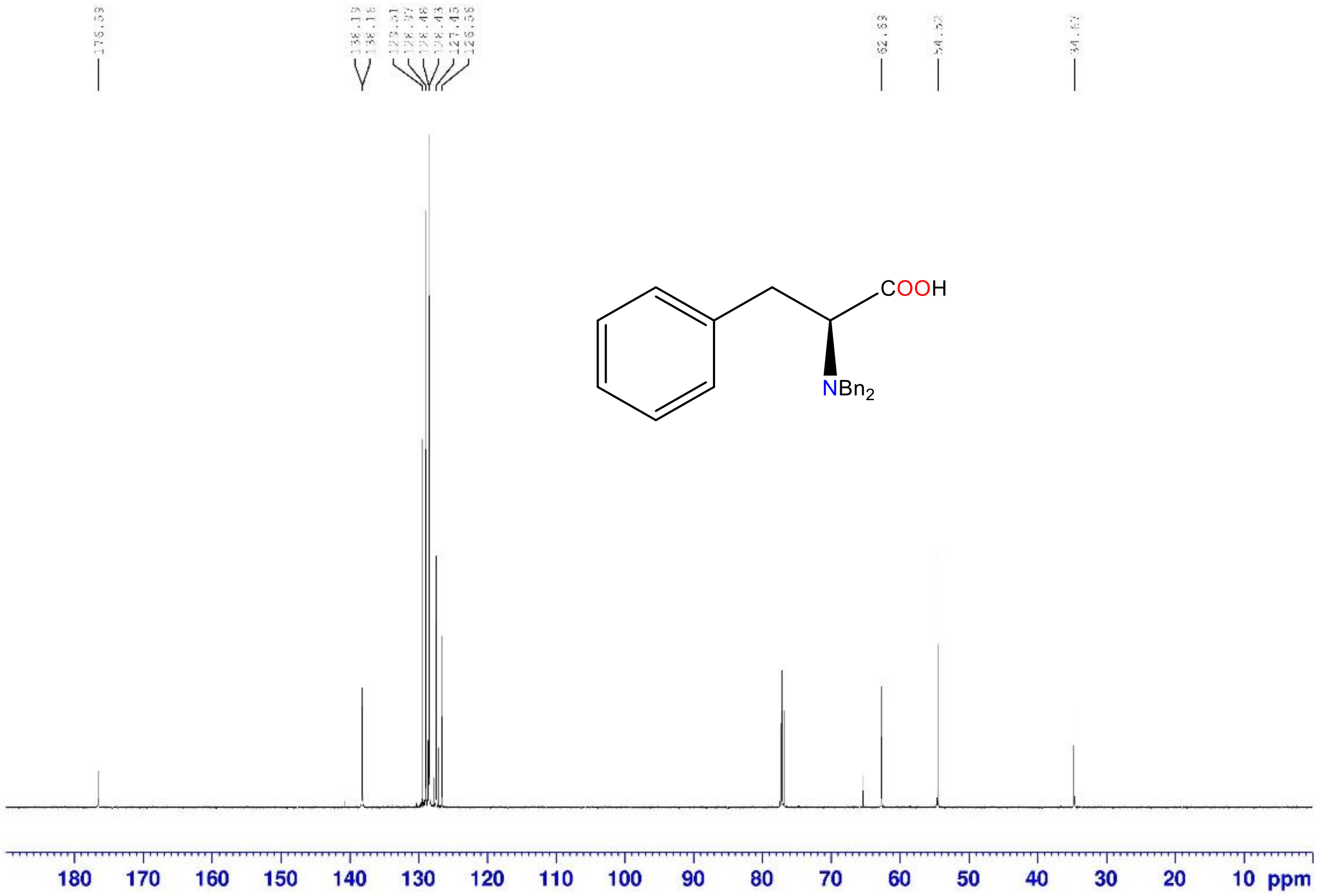

Fig S56 ^{13}C spectrum of *N,N*-dibenzyl-L-phenylalanine (**7e**)

Elemental Composition Report **Page 1**

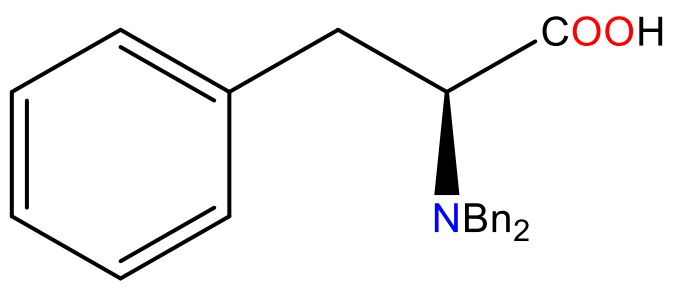

Tolerance = 5.0 PPM / DBE: min = -2.0, max = 230.0
Element prediction: Off
Number of isotope peaks used for i-FIT = 9

Monoisotopic Mass, Even Electron Ions
5 formula(e) evaluated with 1 results within limits (all results (up to 1000) for each mass)
Elements Used:
C: 0-23 H: 0-28 N: 0-1 O: 0-3
SYNAPT-XS#NotSet
1: TOF MS ES+

20260720_JBD0021-I 242 (1.759)
2.54e+006

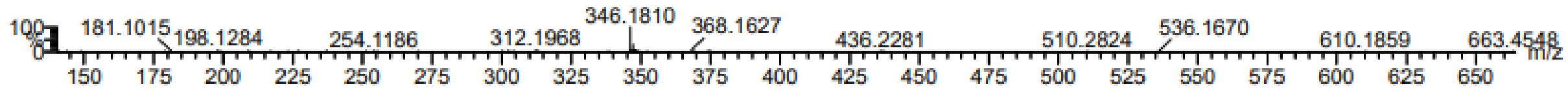

Minimum:	30.00				-2.0				
Maximum:	100.00		5.0	5.0	230.0				
Mass	RA	Calc. Mass	mDa	PPM	DBE	i-FIT	Norm	Conf(%)	Formula
346.1810	100.00	346.1807	0.3	0.9	12.5	363.0	n/a	n/a	C23 H24 N O2

Fig S57 HRMS spectrum of *N,N*-dibenzyl-L-phenylalanine (**7e**)

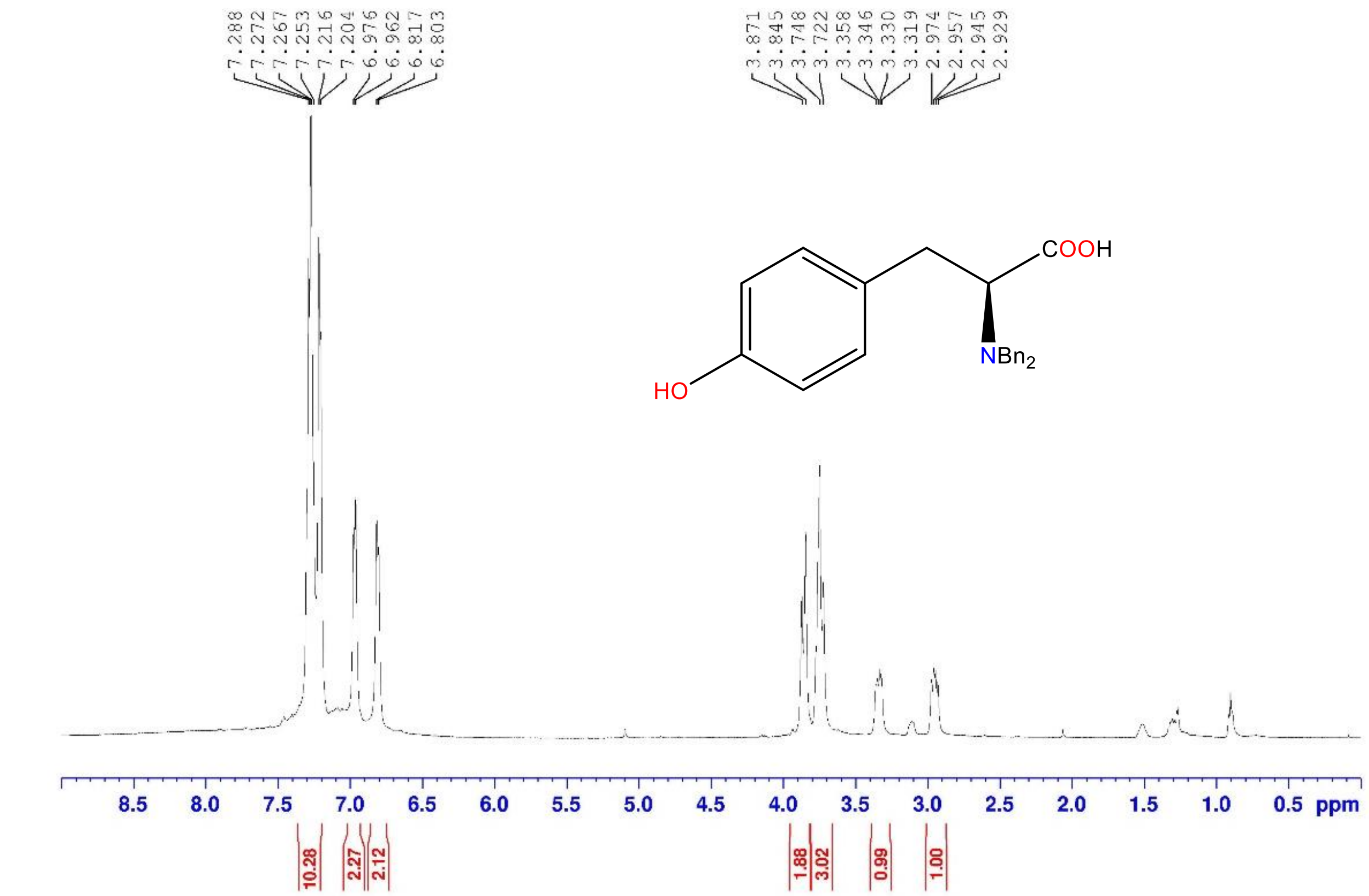

Fig S58 ^{1}H spectrum of *N,N*-dibenzyl-L-tyrosine (**7f**)

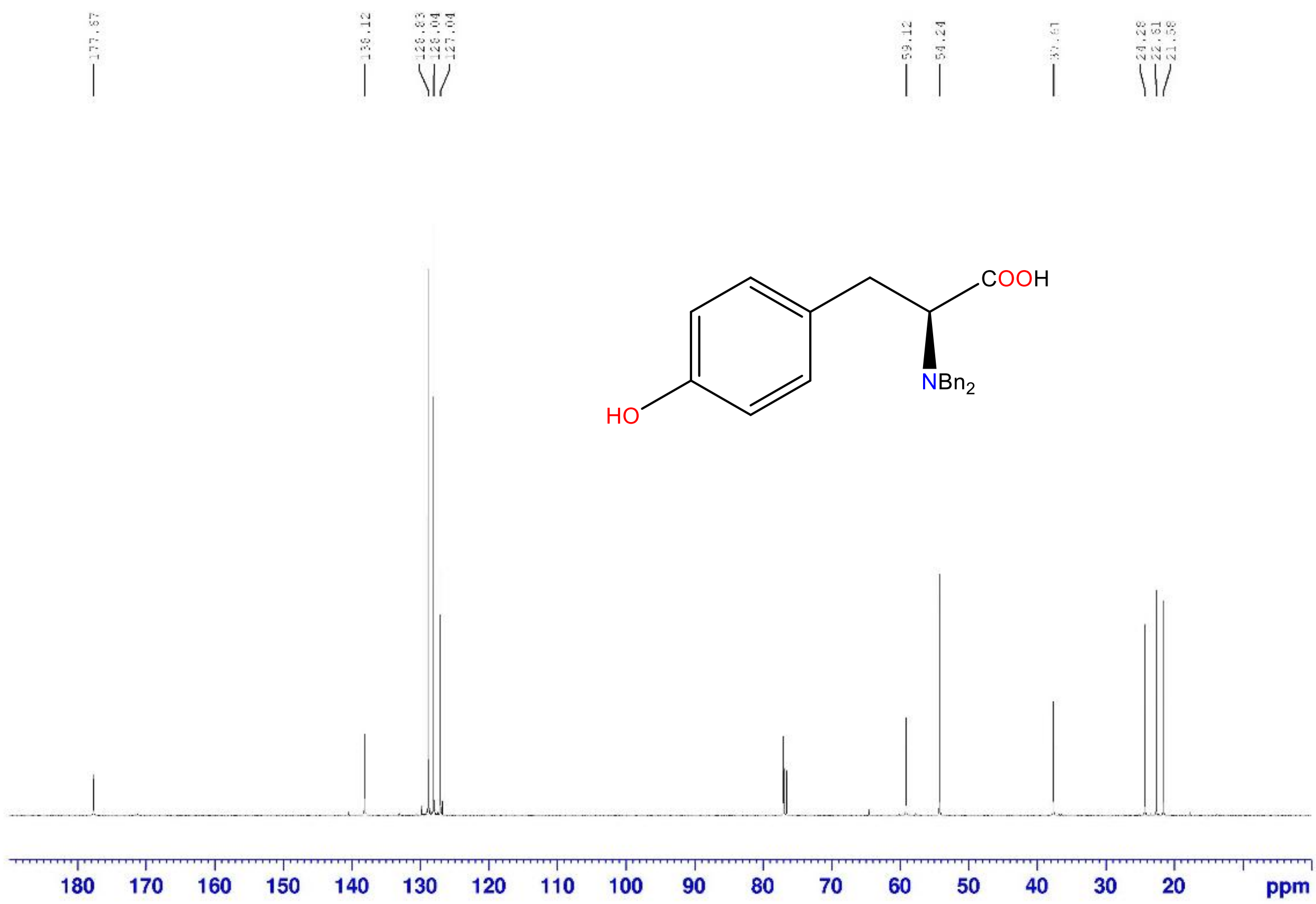

Fig S59 ^{13}C spectrum of *N,N*-dibenzyl-L-tyrosine (**7f**)

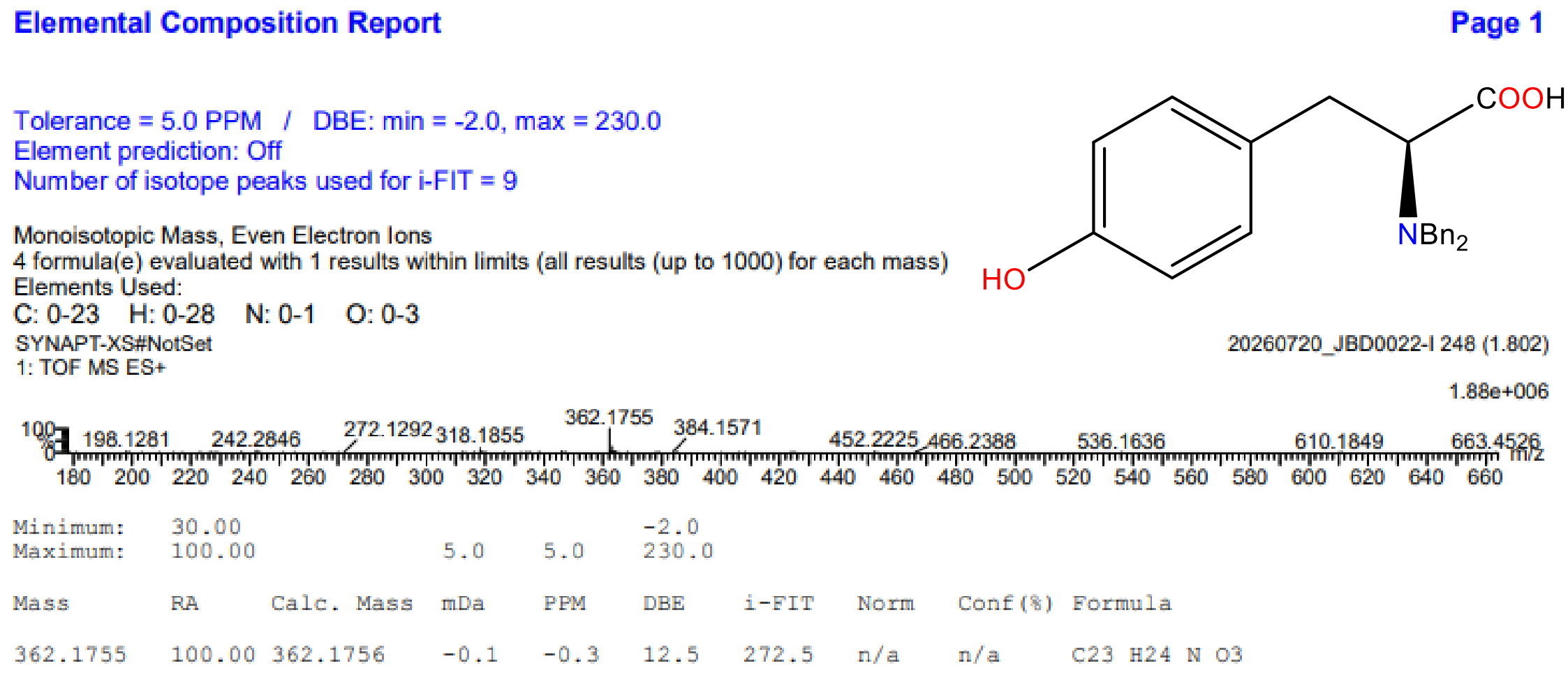

Elemental Composition Report Page 1

Tolerance = 5.0 PPM / DBE: min = -2.0, max = 230.0
Element prediction: Off
Number of isotope peaks used for i-FIT = 9

Monoisotopic Mass, Even Electron Ions
4 formula(e) evaluated with 1 results within limits (all results (up to 1000) for each mass)
Elements Used:
C: 0-23 H: 0-28 N: 0-1 O: 0-3
SYNAPT-XS#NotSet
1: TOF MS ES+

20260720_JBD0022-I 248 (1.802)

1.88e+006

Minimum:	30.00				-2.0				
Maximum:	100.00		5.0	5.0	230.0				
Mass	RA	Calc. Mass	mDa	PPM	DBE	i-FIT	Norm	Conf(%)	Formula
362.1755	100.00	362.1756	-0.1	-0.3	12.5	272.5	n/a	n/a	C23 H24 N O3

Fig S60 HRMS spectrum of *N,N*-dibenzyl-L-tyrosine (**7f**)

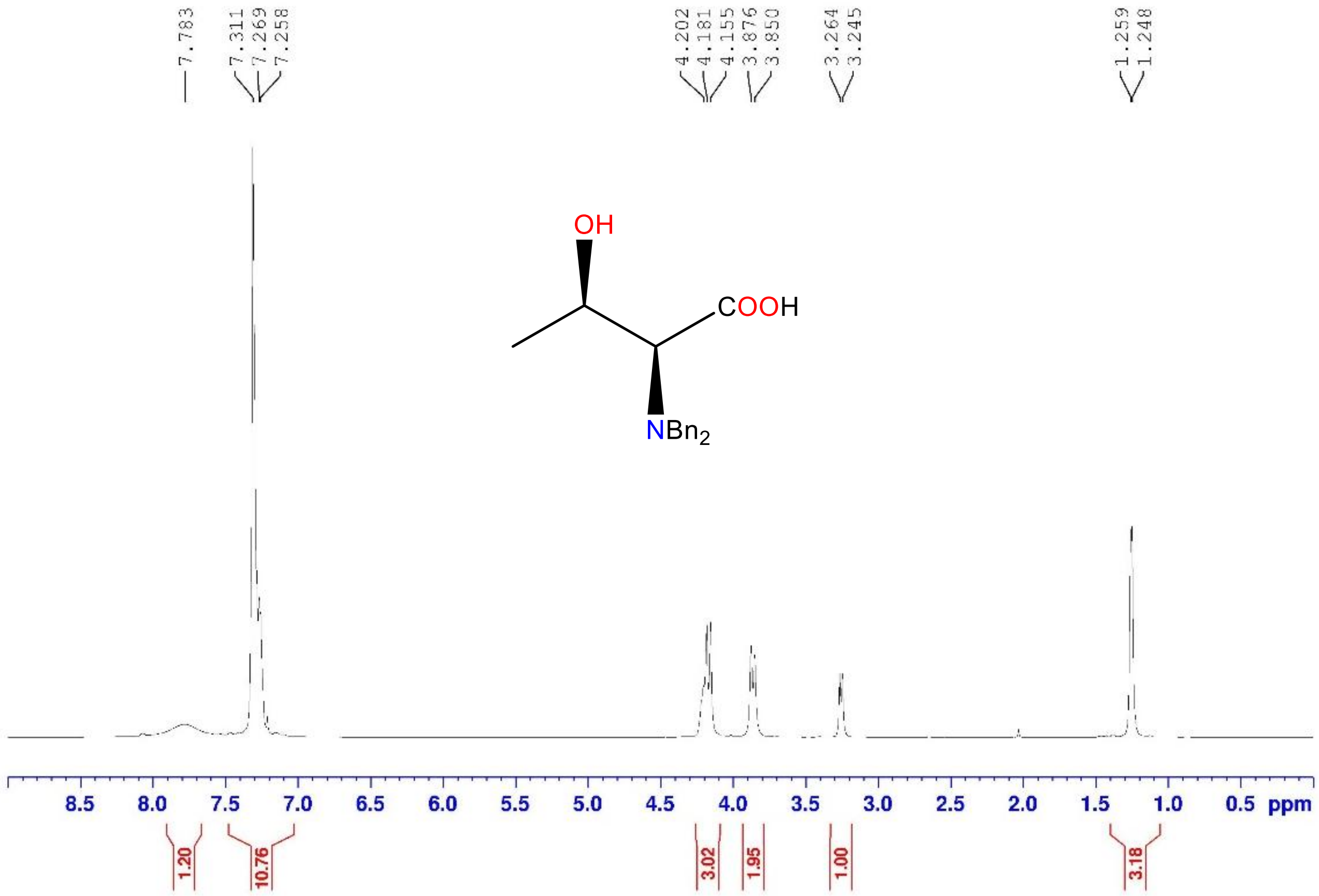

Fig S61 ^{1}H spectrum of *N,N*-dibenzyl-L-threonine (**7g**)

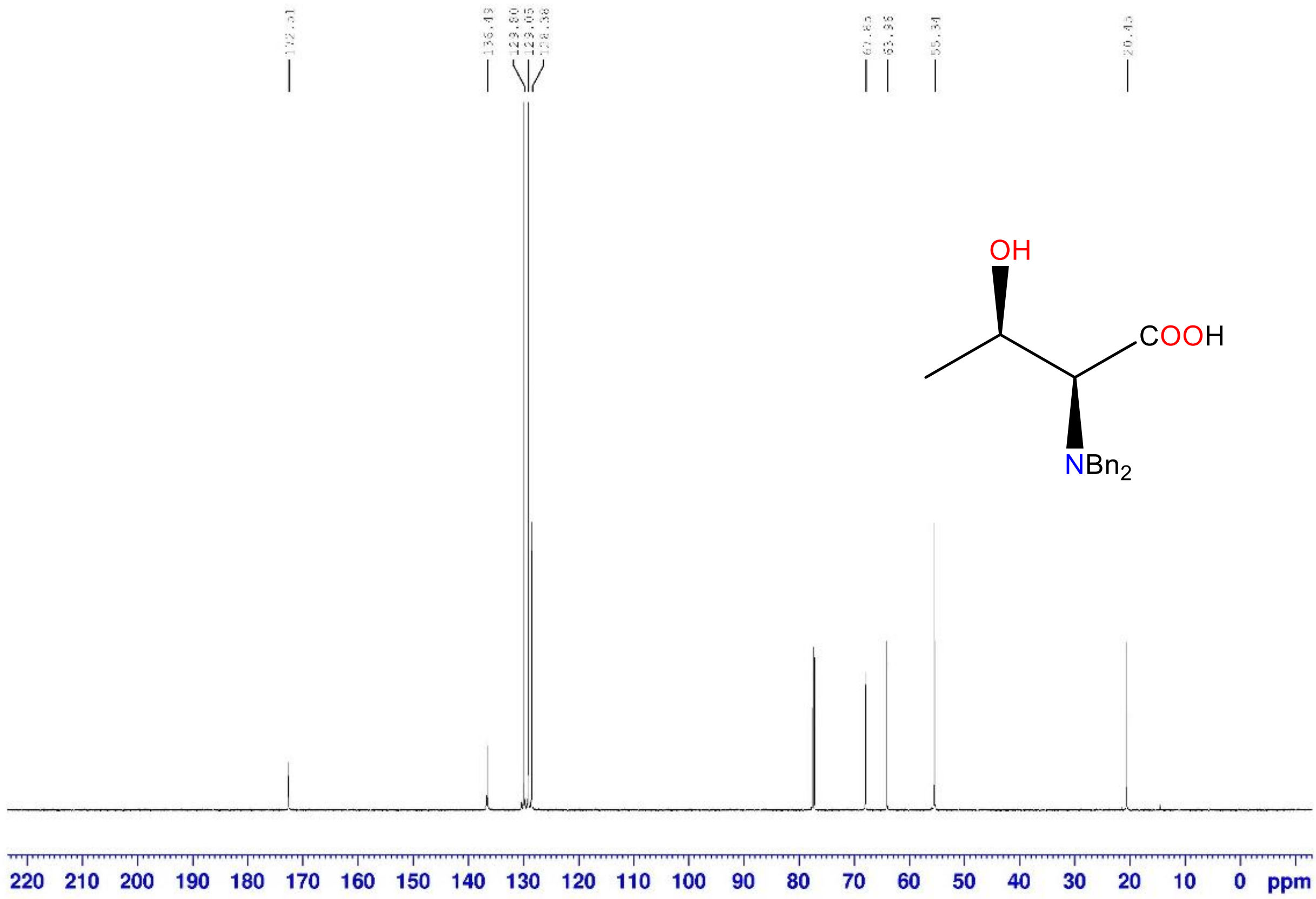

Fig S62 ^{13}C spectrum of *N,N*-dibenzyl-L-threonine (**7g**)

Elemental Composition Report **Page 1**

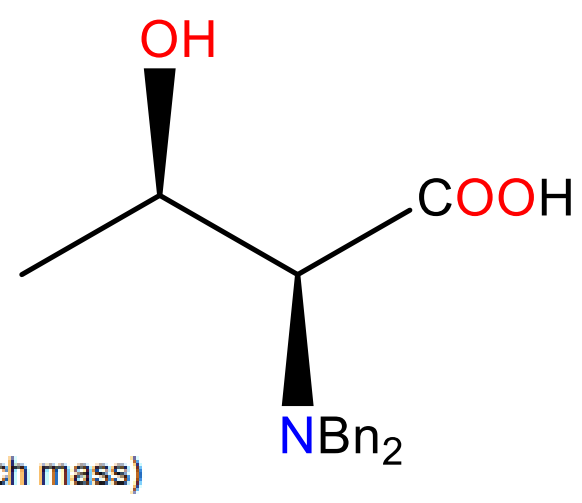

Tolerance = 5.0 PPM / DBE: min = -2.0, max = 230.0
Element prediction: Off
Number of isotope peaks used for i-FIT = 9

Monoisotopic Mass, Even Electron Ions
26 formula(e) evaluated with 1 results within limits (all results (up to 1000) for each mass)
Elements Used:
C: 0-25 H: 0-30 N: 0-1 O: 0-4
SYNAPT-XS#NotSet 20260720_JBD0023-I 312 (2.267)
1: TOF MS ES+
2.79e+006

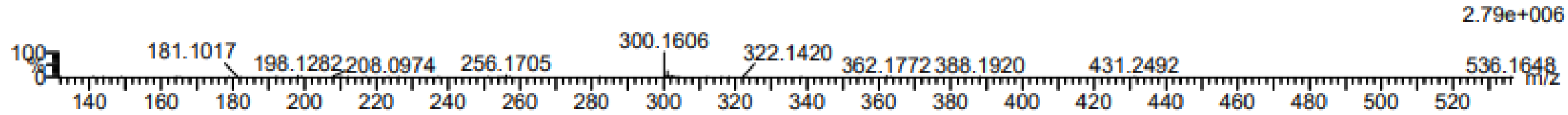

Minimum:	30.00				-2.0				
Maximum:	100.00		5.0	5.0	230.0				
Mass	RA	Calc. Mass	mDa	PPM	DBE	i-FIT	Norm	Conf(%)	Formula
300.1606	100.00	300.1600	0.6	2.0	8.5	350.0	n/a	n/a	C18 H22 N O3

Fig S63 HRMS spectrum of *N,N*-dibenzyl-L-threonine (**7g**)

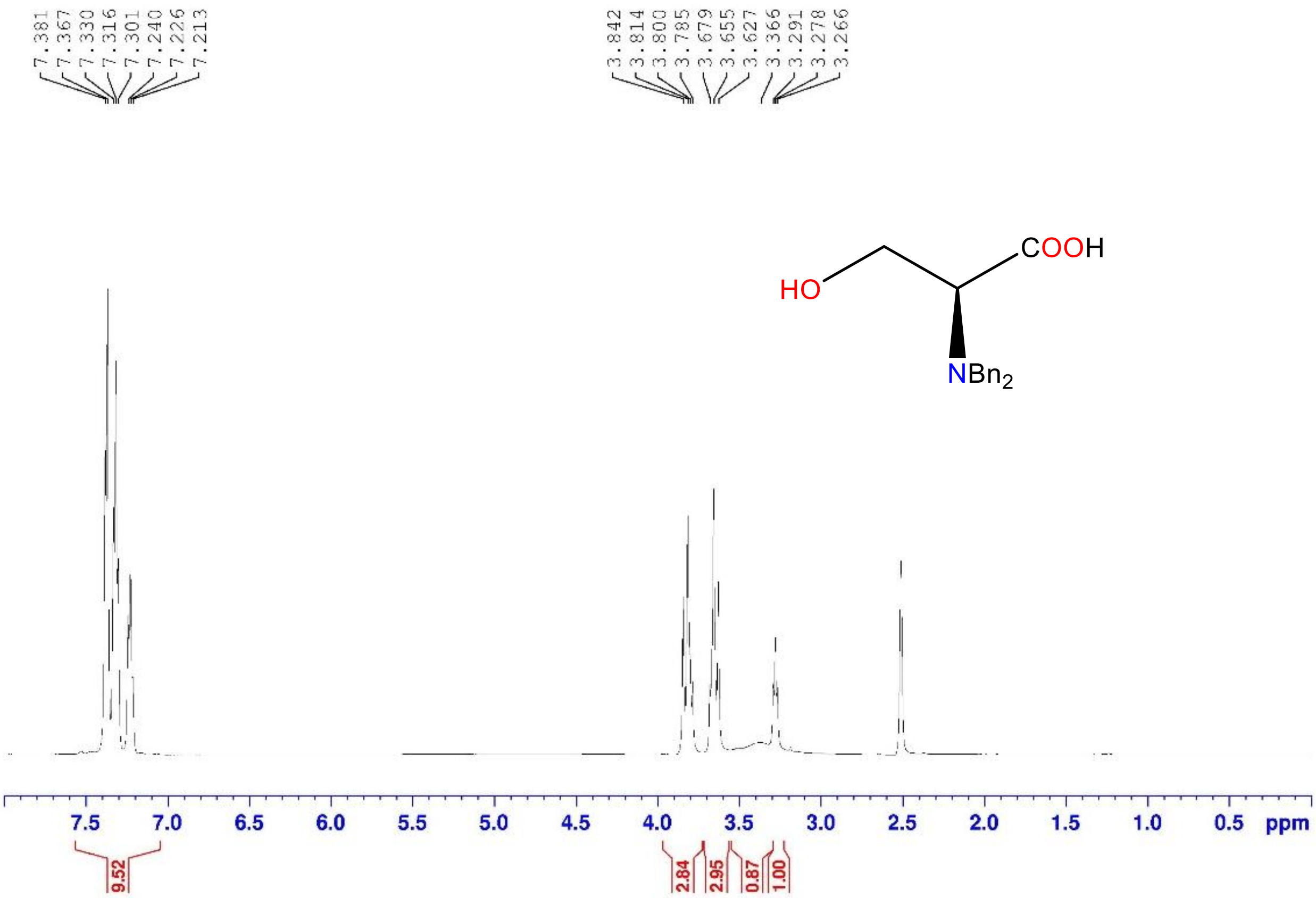

Fig S64 ^{1}H spectrum of *N,N*-dibenzyl-L-serine (**7h**)

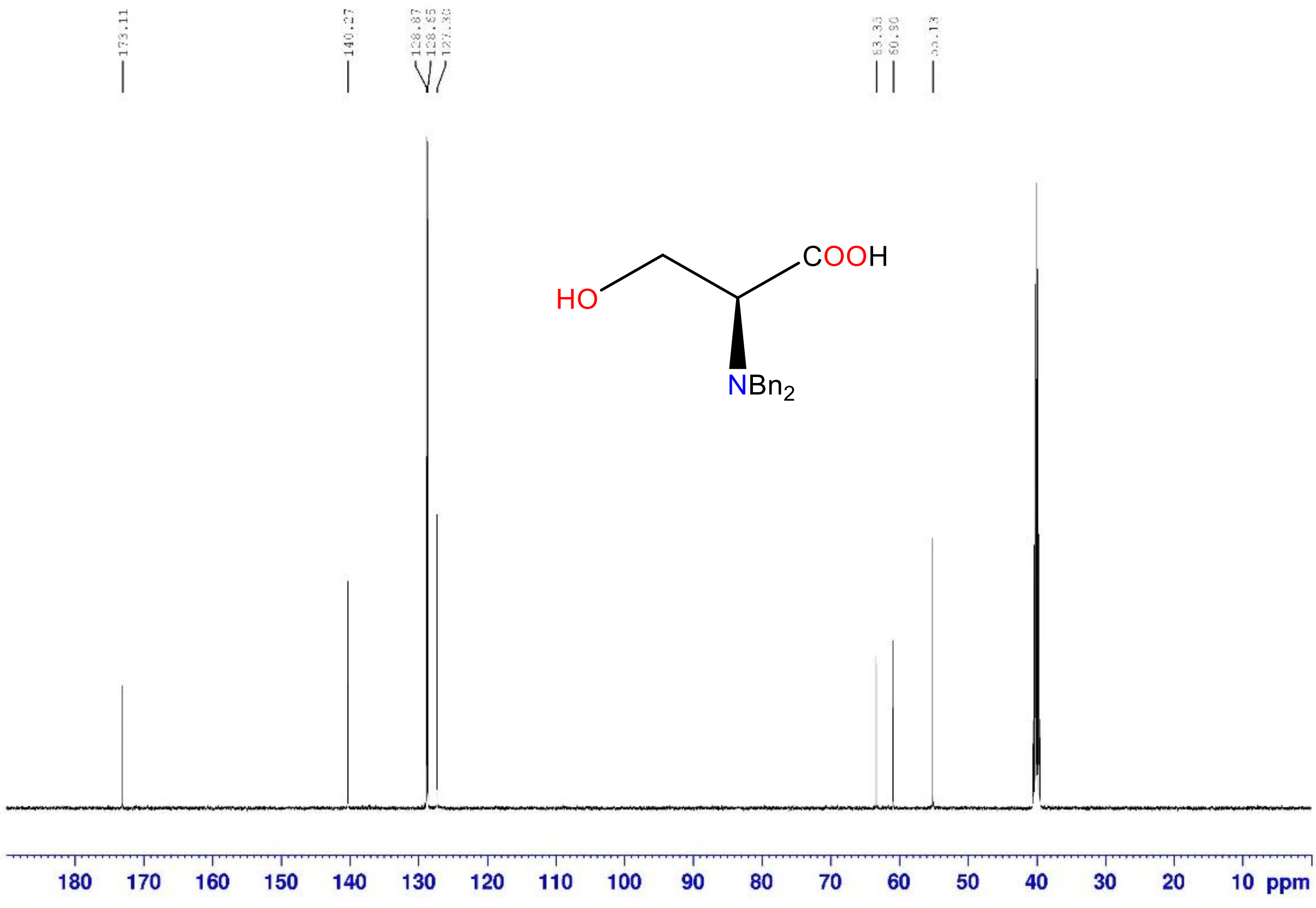

Fig S65 ^{13}C spectrum of *N,N*-dibenzyl-L-serine (**7h**)

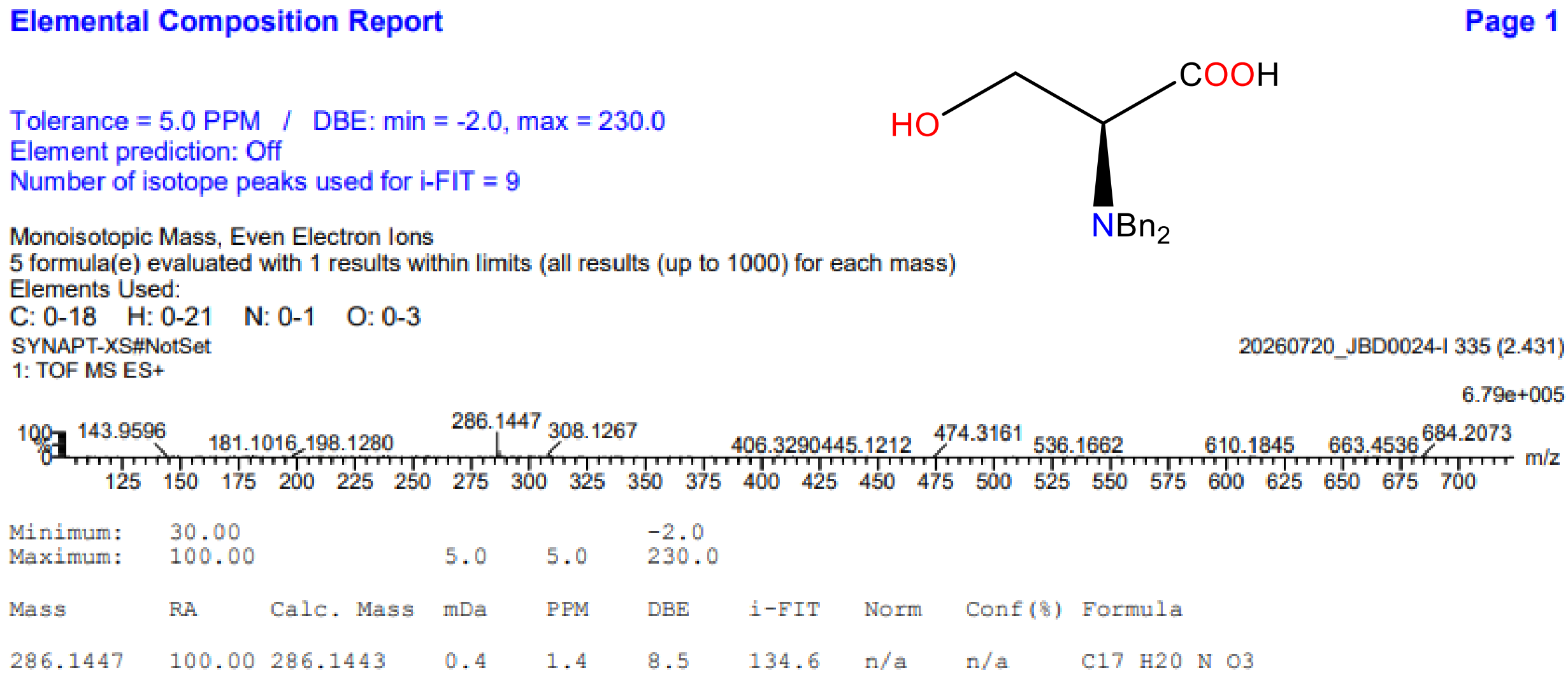

Fig S66 HRMS spectrum of *N,N*-dibenzyl-L-serine (**7h**)

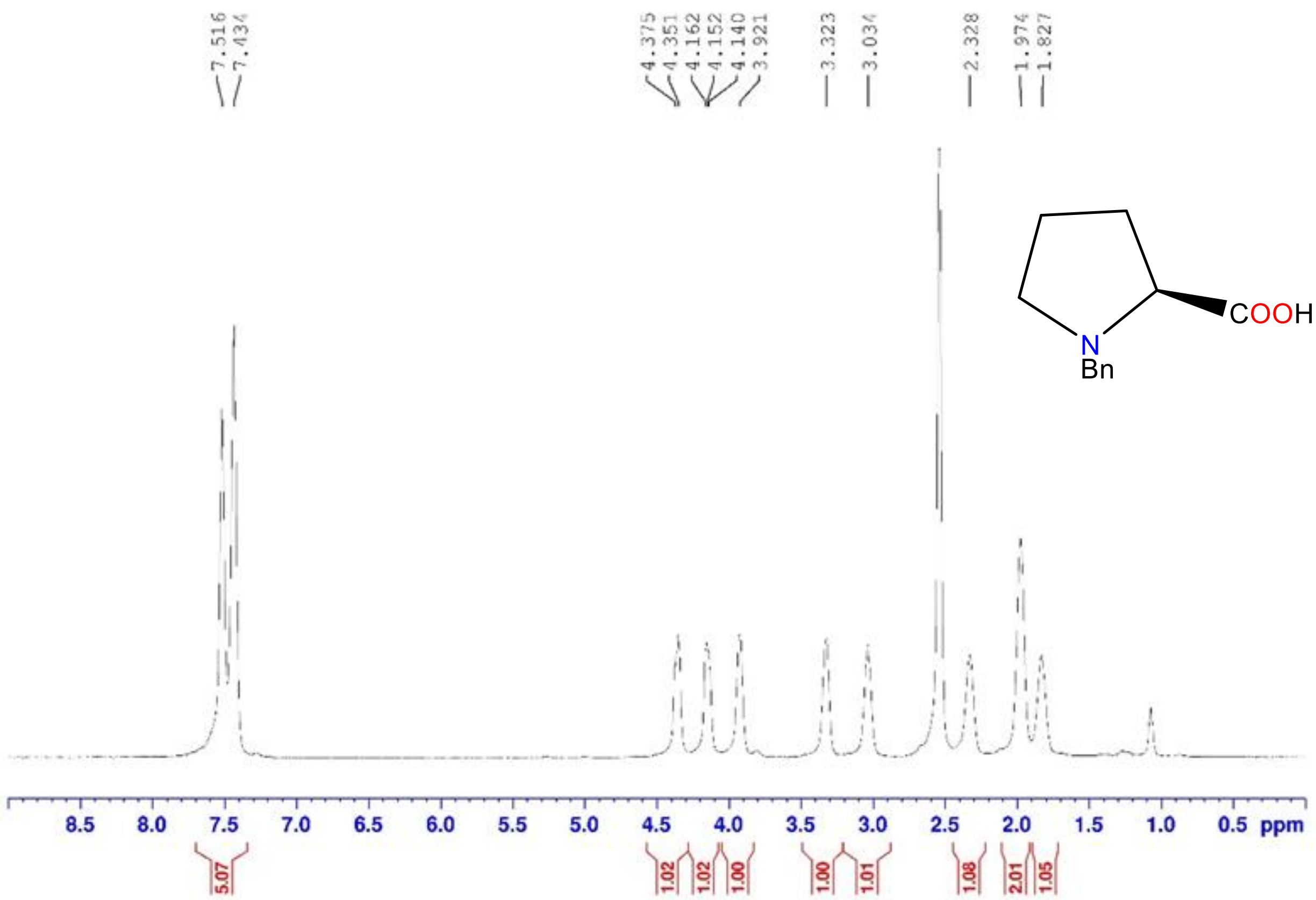

Fig S67 ^{1}H spectrum of *N*-benzyl-L-proline (**7i**)

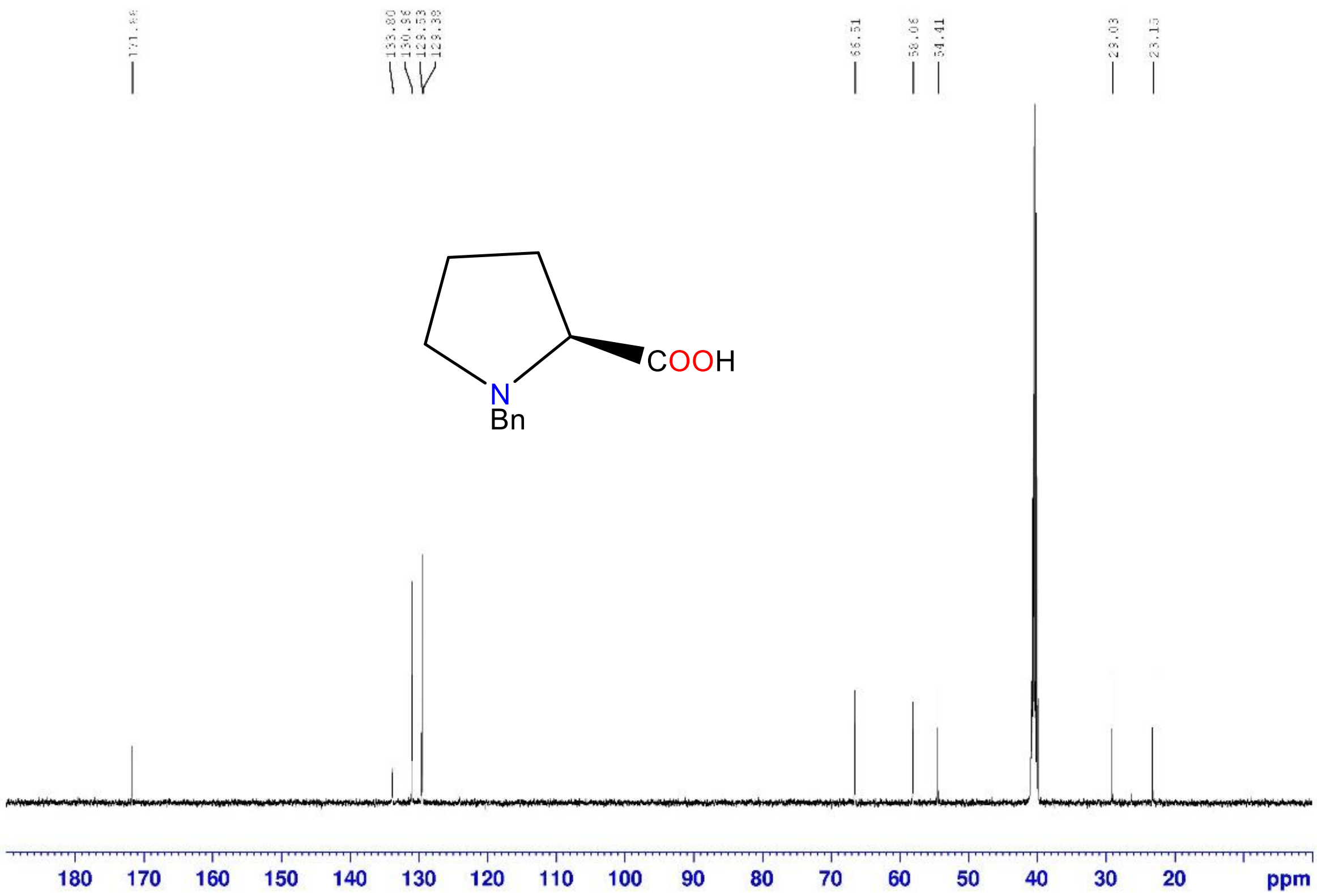

Fig S68 ^{13}C spectrum of *N*-benzyl-L-proline (**7i**)

Elemental Composition Report **Page 1**

Tolerance = 5.0 PPM / DBE: min = -2.0, max = 230.0
Element prediction: Off
Number of isotope peaks used for i-FIT = 9

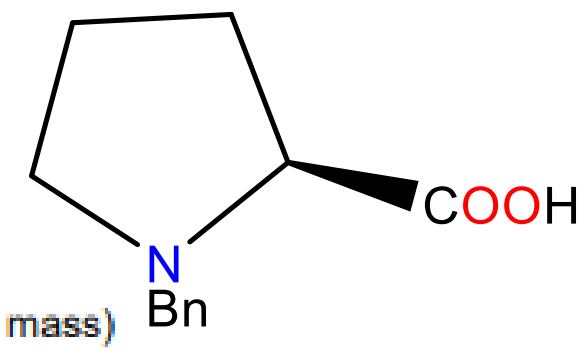

Monoisotopic Mass, Even Electron Ions
26 formula(e) evaluated with 1 results within limits (all results (up to 1000) for each mass)
Elements Used:
C: 0-25 H: 0-30 N: 0-1 O: 0-4

SYNAPT-XS#NotSet 20260720_JBD0025-I 40 (0.306)
1: TOF MS ES+

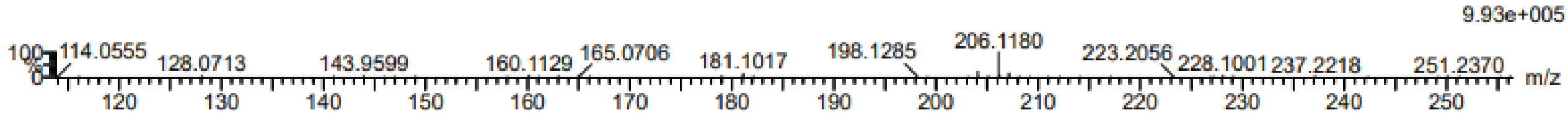

Minimum:		30.00			-2.0				
Maximum:		100.00	5.0	5.0	230.0				

Mass	RA	Calc. Mass	mDa	PPM	DBE	i-FIT	Norm	Conf(%)	Formula
206.1180	100.00	206.1181	-0.1	-0.5	5.5	150.2	n/a	n/a	C12 H16 N O2

Fig S69 HRMS spectrum of *N*-benzyl-L-proline (**7i**)

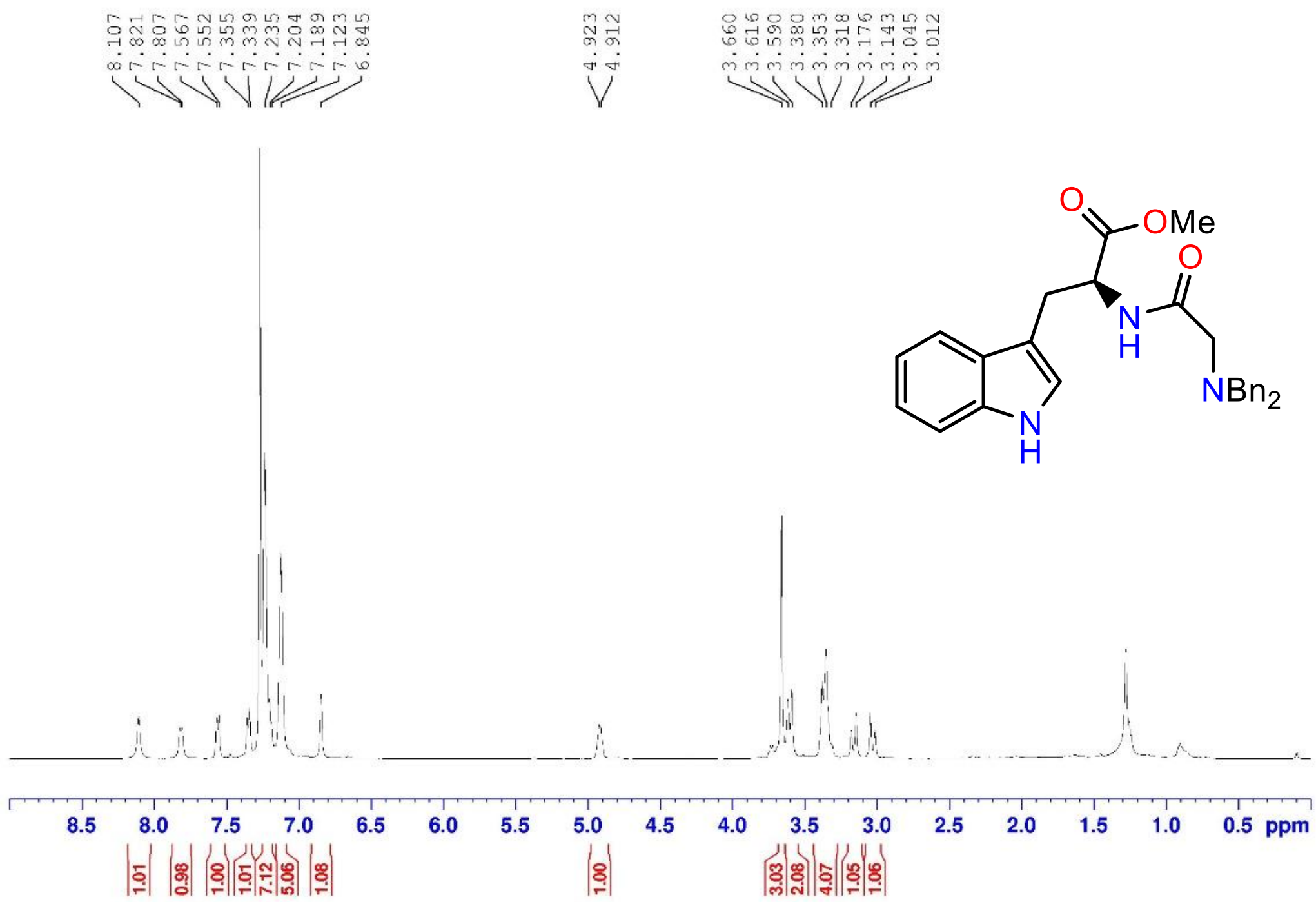

Fig S70 ^{1}H spectrum of methyl dibenzylglycyl-L-tryptophanate (**8a**)

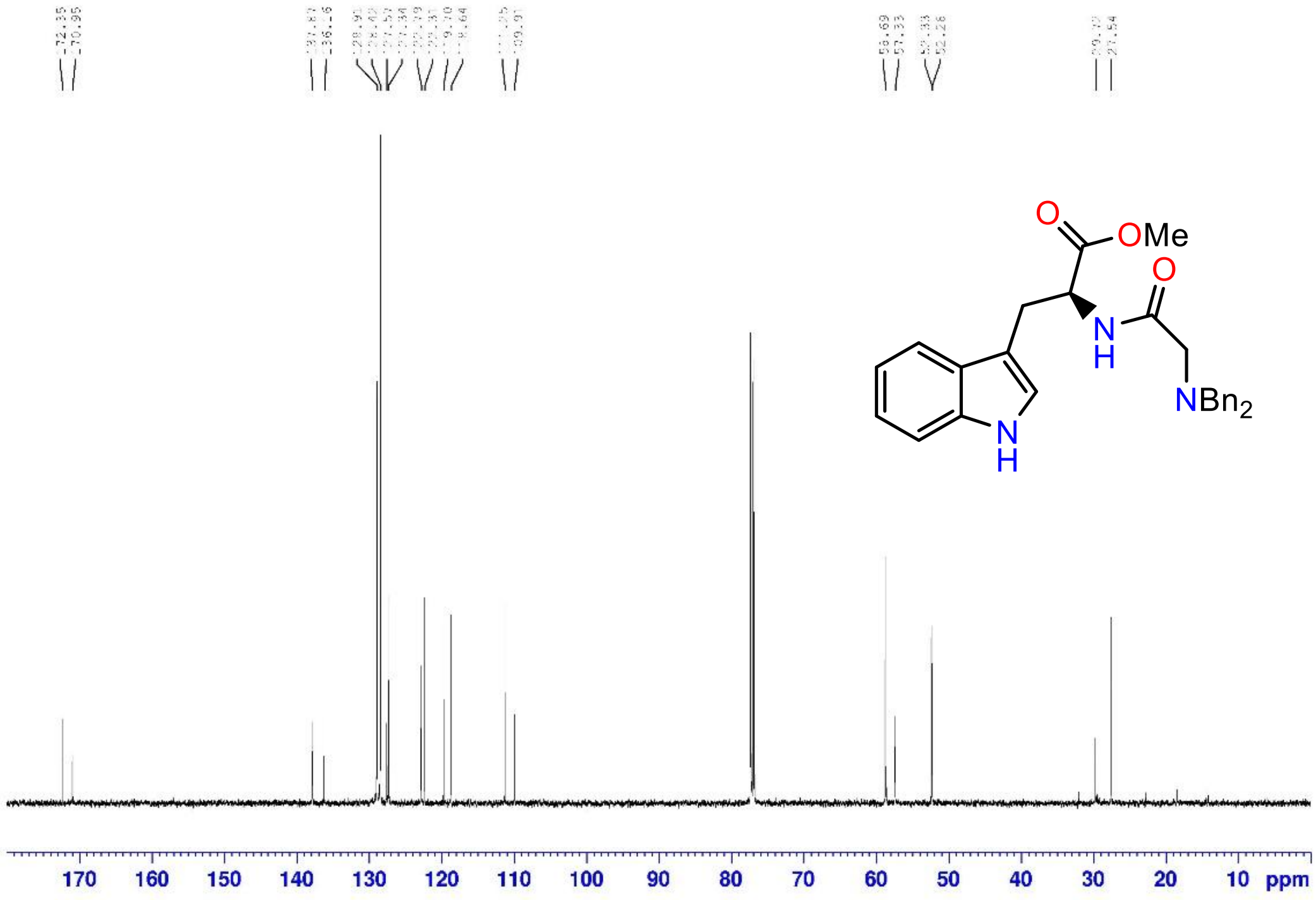

Fig S71 ^{13}C spectrum of methyl dibenzylglycyl-L-tryptophanate (**8a**)

Vion IMS Qtof (Waters)

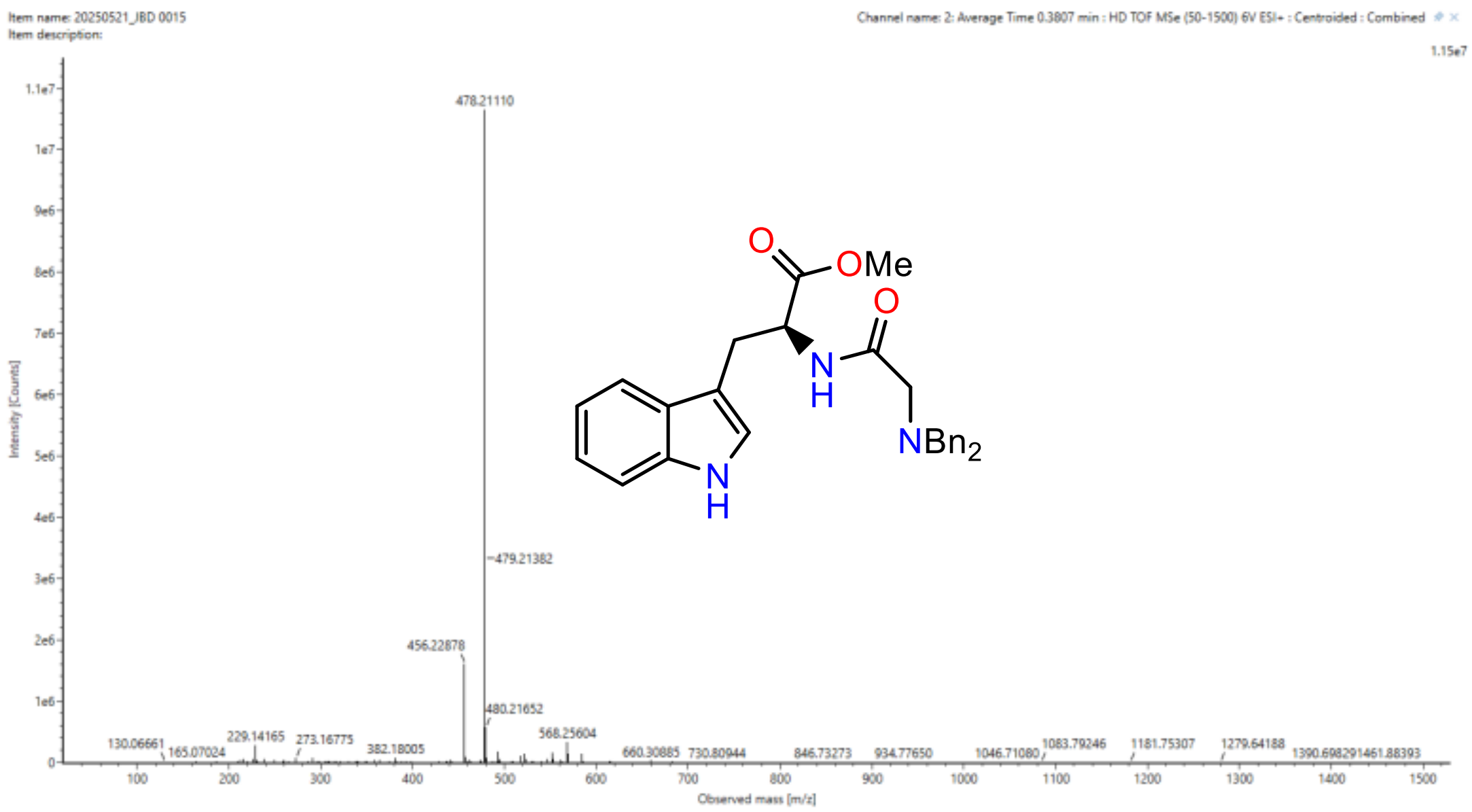

Fig S72 HRMS spectrum of methyl dibenzylglycyl-L-tryptophanate (**8a**)

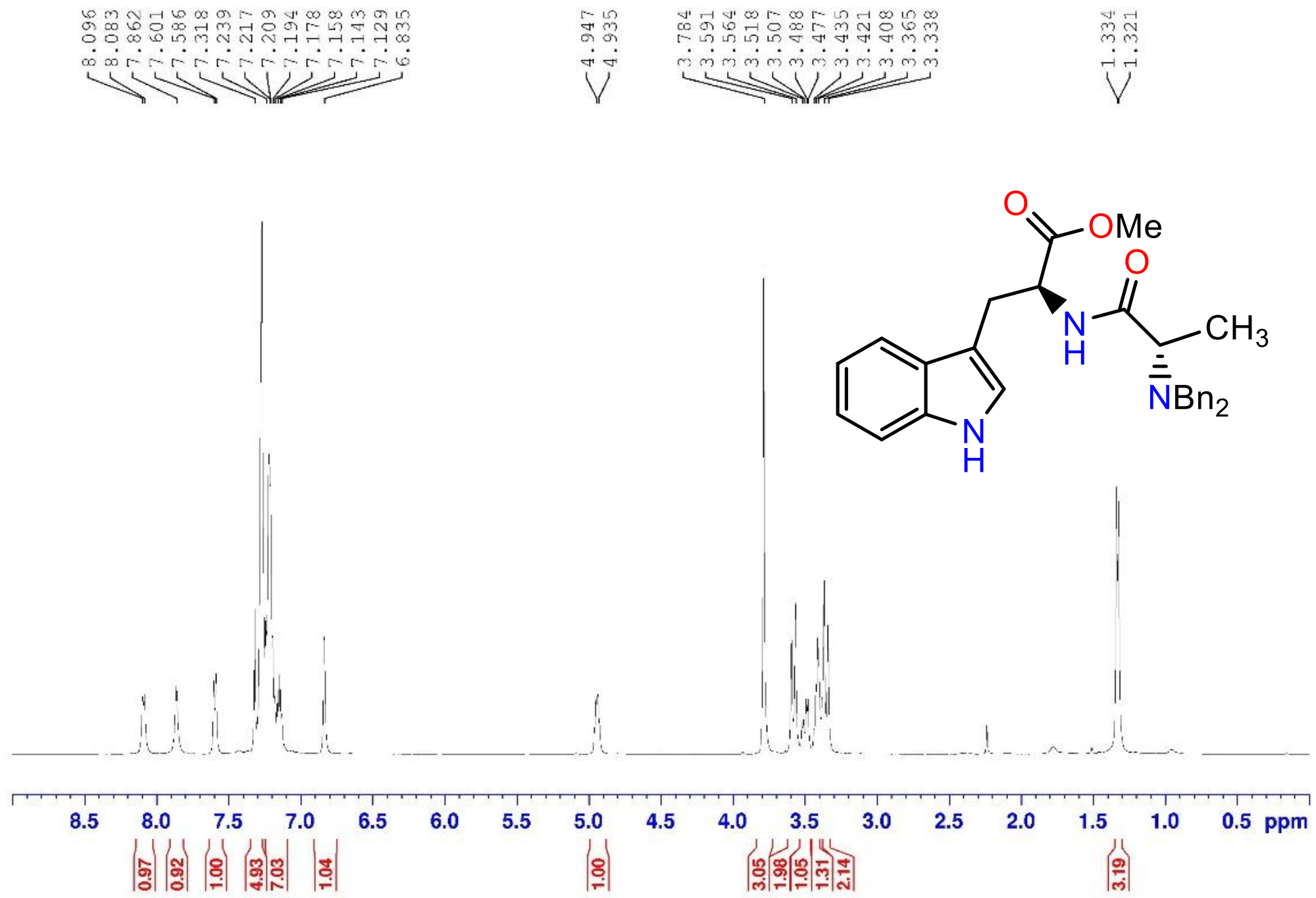

Fig S73 ^{1}H spectrum of methyl dibenzyl-L-alanyl-L-tryptophanate (**8b**)

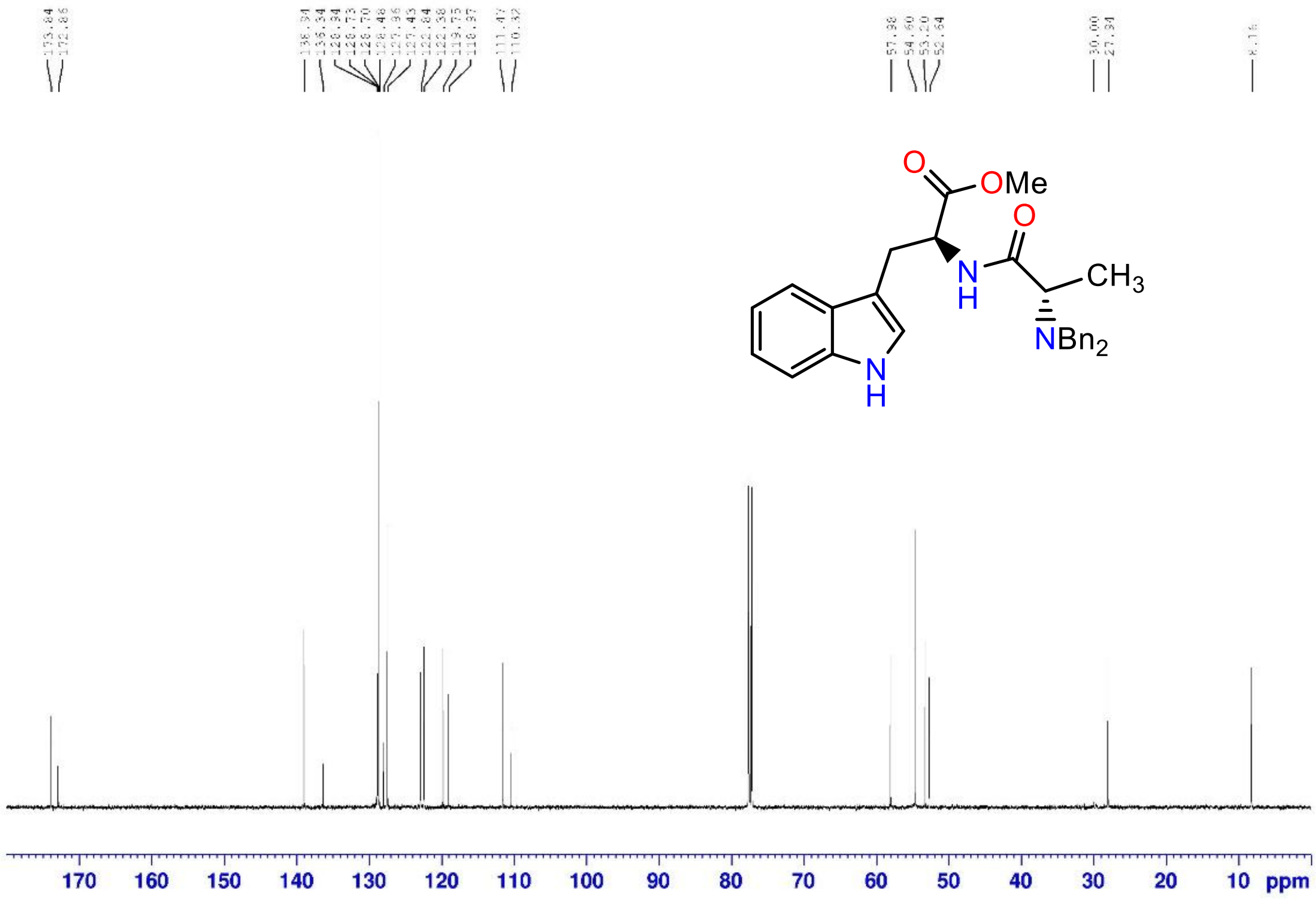

Fig S74 ^{13}C spectrum of methyl dibenzyl-L-alanyl-L-tryptophanate (**8b**)

Vion IMS Qtof (Waters)

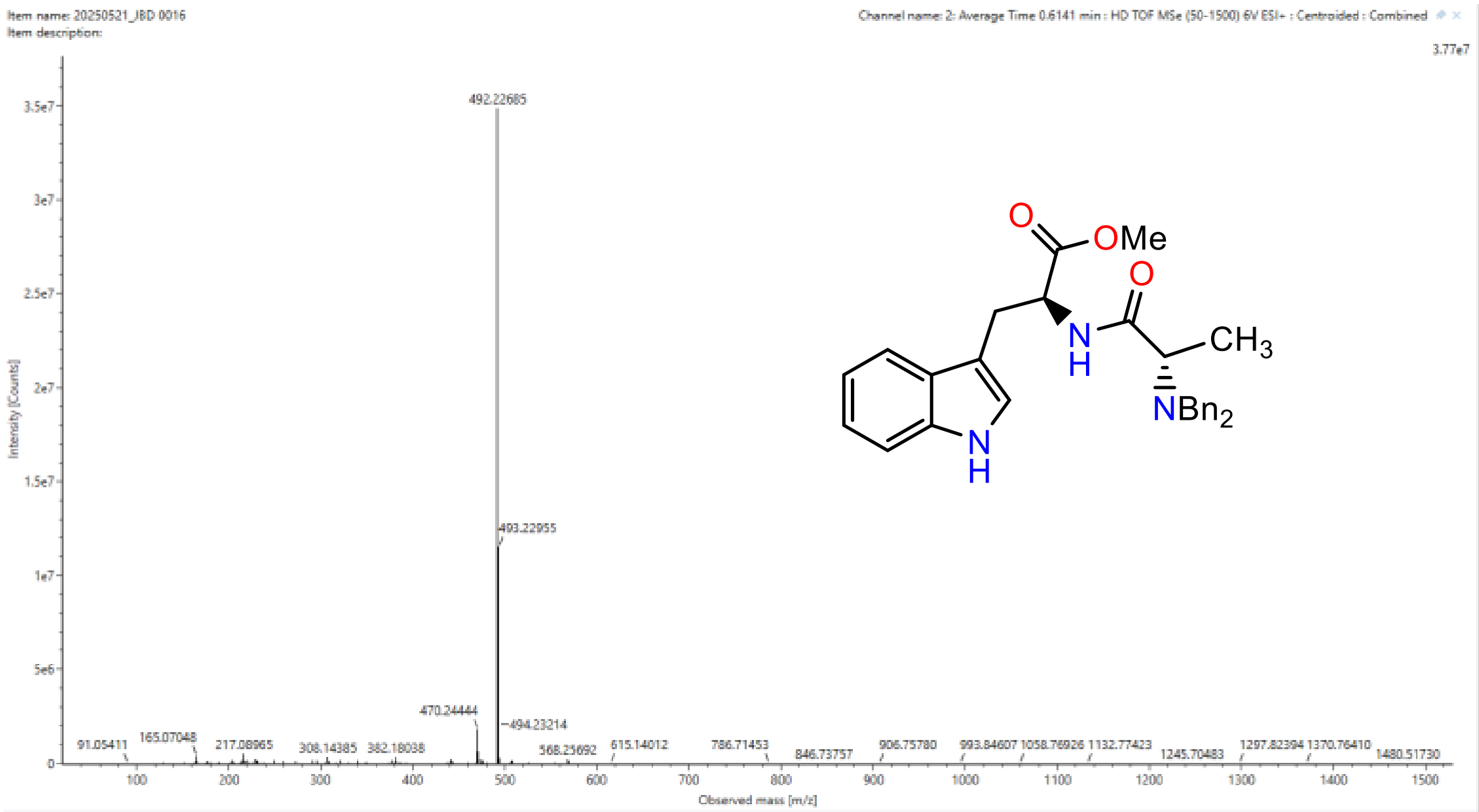

Fig S75 HRMS spectrum of methyl dibenzyl-L-alanyl-L-tryptophanate (**8b**)

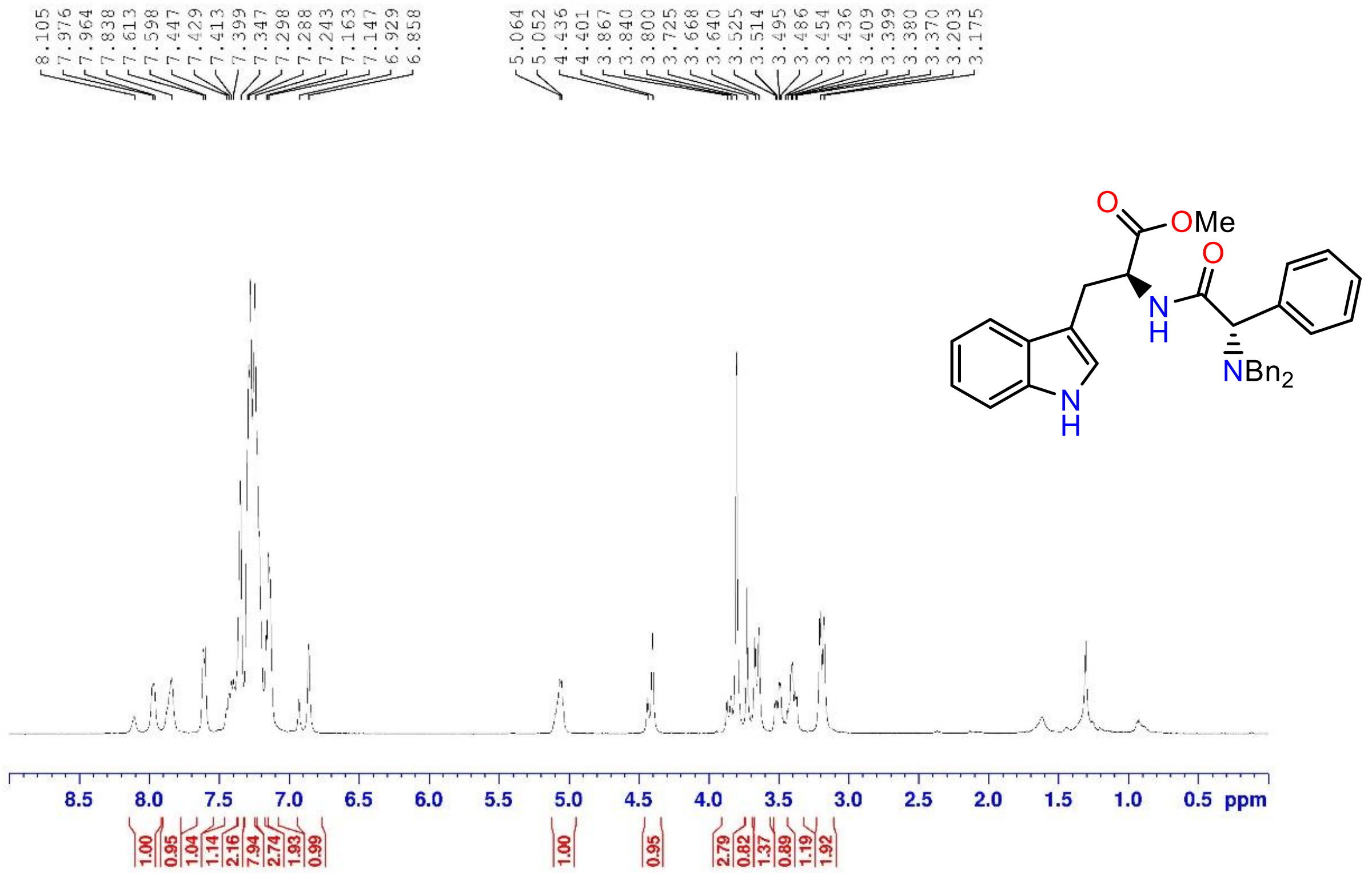

Fig S76 ^{1}H spectrum of methyl ((S)-2-(dibenzylamino)-2-phenylacetyl)-L-tryptophanate (**8c**)

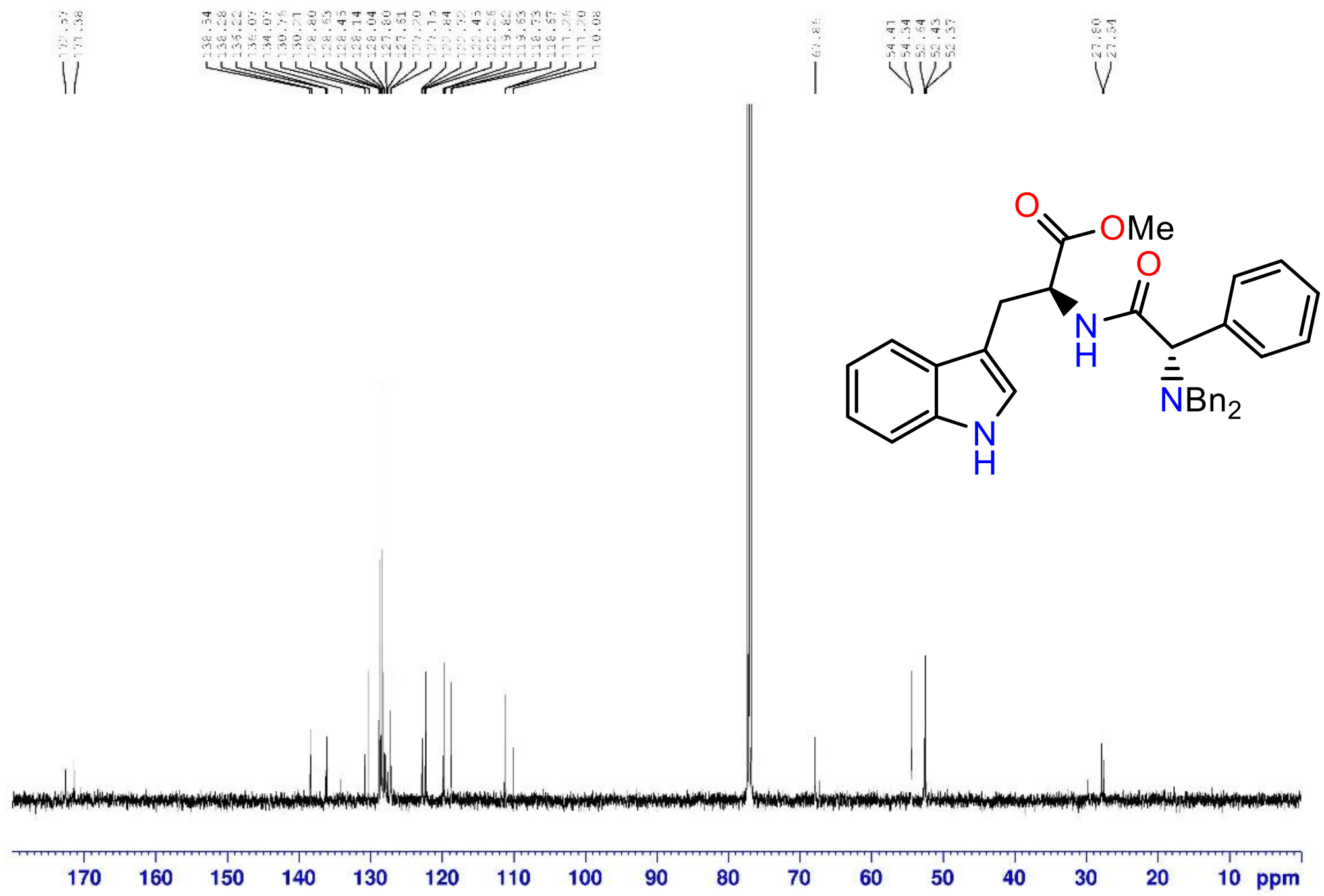

Fig S77 ^{13}C spectrum of methyl ((S)-2-(dibenzylamino)-2-phenylacetyl)-L-tryptophanate (**8c**)

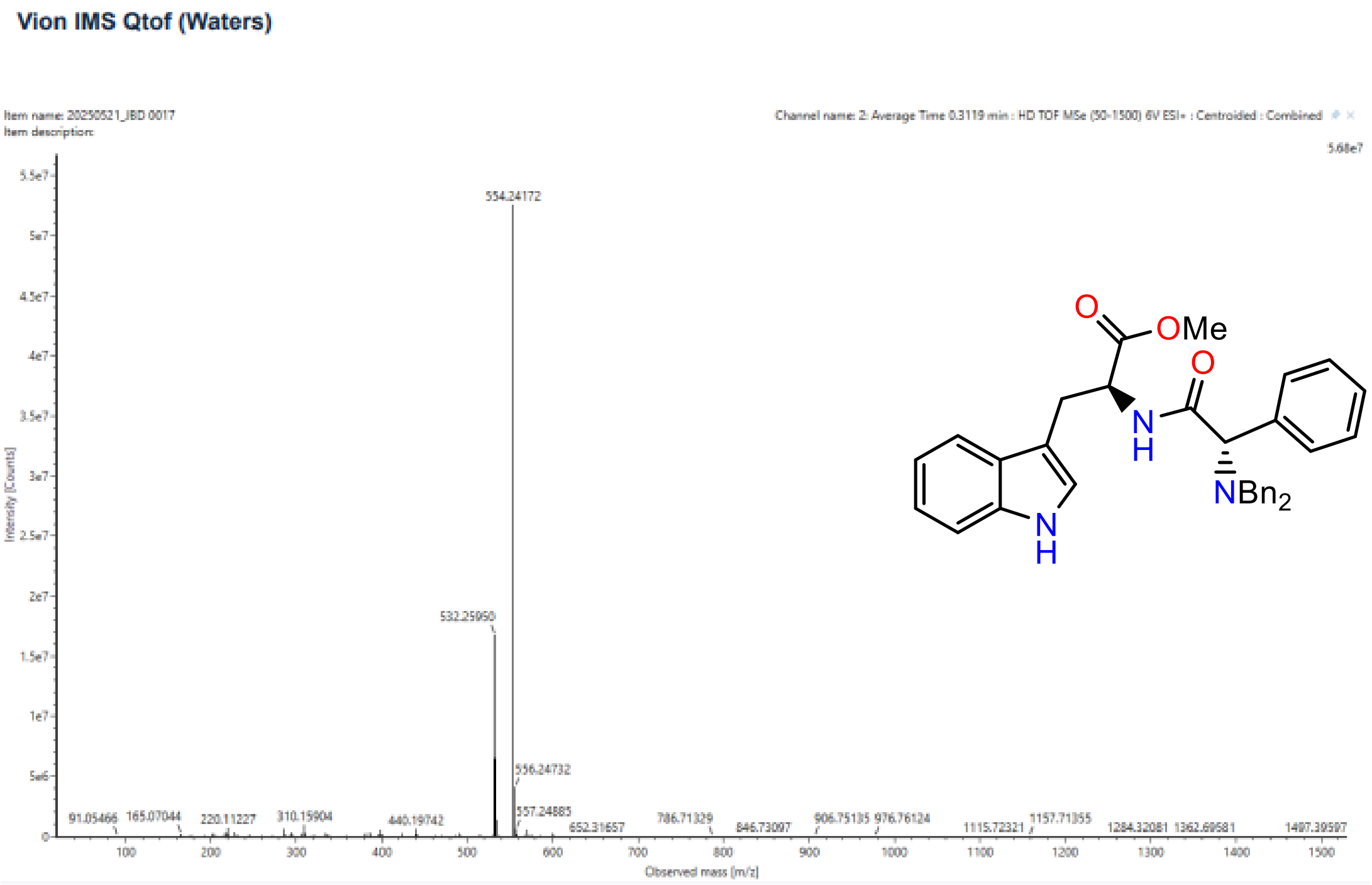

Fig S78 HRMS spectrum of methyl ((S)-2-(dibenzylamino)-2-phenylacetyl)-L-tryptophanate (**8c**)

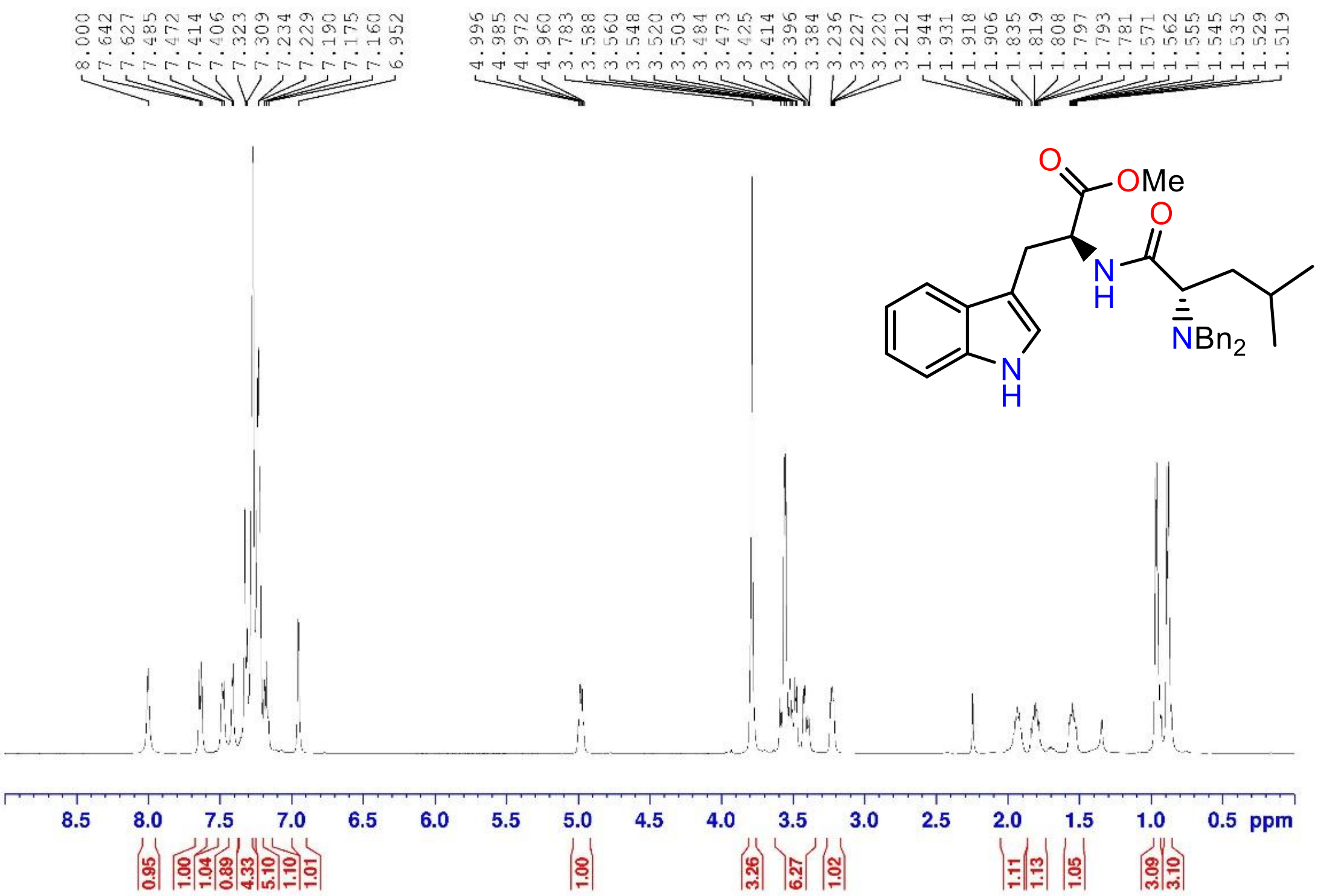

Fig S79 ^{1}H spectrum of methyl dibenzyl-L-leucyl-L-tryptophanate (**8d**)

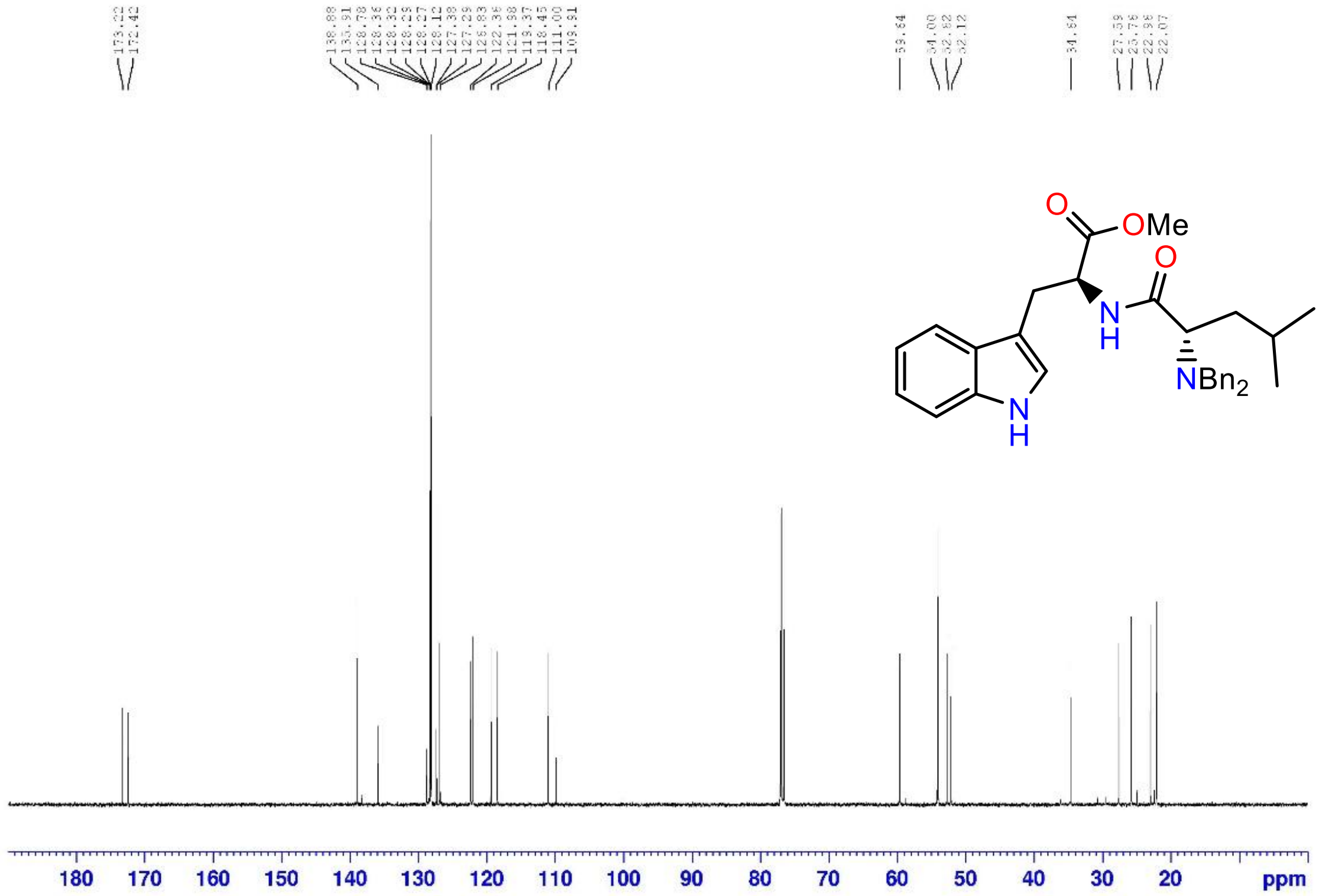

Fig S80 ^{13}C spectrum of methyl dibenzyl-L-leucyl-L-tryptophanate (**8d**)

Vion IMS Qtof (Waters)

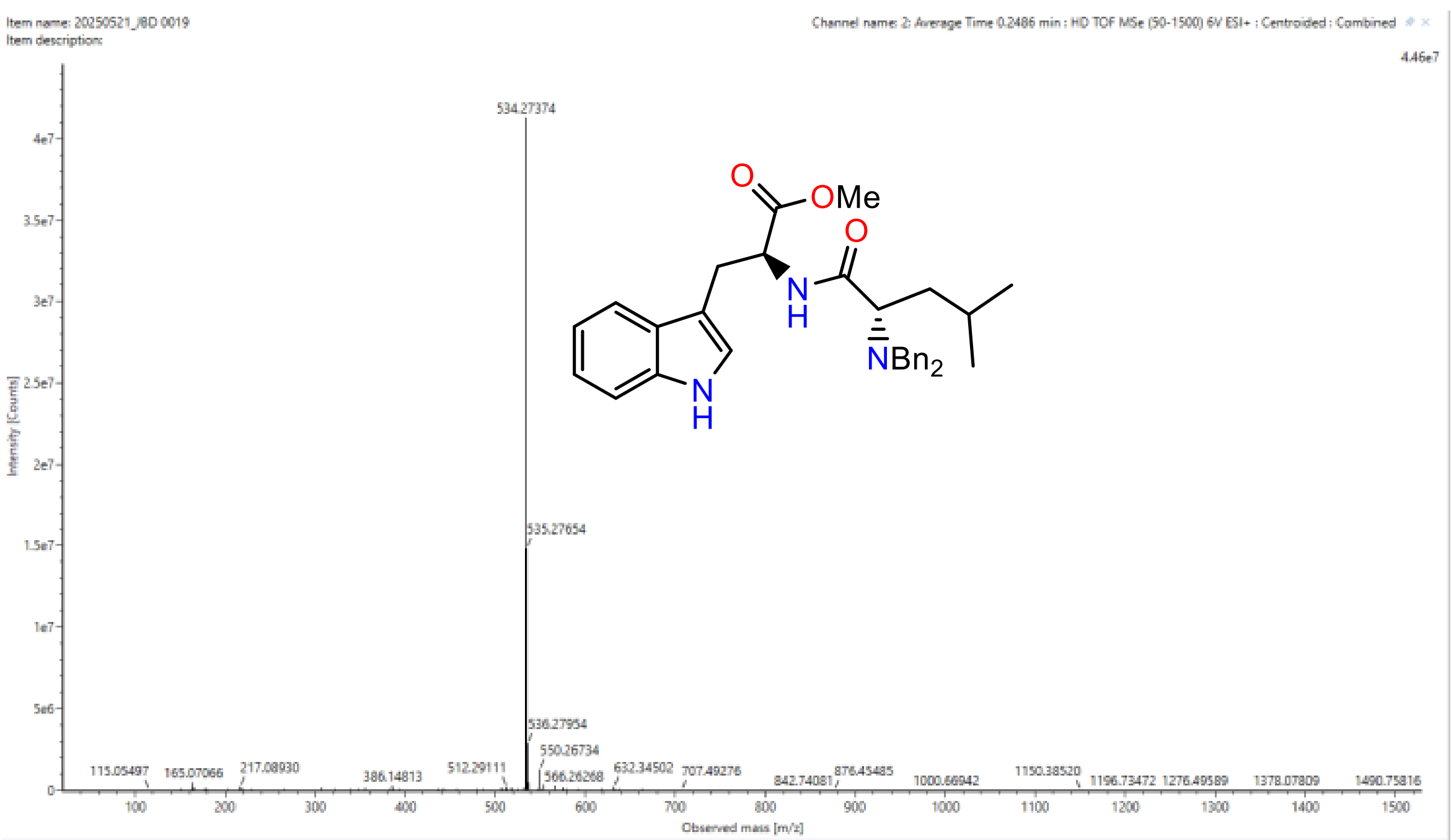

Fig S81 HRMS spectrum of methyl dibenzyl-L-leucyl-L-tryptophanate (**8d**)

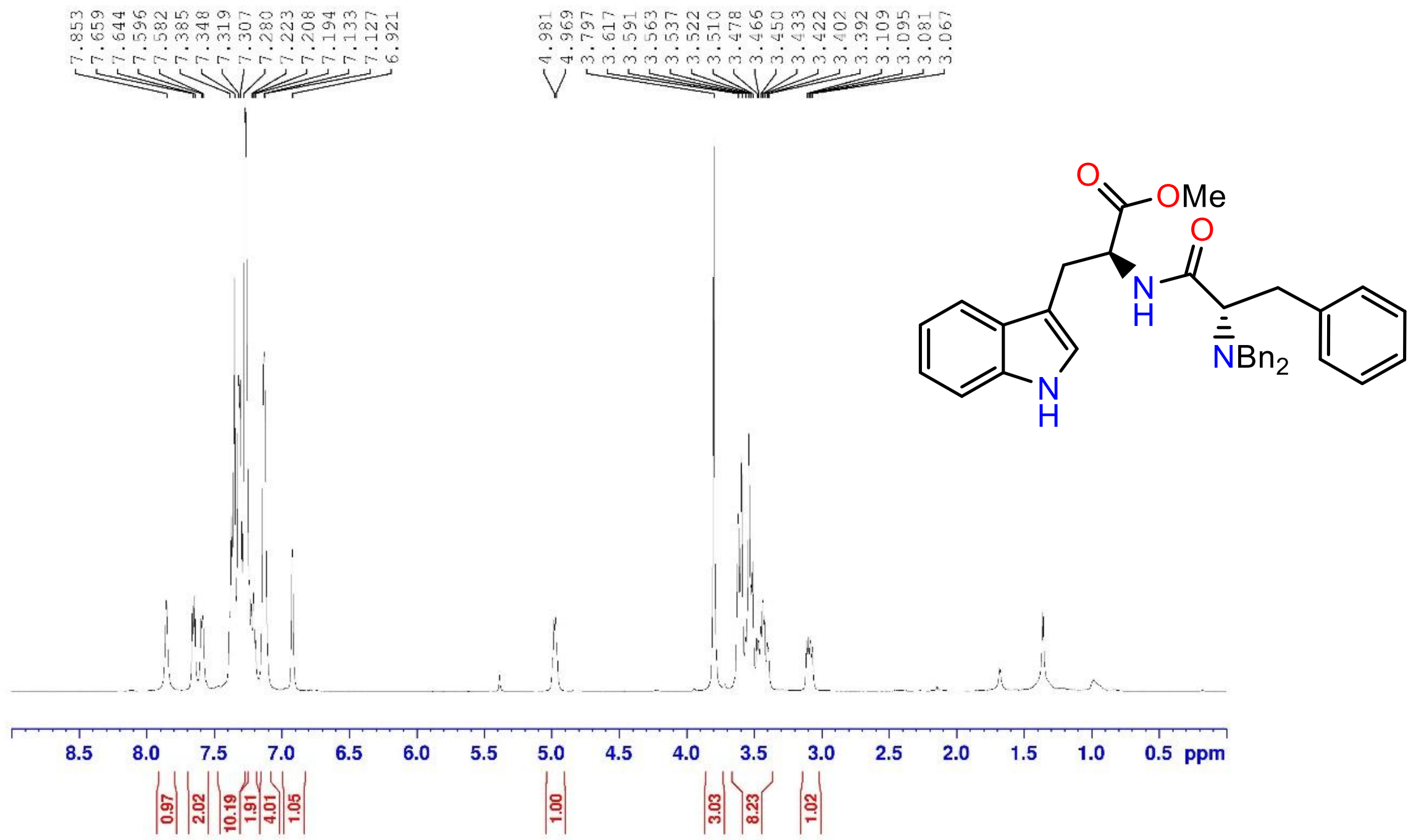

Fig S82 ^{1}H spectrum of methyl dibenzyl-L-phenylalanyl-L-tryptophanate (**8e**)

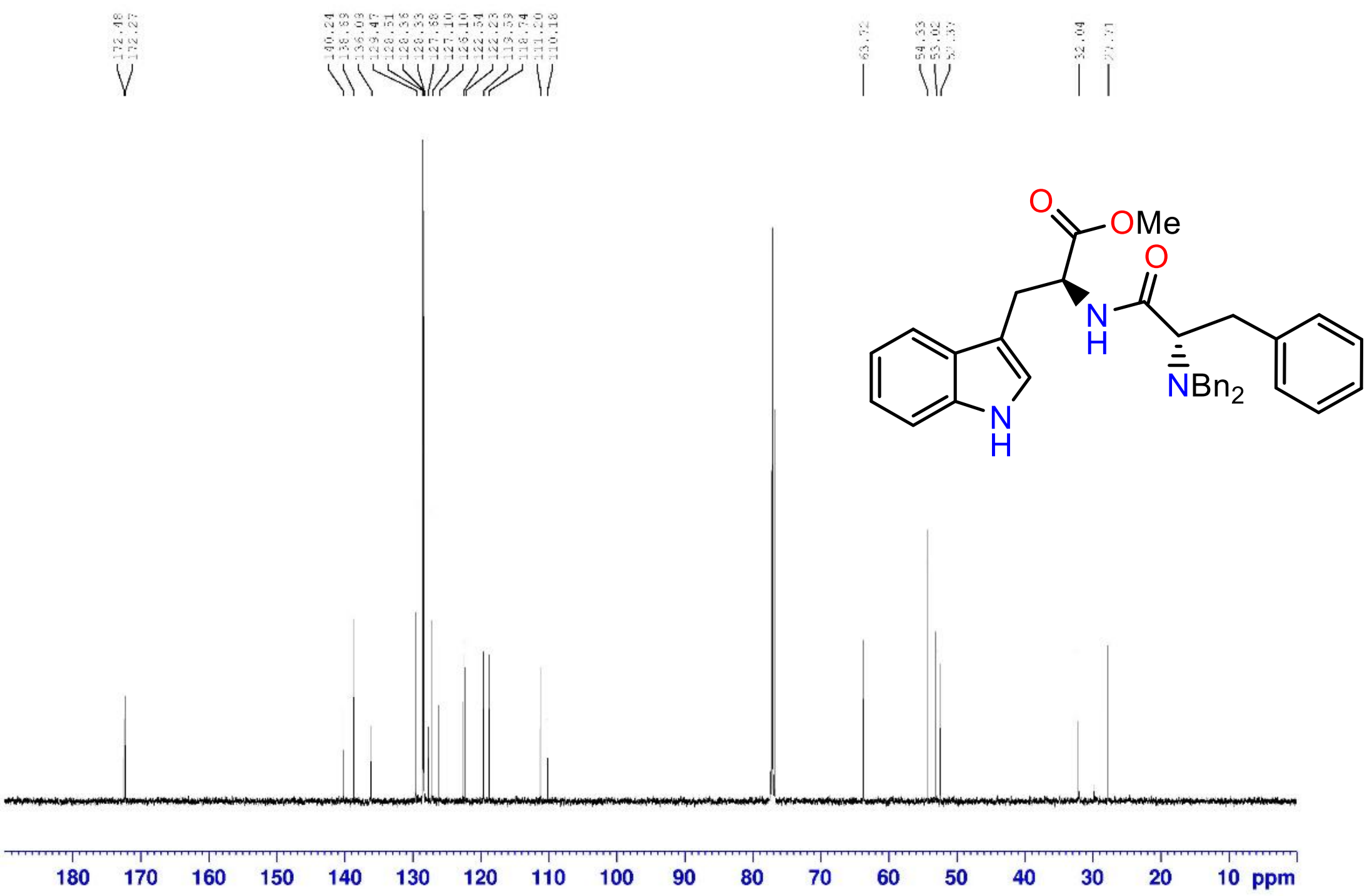

Fig S83 ^{13}C spectrum of methyl dibenzyl-L-phenylalanyl-L-tryptophanate (**8e**)

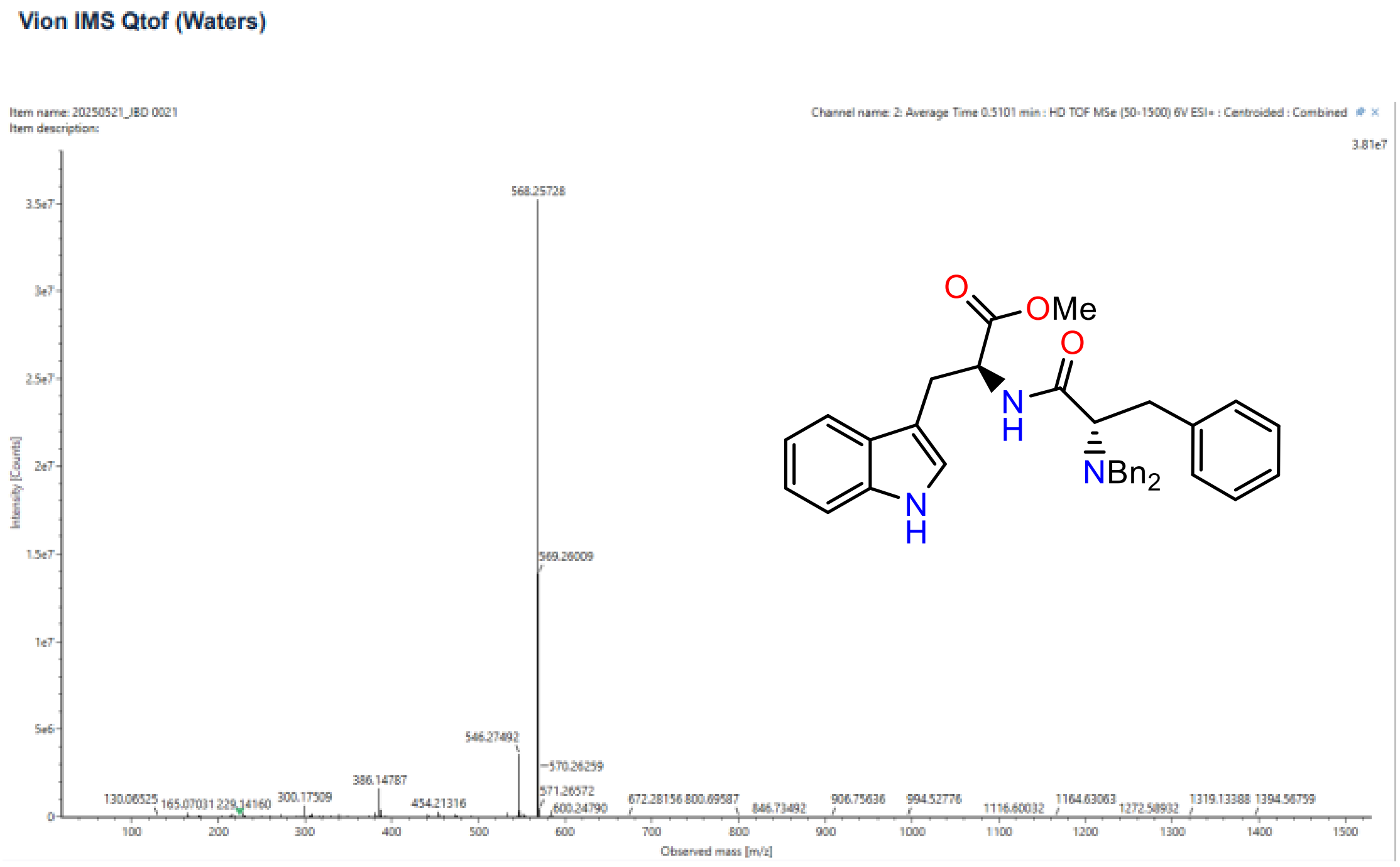

Fig S84 HRMS spectrum of methyl dibenzyl-L-phenylalanyl-L-tryptophanate (**8e**)

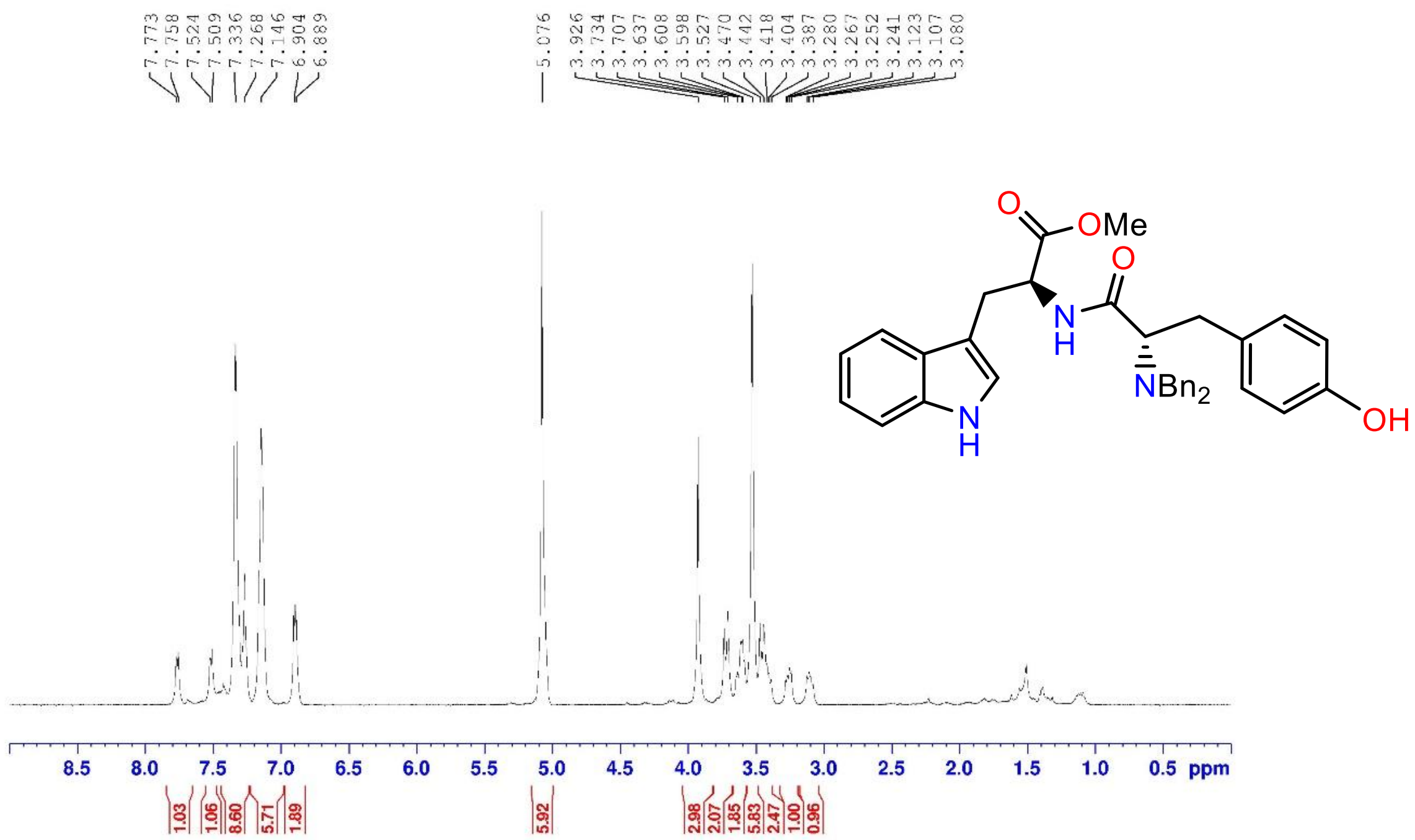

Fig S85 ^{1}H spectrum of methyl dibenzyl-L-tyrosyl-L-tryptophanate (**8f**)

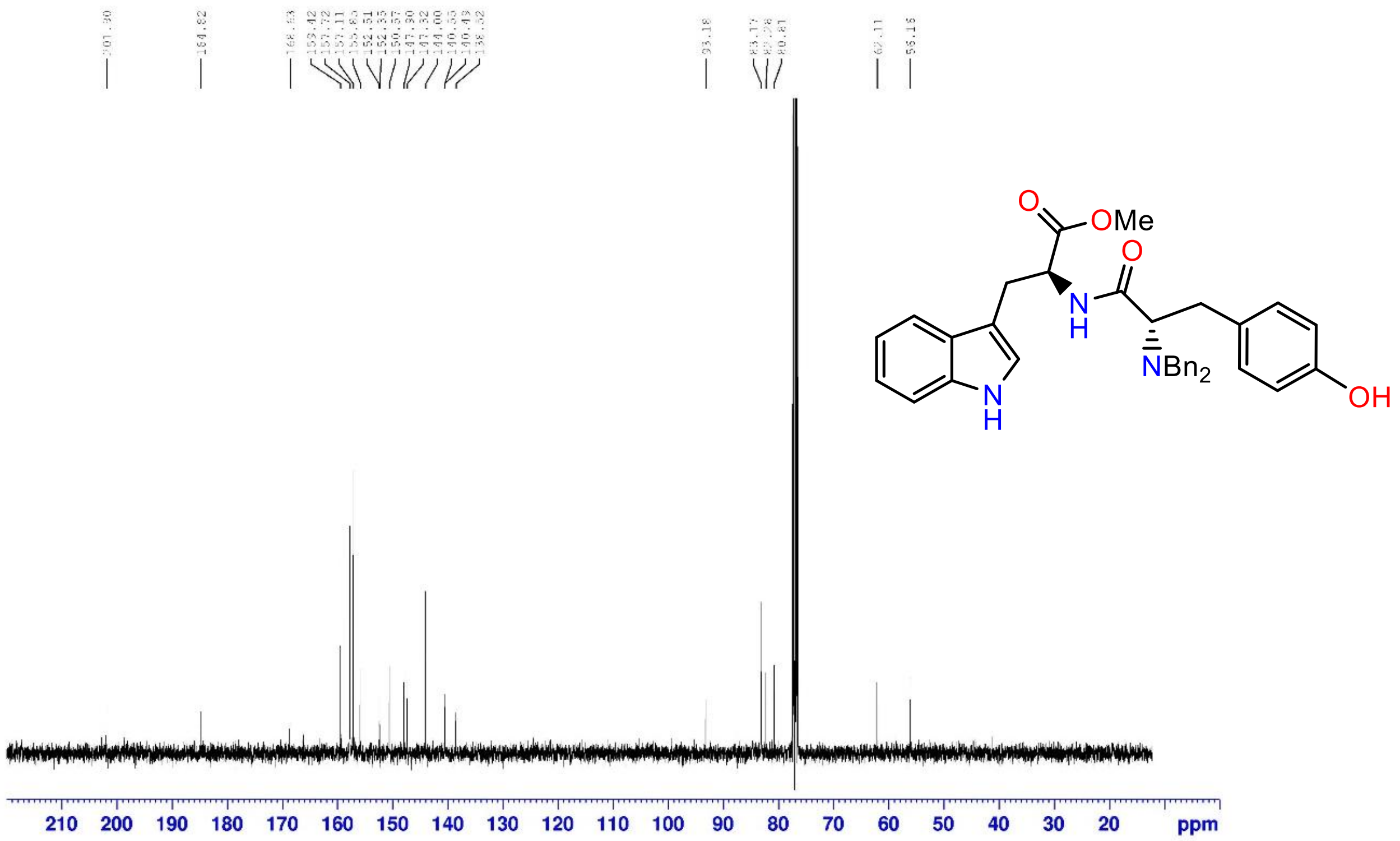

Fig S86 ^{13}C spectrum of methyl dibenzyl-L-tyrosyl-L-tryptophanate (**8f**)

Elemental Composition Report **Page 1**

Multiple Mass Analysis: 2 mass(es) processed
Tolerance = 5.0 PPM / DBE: min = -1.5, max = 200.0
Element prediction: Off
Number of isotope peaks used for i-FIT = 9

Monoisotopic Mass, Even Electron Ions
2569 formula(e) evaluated with 14 results within limits (all results (up to 1000) for each mass)
Elements Used:
C: 0-62 H: 0-66 N: 0-6 O: 0-10 Na: 0-2
20260206 JBD 0022 95 (4.109)
1: TOF MS ES+

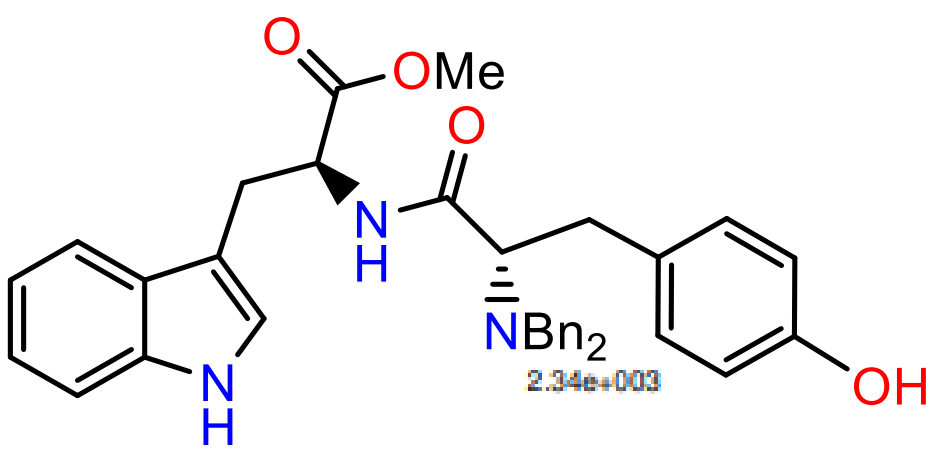

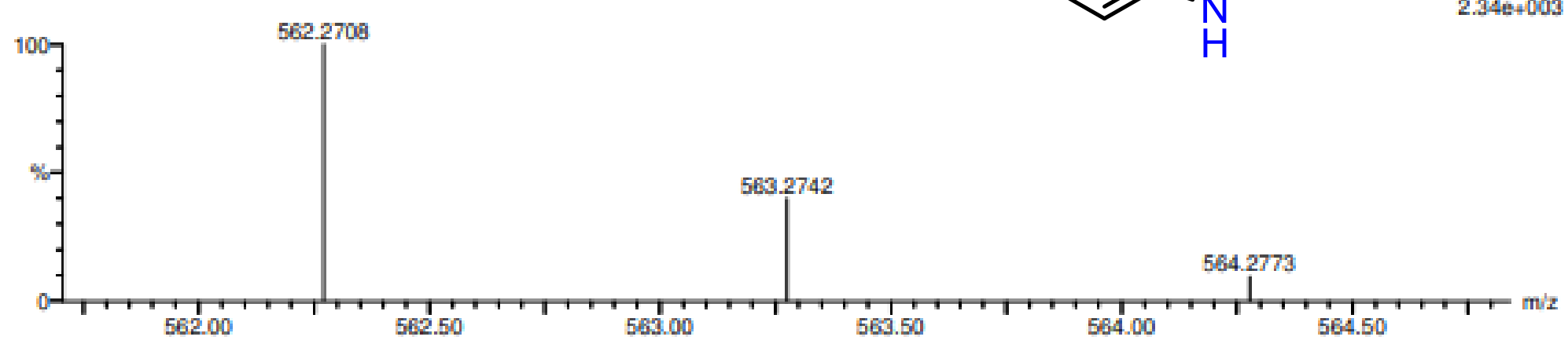

Mass	RA	Calc. Mass	mDa	PPM	DBE	i-FIT	i-FIT (Norm)	Formula
Minimum:	30.00				-1.5			
Maximum:	100.00		5.0	5.0	200.0			
562.2708	100.00	562.2706	0.2	0.4	19.5	37.4	2.3	C35 H36 N3 O4
		562.2716	-0.8	-1.4	4.5	35.4	0.4	C24 H42 N3 O9 Na2
		562.2698	1.0	1.8	17.5	36.9	1.9	C36 H38 N O2 Na2
		562.2722	-1.4	-2.5	20.5	38.4	3.3	C38 H37 N O2 Na
		562.2682	2.6	4.6	16.5	38.9	3.9	C33 H37 N3 O4 Na
563.2742	39.73	563.2739	0.3	0.5	27.5	42.3	2.5	C44 H35
		563.2747	-0.5	-0.9	16.5	41.3	1.5	C31 H36 N6 O3 Na
		563.2749	-0.7	-1.2	12.5	41.6	1.8	C33 H41 O5 Na2
		563.2733	0.9	1.6	11.5	41.5	1.7	C30 H40 N2 O7 Na
		563.2757	-1.5	-2.7	14.5	42.3	2.5	C32 H39 N2 O7
		563.2723	1.9	3.4	13.5	41.9	2.1	C29 H37 N6 O3 Na2
		563.2763	-2.1	-3.7	17.5	42.6	2.8	C34 H37 N4 O Na2
		563.2717	2.5	4.4	10.5	42.6	2.8	C27 H39 N4 O9
		563.2715	2.7	4.8	24.5	43.8	4.0	C42 H36 Na

Fig S87 HRMS spectrum of methyl dibenzyl-L-tyrosyl-L-tryptophanate (**8f**)

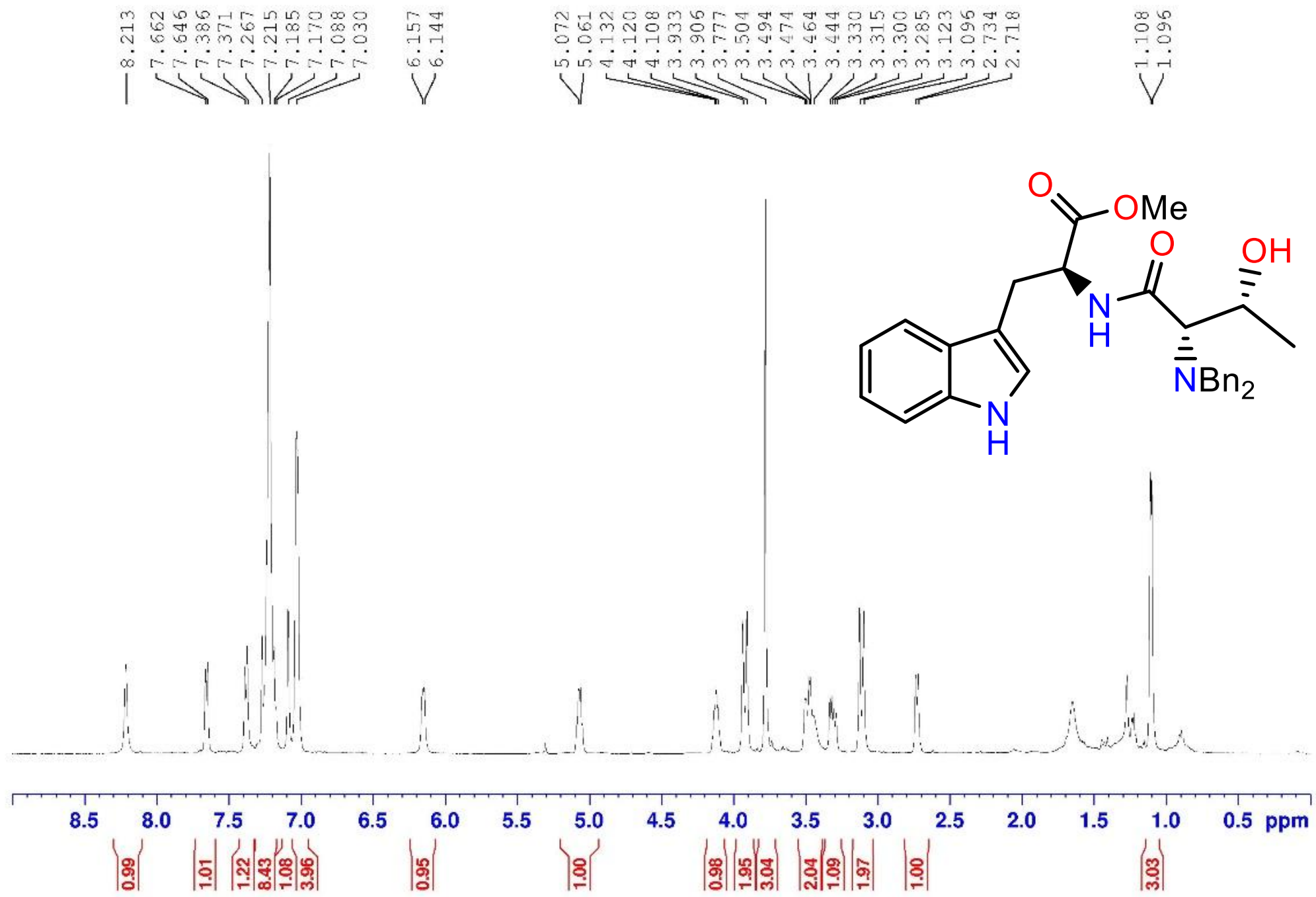

Fig S88 ^{1}H spectrum of methyl dibenzyl-L-threonyl-L-tryptophanate (**8g**)

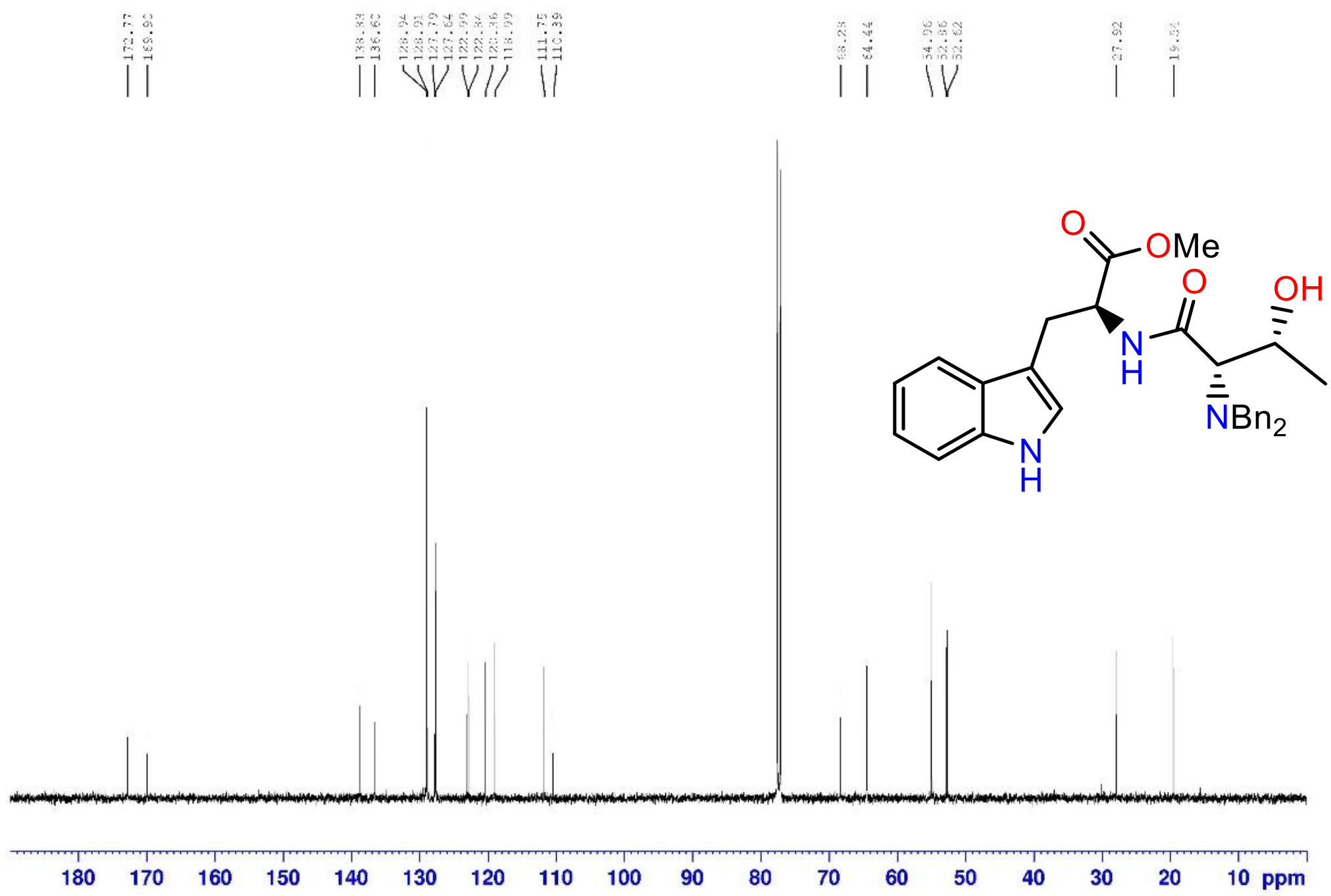

Fig S89 ^{13}C spectrum of methyl dibenzyl-L-threonyl-L-tryptophanate (**8g**)

Elemental Composition Report **Page 1**

Multiple Mass Analysis: 2 mass(es) processed
Tolerance = 5.0 PPM / DBE: min = -1.5, max = 200.0
Element prediction: Off
Number of isotope peaks used for i-FIT = 9

Monoisotopic Mass, Even Electron Ions
2404 formula(e) evaluated with 11 results within limits (all results (up to 1000) for each mass)
Elements Used:
C: 0-62 H: 0-66 N: 0-6 O: 0-10 Na: 0-2
20260206 JBD 0023 89 (3.044)
2: TOF MS ES+

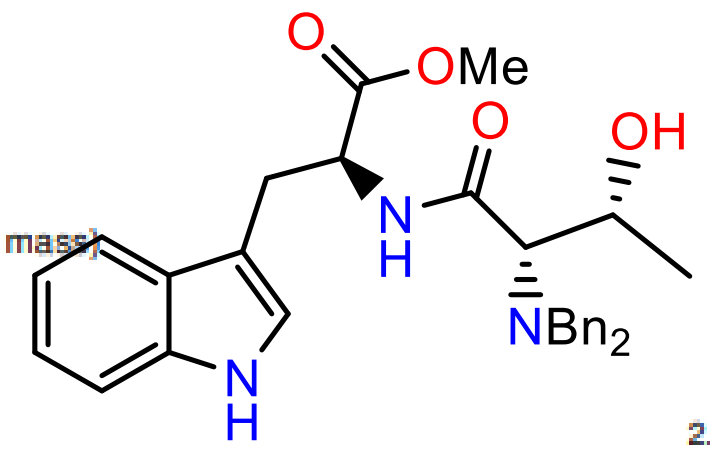

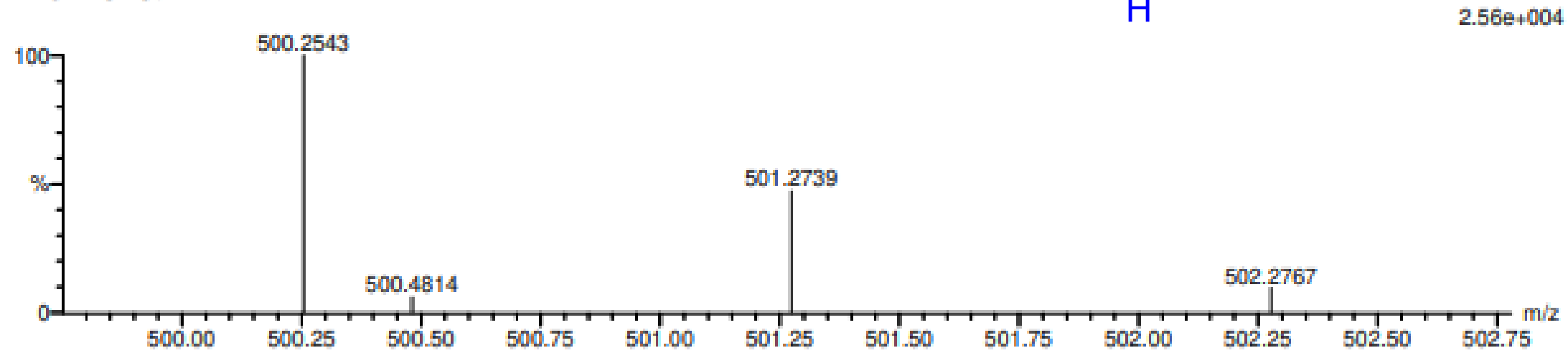

Minimum:	30.00				-1.5			
Maximum:	100.00		5.0	5.0	200.0			

Mass	RA	Calc. Mass	mDa	PPM	DBE	i-FIT	i-FIT (Norm)	Formula
500.2543	100.00	500.2541	0.2	0.4	13.5	82.4	1.7	C31 H36 N O2 Na2
		500.2549	-0.6	-1.2	15.5	83.0	2.3	C30 H34 N3 O4
		500.2560	-1.7	-3.4	0.5	81.5	0.8	C19 H40 N3 O9 Na2
		500.2525	1.8	3.6	12.5	82.5	1.8	C28 H35 N3 O4 Na
		500.2565	-2.2	-4.4	16.5	82.9	2.2	C33 H35 N O2 Na
501.2739	47.11	501.2743	-0.4	-0.8	16.5	56.3	1.0	C30 H34 N6 Na
		501.2745	-0.6	-1.2	12.5	56.8	1.6	C32 H39 O2 Na2
		501.2729	1.0	2.0	11.5	56.7	1.4	C29 H38 N2 O4 Na
		501.2753	-1.4	-2.8	14.5	57.7	2.4	C31 H37 N2 O4
		501.2719	2.0	4.0	13.5	57.8	2.6	C28 H35 N6 Na2
		501.2764	-2.5	-5.0	-0.5	58.5	3.3	C20 H43 N2 O9 Na2

Fig S90 HRMS spectrum of methyl dibenzyl-L-threonyl-L-tryptophanate (**8g**)

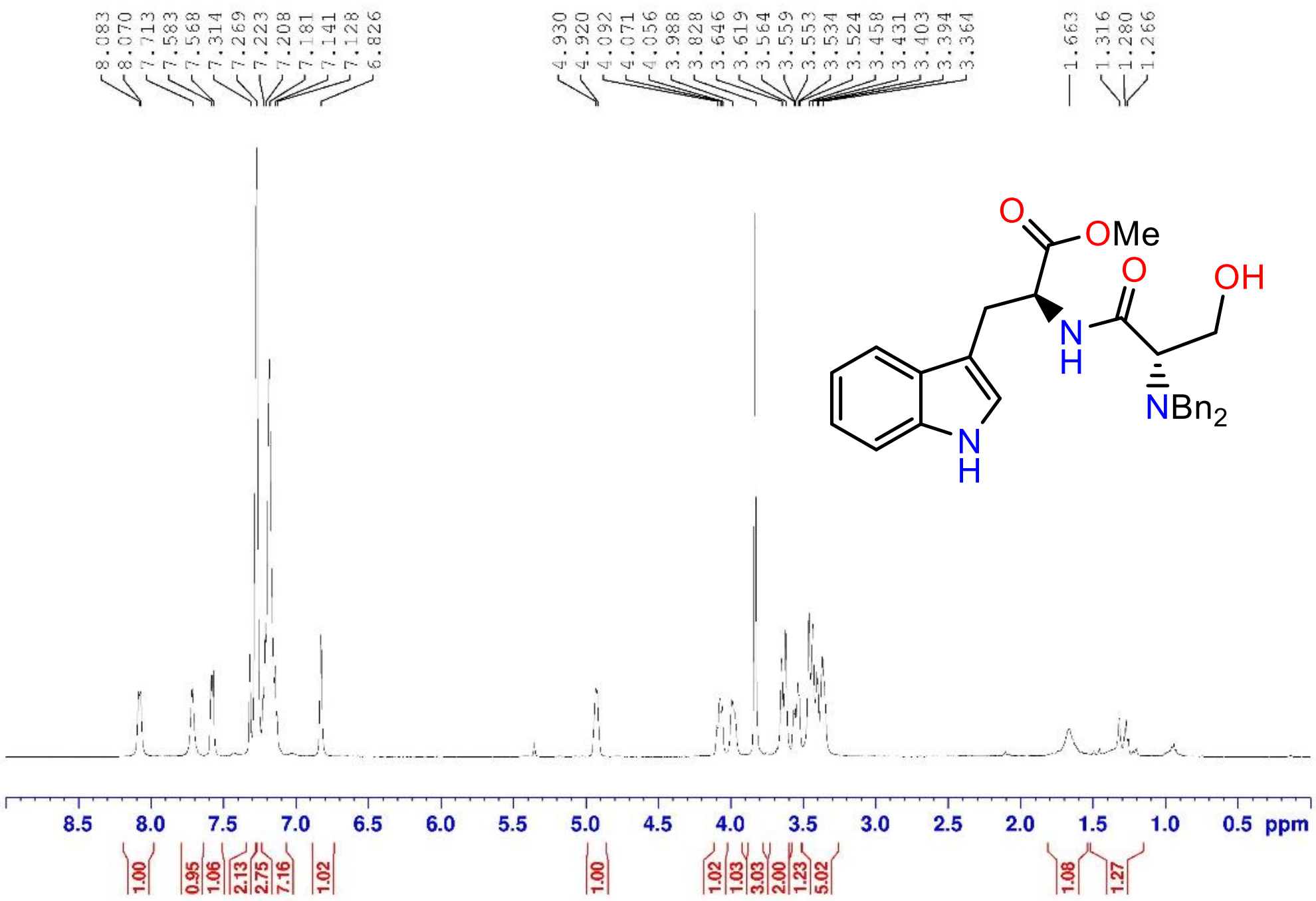

Fig S91 ^{1}H spectrum of methyl dibenzyl-L-seryl-L-tryptophanate (**8h**)

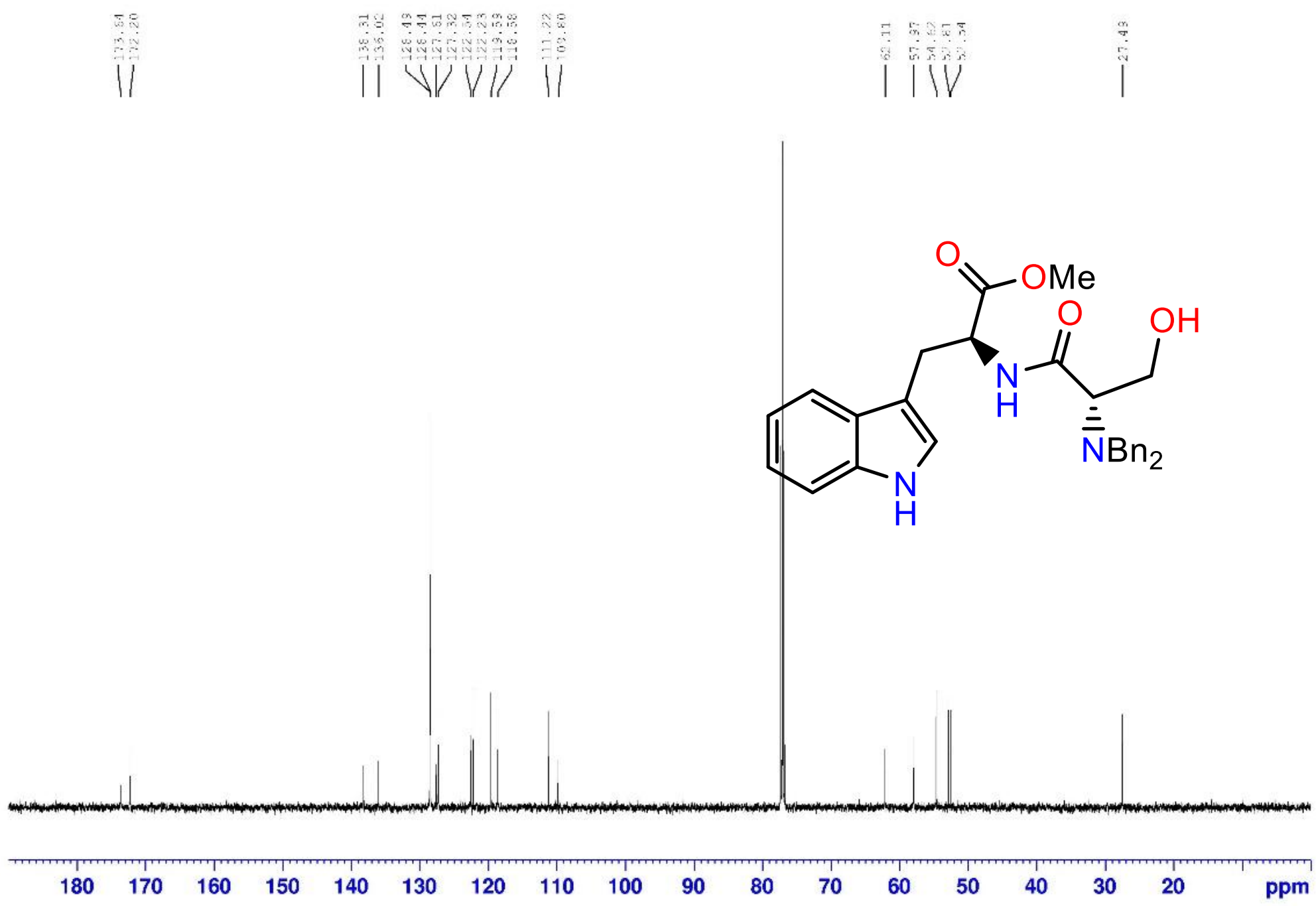

Fig S92 ^{13}C spectrum of methyl dibenzyl-L-seryl-L-tryptophanate (**8h**)

Elemental Composition Report **Page 1**

Multiple Mass Analysis: 2 mass(es) processed
Tolerance = 5.0 PPM / DBE: min = -1.5, max = 200.0
Element prediction: Off
Number of isotope peaks used for i-FIT = 9

Monoisotopic Mass, Even Electron Ions
2358 formula(e) evaluated with 11 results within limits (all results (up to 1000) for each mass)
Elements Used:
C: 0-62 H: 0-66 N: 0-6 O: 0-10 Na: 0-2
20260206 JBD 0024 92 (3.151)
2: TOF MS ES+

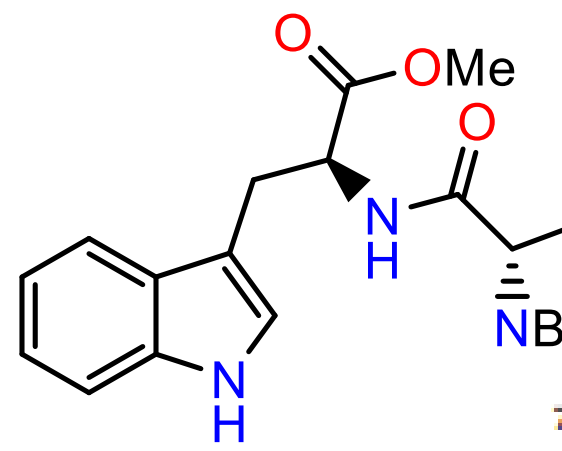

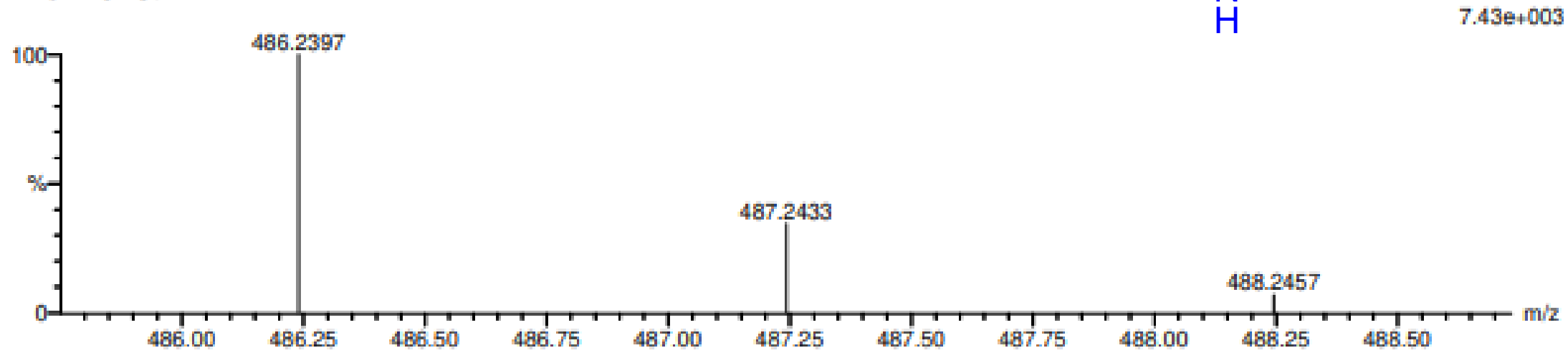

Mass	RA	Calc. Mass	mDa	PPM	DBE	i-FIT	i-FIT (Norm)	Formula
Minimum:	30.00				-1.5			
Maximum:	100.00		5.0	5.0	200.0			
486.2397	100.00	486.2393	0.4	0.8	15.5	44.5	2.4	C29 H32 N3 O4
		486.2403	-0.6	-1.2	0.5	42.4	0.3	C18 H38 N3 O9 Na2
		486.2409	-1.2	-2.5	16.5	45.2	3.1	C32 H33 N O2 Na
		486.2385	1.2	2.5	13.5	44.1	2.1	C30 H34 N O2 Na2
487.2433	34.44	487.2434	-0.1	-0.2	12.5	46.5	1.1	C25 H32 N6 O3 Na
		487.2436	-0.3	-0.6	8.5	47.0	1.6	C27 H37 O5 Na2
		487.2426	0.7	1.4	23.5	47.8	2.4	C38 H31
		487.2444	-1.1	-2.3	10.5	47.7	2.3	C26 H35 N2 O7
		487.2420	1.3	2.7	7.5	47.3	1.8	C24 H36 N2 O7 Na
		487.2450	-1.7	-3.5	13.5	48.1	2.7	C28 H33 N4 O Na2
		487.2410	2.3	4.7	9.5	48.1	2.7	C23 H33 N6 O3 Na2

Fig S93 HRMS spectrum of methyl dibenzyl-L-seryl-L-tryptophanate (**8h**)

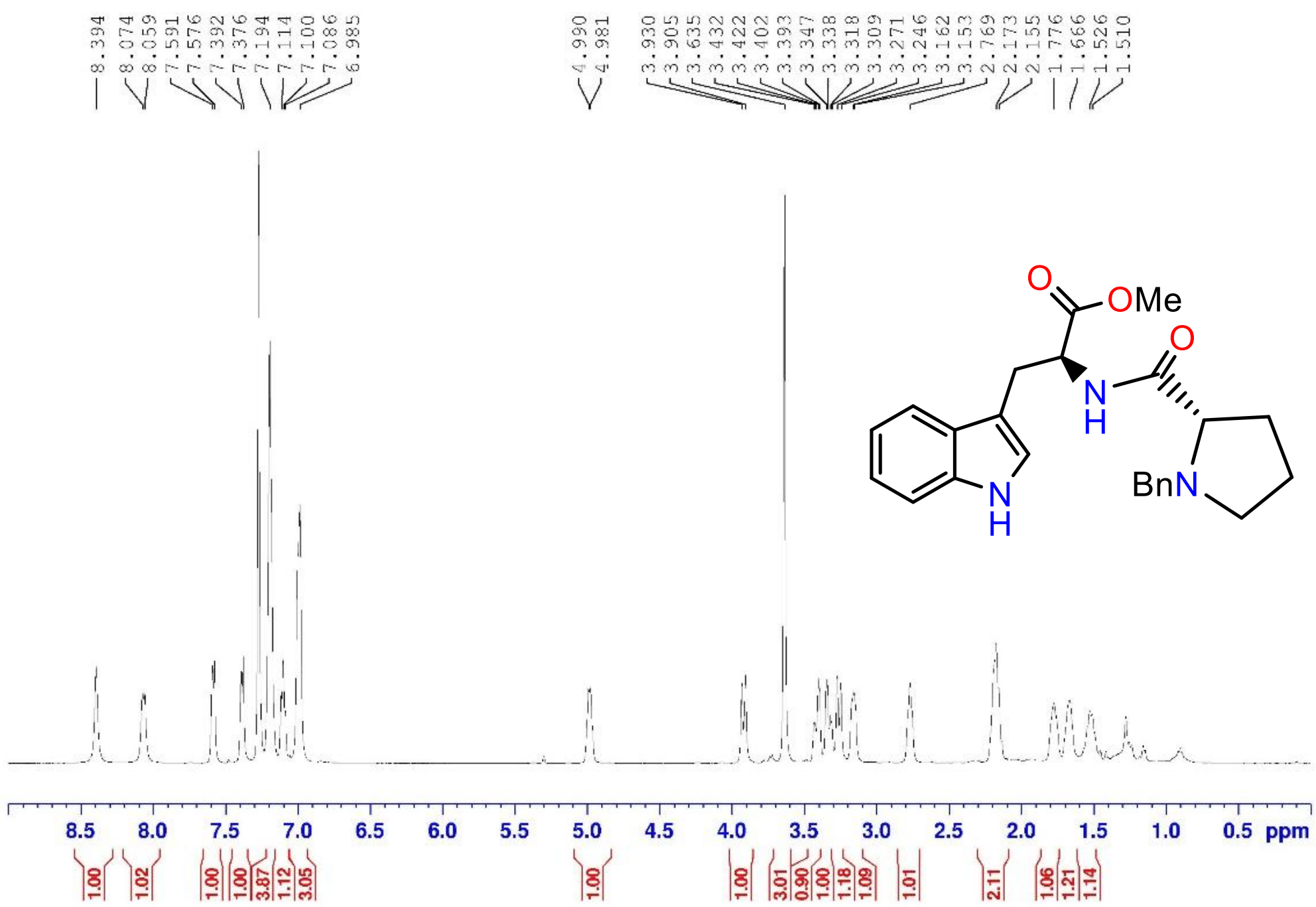

Fig S94 ¹H spectrum of methyl dibenzyl-L-prolyl-L-tryptophanate (**8h**)

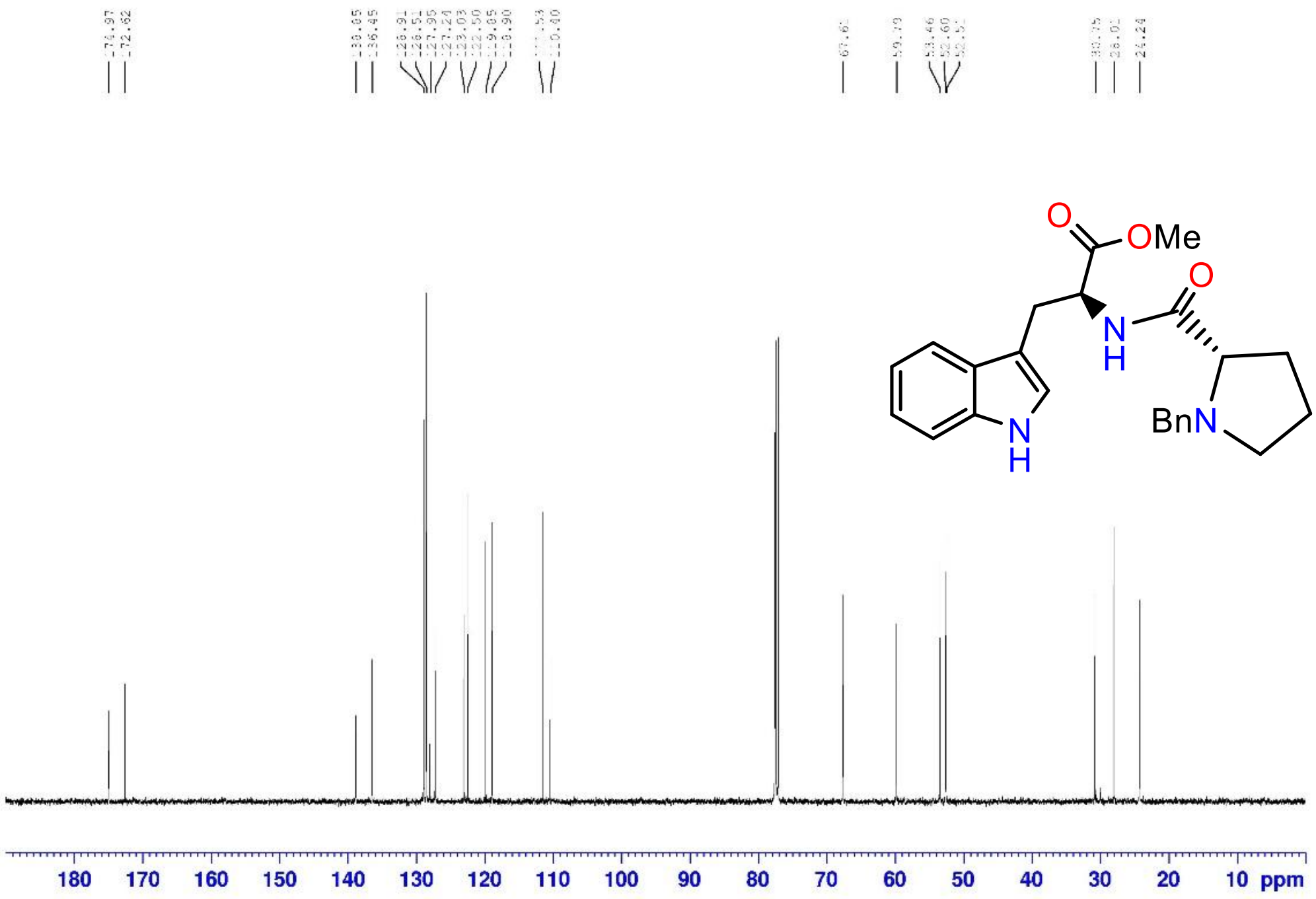

Fig S94 ^{13}C spectrum of methyl dibenzyl-L-prolyl-L-tryptophanate (**8h**)

Elemental Composition Report **Page 1**

Multiple Mass Analysis: 2 mass(es) processed
Tolerance = 5.0 PPM / DBE: min = -1.5, max = 200.0
Element prediction: Off
Number of isotope peaks used for i-FIT = 9

Monoisotopic Mass, Even Electron Ions
2023 formula(e) evaluated with 8 results within limits (all results (up to 1000) for each mass)
Elements Used:
C: 0-62 H: 0-66 N: 0-6 O: 0-10 Na: 0-2
20260206 JBD 0025 58 (1.969)
2: TOF MS ES+

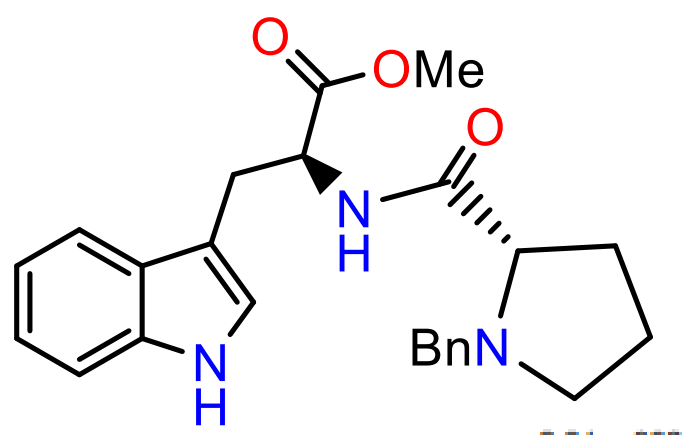

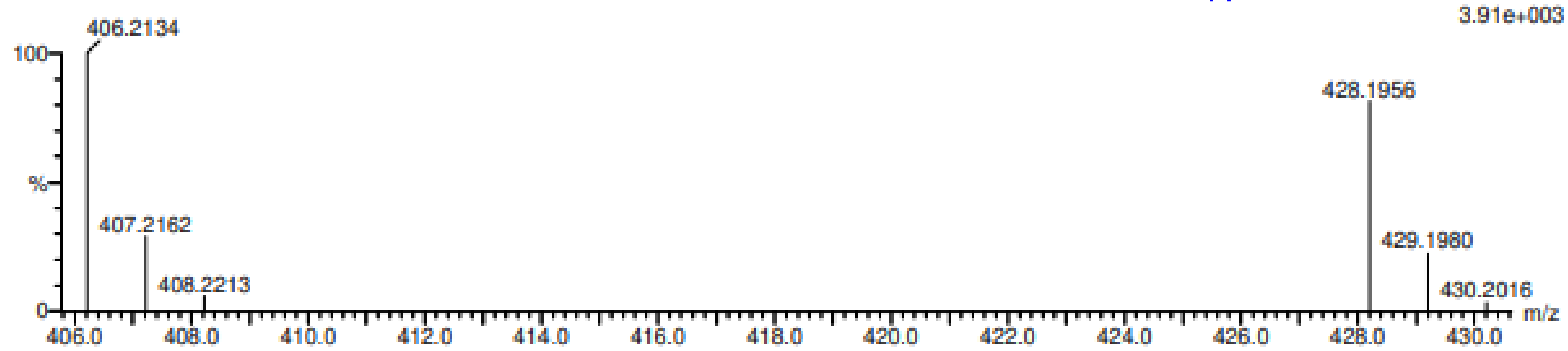

Mass	RA	Calc. Mass	mDa	PPM	DBE	i-FIT	i-FIT (Norm)	Formula
Minimum:	30.00				-1.5			
Maximum:	100.00		5.0	5.0	200.0			
406.2134	100.00	406.2131	0.3	0.7	12.5	33.7	1.8	C24 H28 N3 O3
		406.2123	1.1	2.7	10.5	32.3	0.3	C25 H30 N O Na2
		406.2147	-1.3	-3.2	13.5	34.4	2.4	C27 H29 N O Na
		406.2149	-1.5	-3.7	-0.5	36.8	4.8	C12 H32 N5 O10
428.1956	81.27	428.1950	0.6	1.4	12.5	30.8	0.4	C24 H27 N3 O3 Na
		428.1966	-1.0	-2.3	13.5	31.9	1.5	C27 H28 N O Na2
		428.1969	-1.3	-3.0	-0.5	33.0	2.6	C12 H31 N5 O10 Na
		428.1974	-1.8	-4.2	15.5	34.9	4.5	C26 H26 N3 O3

Fig S95 HRMS spectrum of methyl dibenzyl-L-prolyl-L-tryptophanate (**8h**)
